# Supplementary material for: Enhancing programmatic scale-up: Applying the consolidated framework for implementation research to evaluate decentralized drug-resistant tuberculosis services in Southern Nigeria
Source: PLoS One. 2025 Feb 21;20(2):e0318274. doi: 10.1371/journal.pone.0318274 (PMC11844838; doi:10.1371/journal.pone.0318274)
Supplement: S1 Appendix — (DOCX) [file pone.0318274.s001.docx]

## Key Informant Interview Guide

**Introduction**

Introductions. Explain study background, obtain informed consent and capture participant’s profile.

##### General Questions

1. Overall, what do you think of TB REACH Wave 9 interventions to decentralize DR-TB services?

- What do you like the most/least about (the concept of) decentralized DR-TB services?

1. What (will) has affected/influenced your practice of decentralized DR-TB services in your state?

- What has helped you the most? What challenges have you encountered so far?

##### CFIR Domain-specific questions

| **CFIR domain** | **Semi-structured interview question** |
| --- | --- |
| **Intervention characteristics** | 1. How does decentralized [_] DR-TB services compare with the routine existing programs in your clinic? 2. What adv/disadvantage does decentralized [_] DR-TB services have? 3. What kind of changes did you need to make for decentralized [_] DR-TB services to work effectively in your facility? 4. Are there components of this [_] intervention that should (not) be altered? 5. How complicated is this decentralized [_] DR-TB service delivery? |
| **Outer setting** | 1. How well does decentralized [_] DR-TB services meet the needs of your patients? In what ways? 2. In your facility, has there been a strong need to increase/reduce decentralized [_] DR-TB services? Why or why not? |
| **Inner setting** | 1. What supports are available to help you to adopt decentralized [_] DR-TB service delivery in your facility? 2. What kinds of incentives are there to ensure the implementation of decentralized [_] DR-TB services is successful? 3. How do you think your facility’s culture/setting affects (improves or reduces) the implementation of decentralized [_] DR-TB services? |
| **Characteristics of individual** | 1. What has been your motivation for wanting to help ensure the implementation of decentralized [_] DR-TB services is successful? 2. How confident are you about being able to implement decentralized [_] DR-TB services regularly in your facility? 3. How confident are your colleagues about being able to implement decentralized [_] DR-TB service delivery in your facility? |
| **Process** | 1. How well does decentralized [_] DR-TB service delivery fit with your existing work processes and practices? 2. Can you describe how? Will decentralized [_] DR-TB services replace or compliment the current program or process? |
