# Supplementary material for: Enhancing programmatic scale-up: Applying the consolidated framework for implementation research to evaluate decentralized drug-resistant tuberculosis services in Southern Nigeria
Source: PLoS One. 2025 Feb 21;20(2):e0318274. doi: 10.1371/journal.pone.0318274 (PMC11844838; doi:10.1371/journal.pone.0318274)
Supplement: S1 Table — (DOCX) [file pone.0318274.s002.docx]

|  | **INNOVATION CHARACTERISTICS** | **OUTER SETTING** | **INNER SETTING** | **INDIVIDUAL CHARACTERISTICS** | **PROCESS** |
| --- | --- | --- | --- | --- | --- |
| **ENABLERS** | **1. Innovation Relative Advantage** i. **Faster notification of diagnosed DR-TB patients**  *WhatsApp helps us to know as early as possible if you have a patient… As soon as the result is out, you get the alert, and you will be able to contact the patient as early as possible. (A016)  It is good because before we have the hard copy of the result you can see the result through the WhatsApp group, so so [patient] name is there, so we start calling his or her number so that we start the baseline, so that we will not delay the treatment. (B010)* ***ii. Brought treatment closer to the people*** *Initially, our patients used to go to state headquarters for treatment, but now, with the help of TB Reach Wave 9, treatment is closer to the people. Some patients don’t like going out of their community to receive treatment; they used to feel as if they would not come back again. (A010)  This one [decentralization of DRTB services] is better compared to the one we were doing before because now the treatment center is very close to the patients; They no longer go through a lot or too far to access their treatment unlike before when everything was only at the state capital. (B018)  i****ii. Enhanced tracking of patients*** *Yes, like I said earlier it influenced us to keep track of the patients. It's not as if we didn't do tracking before, but because we are meant to since this intervention is on ground, you can't say that there has been a notification since two weeks ago and you didn't pay attention to it. (B02)*  *“As a result of this decentralization program, we were given [patients'] sputum examination form that has the next of kin name and the next of kin phone number... So, it helps us so much to track our patient immediately after the result is out by making provision for an alternative phone number. (A015)* ***iv. Ease of conducting baseline investigations*** *So, you know this decentralized program has been able to provide a facility, in fact, in every zone they have a center where they can take the baseline [investigation] easily without travelling miles. So, it has really contributed to very good improvement in DR-TB service delivery. (B020)* ***v. Reduced transportation barriers by reducing cost of transportation*** *The decentralization help prevent something like out-of-pocket expenses, in the sense that people need not pay a huge sum of money to go for baseline investigations. Although the treatment is free but people were spending a lot of money to go and access care at a very far place, so that aspect is no longer there. (A01)  …for the patients, it reduced their transportation cost, and reduced stress also. One can easily walk down and get drugs instead of taking a vehicle to the capital of the state.’ (B011)* ***vi. Empowerment of the health workers*** *“Yes, yes we can compare and one is better than the other. The decentralized approach is better, it has performed excellently more than the routine one. In the routine system we had perpetual problems and challenges… but this one is better because it empowers the TBLS with information to act and encourage us; it empowers the patients too.”(A013).* ***vii. Improved counselling using DR-TB survivors***  *“Counselling is part of the advantages; we invite the survivor to come and talk with them. So, when they see that somebody has been treated before, and that the person has been cured after taking the drugs, the patients will know that they are not the first to experience DR-TB. Improved tracking with the CBOs is another advantage too. (B012)* ***2. Innovation Complexity i. Intervention was not complicated in any form*** *“It is not complicated because it is actually the same thing, (diagnose DR-TB patient, do baseline investigations and treat) we have been doing; only that it is no longer done in one centre but in different centres. It is the same thing and it has not been changed. Only that everybody is not coming to one centre now. (A017)* ***3. Innovation Design i. Unique role of VLO*** *Yes, the VLO will be calling, in fact that has pushed us (TBLSs) to do more, to do what is needful… (TBLS)* ***ii. Use of DR-TB survivors as counselors*** *‘Yes, if you ask me the highest point of wave 9 intervention was the use of survivors in the counseling process, to me, it was a tie-breaker. Because once you see someone that tells you I have been through this thing you see that there is nothing there, I think it actually meant a lot to some of our patients. I think it was a game changer.’ (B01).* ***iii. Involvement of private sector*** *What I really appreciate most is the incorporation of private sector laboratories because Government cannot make it alone, they cannot do it alone. (B03)* ***iv. Tracking of patients*** *“I like everything about the intervention, especially the follow-up and the tracking aspect because we are also involved in tracking TB [not] just to follow up. We also work on tracking them.” (A020)* ***v. Use of WhatsApp platform*** *What I really like most is the aspect of prompt alert. Based on the result that is posted on the WhatsApp group, the VLO will immediately intimate the TBLS and other Team members to be able to contact the newly diagnosed DR-TB patients on time whenever we receive the alert that so-so person in your LGA is a DR-TB patient. So, this person has to go for baseline test so, that was the real thing that really- really impressed me during the wave 9 intervention. (B020)* | **Critical incidents** i. **Removal of fuel subsidy affecting cost of transport** *‘So the transport support was a bit okay before, but this fuel matter [fuel subsidy removal] has worsened the whole thing. Now a lot of patients are also hungry that if they do not have money, they will prefer buying food to eat with that transport money and forget about the care for DR-TB, that’s why I was saying that to further decentralize will be good but there should be training of more health workers.* (A05).  ii. **Prevailing economic difficulties** *‘So, for the patients I don’t know how we will handle this issue of transport. A lot of the DR-TB patients have complained that the money given to them for transport is not enough. This is because most of them if not all the patients are poor and you cannot blame them. Almost all the patients that have been diagnosed of DR-TB are poor. So, this transport matter is a real problem. (B05).* | **1. Structural characteristics  i. Existing physical infrastructure and health workforce** *Well, because in the different zones of the state; we have very good hospitals there… because they have doctors, nurses, midwives. That made the implementation easy.* (A04)  *We were able to adjust to decentralization because we have the structure, also we have the man-power* (A07)  *This facility has already been known for TB diagnoses and treatment, so DR-TB was not much different. So, with the facility that has been doing TB care for years when the DR-TB treatment came in it was almost the same*. (B05)  ii. **Information Technology Infrastructure**   *That platform is the DR-TB WhatsApp platform, yes, so with it, you know the results of GeneXpert test somehow in real time and that is very, very important, unlike before, when a sample is processed and a result is available, it will take days or weeks before one can get that result. That is one of the most important achievements for me. (A05).*  *Even if somebody receives alert and it’s not you, the consciousness that this is a DR-TB patient there is a WhatsApp group that we have created and they bring it there. These things were not done before so the person will send it to the group and say look, I received this alert for this patient…the patient is quickly tracked and placed on treatment. (A03)* ***2. Culture*** *i.* ***Consistency and passion***  *Consistency of the State TB program, ever since I joined this TB program, they have been very passionate and consistent. That alone actually motivates me. (A07).*  ***3. Available Resources \| Materials & Equipment***  *i.* ***Resources for the implementation are provided*** *Since this TB Reach Wave 9 came on board, it has improved our work because what we need for rendering services to the patients... The organization, (RedAid) provides examination request forms; then we have all the PPEs; we have plenty of it, like nose masks and hand sanitizer, so now we have enough PPE. (A014)*  4. **Access to Knowledge & Information i. Regular training of health workers** *We kept training and re-training our health workers at the local level. On some occasions I will invite the State Program Manager to join in the training. This is because I noticed that when both of us [the State Program Manager and I] talk, it brings more confidence to the health workers concerning the program. That helped us very well.* (MS IK)  5. **Tension for change** i. **Patients used to refuse treatment because of distance to DR-TB center** *Why I said so is because even the patients that were with me 3 or 4 years ago, they refused going to the state capital for their drugs and treatment; sometimes they asked me to get the drugs for them even when there is need for the patients to go there for physical examinations; so when the services were brought here for them to be coming here, they [patients] were so happy.* (B012)  **ii. *Patients experienced long waiting time during baseline investigations before decentralization*** *Before now, when we diagnose DR-TB, it will take time before we have baseline investigation results... So, it was good that baseline investigation is decentralized. In-fact, it should be decentralized the more*. (A018)  iii. **Transportation challenges hindered patients’ access to care prior to decentralization** *No, the former method wasn’t good, everyone going to the capital was hard… Sometimes we just charter a motor to go to the capital, which was difficult, we have done that before, we charter a taxi, it was very costly. It was difficult but you can charter a bike and move to the zonal facility and come back.* (B013)  iv. **Health workers felt frustrated due to high rate of pre-treatment loss to follow-up before decentralization of services** *It is discouraging when so many of our diagnosed patients were lost to follow up because they will say who will make the payment, who will do this, but in my state, we have another laboratory now due to the decentralization, so they go there with ease*. (B014)  6. **Compatibility i. Aligns well with our organizational vision** *It fits in perfectly [with our vision] because we have been dealing with TB patients before now. This [decentralization of] DRTB services makes it easier for us because we are used to it; be it counselling; educating them on what they should eat, on what they should do and the precautions. So, it didn’t affect us in any way, it just made us improve more and more*. (B015) | **1. Capability**  i. **Confidence and self-efficacy**  *I have confidence in doing it because we have been dealing with DS-TB patients before so when it comes to the DR-TB patients it made it easier because we have been trained for it and we are doing it regularly we are doing it almost every day so we are used to it and the confidence is there, we are competent to do it.* (A03)  *I am confident because it’s part of my job. I have been doing it before; I have been doing TB management since 2006. And I have gone to so many trainings so I am used to it. That gives me confidence.* (B015)  *I’m confident about my work. I supervise laboratories within the state, so I try to do my job with confidence. Morning, afternoon, and night, what we do in our facility is tuberculosis, so with that, I don’t have a division of attention.(B016)*  **2. Opportunity**  i. **Syncing with routine program practice**  *…There's no blessed day we will not come in contact with a TB patient. We are confident in working with them and the program has been wonderful.* (A020)  *I was able to fit into the counselling services for DR-TB patients through the knowledge and experiences I derived from the health workers that offered treatment services to me of which counselling was one of the services I received.* (B08)  **3. Motivation** i. **Passion for DR-TB service provision** *Job satisfaction when you see whom you have treated for DR-TB and he is getting well that alone is an incentive* (A011)  T*he motivation is that I love my patient to get treated quickly and be okay so I welcome the intervention program… I support that initiative and the program helps me to contribute my own quota to the eradication of the disease by calling the patient to get their treatment on time.* (B018)  *Yes, what has been my motivation is that first of all the number of patients that are lost to follow up has reduced. That’s number one. Secondly, we were able to monitor our patients very well compared to what it used to be in the past. All these things put together have been a kind of motivation that if they are doing this decentralized TB care our treatment outcome will be a good one. (*A01)  *Yes, the love for the patients is very much important; we love our patients to get well so that they will not transmit the infection to other people, but the fact that we are paid for the services also motivates me*. (A06) | **1. Planning** i. **Outlined specific steps and workspace** *“… we now discovered that there is a need for rearrangement of our place in the sense that we rearranged that if any DR-TB patient comes he sits here. Then there is a need for triage, such that the DR-TB patient who comes for drugs does not waste time.” (A012)*  ii. **Appointment of desk officers** *The changes we made is that, for DR-TB/patients, I appointed a desk officer that was in charge of the DR-TB treatment services in the facility.* (B05) |
|  |  |  |  |  |  |
| **BARRIERS** | **1. Innovation Design i. Verbal autopsy perceived as stigmatizing by the late patient’s relatives** *I can say that verbal autopsy should be modified or dropped. There are some patients that died, by the time we were asking the relatives what really happened, at times they felt as if we want to stigmatize them.* [A015]  *ii.* ***USSD not considered as priority for the program*** *The entire components of the intervention are good, but we have to put it in order of priority as I said earlier, the ones that need to be changed are USSD, and verbal autopsy. These ones are not all that important to the program [A012]*  *iii.* ***Inadequate utilization of the project DR-TB IMS platform.***  *That innovation, I have engaged it so much, right from during primary training and now the implementation. Immediately I complete my tests, I simply go to the DR-TB IMS platform and key in my results and with that all participants could view the results in a matter of minutes of inputting the results in. So, it is a very good innovation, I have challenges concerning it. The only challenge is that I understand that some of the key actors in the program were unable to access the IMS platform and that warranted after inputting it into the DR-TB, IMS platform, I will also snap the results and send directly to the key people involved via WhatsApp. (B06)*  **2. Innovation Cost** i. **Prizing of baseline tests** *Challenges in that aspect came from the fact that some of the tests that I was expected to do, I was not able to do them. I had to go the extra mile to get the materials so as to perform the tests and more importantly too, the pricing was far far below the general pricing in the private sector and it became worse when the economic difficulties emerged last year; the cash crunch, then the prices of the reagents doubled and tripled but we were still asked to charge as low as what we were asked to sign for. (B06)  ‘We have people conducting the investigation tests but their prizes are not friendly; so like in one of the decentralized centers, there is no lab there for now, so we still have challenge in that area, so if we have any patient in that zone you still need to bring the patient down to the state capital for the investigation test.’* (A02) | **1. Critical incidents i. Inadequate laboratory facilities and services in some rural areas** *Yeah. We have challenges, and some of those challenges are that we do not have comprehensive services in some of our rural areas. This makes it difficult to decentralize in such areas because of the limited services they provide. We still need to bring them to town, but one way or another, we have had support to provide transport allowances.* (A014)  *Another thing is the unavailability of those services in those places, yes, we get clinics but they will not offer the services that we want and they are not ready because the number of people we get there are not many so for them to incur the expenses of the services to them is not profitable. (A04)  ii.* ***Poor power supply*** *Occasionally some labs will not work for some days maybe due to power failure or maybe the weather is bad, the solar panel is not charging enough or maybe there is a breakdown in AC but things are getting improved.” (B03)*  *“Some of our GeneXpert sites the solar panels do not charge the batteries… So, there are may be samples but when the battery is low the laboratory will not be able to work so we have to wait until when there is enough sunrays for the solar panel to charge. (B03)* | **1. Access to knowledge and information i. Non-training of health workers** *In decentralizing, you need to build capacity of health workers, so some of these health workers outside the state capital that we visited have little or no capacity in the management of DR-TB; that too was a disadvantage. You know we [the national program] didn’t train them on DR-TB; I mean proper training on DR-TB but because it has been decentralized to those places; we need somebody to stand in; at least a doctor to stand in to see these patients and attend to them; so, this was one of the disadvantages of this decentralization.* (B02) | **1. Motivation  i. Fear of being infected with TB among health workers** *There is also the issue of being exposed to TB, when the members of staff in my private lab realized that we were having DR-TB patients, they were frightened to the stage that when the patients come, they will prefer that I should handle them alone, it was really challenging*. (B06)  *Well, in every situation, there must be apathy; there must be apathy to issues. For example, not all doctors are interested in TB services especially DR-TB services; because they know someone could contact the disease while treating it.* (A04) |  |

Table 1 above shows the quotes for all the domains
