# Supplementary material for: Enhancing programmatic scale-up: Applying the consolidated framework for implementation research to evaluate decentralized drug-resistant tuberculosis services in Southern Nigeria
Source: PLoS One. 2025 Feb 21;20(2):e0318274. doi: 10.1371/journal.pone.0318274 (PMC11844838; doi:10.1371/journal.pone.0318274)
Supplement: S3 File — (DOCX) [file pone.0318274.s004.docx]

**KII/A1**

| **Interviewer/facilitator/questions** | **Respondent/responses** |
| --- | --- |
| Thank you very much, sir, may I start by asking you what you think of TB reach wave 9 intervention/ Decentralized DRTB services? | Ok, I think the TB Decentralized DRTB program has been wonderfully well because in terms of the services they provide and in terms of making services more convenient for DRTB to access. I think it's more wonderful and very accessible for the patients to access their drugs and services easily, especially in the aspect of the case line, baseline examination. So, you know this decentralized program has been able to provide a facility in fact local government root for DRTB in a remote area to access their baseline in a more easy way rather than before where they have to travel some mile before they take their case line. So, it has real contributed very good improvement |
| Ok, is there something you like most about the intervention? | No, I'm just talking about one of the challenges that it solved very well and other things. |
| Ok, I’m asking if there is another thing, if there is something you really like so much about the intervention. | About the intervention? |
| Yes | I like everything about the intervention, especially the follow-up and the tracking aspect because we are also involved in tracking TB just to follow up. We also work on tracking them. So, I really like the way they handle the program, you know calling, making sure that all the patients are being enrolled on time and everything. So, I like that also, at least it also called the attention of everybody that this DRTB patient needs to be taken care of and they have to access treatment in order not to spread to other community members and households and everything. So, I really like everything about the program. |
| If you were asked to rank, is there anyone in order of rank? Are there any of the components you would like to come last? | Talking about the rank, you know when you're talking about ranking, there is no intervention that is supposed to come last because if you're to remove anything from the program it will affect the other activities and then the outcome of the patient is what we are looking for So, I will not rank anything less; everything should be at the top. |
| So what influences your practice of decentralized DRTB Services in your state? | I think what influences it is because you know before we have been in the program that has been so long and then during when they were practicing the routine program. You know if you have a patient in Ogbomo-Osho for instance now you have to travel down to Ibadan to access the baseline and everything and the difficulty is there also. Apart from that the transportation of the patient, you know most of this DRTB let's just say the reality that most people that these DRTB diseases affect a lot are those people that don't have enough to eat also. They are the extremely poor people that this thing affects. So, when they are coming to access our treatment, they have to carry a pot of money for transportation to access the baseline and everything. It has been difficult for them but thank God for this program, it made everything easy. Now in Ogbomosho, we have 2 facilities where people can go to access their baseline easily without thinking about their transportation or anything. So, I think this one is better than the normal routine DRTB that we've been practicing. |
| Have you encountered any challenges so far in the implementation process? | Yeah, you know some challenges are there because you know it is not everybody that will be so stressed to access the treatment. But some people have religious beliefs and we have cultural beliefs also. |
| Can we talk about some of these beliefs? | Like religious beliefs some people will think like now there are some churches that we know don't take drugs, they believe in praying and everything. If that kind of member of that particular church comes for treatment |
| What's the name of the church? | You said we are not calling names |
| No ooh! It doesn't matter, you’re not calling anyone's name, and the church is an organization, so it's ok. Continue. | There's one particular church that is around our area Orisonwa ministry, so in that church they don't use to take drugs. Now for instance, if that kind of member of that church is TB maybe a DRTB patient he has to be placed on treatment |
| So have you encountered that as a challenge? | Yeah, the one I have encountered as a challenge I can't categorize it as a major belief because that person refused to take a drug he said he would be fine and everything. Even that scenario happened in Ibadan here. There is one particular patient that we used “Mr.Abdulai” the one that sort that intervention, we had to go to the house of that man the DRTB survival, you know I spoke about that earlier on, with the DRTB survivor to talk to the person and say as you can see I'm ok when I take the drugs and all that that once you take this drug you will be fine and everything. But one of the points he makes is how I will be taking drugs with an empty stomach. He is not working and he is not hoping for anybody to provide food and all. That is the reason why he's scared to take the drugs that I cannot be taking on my empty stomach every day. At least I have to eat before I take the drugs. So, we counseled him and then sometimes even the organization supports giving extra money apart from the transportation. In fact, let’s just say the reality; transportation and social support are not enough, N2,000 per month. Sometimes nutritional challenges of the DRTB that have to be looked into are that most of them have a job and then currently the situation of Nigeria as you can see everything is now expensive. |
| You mentioned culture may be some cultural issues, are there any cultural issues you think that put a challenge to this whole thing? | Yes, there are some cultural beliefs, you know there is one particular one that I mentioned. You know like all these Hausas they don't have a location. So if they can get one, there’s one particular patient we detected in Ogbomosho, and then when the layer thing came around he just disappeared. So, most of the time when he gets to the family house where they have sabo (saboteur) in the family house where they live, they will say he has not returned from the most, so it's one of the things that will affect the program and we have some or a lot to follow up. You know all these Hausas don't have a location and then they are not based in the Yoruba residents. |
| Ok can you now compare the decentralized DRTB services to the routine program in your clinic? | In our clinic? |
| Yes | I've explained a lot about the routine and the decentralized one |
| Any other addition to what you've always known? | Ok apart from the baseline |
| Compare the 2 looking at the components of the DRTB Services | Ok I've mentioned the baseline also, and then this issue of GX alert has also helped in early detection and early follow-up of the DRTB. You know you have to wait for results to come out from the lab and everything but with the GX alert, when Mr. Shittu and some of us, me, for example, I'm in the group of GX alert, so when the result is out I get the result immediately and we also follow up, we call the TBLA madam you also know we have DRTB in your facility how is be doing and can we track it so that he can continue on a certain treatment that's possible. So, the GX Alert also helps for early detection and early follow-up of the DRTB patient. So, it helps a lot. And then I've also talked about the baseline investigation. At least it's no more like before. It's now decentralized that in every local government they have its center where they can take the baseline easily without traveling a mile to take their baseline.  Well, I don't know if this intervention of transport support, social support, and treatment support also has helped in the program. You know one of the things is there is also one of the strategies that also helps. You know because they know they will collect money for them to come more for the OPD. We usually schedule their payment for the day they will come for their OPD. So, because of that money a lot of them come, so it also helps. The payment of that stipend is because it always makes them come because we schedule the payment that day. We know that they come for that money but at least when they come, they do OPD the doctor will see them and attend to them and everything will go on fine. So, the program really helped very well. |
| OK, are there other things you would like to talk about like the engagement of the liaison officer?  Ok you are talking about the initiation of the treatment at the local government level? | Yes, yes we talked about it and it has been working very well. They can now take drugs from the facility and all that |
| It's ok.  Can you think of any disadvantages of the decentralized DRTB services? | For me I don't see any disadvantages in it. One of the things I just want them to improve is to know if there's a way they can increase the support of the DRTB stipends. You know everything is now expensive, the N2,000 even let's just say the reality, #2000 as transportation within Ibadan is not enough now talk more of traveling from one end to another I think if they can add more to the stipend for our patients it will be fine. And then one of the things I also want to talk about because we discussed, there is one particular stipend that they are giving for baseline transportation I think that money should not be fixed, it should be calculated by how far the patient is going and not to fix it N3,000 maybe I'm going from here to that junction I will collect N3,000 and someone that is going to far distance from another region entirely will also collect N3,000 I think they should look into that so that they will be paying it based on how far they are coming from and not as a fixed price because it will be an advantage to someone and disadvantage to some people and they look into it. |
| So what changes would you want to make for the decentralized DRTB services to work in your facility? | I don't think there's any changes |
| The changes that you made in your facility to implement the Program. | Ok one of the things I just mentioned, you know I mentioned something about OPD, you know you have to organize it together so that they could have the doctor's services, also they will be ok. It's one of the things that we brought so that because of that money, they will gather more to access their clinic effectively. So it's one of the things that we made that we improved on. Then also there's another thing, you know most of the programs said we should be transferring and that we should not be paying cash and everything. Let's just say the reality, all these people who are accessing their treatment, all these TB-positive cases not everybody has have account number even if we write cheques for some of them have account numbers talk more of bank accounts so in that area we have to be flexible as the CBO, we pay the money to the account of the TBLS so that they can give it to the patients that are ready to access the care. Sometimes, one of the CBO officers will withdraw it in her name and give it to them. It's one of the flexibility for the program to be running smoothly and effectively. Even when a doctor is around we schedule it on that because it was based on that he witnessed one of the OPDs that were paying the patients. So, you see how we are working because they wrote to me that we should not pay cash to the patients again. So, he now came and said to me what about those people that want to start treatment, you know they are not in the program, we want to introduce them to the program and I said for those people that are coming to the program at the baseline we try to be flexible because we know if we are not flexible at that moment those patients may be frustrated and leave. So, in terms of that, we accommodate a little bit of cash, which we pay to their supervisor which is CPLS at least when it gets to Abuja when they see that it's TBLS the person in charge that assigned to them everything should be ok. So there one of the things we are being flexible for the program to run effectively. And then there are others we do tracking of DRTB, we call them for the OPD you know we use to collect their sample for culture, we also call them for reminders and everything because when TBLS have difficulty in reaching them they call us also. But as we go to the TBLS office with our car the TBLS will join us, we do the visiting, others are encouraged, and to the patients, we say ok this is life and death, and if you don't think about yourself just think about your family, the children that are living with you and everybody. I think the program is fantastic, let's just say the reality and then it's not that complicated because you know the normal routine is complicated, there are bound structures that you have to follow but now everything is flexible for everybody to access. So, I think is wonderful. |
| OK, so the implementation is quite an easy one? | Yes |
| I mean the implementation of the decentralized DRTB services? | Yes, it's easy and very flexible |
| You've already said that all the program interventions are the way they are, is there anyone you think should be altered? For instance, when you mentioned that stipends should be reviewed | Yes, I said I think they should look into that |
| Ok apart from that do you think there is any other thing that should be altered? | I think everything is fine. If they want to add any other things they should add but they should not remove anything from this present one |
| Alright, how does decentralized DRTB meet the needs of your patients?  We may have addressed some of this in one way or the other but I just need concreteness of them | I think it meets the needs of TB it's now more flexible for ourselves at least the care is now everywhere you can walk into the facility, take the test if you're positive you get drugs easily |
| So it addresses their medical needs? | Yeah, their medical needs and it's now flexible and the knowledge is there for everybody, the TBLS is there, and the community facility or the staff in the facilities is aware, so the program made it easy for the patients to access all the DRTB intervention easily without any discrimination, without any stigmatization. I think the program has educated all the important facilitators involved in TB and they know how to handle TB patients fine and effectively without any negative discrimination or anything. At least we do training; we do online programming and everything. So the program is well structured and planned |
| Do you think the counseling of patients, especially team counseling with the DRTB survivor, fills any need? | Is it the counseling of the DRTB survivors?  You know according to my understanding one of the things is if they want to embed this thing to the program at this point, I know it's there already but if they want to embed it into the program |
| Does it fill any psychological need? | Yes, it fills the psychological need because you know the TB survivors talk based on experience by saying I experienced this and I overcame this. You know it will give the new patients a kind of relief that ok, at least someone has gone through this and survived. So, if I take my medication very well I think I will survive this too. So, since they embedded it into the program it's fine. |
| It's alright do you think there has been a need to increase or reduce? You've answered that you said there is no need to reduce, but why did you say there is no need to reduce, that if they want to add they should add. You know you mentioned something like that | TB survival, can I give an example? |
| Yes, go on | Ok let's talk about HIV. You know HIV is a continuous treatment so when you are talking about HIV survival, when you are positive you're positive for life. But TB survivors now when you are talking about TB you know some people will be positive when they are positive and then when they are cured they go on with their daily life without even remembering they were once a TB-positive patient. But if they want to embed this into the program you know if someone will be willing to be used as a TB survivor, then that patient will be ventured into the program. But if someone is not willing to be showcased as TB survival it has not to be embedded into the program that's all I can say about it |
| Ok that one you're feeling that is optional | It depends on the willingness of the TB survival if you want it fine |
| Ok but do you see any need for that? | Yes, I've talked about the importance at least you will talk from experience not from what you read. Talking from experience is something you've experienced you talk from it and I will be a kind of motivation to someone new to TB by telling them that you've taken this drug and you are fine |
| What support is available to you for the adoption of decentralized DRTB services in your facility? | The support that's available to us, at least we have support for transportation for the patients, social support for the patient, and then we have support for the house-to-house visitation for the patients. So, these are the supports that are available and then the baseline investigation support is also there. Even though there have been opportunities to be paying the lab, we are the ones paying the lab for the baseline investigation follow up. So, there's a lot of support that's been given to their facility |
| In order not for us to confuse support now and incentives can we approach the differences?  In your office, apart from some of these incentives, are there kinds of support or maybe factors that enable or enhance your ability to implement this not necessarily now? Those incentives as mentioned let me give you an example. Some people have mentioned something like WhatsApp as a form of support, but I don't know what you think about it. And there is an issue with the DRTB request form. Do you think these are the kind of factors that support the facility in adopting the implementation of the service delivery, or maybe there are other things we can embed into the program? Those that are currently in place | I think all the support is there already, we have the WhatsApp group we can communicate with even with the state and everything. And then you know the issue of request form they register and everything will be in charge of TBLS |
| But do you find them supportive for the adoption of the program? | Yes, we find them supportive at least according to what everybody used to say if an activity is done and it's not well documented and if they want the activity to be more recorded and documented, all these registers, request forms, and everything should be available. So, I think it's one of the supports that is supposed to be available, the national is in charge of the documentation and all these papers and everything. We recently have been having difficulty with that. In DSTB we have a lot of support and we have been battling the shortage of referral forms at least since last year till now nothing has been done about it and everything. So, all these supports are supposed to be there for proper documentation of the program. |
| Do you want to talk more on incentives? | Yeah, I think that incentive side of it if they can look into it and try and increase it |
| Yeah, but do you want to talk about the different kinds of incentives that are available for the implementation? | I think I've talked about the available transportation, I talked about the available social support, and then transport for the baseline is available also, especially since transport for the baseline has been effective in the program. Though they didn't sign it at the beginning of the project when you look into it they sign it. It has been very effective. Ok, when you tell some of the patients to come to the facility, they will say I don't have the transportation but when you tell them to come I will give you money, they will come and they will do their baseline so that we can put them on the treatment as early as possible. So, it's very effective for all these incentives that they provide with the program. One of the things I used to talk about is transportation and social support, they should please review it because of the current situation and everything, it’s not enough. |
| So how do you think your facility culture and setting affect the implementation of the decentralized DRTB services in your facility now?  How are your culture and settings, by culture we mean how do you do your things there normally?  Every society, not the culture of the people we are talking about, every facility or organization has a way of doing their things which can be described as their own culture or the setting of that area. How has that been helping your implementation of that program? | I think as an organization there is a culture to follow to make sure our work is effectively done. But on the other hand also you have to work according to the program that you've been given. Their guidelines, their procedures, you need to follow regardless of your culture |
| Our interest now is did your own culture as you know harmful to the program or was helpful to the program? | Our own culture was helpful to the program |
| How? | You know in our organization one of the things that we work on is we make sure immediately we see a TB program. We make sure to bring a case of treatment immediately because the more you delay the treatment, the more harmful it gets. So one of our cultures is early detection, early treatment that means you will place them on treatment. And then if there's any reason or any patient to follow up, everybody, every hand even from our facility to the state will be on deck to make sure that all hospital follow-ups are being brought back to the program. So it's one of the cultures that everybody is working towards, at least my oga here used to call and everything and communicates. I make sure that the objective of the project is being met. I think that's all |
| You don't want to be specific now? | I think I'm specific to some point. You know one of the things as an organization I talked about that is early detection treatment is one of our cultures that has been very useful |
| OK let's look at your motivation. What has been your motivation for the program? | That motivation part you know, one of the things I feel is when I'm working with TB, we also work in HIV we work in orphanage children, it's like saving people. When you save people when you see them smiling you know for someone who was in a sick bed now talking to you will see the joy in them you will feel happy to know that you were able to save this person. The joy is there and everything is there and that is one of the things that motivates us. I think saving lives is one of the things that motivates us and saving a community also. You know when we have uncared TB patients it affects a lot of people and if you think you're not being affected, remember your children are going to school and they take the school bus with other people so in one way or the other you will be affected. So to prevent that you have to work towards eradicating TB in our community so that everybody will be saved |
| OK, how confident are you about being able to implement the program regularly in your facility? | How confident? I think we are more confident about the security program, and TB program, even though there are some areas and some local governments that used to call us Baba Oniko in Yoruba and it's nothing, it's what we do daily so we are not ashamed of it because it's part of our daily life. There's no blessed day we will not come in contact with a TB patient because we call them, we have meetings with them, we go to the facility where we see new cases and we follow them up. So they are like our family and they are like brothers so there's nothing shameful about it. We are confident in working with them and the program has been wonderful. To be sincere with the program we too are fine at least it's our daily activity and we are happy to do it also. |
| So what you are saying is that you are also confident that your colleagues will be able to implement the program regularly in your facility? | Yes, my colleagues have been wonderful even my CBO, the colleague, the M&E, Clark, the CTW, everybody has been working towards TB so they are confident in working with TB. You know if you're not confident because some people feel stigmatized, and if you are feeling that way you can't work in TB premises or TB environments, you can't work where there is TB because you make contact with them, and everything. And then we are talking about people, we are not talking about animals, there is some emotional support and everything is there. We have to be confident with them so that they will know that we are with them psychologically, and emotionally, and then we are wishing for them to be ok. You know if the person that wants to console a sick person is not that confident it spoils everything, so we are confident in what we are doing, we are very-very confident and everybody is confident with it |
| Let's talk about the fit of your service delivery with your existing work and services. What I mean is the program does it fit well or align well with the processes in your office or services? | Yes, the program aligns well |
| How? | In terms of testing service delivery, you know the organization has been working even before TB. We've been working on HIV for over 15 years and I've worked with the organization for 13 years now, especially in the HIV program and everything, and then when the TB program also came, we did it together. The organization has been aligning with the program for a very long time. Everything in the organization works in terms of treating or in terms of testing or in terms of screening and everything |
| Is the work burdensome or rather cumbersome to you? | Yeah, let's just say the work is cumbersome you know one of the things is documentation, tracking, and everything. When the work is cumbersome one of the solutions is to recruit more so that the work will be easy for everyone and all the personnel so that the work will be easier for everyone. Although everybody knows that the TB work is cumbersome. |
| I'm talking about the decentralized TB service delivery maybe you find it time-consuming or quite challenging or doesn't really fit well into what you're doing, not distinctive, but if it really does it's fine | Is it about TB generally? |
| No, this program | Well it's flexible. You know as an organization it's not a one-man program so everybody works together every day. It makes ms the work more flexible, so it's not that cumbersome. |
| So I want to now draw a conclusion on whether you think the decentralized DRTB services should be replaced or complemented with the routine one. | I've said in the beginning of this interview that the decentralized program is far better than the centralized one. In fact the decentralized should be the main one |
| So should it replace the routine one? | Yes, it should replace the routine one, at least with the advantage I've listed before the baseline and everything. So, I think the decentralized one is better |
| So how do you think that this program can be scaled up to have more centers in other places that have not gotten? | What I think for this program to be scaled especially on DRTB baseline investigation now it has gotten to a point that the money we pay for the TB testing at the lab, got to a point that the facility is no longer collecting that money, so we will have to shift from one lab to another looking for the lab that can accommodate the price we have. So, the program should look into the price so that when they scale it up the screening will be more flexible for people to attain and that's one of the recommendations that I think the program should look into; the price in the lab. They should keep up with the standard situation of Nigeria and keep up with the standard price. You know when the lab people are 2orking they will be working in a very rude mind and not a good one, so they should look into that. One of the things I said also concerns the stipends of TB patients they should look into it. |
| How do you think that this program should have been sustained? | I think this program can be sustained by taking it to the state to be a safe of it, I think they are aware of it already but we have to get them to be more involved in the program you know we should not depend on the foreigners to contribute everything so that the state can be contributing, even if we didn't own everything but when the state contributes small it will reflect in the program at least with that gradually by gradually the program can be sustained by the state themselves. |
| So, are you done? If you still have anything to say then just continue but if you're done then we are done. | I don't have any other thing to say |
| Okay, thank you so much | You are welcome; thank you too. |

1

KII/A2

The focus of this interview is going to be on the TB reach wave 9 interventions and decentralizing DRTB services and just as an eye breaker sir, we want to know what you think of the interventions, I mean the TB reach wave 9 interventions

*Res*; Thank you very much well about TB the interventions is very good and this will help us at least to reduce the incidence of TB in our domain and not only that the intervention is very good so far we have been able to reach I mean to diagnose many of the patients and some of them are now getting better than before and for the look of things many of the diagnosis that we have doing we have been seeing improvements so there’s need now to do the decentralization and I hope by the end of the whole program. We are going to record a better success rate.

*IT*; Now that the implementation is ongoing, I want to know what you like most about this decentralized TB services.

Response; the decentralization as I told you before is good because it would help to reach all the other cases and not only that it reduces burden, you see when you want to reduce the burden it is better you detect as early as possible by doing early detection it means that the burden is going to be reduced and the patient is going to have effective treatment, that is why it is good. They don’t want to spread it again.

*IT*; is there anything you like least about the intervention?

*Res*; well, what I really appreciate most is the incorporation of the other private sectors the incorporation of other private sectors laboratories because Government cannot make it alone, they cannot do it alone and when you are doing diagnosis you will be able to know the response of the patients in term of treatment so is very good.

*IT*; what has influenced your practice of decentralized services?

Res; well, you see Sometimes when is only Government issue when it comes to strike and other things and shortage of manpower so the issue of decentralization is very important when you incorporate private sector there is nothing like strike likewise you think of manpower because the syndrome of japa will not japa tomorrow, is not much rampart in private sector is only that it involves a lot of capital in private set of Lab there is no any much support in all these infectious diseases.

*IT*; you know that right now sir because of this intervention, we’ve been able to devolve GX alert to now include local government TB supervisors as well as the patients, and there is been improvement even in the tracking of patients so and also in the pretreatment counseling there are some other areas there are other ones I want you to look at for instance are there challenges you have encountered with regard to I just mentioned these.

Res; you know some of the challenges that I can just say is the attitude of the patients, you see is just like the other time when a patient has been on treatment and is under monitoring as well when you tell the patient this is the he is supposed to go for another investigation maybe the might not be around and he will not know that the side of diagnosis is monthly maybe first week every month you have to go for this thing apart from taking the drugs I think you understand because whether you just need to look at how to track the patient and call the patient so look you for this diagnosis this month

*IT*; ok how have been able to cope with these or rather are there things that have helped you in the implementation?

RES; I have to liaise with the TBL supervisor. Sometimes the supervisor will help to tracks the patient in the local government likewise I do call the patients just inform the patient that look don’t forget first week next month you will be having your investigation come for further investigation

IT; thank you very much sir, can you compare these decentralized services with the existing routine ones in your clinic

RES; Decentralization is very good likewise the routine aspect of it but the decentralization we are able to move closer to patient, in routine services when a patient is able to produce sputum or all other things the patient may not even feel sometimes he will think he has submitted everything and everything is left to them then he will go and meet the supervisor but in the decentralization sometimes you will see the patient even right from the onset by the time you conduct 2^nd^ or the 3^rd^ aspect of the test you will see that the patient is improving and that one will serve as a better this thing morale for you that you making things working.

IT; in a nutshell what are the advantages of decentralized over the routine?

RES; you see in decentralization there is better human services there compared with routine services and in the decentralization, we also have a means of checking the patients though we are not the ones giving out the real services in term of treatment but in term of diagnosis we are able to see the patient and know that okay if sometimes if the patient needs additional this thing, we just refer to the supervisor that this patient you have to look much into the patient.

IT; do you think this decentralization of services has some disadvantages

RES; well, there is in life that doesn’t have any disadvantage is only if you want to learn in this decentralization, you the facility that is being referred to they too they have their own work but in order to achieve a better goal common goal we have to work amicably better communication so that there is no any communication gap between the supervisor, the patient as well as the facility, if there is no much communication it means we will be losing a lot

IT; so, in comparison with routine do you think is advantage to decentralize services

Res; yes, in routine you don’t have much communication you know you accept that this is coming you have to work it may come at the right time or it may not come at the right time but in the in the decentralization when there is better communication you get prepared for the services you want to render for the patients

IT; alright I would want to know sir as the director what were the changes you made in your facility in order to implement these decentralized services

RES; well in my own area what I did is that before I don’t have safety cabinet but because I know that am dealing with MDR I purchased a better safety cabinet, biosafety cabinet likewise some of my staff those staff dealing directly I have to mentored them give a kind of this thing I mentored them so that they know how to handle all these samples because I don’t want any of the staff to get infected likewise the environment I tried to curtail all disposal most of the contamination and disinfection

IT; how complicated do you think these decentralized services are, I mean the decentralized DRTB services how complicated or how complex?

RES; is not complicated at all I don’t see it as complex I don’t have any problem with it in term of complicity compared with the routine services, is simple is only that you have to do better documentation

IT; does it increase you burden

Res; it doesn’t increase my burden only I just have to have personnel on ground, the only area that I do have is the cost of the commodity the prices are changing in the market.

IT; ok now this decentralized DRTB services how does it meets the needs of your patients?

RES; the moment I know that am having patient with DRTB, I don’t discriminate what I do is just the way of handling the patient

IT; do you think the services meet their demand, their health needs

Res; yes is meeting their needs you know before DRTB patients at initial stage before starting other drug regimen when you see the patients some of them in the facility will be trying to dodge him but the moment the patient start the improving you will rarely know whether is the patient or not any more you rarely identify as DRTB patient because is going to live a normal life and by the time the other cx-ray, ECG and other things you is getting normal and other LFT E&U all those investigation you will see there is improvement ,yes is meeting their needs without any controversy.

IT; so, you it meeting their needs

RESP; Yes, it is meeting their needs without any controversy

IT; has there been a strong need the services in your facility

RES; Yes, there is need, the MDR patients are very small as compared to other areas and by the time we do much diagnosis them, one will be able to can pick them and when you are able to pick them track them then you will be to know the next step so that they don’t spread it anymore those people that have been cured they have been cured and agreed the will not come back and their family they do come and appreciate because initially when they come they normally been supported by the family and the time the patient come by himself you see that changes has come.

IT; in essence you have no any need to reduce DRTB services rather there is to increase the services from what you have just said.

RESP; yes

IT; what support is available to help you adopt these services in your facility

RES; you see I only talk about my own personal support in term of this thing but support I need from other partners any other things that can improve the diagnosis services I would appreciate just like microscope now I have my own personal microscope for diagnosis.

IT; apart from that are there any other support from external bodies

RESP; Damien Foundation and the state government all these things they are the ones supporting sometimes they in terms of logistics and finance. Through the TB, we have reagent and other things, they provide reagents for TB not for the MDR. For the MDR what we normally do is the LFT, those baseline investigations, I think you understand?

IT; yes, but how does the legend now support your own work in decentralized service?

Resp; you see that, most of these reagents even the glove and other thing they don’t have it for our own head, it is those people that bring it, I think you understand? Even for the normal AMB that we do, it is Damien that bring it, I do collect from there, I think you understand?

IT; yes

RESP; and the baseline for all these diagnoses everything has to start from those area even if you want to detect MDR, expert and other things I think they are still under diagnosis, they also assist in detecting the MDR, I think you understand?

IT; yes

RESP; but for the treatment days, and further diagnosis that’s why we now come in to do our best.

IT; so, what kinds of incentives are there to ensure the

RESP; have told you in term of reagent support, all the other things, the investigation we do even though we are managing our self in this economy crisis, you know to buy fuel you know how per liter how it is and we have to run the lab 24hours so if they still have other equipment that they can use to support us, if there is equipment now apart from other equipment that I have then i will appreciate it.

IT; okay, are you saying there are not incentives apart from those support system we’ve talked about?

RESP; the incentives I received is just this only

IT; but how do you think that your facilities setting affect the implementation of this decentralized service?

IT; you see my facilities is at the central of the and the proximity is easier to anywhere you talk that you want to go to practice this thing then you want to go to the diagnosis center, they will easily tell you that this is the place

IT; or the culture, I mean the way you do things in your facility, the way you have organize things in your facility, are there ways that they helped to improve the implementation of this decentralized services ? or ways they or either way, can you explain sir?

RESP; in term of organizational structure and the culture the way I do things and you know it’s not congested as well so there is free movement everywhere so it’s easier for us to do all these things, so there is no its not jam in term of complexity then the way we disposed our waste management is even good so everything is not littered

IT; okay it has helped improved implementation

RESP; yes

IT; okay but can you tell me or tell us about your motivation for one thing to help ensure that the implementation is successful

RESP; well, the little support I receive I appreciate it, I appreciate the donor, I appreciate everybody. Well as it is now, if the other support I can get from them I will still appreciate it just to assist me in doing further investigation diagnosis of this MDR patient.

IT; Okay. Now are you confident that you’re able to implement these services regularly in your state?

RESP; yes, you see all what i have put in place in the facilities has really helped me a lot and I told you that if there is no better communication, I will not achieve so base on all those things I’m able to achieve a better thing.

IT; how about your place those you work with, how confident are you that they are able to implement these services in your facility?

RESP; you see I told you that, I told you I do stead hand training likewise apart from doing stead hand training I do a kind of mentoring because I do participate, do it by myself. By doing it I try to motivate them as well and all the thing that they may even run away from, i have already put all the safety things in place

IT; so, are you saying you are confident or that you’re not?

RESP; I’m confident that I have done a lot

IT; then I’m talking about your place

RESP; yes, they will be because I’ve been doing this for quite a long time and I’m not infected so I’m following the safety procedures so they too I think they have all these things.

IT; I know that as we’ve discussed, we might have mention this but I just want you to encapsulate that how the service delivery I mean decentralized DRTB service delivery fit into your existing work processes and practice, I mean your work processes in your facilities, the practice of your work, how this delivery I mean how this intervention fit into it.

RESP; I don’t have much problem with the intervention fitting into my system.

IT; how it fit into your system.

RESP; you see, it’s because I’ve already provided a better avenue for service improvement. Based on this any infectious diseases coming in, in term of maybe doing diagnosis, I have already made much improvement in term of equipment and the safety, facility safety there so that I don’t spread infection. So that’s why something like this, MDR this thing is able to fit in. it’s not only MDR that I’m dealing with so any infection disease can come in any time so I will be able to do better diagnosis so that I don’t spread infection.

IT; okay but there are three questions I will ask you; one is; now are their components of this intervention that you want to altered? If you can remember the components including the fact that GX alert is now send directly to the local government TB supervisors as well as the then the efforts that have been made, the financial incentives, the use of USSD among others as well as the use of autopsies in investigating or verifying claims of deaths. So, all those components are their ones you think can be altered? Or do you think they should be left as they are?

RESP; you talked about the GX alert

IT; yes

RESP; all those components that has made it now easy for the gap between treatment diagnosis and enrolment, presently I don’t think there’s any problem in those areas, it’s only the incentives that I can really talk about there that it has to be improve from that. That’s the only area I can talk about.

IT; how do you want them to improve on that?

RESP; you know, the standard of living now and the means of communication everything has gone up so that’s why they just have to, likewise in those other areas, I talked about equipment as well so.

IT; alright. You’re telling about how they can improve on incentives for

RESP; I told you that following the reality of things now, the incentives is too small. Well, it depends, they can just revisit it, call for meeting and see how the thing can be review

IT; do you think that this decentralized service should replace the routine one or compliment it?

RESP; It has to compliment it, it cannot replace the routine program because in decentralization you cannot have much vaccination like the routine aspect, I think you understand? Because when you visit each community, they have their local problems so you can only look for some of the facilities that can complement the effort of the routine services so that we can move forward.

IT; alright, so your conclusion is?

RESP; my own conclusion, well it’s just to look for better way of services so that we are going to have maximum resort.

IT; and you believe that the decentralized service should complement the routine service

RESP; yes. Decentralization should complement the routine services

IT; thank you very much sir, I appreciate. Thank you very much

**KII/A3**

| **Interviewer/facilitator/questions** | **Respondent/responses** |
| --- | --- |
| Alright, as I explained before that the intervention will be focusing on the effectiveness of the interventions for decentralized DRTB services and then, also looking at the factors that influenced the implementation of the interventions in your state.  So, My first question ma is, what do you think of TB Reach Wave 9 interventions to decentralize DRTB services? | What I think is that the organization has provide new form and that form is full more than the one we were using before; so it contains the next of kin of patients and that was not in the former one we were using before. |
| Okay, so in that case there has been an improvement? | Yes, with the interventions. |
| Okay, do you think that the interventions are good? | It is very-very good. |
| Can you tell me what you like most about the interventions? | What I like most is that it has been making our patients very happy to come receive their drugs because of some supports that this organization use to give to the patients when they go for this monthly check-up that they use to go; so they give them some money. |
| So, is there something that you don’t like much or let’s say what you like the least; for instance if you talk about all those things they do; those components; the transport support that is given to them; the one that is given to CBOs; the messaging or the alert that is send to them; the fact that the baseline investigation is now at the preferable centres and all other ones including the engagement of liaison officers; which one do think you like the least? | It is the money that they use to give to the patients because before; they have been complaining that they don’t have money for transportation; so that money helps them alot. |
| It is okay; so, what influenced your practice of this service delivery? | It is the love I have for the patients because they are our people; we live together; we do everything together; they are our neighbours so, seeing them well is our pride. |
| So, seeing them get well as they take their drugs? | Yes. |
| So, that has been motivating you to do more? | Yes sir. |
| Okay, have you had any as the result of implementation of the program? | “long pause” |
| Any challenge that you have encountered; have you encountered any challenge or you have not encountered any? | There is no any challenge. |
| That you can think of now? | Yes sir. |
| Okay; let’s look at; let’s compare the decentralized DRTB services with the routine program; the routine existing program in your clinic; what I mean by this is the former way of doing things and the decentralized ones; that one that the patients have to go to the state capital; so, I want you to compare it to this one we are doing now, where they have to access these services at their community level; so, can you compare which one is better and why.  You might have said something about it when you started but I want you to address it more as a question? | This one that we are doing now is very good than the one we were doing before because now the treatment is very –very close to them; it is not like they go through a lot or too far to access their treatment unlike before when everything was only at the state capital. Now it is just two weeks they begin to receive their treatment. So, I think what we are doing now is very-very good. |
| So, delay before enrolment has been reduced? | Yes, it has been reduced. |
| Are there other advantages you can talk about? | Another one is about giving the TBLS alert; that is if they run test inside lab and dictate any RIF resistant, the only problem there is that they won’t mentioned the facility that patent is coming from but they will indicate to all the TBLS that a patient is with RIF resistant from so-so geneXpert number but the only thing is that they will not include the name of the patient and the centre name where the person comes from. |
| Okay; okay, now are there any disadvantages of this decentralized DRTB services? | Haah!...there is no disadvantage oh; it is all about advantage to the patients and we that are giving them the treatment. |
| What is the advantage to you? | You know patients come when they are supposed to come.  They do their tests when they are supposed to do it and nearer to them not that they will go far like before. |
| Okay; so, it helps you to take care of your patients quickly? | Yes, very quickly. |
| What kind of changes did you make or did you need to make for decentralized DRTB services to work effectively in your facility? | “Long pause” |
| I mean during the introduction of decentralized DRTB services, were they certain things maybe some adjustments you people made at your places of work or offices just to ensure you help to implement the program well? | Well, we have to set some space apart for the patients; space like OPD for them to be sitting down and we give them health talks for them to know the importance of taking their drugs everyday; and we let them know that if you fail to take your drugs; that those things that will happen to you when you fail to take your drugs; so, we set a space apart for them for all the health talks.  So, any new patient that comes; we provide him or her with nose cover and tell him or her how to be using it; that the usage is not in this facility alone oh, even in your house you have to be using it when you want to cough. So, we give the patients nose covers. |
| Okay; are you saying that before the introduction of the decentralized DRTB services, you were not making use of nose mask? | No, we were making use of it before but now; you know when one person is doing something it may not be enough but when two people join together to do something it will be better; this because the intervention has made the nose mask to be available always. |
| So, you now have the nose masks available? | Yes sir. |
| Now these components that I have mentioned earlier that have been mapped out; are there ones you feel that should be altered or changed or should be left as they are?  Are they good as they are? | “Emmmmmmmmh”.....all those things are good but the only thing the organization has to do is to be giving “we Dots something” to help us too as they have been giving to our bosses “the TBLAs” |
| To be giving you something like what? | “Long pause” |
| The materials to use for work? | “Laugh”....the materials are already given to us; but maybe like “some changes (little amount of money) that they use to give our TBLAs; may be small money; “laugh” you want me to mention that. |
| Thank you.  How complicated is this program of service delivery or is so difficult to implement? | No!!!  What I know is that it even make it easy for us. |
| How? | You know when patients want to run tests; you know they used to run their tests every month; they now do it at nearby laboratory that is not far unlike before the interventions.  They give patients some changes (money) for transport; there is no longer complaints of saying I don’t have money, that is why did not come to collect their drugs. |
| And that makes the work easy? | Yes sir. |
| Okay; let’s look at how it meets the needs of your patents.  So, can you tell me how the decentralized the decentralized DRTB services have meet the needs of your patients? | I have said it earlier, no some people or some of them use to say; how much are they given us but some of them appreciate it because a times if they collect that money; the organization call it transport money; they still use it for other things like food that they use to buy; so it helps. |
| What other needs that the people that have this RIF resistant in terms services?  Do you think that it has bring the services to their doo steps or bringing about the cure of the problem that is their medical needs; can you tell me about that? | Because patients come to the nearest facility to collect their drugs; so will not fail it; they not fail using of the drugs; there will no way we will not follow up with them; so, they use to come every time.  So, it has been helping them to use their drugs everyday because the facilities are now near to them. |
| Alright; let’s look at whether there is a strong needs to increase or reduce the decentralized DRTB services in your clinic or in your facility? | There is nothing to reduce. |
| Is there need for increase? | The only increase you should be talking about is to have the name of the facility when the REF resistant that is dictated in the lab.; the name of the facility and the local government that has the facility; they should be having to the GX alert that they use to give our boss. |
| But I am now asking if the program is good; do we now need to increase the services so that the needs of our patients can be better met | Yes. |
| Can you talk on that; you should explain if you said yes? | That is what I just said that the alert that GX alert should includes the name of the patients, the facility name and the local government of the facility. |
| That one is noted. | Okay. |
| I am now saying that should be done to include the name of the facility and patients; Am I right? | Yes sir. |
| In addition to that; if you think that it should be increased; you said it should not be reduced?  That the activities or services should be increased? | Yes sir. |
| Why did you said so?  Why do you think it is necessary for these services to be increased? | “Long pause” |
| Is it to meet their needs or to serve them better? | Yes, that is what I had wanted to say; it meets the patients’ needs and serve them better; yes, the program should continue. |
| It is alright.  Now, what supports are available for you to adopt this approach of decentralized DRTB service delivery in your facility? | “Long pause” |
| We are talking support system now; is there anything that supports you or any enabling factors or anything that is helping you to be able to adopt those things not things that are making it difficult for you to adopt those things? | Because of the love I have for the patients. |
| The love you have for them supports you; are there any other supports you can think of like are in any WhatsApp group? | Yes sir. |
| What other things do you think that they are your supports in the adoption of the strategy? | Because they made everything we wanted to do for the patients easy for us. Their drugs come all the time hence we have not had shortage of drugs. They patients come for their drugs always because of the little money they are being provided with for transport. |
| It is okay; let’s look at the incentives, you have been mentioning them but can you speak about them more?  The incentives; what kind of incentives are there to ensure that the implementations are successful? | They include; transport support to the patients.  Communication supports to the TBLAs and to the patients. Communication supports to the survivors of TB and to the CBOs too. |
| Now, let’s talk about how your facility hall or setting affects the implementation of the services?  The way you do things in your facility or the setting of your facility; does it in anyway improve or enhance the implementation or does it affects the implementation of the services? | We have a place set aside in our OPD; that is where our patients sit, the we go there communicate with them; give them health talk and advice them to be taking their drugs; tell them what to eat and what not to eat. |
| What I am saying is your culture the way you do your things; do you think it helps you or hinder or frustrate your efforts? | No!  It helped us. |
| Now we want to talk about this; though we have talked about this but now discuss your motivations; what has motivated you to want to see that the implementation of DRTB services are successful? | Is the love that I have for the people. |
| What of the love?  The love you have for the patients like seeing them get better? | Yes. |
| Tell me about it? | You know these patients are our people? |
| Yes. | So, when see them looking healthy is always our happiness and this motivate to want this program to continue and be a success. |
| How confident are you about been able to implement the program regularly by your facility or are you confident that you will be perform the services regularly? | I am confident since we have been doing it before and we have been treating or dealing with DRTB before; so, I am okay with it. |
| What about your colleagues; are you confident that they will be able to implement the interventions regularly? | Yes. |
| Why do you say so? | It is because if I am not around they too can give patients their drugs and other services. |
| I want to ask you whether the service delivery (the decentralized service delivery) does fits with your existing work processes.  Does it fits well or does it distorts or increase the burden of your work unnecessarily like time consuming or does it integrate well with what you already have in place? | It integrates well; although you cannot compare taking DR drugs with DS drugs but we are still okay with it. |
| Finally, I want to know what your opinion is; whether the decentralized DRTB services should replaced the current program which is the centralized one where patients use come to one centre for their TB services; should they complement each other or left side by side with each other? | “Long pause” |
| What do you think? | They can go together. |
| Why do you say so? | They don’t disturb each other because they are in the same line. |
| So you think they should go together? | They should go together and continue. |
| Alright; thank you very much ma for your time and all your patient and responses. | Thank you sir.  You are welcome. |

**KII/A4**

| **Interviewer/facilitator/questions** | **Respondent/responses** |
| --- | --- |
| The interview will be focused on the effect of the interventions for decentralized DRTB services and then the factors that influenced the implementation of these interventions and sir we will start by asking you; what you think of TB Reach Wave 9 Intervention to decentralize DRTB services in your State? | Thank you very much. Once again, XX. I’m the chief medical officer of health and director primary health XX. I work as one of the OPD TB clinician at XX. Now in the first place let me appreciate the organization for having to introduction this package into our health system for their choice of Oyo state as one of their site to implement the TB Reach Wave 9 project in Nigeria. The wave 9 project has brought a lot of improvements to TB care particularly in Oyo State. The old system we use to operate the TB Reach Wave 9 project has changed that old regimented routine programme. Now it is made easier particularly for the clients because the decentralization of most of those activitieshas made things to be seamless has made all things to be seamless particularly for the clients and even to us even as TB implementers.    To start with let me say that for us the turnaround time for us to get result of Jin expert is quickened and everybody is brought to that same level where we can access that information across the state. There is a common platform that is created for all the all the TB personnel, all the TB care personnel in the state whether at primary health care level as TBLS as the State level as TBLS in fact even to Tertiary level there is a common platform where we all relate to an information is commonly shared at that level so what is going on at every minute we can all key in into it and relate to information within a very short time so that opportunity for us you know to get information is very very-very critical, is very critical in TB care. The other thing is for Laboratory services you know okay so the other thing is well a lot of supports you know is been offered to the clients to the client themselves so much so much |
| Alright, alright sir. Thank you very much although we will have time to talk more about the advantages of the intervention. However, you mentioned about a platform what platform where you referencing to? | That platform is DRTB WhatsApp platform, yes so with it you know the result of gin expert we can get that result in real time, real time information we get that and that is very-very important very unlike before you know when a client process, when is sample is processed and result is available it will take some time before one can get that result for that I’m in fact is one of the most important achievement even to me. Very-very important to me. You know you don’t delay in whether before treatment before we commence treatment of the patients’ pre-treatment care. You know you get information about your patients and the patient can even get information about his/her own result in real time I mean real time result that is very-very important. That is very-very important. |
| Alright sir. So sir what you said now are you inferring that or insinuating that the most important aspect of the interventions that you like or the aspect of the intervention that you like most is the sharing of information on that platform? | Yes |
| Okay if that is the case, may I know what you like the least about the intervention? | That cannot really come to my mind for now. you know for everything about the whole programme I mean I can really for now you know bring out thing as regard weakness or shortcoming but, in fact you can begin to talk and talk the positive impact of that result you will not understand. When you look at you know consider it from the perspective of the patients it is, the benefit is huge and you look at it at from the perspective of those of us who care for the client I mean it is also hug and if a patient is defaulting or we are losing let’s say a lost to follow up or either to pre-treatment follow up or to treatment follow up, we track them the information you know can commonly share in that platform and we see. Is very-very good. Is a good thing to happen |
| Is alright, may we know what influenced your practice of decentralized DRTB in your state? | You know the complaints that we the service providers have been rising before the intervention of the organization. That turnaround time of one result of information sharing because you see in the old system it was the hard copy okay, the gin expert result in hard copy form is been used to share information and sometimes text messages but now it is made enough easier seamless because it through digital now every has been digitalized and that is very important. |
| So what has helped you the most in the implementation of this DRTB? | What do what? |
| Has helped you the most? | Well, what has helped me most is being able to the GeneXpert result, that is number one. Two, for information you know to be for instance when result from laboratory is conducted or is out or result from laboratory |
| Especially the RIF resistance | Yes, so when we get result from the laboratory either from the tertiary usage or from the laboratory that is been used you know for us to of cause you know to relate to the progress of care of our clients you know we can get those result and real time do you understand digitally without having to delay do you understand so and this you know has really made the whole exercise to be so; to be not better than what it used to be |
| Is alright, so sir has you encountered challenges in the cause of the implementation of this service delivery? | Yes sometimes you know the, one of the challenge that I have seen is when clients will presents do you understand and for them to go back they seems to be a little delay I mean a challenge in supporting I mean in the area of transporting themselves you know back, okay I have seen that then some that has multiple body conditions like you have some clients you know apart from TB presenting you know with diabetes, presenting with an accident like a man that had fracture you understand that in fact it took sometimes for the man could recover.When you are managing a DRTB patients and another complication set in different from the TB you are managing is not going to make things you know easy for you as inclination. For some of those few cases there really cost on me with problem you know but thanks be to God we were able to at least try manage some of them |
| But how is that a challenge in the situation of decentralized DRTB? | It is a challenge one when the clients couldn’t transport themselves, okay I mean let say to the clinic. Too; when a patient that has fracture for instance couldn’t support himself, I mean to the clinic do you understand so that can lead to lost in follow up and treatment do you understand that can happen yes. |
| Yes, although the program seems to have reduced lost to follow up | Yes, of cause drastically because before a lot, a lot |
| Alright we will come to that sir but... | And that is one good thing that the TB wave 9 you know TB wave 9 has actually achieved, it has tremendously brought low that challenge of lost to follow up and treatment |
| Yes Sir, you have mentioned the issue of transport of the clients at least twice in this interview but I am aware that they receive some support for transport especially for baseline investigations. | I thank you for that it is very true,there is no deny that fact that that support wasn’t there but the timeliness of the release of that fund,the bottleneck that is been created around it do you understand for the clients you know to get the fund for instance they insisted that they must open an account do you understand and the people we are dealing with here you know TB and poverty they are linked, is linked so sometimes you know to some of them they don’t have a direct account you know any personal account of theirs may be the kind of account they have is for their relations and when you, the community stakeholders you know that is anchoring the payment process do you understand is since they cannot pay to any other account apart from that of the clients so such bottleneck you know creates some little hitch do you understand for them to get the fund do you understand so that timelines is every big battle you know this people they are own employed, they are own employed look at inflation in the country today, the level of poverty is increasing and when you have token to give to people you know to support their transport that fund is not coming as at when due then that can create a kind of discouragement or set back do you understand, is not to say that you know the fund is not been provided you understand |
| But the bottleneck around it | Yes. You see, you know out of my own personal commitment as a director I had to release N25,000 every month to support the clients you know I mean in the area of transport you can see if I had not you know observed that aspect of the challenge that wouldn’t have come in the first place |
| Is alright sir, is alright sir | So, I just want them you know to make it you know seamless you know for them to get the fund and that timeliness of the release of the fund, we did discuss this even in one of our zoom meeting I talked about it |
| Is alright thank you very much sir. Woo! Although you have mentioned or made reference to the difference between the decentralized DRTB services and the routine existing programme in your clinic, I wound want you sir to address this issue as in compare the two the decentralized DRTB services and the old way of doing | Thank you very much you want to dig into my memory nowyes for me to bring out the goods about what the programme the TB Reach wave 9 has brought into our State. One in terms of the advantage I did told you earlier that the real time release of result okay is a lot better, is made easier okay than the previous routine process where is only the State TB programme manager or officer that will get that result then release that result you know piece meal to TBLS okay, now everything has been centralized digitally and as soon as that result you know is out from the laboratory everybody relates to that result without any delay okay so that has really brought a lot of improvement you know to the whole exercise. Two secondly the decentralization has helped in caring in treating even the patients even our clients for instance we have OPD site do you understand where testing can be done, let say even within Ibadan okay at Jeriko, at |
| Okay decentralization of | Yes, of laboratory Services |
| Services | You know before now, until now or before now, result can only be processed at Ibadan and sent to Saki and sent to Oyo to Ogbomosho do you understand all those have been taken care of now can you see, all that have been taken care of now so we know have a TB Liaison an engagement officer who now help in facilitating all these so well for laboratory processing I mean that can be readily be done in Oyo it is done I think in Ogbomosho in Saki in Ibadan here I think about two or three places here in Ibadan so that has really made it you know so easy, so seamless you know for people around that zone, do you understand to access care so patients wouldn’t have to come to Ibadan let me say this before now infact the cause clients the inconvenience of having to transport themselves even to Ibadan for them to get some of this stipend so all that have been taken care of now. The TBLS you know in all those zones or the axis they have to come to Ibadan all that have been taken care off now. can you see so getting result, processing result everything has been made easier for all us in the state |
| So compared to the old routine programme, what can you now say, are you saying that this decentralized DRTB better | It is perfect now. It is perfect that is what I’m just trying to say. In fact, we can’t really even emphasise the benefit of the TB Reach this decentralized system. We can’t really say it all, is really much. Is something we really wish to continue to see. Oyo State is too big for things to be centralized at a single point. When you decentralized you get things you know the easier you know you make things to work better, you bring about convenience you know in terms of turnaround time of result you know that even patients you know you wouldn’t lose even your patients you know patients will find it even easier to relate to whatever you are bringing to the table to them. |
| Is alright sir. Are there disadvantages you can think of although initially | As relate to decentralization? |
| Yes, yes. | God will help me to think out that one I can’t think of any now. really is a good thing |
| Were there changes you needed to make for the implementation of this decentralized DRTB services to work effectively in your facility? | Yes, as suggestion now abi? |
| No I mean the ones you had to make; were there things you had to put in place, were there things you have to change in other to accommodate decentralization of DRTB services? | You know at a time we had a privilege of getting N250,000 during the world TB day, okay you know what I did, I printed case note for you know this kind of case note I printed for our OPD site, I got wineskin because the wineskin we had then is faulty so we got a new wineskin, we printed you know the description jotter, we bought a fan okay and I know my TBLS also did something, I think a shelve or so for her own clinic there so that we did just to support you know the because at the OPD site there we support other points like clients from North-East, clients from Ibadan North, Adeoyo, Maternity Teaching Hospital Adeyini local government and Ibadan North West you know we are the ones you know that receive their clients so we had to make things you know more comfortable and convenience for both the clients and the even TBLS when they come during the GOPD clinic so the environment were made suitable in terms of benches and other things. So were alreadymade in. and like I told earlier even for the clients because we don’t want any of them to be lost to treatment follow up even to treatment, do you understand we also support what they also do. So, we also support what they do. Those are the kind of things we had put in place for now to make the whole programme to be smooth |
| Now looking at the component of this interventions are there ones you think should be altered or should be changed as the components you know while we were discussing you mentioned setting components yes including GeneXpert or GS Alert to Local Government TB Supervisors as well as the patients and you know they are quite a good number of them I just mentioned one then the other one has to do with giving output based financial support to the CBOs by way of or in or attempt to improve the tracking of patients. We also have the structured pre-treatment counselling, team counselling which involved the TB the DRTB survivors, also some stipend given to patients for baseline investigations as well as use of USSD to improve also the turnaround time of baseline investigations so I think there are other ones including the decentralizing the treatment initiation at the local government area level as well as use of verbal autopsy to verify unwanted claims of pre-treatment test and lastly the engagement of the state liaison officer volunteer. So, these are so these components are there ones you think should be changed or should be altered or they should remain as there are | Thank you very much. I wouldn’t give you a yes or no response is better to speak to a few of them like the GeneXpert alert is one of the main thing to happen you know made the remark earlier that for us to get timely information do you understand on clients result is very critical to treatment do you understand, somebody who has DRTB even les say DST even drug treatable you cannot delay treatment, you cannot because you see when you delay treatment the complication you know the conditions become more and more complicated do you understand this is the issue of a lung disease do you understand which will affects the heart which will affect you know the entire organ of body system now a drug resistance, you can’t delay so is one of the best thing you know to happen do you understand that GeneXpert information do you understand that technology of having to share information is one of the best thing so we cannot even thinker with that. Okay for CBOs, for the CBOs what I want them to do is to be more committed particularly in the area of home visit, home visit to the patients. You know some of you know the clients they doesn’t have, they don’t want to give their home address because of stigmatization do you understand but there should be a way round it do you understand of where the clients some of them you know were mate at motor park they don’t really have any fix address so but a way of working round it you know to make sure that the fix address of the clients is fully or genuinely determine so that we would have this problem of lost to pre-treatment or lost to follow up or anything do you understand because it can be liken to a day break when a criminal escape from the prison and you know what that constitute the danger that constitute you know to the larger society that is how I can liken lost to follow up or treatment or whatsoever in the DRTB case so a way of making sure that the home address you know of these clients is genuinely determined and with some level of wisdom in visiting them do you understand. Sometime some of them you know the phone contact they give do you understand you call again unreachable but there should be a way round it okay and that goes you know that touches on tracking the patients do you understand so apart from the what is it call now the nest of kin, you know to the clients we may, we can choose you know more than two if possible. There should be away of capturing the information of next of kin apart from the one on our own |
| Treatment supporter? | Yes, yes expertly we can have that do you understand so that we wouldn’t have this problem of having to lose patients you know to treatment or to follow up.  Now counselling; under counselling one thing I want to suggest you know we should do is you know the use of radiological evidence you know that is chest x-ray is very important you know usually we suggest our clients you know to chest x-ray at baseline isn’t it? That should be used as one of the focal point to convince the clients.Whenthe clients see you know their own film and they see the kind of damage on it, it is going to create a kind of make an impression in their mind because to some of them they don’t believe they have TB, they don’t believe so but when they see the damage, they will see normal now, we will copy normal with the one that is diseased so when we compare this two they will now come to the point I shared it to one of our zoom meeting, now stipend you know for baseline yes is if we don’t provide you know support to this clients do you understand where will they get the fund, we talk of end TB, end poverty isn’t it?, we want also end HIV isn’t it, so if that support is withdrawn then is as good as fuelling the problem more and more so the good thing that we have been achieving is to sustained so we should sustain that effort. Other loads are Constance in name of that should be withdrawn. I even want to advocate there some exceptional cases where you need you know for chest x-ray now will only be done at baseline for instance chest x-ray I just want to use that as illustration is done at baseline and at around 6months, 4 to months you know when you want to move to continuation isn’t it okay is done for I think end of treatment to confirm cure but I know now is only done twice. Before it used to be three now it is reduced to two film. What I want to bring our here is that sometimes while managing the patients, patient development you know some other funny complications which may be cardiovascular do you understand some of them you know they have heart failure complicating you know the problem do you understand some times some of them develop corecoconaliin cause of you know taken care of them complications in one form or the other. We need to track you know their progress with radiological evidences do you understand so we need that. So I think if they can be a way of making special provision for people even to some of them is just let’s say urinalysis sometime some of them they come down bilateral fiddlers oedema or leg swollen, so we need to know is it a renal thing do you understand I know for what is it called A & U that is been done before we seal the arrangement with electrolyte, it must have gotten far, you know that condition must have gotten farso these are the end of things I want to say and for the engagement of our liaison officer the best thing to have he just a coordinating person for us |
| Alright thank you very much sir. From what you have said I take it that the GX alert comes first may be in your ranking, so which one do you think should come last in the ranking? | In the ranking, if I de-rank anything here |
| No, not de-ranking but I said in the other of importance. May be let’s look at we already know the best which do you think should come last | This is a very big one, I can’t remove tracking of patients, I can’t remove CBOs, I can’t belittle any here, I don’t know, I don’t know. May be well for that of radiological if because of having to fund you know, I want to appreciate the partners and all the NGOs you know particularly this organization for what they are doing because is not really easy. You know we know what the economy is saying now the state of our economy so may for that and they want to put it you know as the lowest in that ranking |
| Is alright sir. Is okay sir. Let’s look at how complicated this decentralized service delivery is | There isn’t any complication about it. Well you see when you, you only talk of complication when you over centralized. When you decentralize you make the job easier do you understand, you bring about linkages, you make people to be more involved you know in the programme. That is why you get a better result. a quality result. You the patients when you decentralized because before now patients will have to travel do you understand our TBLS will have to travel, all that have been taken care of. All those things is within their reach within a short time we get all this things. Is it the GS expert alert that you are talking about everything you know is made seamless you know for us now, very unlike before |
| The decentralized DRTB | Is the best thing to happen |
| Alright | Look at governance I mean governmental system that we operate do you understand, Federal is there, state is there, Local Government is there. Federal is not going come and do the work of state, is the best thing to happen in any system or any organization, is the best |
| Okay sir, thank sir. Let’s look at the needs of your patients. Do you think that this system I mean the decentralized DRTB service delivery meets the needs of your patients | Yes |
| How? | It is meeting their needs |
| How? | One let’s look at it in the content of CBOs, okay that is part of the decentralization processes okay. the CBOs you know, they are the one that interface directly with the clients at the community level now community treatment and community diagnoses and treatment isnow in place so we can reasonably make diagnoses at the community level through the support of the CBOs and commence treatment even at that level okay, is not the space even at tertiary at special care unit for TB clients the space is actually limited do you understand and even the clinicians that takes care of TB cases is also limited mind you is one of you know the disease condition that people or whether clinicians or nurses you know doesn’t like at all, they dread it okay but if with what the CBOs are doing with the TBLS we can reasonably made diagnoses at the community level, commence treatment at that level, do you understand then patients to be taken you know to the OPD clinic you know for the monthly DC where we now review their cases and manage them accordingly. So that is very good. Now for which other one did you mention now |
| Well you have addressed how it meets the needs of your patients | The other way it meets the needs of the patients is I mean that token you know that is given to them is also another thing |
| Financial support | Yes, that financial support is also another thing even for the CBOs even visiting them do you understand supporting them, I mean encouraging them you know to treatment |
| And their own support is output based the CBOs | Yes, yes is output based that is also true, The higher the number of cases you know the more the support they also get, so that is also driving the process, it drives the process. |
| Alright sir. Now has there been a strong need? | There are a lot more to be said even as far as that decentralization is concern but is still okay |
| No, you can go on | Apart from CBOs, when you decentralize information gets you know to everybody at a faster rate within the shortest time possible because we all relate to information do you understand at the same platform do you understand, the TBLS know the need for them to open that thing and read every in fact if not more than two to three times even in a day because information will keep you know coming and that is one platform we all relate to do you understand and that in a way helped in treatment. It helped you know in processing all things you know everything we do even to the State liaison officer I mean once information comes from that level everybody you know will key into it and you know we take care of our clients’ treatment as soon as possible. Sometimes they say oh so- so- so patients has defaulted, so- so- so patients has done this, so- so- so result you know is this, we see all these things you know and there is a prompt response you know to them |
| Alright sir. I want to ask you now sir; has there been a strong need to increase or reduce the decentralized DRTB services in your facility? | We cannot reduce |
| Okay | We can only increase, we can only increase because why when you look at the number of clients that we get in our OPD clinic sometimes is over twenty, is over twenty. you see in a situation where you have many cases in your hand you know to manage you can’t put all that you know under a single hand or a single head you understand, so the issue of decentralization in fact is nothing to debate or to talk about is just to maintain it and to support it, to strengthen it that is all we need to do more do you understand and that has to do you know with the aspect of having you know that tokens you know that little support you know that is offered the clients. Let it be sustained do you understand, so that will make you know the clients to find it you know easier do you understand to come for clinic and to access treatment more. Sometimes you know when we give them you know that token let say the N2,000 N3,000 that you know we give from our own end here you see them they come to prostrate then you won’t imagine is it because of N2,000 or N3,000 that this one you know is prostrating, really they don’t have that transport incentive, they have so that is why you know they have to really continue to sustain it to maintain that process and the decentralization you know will help you know like I said earlier even the TBLS to relate better to their clients. It will help them to relate better to their clients. It will make them to be more involved in caring for their clients. |
| Thank you sir. So next question is on availability of support. So, let’s look at the supports that are available to you to adopt the decentralized system we have been talking about in your facility | Well, let’s look at laboratory, laboratory very close to our own OPD site Sabo, there is a private laboratory outfit that is working on the project okay, so the lab personnel they come directly to the clinic to collect sample. Is not to say that the client you know will walk to them, no they come directly to the clinic they collect sample let say their electrolyte urea, their, their what is called the just their blood you know for other investigations. Yes so they do this and even for those who will need to do, who has the need you know to do chest x-ray they go to the clinic those you know they can’t carry the film around so those ones you know will go to the clinic those who need ECG they will go to the clinic you know they must do a monthly ECG, sothey go to the clinic you know to run their ECG test do you understand so that in a way is, it makes the programme you know to be better. Is one of the support. Two the support of even the CBO like I said earlier is another one, okay. the TBLS and even the clinicians were also supporting you understand so the support that is coming from even the organization we can’t you know really over emphasised you know that measure that degree of support even to the programme. The support coming from the state liaison officer from the state programme officer on TB I mean that whole body of support from the ministry do you understand is something that we can’t actually measure. We can’t say it just in word. So, the support is really much from our own end we are supporting you know like I told you, you know monthly statutory release of N25,000 I think N30,000, I think N30,000 to support you know transport is even N30,000 to support their transport. We release it directly to the TBLSokay, so is one thing that made the programme to run smoothly because if you take away all those support, it is going to cause us a lot of problem and those things you know there enabled you know service delivery at you know particularly at their level the clients you know once they know that there is fundavailable you know that they are going to get something that is when you see increase in the number of flow do you understand of that particular clinic day. |
| Is alright so let’s look at the incentives. So, what kind of incentives do you think are available to ensure the implementation of the decentralized service delivery? Although you have already mentioned some of them | Well let me start with the service providers now. service providers you know the TBLS, myself as the clinician we come to the clinic we do the work do you understand, we also need you know support, we need support like to some of those TBLS they come from Akiyeni very far place do you understand though that is been offered, we get that transport support and that is very good do you understand, I’m only saying that that should be sustained that is what I’m saying. It should be sustained because it drives you know it motivates |
| Yes | It motivates. The other thing is the incentive given to the clients we need to sustain that do you understand, that tokens you know for transport should be maintained. It should be maintained and it should be made timely, it should be made timely do you understand that will encourage you know that clients you know to come without any excuse do you understand so one of the incentives. Then the other incentive it is not just about money; you know some of those tools that the TBLS need to also work do you understand, it should be provided because when they don’t have you know the tools that can be a factor though is also available but is should be provided. |
| Alright. Yes, you know we are looking at the available incentives. The ones that are available already | Okay, for whatever they are doing, you know the organization are doing their best and we are also doing our best, the CBO is also doing but we need to improve more on what we are doing that’ |
| Is okay so let’s look at how the setting or the culture in your facility, cultures, your facility culture how there affects the implementation of the service delivery the decentralized service delivery? | Thank you. You see there is a way we do it, the TBLS of the respective LGA okay they put a call, they have the contacts of all the clients do you understand so they get to them for instance you know for the OPD clinic they contact them directly and they send them reminder for the clinic. The clinic held last Thursday of the month but sometimes you know we change it so if that is going to be changed due to one factor or the other we communicate with them directly because they have their contacts. That is helping a lot do you understand to bring all of them together do you understand and when they called, they must comply with the infection prevention and control strategy |
| Preclusions | Preclusions yes. You know that precaution they must, there is water stand by for hand washing then they must put on their face mask, their nose mask they wear their face mask do you understand. That is very compulsory to all of them. It may be common face mask you know or sometimes the earn 95. Sometimes you know some of them they put dirty mask I just you know give them the earn 95 you know I will tell them to remove the dirty one and I will offer them the earn 95 because for them to also put on dirty mask is another, that also has implication. I do that often to them okay is one of the culture that we maintain there. Then the sitting arrangement we make sure that you know they sit with space you know spaces in between. We don’t allow them to just you know, anyone you know because some of them they yet to convert do you understand to negative, ehee to TB negative so those ones you know we just tell them no if you are still coughing actively we wouldn’t allow you still sit among those ready you know that culture result you know has come two to three times negative because we don’t want to get them re-infected again so these are part of the culture of the environment and all the TBLS you know they come they relate to their own patients when I want to see let say a patient of xx the xx focal person TBLS you know for xx will be on her to bring the patients with all the information card you know at that points you know |
| And all these helped to facilitate | To facilitate you know the treatment you know |
| And the implementation of the decentralized programme | Of the programme, yes. |
| Is alright. So let’s talk about your motivations. What has been your motivation | Okay, thank you very much you know what I have been let me say I’m a clinical oriented Doctor. I’m a director in public service a grade level 17 at a time we had this training at Jericho for South West you know doctors okay, as you know for South West doctors I did very well in that you know training do you understand I happened to be the only one the pick in the State that is number one motivation. Okay when you somebody who is outstanding do you understand in performance clinically and you just you know say okay recognize that person and you chose him out of the crowed. I was the infection prevention and control specialist in Oyo state as well so they made that choice for my person that stand as one of the major driver. Secondly my person I’m not you know the type that you want to say the type of person that you know that hankers after money yes so I’m interested in result, in taken care of my clients okay, so taken care of my clients do you understand so in in in at the Sabo OPD clinic do you understand I do visit all these facilities do you understand with my interest in TB care even before now wehaving a form of training you know even prior to the intervention of organization do you understand all these you know has wholesome donereally you knowreally motivated me a lot. Is not for gain because if somebody is going to work in TB you don’t work you know because you are looking for money no because you would want to help. You would want to help the sick and this has been the major motivation you know to me |
| You mean the motivation have been there to help you ensure that the program is implemented? | As we are sitting here now you see that clients are already waiting outside. I’m a person like that and am not for any fund or anything. Let me say I introduce free hanouracy in xx State even at local government PAC level. I operated 62 persons free of charge you know in xx before the State Government took it up, 62 people for Harnoracies I operated go back to year 2000 do you understand so is just self-motivation, is just you know in built do you understand in everything depends on your background and your person, you know you person so these are just the simple thing I would want to say as a Christian too contentment and to care for my fellow again those are my motivations |
| Is alright sir. So let me ask you how confident you are that you are able to implement the decentralized service delivery regularly in your facility | Thank you very much. You see is not one man affair do you understand, is synergy. That effort must be synergized but when you talk of my own personal commitment to decentralization I will say 100%. 100% committed to it and I am confident with what I am doing do you understand but we need other hands as it has been coming those hands and support you know like for instance was it not last month you know we had this training for all clinician in the south west on how to read x-ray in managing the clients. We heard that at XX house there at XX do you understand I was there and the only person from the local government so that has you know added more to my knowledge in taking care of all these clients do you understand so is a support I cannot relent on I will continue to support and I am confident with what I’m doing. The clients are in a better position to even say to it which doctor has you know the calmness coordination you know the patience to live in toyou and to take care of their condition they know |
| So how, let’s talk about the confidents in your colleagues. How confidents are you? | You mean among my colleagues |
| Yes amongst your colleagues been able to implement this | You mean for my colleague to implement or for me to implement |
| For your colleagues | To implements |
| Yes, so you have talked about your confidence in yourself so this questions is about your confidence in them to be able to also see to the effective implementation of | Yes they will buy into whatever you bring in for them to do. One of unfortunate thing is presently we are having a decline in the number of doctors, there is a decline in the number of clinician even at XX do you understand we started with 33 presently I think not up to is it 13 or there are about now okay, so as far as clinical care is concern I may not I don’t want to hold brief for anybody I only know of myself but as supporting TB care TB decentralization they will ready let go by the virtue of their position as coordinators or directors in the system. We will readily do if challenge now the will release fund now for these purpose I bet you they will commit to it |
| Is alright, So the decentralized DRTB service delivery how well does it fit into your existing processes and practises in the facility | Well you know because I told you earlier that I’m clinically inclined person do you understand is not just about management of each at office level do you understand to be taken office decision that I’m trained for as a medical doctor do you understand thank God here by my own position I support the training of resident doctors even at each level resident doctors I mean. I lecture final year medical students do you understand I give them lecture okay in their final year 600 level students I do. I support the training of other students you know for other institutions here |
|  | You see when you see a man that is multi task yes so I am, I am multi task, when you say training, support this and that I am into that, when you say caring for patients I am into that, it depends on the joy you derive in what you do and that has been my pleasure so far in offering the best you know to people in whichever area you want to think of |
| So sir are you saying that the decentralized system or service delivery fits well into... | Exactly into my, into what that’s what I want to say by implication. That’s what I’m implying that look I’m a man that is multi task so definitely my interest you know spread into all this you know domain, do you understand |
| So finally we want to look at how do you want the decentralized system to replace the current programme or do you want this new system that is decentralized system to complement the current programme | Well, I see the decentralized system now as been invoke that is what we have invoke now I don’t even know of any other system that is in place, so if there is another one which of cause you know we have moved you know from Egypt we are in Canaan, isn’t it |
| So, they should be absolute replacement | I mean I mean I mean, is something you know to just sustain and made to work |
| Is alright sir | Thank you |
| Thank you very much | Thank you very much sir |

**KII/A5**

| **Interviewer/facilitator/questions** | **Respondent/responses** |
| --- | --- |
| So, let’s start with the preamble which is your thoughts on TB Reach Wave 9 Interventions to decentralize DRTB services, what do you think about it sir? | Thank you sir; for the TB decentralized DRTB services, what I have observed most or I like about it is that it will be very easy in some areas to be able to achieve some things like maybe the Doctor attend to the TB, maybe the distance is far to where the TB patient is, been decentralized it will be easy for the nearest TB survivor or a person that is well trained to be able to attend to the patient before transferring to the hospital because a little delay may affect the TB patient but been decentralized, it will be able to achieve more than what we are achieving now |
| Okay, so you mean that decentralization takes the TB treatment closer to the people? | Yes Sir. It will even support the one they are using now before decentralization. |
| Is there anything you dislike about decentralization or something you like? The List | Although in everything there’s advantages and disadvantages. The advantages to my own side are more or 50-50 with the disadvantages. At least, in the advantages it will be easy to communicate like someone that is more than a kilometre to the patient and someone that is 5-10 kilometre to the patient, the communication may not be easier for the patient but someone that is very closer, it will be easy to attend to the patient then before transferring to the hospital because not every centre is more closer to a TB patient. So the advantage is that if they are using politics for the decentralization it will affect most like someone that’s not in the line or not being trained and they put the person into that side, the person may not be able to satisfy to what you expect maybe instead of 98-100% it will be like 10% by 100%. It will affect, that’s the disadvantages but the advantages is that if they do it well, the person that is meant to be in charge, apart from doing the hospital or the healthcare centre, it will be easy to communicate and it will give some people a job because they are some people now that finished from school and have the experience of treating patients it will expand them to use their knowledge and what they learnt to support. |
| What has influenced your practice of decentralized DRTB in your state? | First thing first, I will have to say thank you to the doctors, all the nurses and some colleagues and friends because without the doctor giving the advice on the way out, I may not be alive today because they all helped me a lot during the time I’m taking my drugs and then I realised that if such people can the way they did to me I can be able to do to some. at least they are some people outside there that they have it even it may not be TB alone maybe the other side and they do have some maybe they encourage them to support them but at least seeing myself passing through that stage I have experienced a lot that it’s good to be closer to someone no matter how the situation to give them the courage so they will be able to survive more because many people lose hope. This thing I don’t have any hope and what I will face outside after getting through this but thank God it has encouraged me a lot in advising even I’ve seen some patients apart from main TB their way of thinking is somehow but I tell them that’s not the best, your focus is worth most remember the children and your family around you. The way you did now, give them courage to support you but if you’re not giving them the support they need they will be discouraged too. |
| What helped you the most? | What helped me most is the treatment they gave me, the support and the advice. |
| Have you encountered any challenges so far? | So far the little challenge I encountered was that time I lost some customers been the work I’m doing, the time range maybe because I’m been treated and I may not be able to attend to them. |
| I understand but you know you are part of this somehow because I understand that DRTB survivals somehow have a role to play in the whole interventions, I’m asking if you have experienced any challenge? | The challenge I experience, the time range sometimes maybe today now I have already have appointment but maybe there’s a patient maybe at that particular time I need to attend to. The time have affected the two appointments, I wanted to satisfy this and I wanted to satisfy this so the problem I have that time is the transport range and the communication is somehow because it’s not easy but there’s a way of making it easier more maybe with the distance, there should be a mini video just to maybe there’s a distance for the counsellor that’s going to counsel the patients, there should be a short video that the guide the patient a little bit before the arrival of the counsellor. Let’s say you go for a seminar, before the seminar starts, there’s a video being displayed, explaining some information before the arrival of the person. It’s to give the person that’s handling the patient a little moretime; you would have already known that the video their playing or the time range is maybe the next 30mins. |
| Let’s look at the Intervention Characteristics, so how does decentralized DRTB services compare routine existing programmes in your facility? | Decentralized is good while the existing routine programme they are using is good but they use decentralized to back the existing programme they are using. |
| Can you talk about the advantages of decentralized DRTB services? | Like I said before the advantages like a doctor or a nurse now, they are in their office maybe attending to some patients already and getting calls sometimes it won’t be easy for them to quickly roundup what they are doing and to move but being centralized there will be a communication between the doctor and the nurse to call hello TB survivor are you around? Where is your location? Give me the nearest TB survivor to that location. It will be easy to communicate to the patient that they want to attend to like let’s say you meet them here now and you need to attend to them, at that moment for them to be able to move down but between a second of maybe we’re coming, the person may move away from that side and you will not see the person again. |
| What kind of changes do you need to make for decentralized DRTB services to work affectively in your facility? | The change I will say they needed to make is the communication first because to communicate with the patient is through from one person to another at least if the doctor is not able to communication with the patient or didn’t know how to there should be an intermediary to the patient because the doctor too they are trying at least 24/7 they’re on their way of inside hospital attending to some patients but they should be a way of talking/calling hello, I’m calling from this so-so centre , please kindly go to this so-so place, attend to the patient. Whether the patient needs to be attended to maybe through drug or to check the position the patient is. If it’s to be moved immediately or there’s a little thing to give the patients till the next day, they move the patients down to the hospital because they are some patients that they will be a type that before they know that the patient is having issue it have been. |
| Now, you know about the components of this intervention, are there some of the components you think should be altered or changed or they are okay the way there are? | It’s okay. |
| Do you think that TB service delivery decentralized approach that it is complicated? | The complications there is if you’re putting someone in charge of decentralization maybe this so-so person is in charge of this local government, let the person be trustworthy because being not trustworthy it will affect a lot. The person that we are putting there should be person have specialised already, have experience, knows what is to be done and what is not to be done. If we can work on that it will be okay. |
| Now, how well does this approach that you’ve been talking about, meet the needs of the patients? | It depends because we human being we are not the same. In some people’s view, what they expect, it may be patient that is being attended to maybe some incentives some things they are giving them, they are some people that will still be saying that it’s more than that. They are some people packing it or they are some people cutting it off. |
| But in your own opinion, do you think it meets their needs? | Yes, it meets their needs because do you know the time they attended to them, all they are giving them is support, the contribution are helping them. |
| Has there being a strong need to increase decentralized DRTB services in your facility? | They are many things. There’s a strong need like patients now being inside the world at least we are not all the same. They are some like being at home alone they like watching TV, listening to news from time to time even they are some maybe events happening outside they need to be seeing too at least they should provide something like maybe TV, something that will be giving them courage at least they will feel at home.  Even me there’s an experience I had during my visit there, they don’t feel like staying, they prefer being at home. But once they reach there, they see the light, the way everything is well arranged, they have time to watch news or they want to have time to themselves, they are that every minute every seconds they like eating, they prefer to cook by themselves, have their own kitchen but at least if they have been able to provide a little that avenue for them maybe they have access to TV and some things that okay we’re at home without being at home and some may even say I feel like eating this apart from what they give them maybe I want to have a taste of myself maybe this is what I want for this time, they will even have access to have a better life like being at home. |
| Okay so, let’s look at the support that are available to help you adopt Decentralized DRTB in your facility? | The available now is the support being received from the voluntary organisations, they are giving out at least that will encourage more to some patients at least once they reach out, they see someone like they are in that their shoes. Their having that TB. |
| I’m talking of the support that’s available to you? | The available support is time to time being called by doctors, how are you doing, how’s everything, are you available, can we see, please can you get me these products? At least they give me something I can earn, this is my job, they call me and they pay me and they of me time to time, that give me more courage that this people they know I’m somewhere and it helps me to adopt decentralization. |
| What kinds of incentives are there to ensure the implementation of DRTB services are successful? | Incentive of let’s say maybe they call me to somewhere now, they need my attention or they say help us to attend to this patient and they compensate me with some amount like this is your incentive for answering us. Incentives like let’s say money because someone that is coming from far or near no matter the distance you encourage the person more although it’s not every time. |
| You mean sometime you receive financial incentives from doctors? | Yes, to support and to encourage more. Even if they don’t give, I will know it’s not available. |
| How do you think your facility setting affects the implementation of Decentralized DRTB services? | No, it does not affect me, any time they call me or I visit the place they always welcome me. |
| What has been your motivation for wanting to ensure that this implementation is successful? | Many things let’s say apart from the incentives, the treatment I got is superb. If they say this is where you’re going, once you reach there no delay, they attend to you straight. Once they see your card being referred from so- so place they will attend. |
| How confident are you about being able to implement decentralized DRTB services regularly in that facility where you’re attached? | Yes, it will be a great privilege for me. Although I’m an electronic engineer but right from day one I’ve loved to help people no matter what the situation so I’m confident. |
| How about your colleagues, how confident are you that they will be able to implement this approach? | I can always say within my reach, I don’t know other parts. I can always say the people I’m working with from the doctor and the nurse we understand each other, we are able to organise how things works. |
| Are you confident that those doctors and nurses will be able to implement decentralized services why? | The people I’m working with now, you know times change unless they change the one I’m working with to another department but those I’m working with now I’m confident in them. |
| How does Decentralized DRTB service delivery fit into your existing work processes and practices? | It doesn’t disturb my work because it’s what I love to do, helping people and encouraging them. |
| Do you think that Decentralized DRTB services should replace or complement the current programme process? | I think it should complement and not to replace. |
| Why do you think so? | Right from day one, before in the olden days we were using book, now we are using phone and we did not remove the book. No matter what you do with your phone you must go through with the book too being document so it will align together. If we say we should forget about the existing one and go to decentralized fully many lapses will come up because being centralized it will not be easy. There should be a particular place that being centralized all their works will be delivered to, it’s from that side they will now deliver the best. From that side they will return what is good to each department. So being centralized will not be easy if we forget about the existing process, it will affect a lot. The communication won’t be good but complementing what is going on now it will support, it will give more courage, it will even relieve them of some stress, some activities that’s being piles up together and it’s affecting some things. It will ease the desk being piled up of too much books. too much information, too much things to attend to, at least each department this is your own specialization, you’re to distribute drugs, this department, you’re to attend to the patient, this department, you’re to visit the patient. It will be easy, it’s not like it’s only one department that will be doing everything it will be easy for each department and there won’t be problem. |
| Thank you for your time and responses. | You are welcome sir. |

**KII/A6** 4

This is the Ibadan North and the interview is with Ibadan north TBLS, it is been conducted by Dr. XX representing XX.

The focus of this interview will be on the effectiveness of the TB reach wave 9 interventions to decentralized DRTB services so we will also be looking at the challenges and we will also express any imagine issues in the cause of this interview.

IT; So, ma, we will start by asking you what you think of TB reach wave 9 interventions aim at decentralizing DRTB services.

RESP; decentralize DR patient is good because before it was between the state team but now that they have decentralize it they are able to monitor the patient very well because after the treatment center 2 weeks they will send them back to the community so we are the one that is taking care of them because they are living within the facility environment so we are able to monitor them very well.

IT; okay can you tell us what you like most about these interventions?

RESP; we are getting the result immediately after the result in the laboratory, they will send the message to the vocal person DR, immediately present it in the WhatsApp so the TBLS or dot officer that is in charge of that patient, they will quickly call the patient that your result is out please come, so they have started the baseline examination

IT; so, treatment initiations delays are reduced

RESP; yes

IT; does it have any effect on follow up

RESP; we don’t have problem about follow up because immediately they started the treatment, because they will send them back to the community, so we are monitoring their follow up, culture, baseline everything

IT; is there anything that you like the least about the intervention? Let’s say what you don’t like very much about this intervention

RESP; its good because before the laboratory can diagnose 10, 20, before we can see 10 or 5 to put on treatment it was very difficult then but now things has been changed when this intervention has come in so we don’t have much challenges again

IT; okay but what affected or influence your practice of decentralize DRTB services in your state?

RESP; in my State?

IT; yes

RESP; Because of the loss to follow up, delay in result even most patient will die before the result came out.

IT; do you have any challenges that you have encountered so far?

RESP; Like this message from the laboratory immediately they diagnose DRTB they will send the message to me but the message all the message was not completed, the name of the patient is not written there, the facility name is not there. They only told us the site, G expert site that diagnoses the patient.

IT; it is the same thing that has helped you in the implementation?

RESP; you said what?

IT; anything that has helped you? Any factor that has been of use?

RESP; When this organization came in so things has been changed because immediately, they saw the patient they will call Mr. Shittu, Mr. Shittu will immediately call the supervisor or TBLS or DOT officer that you have a patient so so so name, so will start the test, the baseline test culture and everything.

IT; okay. Let’s look at some intervention characteristics so I will like to ask you how you can compare the decentralize DRTB service delivery with the routine program in your clinic

RESP; routine program?

IT; Okay the routine program is the usual way whereby the formal was more or less whereby everything is centralized, the investigations, the test is received only by the state program manager and the state DRTB vocal person and now message is sent to

RESP; this decentralization has helped us so much because before we get to know that we already have patient, at times it may be 2 or 3 months, a month at times but now all these things has been waved out so now we get our patient immediately they diagnose them on the baseline done so early diagnosis and prompt treatment

IT; yes, but sound like advantages of this interventions and I don’t know if you can elaborate more on advantages of this interventions

RESP; like one time they gave us sputum examination,. That sputum examination has the next of kin name, next of kin phone number because at times when they call the patient the number that the patient gave us when he first came for the presumptive, it might not go or wrong number. for this sputum examination that produce by the organization we are able to call the next of kin number that so so so person came to our clinic and his or her result is out so he help us so much to track our patient immediately the result is out

IT; are there some disadvantages you can think of about the decentralize DRTB service delivery?

RESP; Well, I don’t have for now because it has been helping us very well, I don’t think there is any challenges what I can say is for the organization to put more effort so that because of we are having challenges about our patient. Most of our patient they don’t have much, they are having problem to eat, they cannot walk, to take their drug is problem, you know this drug is too much so if they can help us in that area not only tracking the patient

IT; it’s alright ma. But okay let’s look at the changes you effected or you had to make in your facility in order to make sure that decentralize DRTB works effectively. Where just certain changes you needed to make so that there will be smooth and effective implementation of the process

RESP; I can say about this feeling of sputum form because before those our rider and dot officers, anytime they fill the sputum examination they will not fill them correctly or completely but now that this going to the laboratory, go and pick some for random checking, Mr. GBODEGBA, Mr. SHITTU. So, we have learnt a lesson that we must make sure that this sputum form, we will fill it completely and correctly so that immediately the result is out we will be able to trace the patient

IT; so that’s like sharpening the skills of your staff

RESP; complete filling of the sputum forms, then retraining the dot officers on how to fill the sputum forms

IT; okay. Other changes maybe infrastructure or

RESP; this sabo clinic we are having OPD here for DR patients so we are able to collect their culture monthly, we take it to the laboratory to monitor their treatment

IT; it’s okay. Now looking at the component of this intervention, are there ones you think should be change or not? If you recall that there are some of these components like devolving the GS alert to TBLS or the patient

RESP; anytime they want to send the message to us, they should make it complete so that we will know where the patient is coming from and the facility that took the sample of the patient to the laboratory so that we will be able to call the person in charge

IT; any other component you think they improved on or changed to meet the demands of

RESP; the training of those patient that has completed their treatment so that they will be able to counsel the main one that is coming in for the treatment, regular training. DR patient

IT; okay. Now how complicated is this decentralized DRTB service delivery?

RESP; complicated?

IT; do you think the service delivery is complicated or complex? Is it difficult to deal with?

RESP; no. not at all. This intervention makes it easy for us to see our patient, to monitor them, they will be able to come to collect their drug regularly unlike before they will go to UCH or Jericho to collect their drugs

IT; so, you are saying that the service delivery is

RESP; it’s okay.

IT; now let’s talk about whether the decentralize DRTB services meet the needs of your patient

RESP; like how?

IT; you may have mentioned it, you’ve talked about some of it in way. What I mean is their medical need which is addressing

RESP; yes now

IT; or whether you think that there is, do you understand what I’m saying? so you just talk those needs whether it really met by the service delivery

RESP; yes now. Their needs are been met. They are collecting their drugs regularly, all the routine something is going on very well so these interventions meet their demand. They don’t have problem.

IT; sorry, can you summarize this point you made now, you know we are being distracted.

RESP; you ask me that this intervention they gave me the demands of the patient and I said yes because they don’t have problem before they get to the facility they are collecting there, they don’t have transportation problem unlike before I don’t have money I don’t have money because they have decentralized them to their nearest home so and all those routine something is easy for them to do.

IT; alright thank you. In your facility has there been a strong need to increase or reduce the decentralized DRTB services?

RESP; strong?

IT; strong need, either to increase or decrease or rather to reduce this decentralized services delivery we are talking about.

RESP; no

IT; yes, I’m listening

RESP; we don’t have now problem with the patient.

IT; 0kay so are you saying now that there is a strong need to increase the services?

RESP; Yes

IT; can you just throw more light so that it will be clear?

RESP; because we don’t have shortage of drugs, they are collecting their drugs and doctor is here once in a month to check the patient maybe they have any complain or AEFR advert drug reaction and doctor treated them, we don’t have problem

IT; So, you think the service delivery should be increased?

RESP; yes

IT; Now, what support are available to help you to adopt decentralized DRTB service delivery in your facility?

RESP; support?

IT; Yes

RESP; You can support us in social something, you know I have said it before when we started the interview

IT; I’m talking of supports that are available.

RESP; that are available?

IT; yes, the ones that are available

RESP; okay the ones that are available. We don’t have any support. Like what?

IT; what I mean is things that has helped you, factors maybe enabling factors one way or the other, whereas let me give you an example some people talked about the fact that there is a WhatsApp group

RESP; okay, okay

IT; has there being trainings, yes things like that.

RESP; okay there is, there is

IT; so, you can tell me more about it.

RESP; like the one we did, factual training we have some factual training from the organization on how to fill the form then supply of sputum request form and sending message to us to know anytime we have DR patient on WhatsApp so that is that.

IT; so, what use has that WhatsApp group being to you in your facility in the implementation of this service delivery?

RESP; it is good because before we have the hard copy of the result you can see the result through the WhatsApp group, so so name is there and patient so we started calling his or her number so that we start the baseline so that we will not delay the treatment.

IT; okay are there other things that are been of use maybe within here, things that have been quite supportive?

RESP; like OPD that the doctor normally came here once in a month to see all these DRTB patients from 5 LGA like Egbeda, North East, South East, Ibadan North so that one really helps us so the patients too they are eager to see doctor anytime they have complained they will come and see doctor then tell the doctor their complains and they were treated according to their complain.

IT; what kind of incentives are there to ensure the implementation of this decentralize DRTB service delivery?

RESP; incentive from where sir?

IT; remember before we mention certain incentives some the patient given to, there is this outlay-based incentives to the TBS. they are some of the things that have been put in place to see to the smooth implementation of the program

RESP; to the patient or to the workers?

IT; I want you to talk about any incentives you know about

RESP; like anytime we have a patient normally send communication fee or something like that 3000, 2500 at times they send it to the TBLS so that we will be able to look for the patient so that we can be able to start the treatment immediately. They send money for us for communication from the organization so I’ve been benefitted so many times

IT; any other incentives you can think of?

RESP; I cannot remember but I remember

IT; now let’s look at the setting of your facility. Setting or the culture around here, how do you think it has helped implementation of decentralize DRTB services?

RESP; like this community, you know we are dealing with hausa people. This community is hausa people that’s why we call it Sabo, so they are very difficult to deal with because they don’t want to take the drugs they don’t want to go far before they can access the treatment so this decentralization is helping so much because they will come quickly to take their drugs. You know most of them they are beggar so they don’t have much time to go and stay in state hospital to sit down for 1 hour, 2 hours. So, it has been helping us so much

IT; you know every facility, every organization has where they do things have their culture, have their setting maybe environmental setting their workers. I want you to look at it from that angle. The way you do things here is it has it in any way either improves or it reduces the implementation DRTB services?

RESP; in this Sabo we don’t have too much, I’m talking about my community, we don’t have much DRTB patient here, Mr. Shittu can testify unless they transfer them from another LGA after the treatment center they said that I want Mrs. Bilau to help us manage this patient, I have patient from Ologuneru, from another LGA that come here for treatment so in here at Sabo they are very very difficult patients, they prefer to go to another place because they don’t want people to see them that they are having even DSTB talk less of DRTB

IT; okay are you saying that the location, the environmental setting here doesn’t encourage people to come around?

RESP; yes, we have one couple here, they are couple husband and wife, they are DRTB patients they have never come here for OPD meeting since they started the treatment to the end of the treatment, although we are collecting their culture monthly baseline, we are doing it but to come here to come and sit down with DR patient for those community to be seeing them that they are having this they are having that no. they find it difficult to come

IT; so that means in a way, it hinders the progress or the

RESP; it’s only this community the hausa people not other DRTB patients that is coming here

IT; okay. So apart from that you can say that the implementation is running smoothly?

RESP; yes sir.

IT; so, let’s talk about your motivation, so what have motivated your wanting to have to ensure that the implementation is successful? What motivation have you had?

RESP; because this disease is a curable disease, if the patient takes her drugs regularly it’s a disease that cure. When we see a patient, when he wants to start the treatment at times, they cannot able to walk but immediately they started the treatment they will get better because there are drugs there is treatment for it that’s number 1 that motivate me this man that you have been that is like he wants to die see him now

IT; the joy of seeing your patient do well

RESP; getting better

IT; any other motivation?

RESP; at times we use them to counsel those that are just want to start the treatment, every day that we are having our OPD we will use those that are about to finish their treatment to educate those that are just coming that please take your drug regularly if you don’t take it, it’s problem. Eat very well if you see me when I want to start my own, you will see that this man wants to die but see me know I’m okay I started with maybe 39 or 40kg I’m now 65kg

IT; okay using the DRTB survivors

RESP; yes

IT; good. So how confident are you about been able to implement this service delivery effectively?

RESP; I don’t have problem. We have started too long; I don’t have problem to deal with them

IT; so, you are saying you are confident to be doing the services regularly in your facility

RESP; yes, sir we have been trained and we have manual, if anything is not clear to us, we will take our manual so we go and look for it to check for it inside the manual.

IT; how about your colleagues or your staff?

RESP; here?

IT; yes

RESP; I don’t have colleagues, here

IT; let’s talk about your colleagues generally.

RESP; I have dot officer, the man you met when you

IT; in your facility?

RESP; Yes.

IT; Okay. Are you confident that he should be able to implement the services regularly?

RESP; for DR?

IT; yes

RESP; No

IT; you don’t have that confidence?

RESP; no, I don’t have that confidence.

IT; why?

RESP; he can deal with DS I’m okay I don’t have problem but for DRTB I’m not confident.

IT; why? Explain.

RESP; he has not been trained for DR he has not been trained and you know we have WHO drug, we have B stream, now they want to change it to be BPAL abi Mr. Shittu so they have not been trained I cannot leave him alone to handle the DR patients.

IT; is it that he doesn’t have the interest?

RESP; no, he has but you know he is the rider he didn’t have much time; he is only the rider for Ibadan north they can call him from Adeoyo, Agbowo, he has so many DOT facilities so he is the only person that go around to collect the sample, take it to the laboratory that is it. He is not a permanent staff; he is the rider.

IT; okay, let’s look at how the service delivery I mean the decentralized DRTB services, how they fit into your organization or your facility what I mean by that is do you think that it fit into your existing work processes, the practices.

RESP; yes, we have been doing it for so many years so it’s fitting.

IT; it doesn’t have any problem with your work schedule?

RESP; I don’t have any other job here it is only TB, DS and DR, we don’t work in the ward, I don’t have any job, we are for TB alone so it cannot affect any other thing.

IT; it perfectly suits your practices?

RESP; yes sir. I don’t have any other schedule.

IT; okay but do you think that this decentralized DRTB services that it should replace or complement the current program or process?

RESP; no replace like how?

IT; you know we have talked about this is decentralized, the routine one is the one that

RESP; that we are doing before

IT; yes, so my question is that do you think it should be replaced?

RESP; no

IT; or exist side by side with it or complement it?

RESP; we like it as the way it is now

IT; which is?

RESP; decentralization.

IT; okay so are you saying that we should continue with decentralization only or that we should marry the two maybe complementing each other that is my question. It’s either you choose one and then you explain.

RESP; decentralization is good, you know that one we have been using it before and we are losing so many patients so this one is okay, decentralization is okay.

IT; so, you think we should replace the former one?

RESP; yes, the former one, it should replace the former one.

IT; why?

RESP; I’ve said it now.

IT; sorry

RESP; we have so many, so many

IT; advantages

RESP; yes advantages, thank you they have derived from this decentralization unlike the one the way we are doing it before, so you know this DR this thing we are talking about we need early detection and prompt treatment, that is it you know this DR is airborne disease before they can come to the facility taking their sample take it to the laboratory, you know so many people will have been affected.

IT; so, this particular one helped achieved that?

RESP; ehn ehn early diagnose and prompt treatment, that is it

IT; thank you very much ma.

RESP; thank you sir, you are welcome, sir.

**KII/A7**

This is Ibadan South WesXX Local Government and the interview is with the local government DOT officer and DR XX, a consultant with XX will be conducting the interview

IT; Sir, let’s start by knowing what you think about this intervention which you have explained before.

RESP; the decentralized service?

IT; Yes, the TB reach 9 wave interventions which are aimed at decentralizing DRTB services, what are your opinions or your views about them?

RESP; I think about it is a good program, it’s a good system because it helps to get the patient on time and start their treatment on time unlike before.

IT; yes. So that means there is increase in treatment initiation

RESP; yes

IT; Okay, are there other things reasons why you think they are okay?

RESP; I think the aim is to get the patient started their treatment on time and get well and be okay. The DOT officer receive the result immediately and then contact the patient then all other processes be done on time for the person to start their treatment on time unlike before, we will be waiting for the lab and the Ogas that they first send the result but now, the result is send directly to the downs something so that we get, you know we are the one that is close to the patient, we will be able to get them treated on time to start their treatment on time.

IT; it is okay. What do you like most or the most about the interventions? What do you like the most about the intervention?

RESP; the aim of the intervention is a good one, I like it. Because like I said it helps us to start treatment on time.

IT; okay, that’s what you like most, is there something you don’t really like about it?

RESP; no.

IT; what has influenced your practice of this service delivery, any factor that has helped you?

RESP; because I love my patient to be quickly get treated and be okay so I welcome the intervention program, you know their own efforts too is to eradicate the disease in the country, I support that initiative and then i contribute my own quota and it help me to contribute my own quota to the eradication of the diseases by calling the patient to get their treatment on time.

IT; have you had challenges so far, in the implementation of this services delivery?

RESP; no

IT; you have not encountered any challenge?

RESP; No.

IT; okay, now let’s compare these types, that is the decentralized DRTB service delivery with the existing programs, what we can now call the former one, the centralized one so let’s compare the two.

RESP; the centralized one

IT; and the decentralized approach? How can you compare the two?

RESP; the decentralized approach helps us to get the patient on time, like I said.

IT; you mean this decentralized approach?

RESP; Yes. Unlike before, the lab personnel will first of all call them at the state before they get to the DOT center, at times the information may not get to us immediately or quickly.

IT; but what happens now?

RESP; what happen now is that we got the result directly from the lab so we will be able to like one day

IT; do you get the SMS from the automated machine about patient, from the machine?

RESP; yes, from the lab.

IT; Direct from the machine?

RESP; yes. No oga got it and he told me to call the patient

IT; okay that’s the local government TBLS

RESP; One day, the patient come for the result and immediately the patient left so the result come from the state, supposing the result comes to me or comes to us directly, it should have been track down that day before he left. It takes some days before you can get the patient for the treatment so the intervention is very good.

IT; okay, are there other advantages does it have any influence or impact on loss to follow up?

RESP; yes, it has.

IT; has it increased it or reduced it? Has it increase loss to follow up?

RESP; it reduces loss to follow up, because if the result doesn’t come out in time so the patient may go for any other means of getting himself cured whereas the step is a wrong one. Now the result comes as quickly as possible then we will be able to track them.

IT; do you think there has been improvement in the treatment outcome

RESP; yes, there is.

IT; do you think of any disadvantages, can you think of any disadvantages of decentralized DRTB services?

RESP; it is very advantageous.

IT; so, you can’t think of any disadvantage?

RESP; Yes.

IT; okay. What kind of changes did you make or need to make in your facility in order to implement decentralized DRTB services?

RESP; they are sending the result directly to the TBLS

IT; yes

RESP; they should include away dot officers by sending the result to us or let us know immediately the result is out so that we will be able to get in touch with the patient, they should include our number to the contact number.

IT; it’s alright but in your facility on this office, were there changes you made so that this DRTB work very effectively, will succeed or that it will be implemented very effectively, were there some things you did? And did you train any person? Were there trainings? Did you have meetings, did you visit mall? Do you have a WhatsApp platform? Were there things that you did here so that things will work better?

RESP; no, it is only whatever the TBLS told us or informs us to do.

IT; you didn’t observe any changes in your facility in order to implement this effectively?

RESP; no, we follow the existing one and it works well for our patient.

IT; are there component of this intervention that should be altered, are there some of these components that should be change?

RESP; no, it should continue like that.

IT; Like it has quite a good number of components including the fact that there is, like now the TBLS get message directly from the automated machine from the center, now there is also there is CBO’s that have the incentives based on output and also support to patient. So, my question is all these, will you say they should all be left as they are, they should not be changed?

RESP; they should include DOT services

IT; Yes, you have mentioned that

RESP; Yes, I have said that one and then at times you know our level of this thing, we might at time needs some incentives like data, training to buying data, at times we might not have data on our phone because of our income, low-income cadre, we need training then we need incentive.

IT; how complicated is this decentralized DRTB service delivery? Is it complicated?

RESP; it’s not complicated

IT; why do you say it’s not complicated?

RESP; because the system doesn’t give us any problem of fatigue so we carry research

IT; how does the decentralized DRTB service delivery, how does it meet the needs of your patient?

RESP; by bringing it down to the local level, to the DOT to the facility. It helps the patient to get their treatment at their door step, very close and very near to them. you know many of them use to complain that they don’t have money for transport, at times they don’t have money for food but bringing it down very close to them so they get their treatment and their drug very easily.

IT; so, has there been a need to increase or decrease the decentralized DRTB in your facility?

RESP; there is need, because we need more training and enlightenment at times, we need training or enlightenment on this system, in this program so we will be able to work effectively on it.

IT; okay. I said whether there are been a strong need to increase or decrease DRTB services?

RESP; the strong need is to give the dot officers some extensive

IT; yes. Like now the service delivery, whether you think that there has been a need to either increase it or maybe to reduce it

RESP; to increase it

IT; okay, what support are available to help you adopt this decentralize service delivery we are talking in your facility?

RESP; like I’m saying we need training more knowledge about

IT; I’m talking of all the ones that are available, the support that are available

RESP; right now, there is none

IT; there is no support? There is no factor that is helping you? Are you not part of the WhatsApp group where they send result of any positive thing

RESP; I’m not. It is only the TBLS that is

IT; but you know about it, do you think it support that support your facility. To you now as an individual

RESP; it supports the facility in the sense that

IT; so, tell me about those support. yes, to the facility

RESP; we got the patient in time and they turn to for their treatment in time so that is how it help us to get the result in time

IT; which one now? I said what helps you to get the result in time? Is it the fact that the G helps alert comes to your TBLS

RESP; yes contrary

IT; alright. Is there any other thing?

RESP; no

IT; So, what kind of incentives are there to ensure the implementation of decentralize DRTB service is successful

RESP; the support is, those people like I said.

IT; I said incentives

RESP; incentives yes. They mostly are in need sometimes, if you don’t see them on time we call them, their complaint is that they don’t have money for transport and at times we that are service delivery, at times we need some money to buy airtime or data.

IT; okay but I’m talking about the decentralize DRTB service delivery. Are there incentives that have given to people so that the implementation will be successful? Like are you aware that transport, some form of transport fare is given to some patient?

RESP; that’s why I said if token is given to them, we will be able to come there will be no complain of I don’t have money for transport, I don’t have money to eat and then you understand what I’m saying? then like before they got admitted for about four months now they are now using only 2 weeks in admission and on getting to their house they may not have enough food to support that drug to be able to help them recover quickly so some take some time to recover

IT; okay how do you think your facilities setting or how you run your affairs at the culture of the facility that it affects the implementation of the decentralize DRTB services?

RESP; the facility setting help us to implement the service

IT; how?

RESP; it helps us because each one of us immediately we get the result so we play our part, we do our own. We started the patient and treatment and they get their treatment and they get recover.

IT; yes, I’m with you

RESP; yeah

IT; okay. Now what has been your motivation wanting to have and ensure the implementation of decentralize DRTB services is successful? What has motivated you?

RESP; what motivated me is those people that are spending on this program, one way or the other they are trying it momentarily and then it makes me to be able to render my own better to support the program so that it come successful. So, I appreciate them and I love to be part of the program to make the program successful.

IT; okay, how confident are you about been able to implement decentralize DRTB service regularly in your facility?

RESP; we don’t have any for them, we are confident

IT; what I mean is, are you confident that you are able to implement that

RESP; yes

IT; why are you confident?

RESP; because I know what to do and I’m able to do what I suppose to do by delivering the program

IT; okay how about your place, are you confident that you are able to implement the decentralize DRTB service delivery?

RESP; yes

IT; why?

RESP; because if I’m not around and any one of us that is around know what he or she supposed to do and they did it for the patient, the system continue successfully. We all know what to do.

IT; okay. So, let’s talk about the fitting, do you think that decentralize DRTB service delivery fit well or fits your existing work processes and practices? Does it fit the processes of your work here?

RESP; it does fit. Because it’s like grading because we have been practicing GS and when the VR comes in so we don’t have any much problem about finding out the service so we don’t have problem

IT; your practice here, DRTB is it well integrated into it, can you tell me about it?

RESP; it is well integrated because

IT; I mean decentralize DRTB service delivery, is it well integrated toward your

RESP; yes now

IT; in other word does it fit very well toward you’re doing your practice?

RESP; it fit very well because we don’t have problem given or during for the patient or giving them appointment, there are records are well kept.

IT; okay lastly, can you describe how decentralize DRTB services will replace or complement the current program or process? Do you think we should replace the current program or it should complement, the both of them should be side by side or we should just now concentrate on decentralize DRTB services.

RESP; the decentralized one that we are practicing is well okay

IT; so, are you saying it should now take place of the other one or they should combine together?

RESP; They should be combined

IT; why? Why do you think so?

RESP; because one helps another, it helps another.

IT; how?

RESP; you know everybody will get the result and they will know about it if it’s concentrated on some few hands so the few hands normally handle the system with the new system, people that are incorporated with the problem now, so they should work together so that to make the program successful.

IT; thank you very much sir.

RESP; yes sir.

**KII/A8**

| **Interviewer/facilitator/questions** | **Respondent/responses** |
| --- | --- |
| So having explained the purpose of the interview I think we can go straight on to have the interview right away, and I will start ma by asking the first question. | You are welcome sir. |
| So, we want to know what you think of TB Reach Wave 9 interventions to decentralize DRTB services in this State. What do you think of it? | Yes, is very good because some patients, the risk of patients coming from another place to come to the State is limit and is very easy for the patients even wherever they are, where they appoint to go so that it can be easily for them to go therefor their drug for their treatment instead of them traveling all over because of finance, because of the transport far. The little transport far they will be having at least they will be using it at least for upkeeping |
| Okay, are you referring to the fact that now treatment is at the community level? | Is at the community level yes |
| So, is easier for them to access treatment? | Yes exactly |
| Okay and again of cause for people like you in the area of laboratory scienceswho are involved in this programme is also easier for them, what do you think about, is it not easier for them to access or to do their investigations, what can you say about that one? | Yes, is easier for them to do their investigations wherever they are instead of them to be going too far place to do their investigations, in their communities wherever they are they will just get into that place and collect their sample there and do their investigations for them there. Then again, they can make it easily for them to collect their sampleto gather them wherever the community they are and collect their samples there so instead of them to be traveling all about and for the fact that they are not even well is very risk for them to be travelling all about |
| Is alright, so but looking at these interventions, what do you like most about it? | What I like most about it is that, mostly what we like about is for us to be collecting the sample from the patients then again to go to their GOPD whenever they have their samples you go to them and collect their samples then again for the text messages, they have been receiving from their |
| Okay when there is positive RIF resistance. Okay the message that you sent to patents as well as the TBLS | Yes sir |
| So, these are the things you like | Yes |
| Okay which one comes first now in all these things you have said? | I love for the fact that giving them message if there is any positive |
| Okay that message they get | Yes, is very-very important |
| Why? | Because if the patients see the messages, he will know that there is cause for alarm that he should come to the centre |
| Alright. Is there some things you like the least as in something you can say if you are asked to grade this thing now which one do you think will come last | The one to come last? Anywhere the one to come last is at least tracking the patients, tracking them. If you did not see them, if you send them message to them and you did not see them or send token to them to come around and you did not see them, there is means for you to track them to see where they are because they may not come |
| Okay you are saying that is important to track them | Yes, is very-very important |
| But it should come last | Yesit should come last |
| Is alright. Is okay | Because some patients may be funny, they will be running away so you need to chase them and bring them, track them wherever there are and bring them around. I think if you do the other things the necessary things and you did not see them you track them, that will be the last option |
| Okay that should be the last thing to be done | Yes |
| Okay, what influenced your practise of decentralized DRTB?  What influenced your practise of decentralized DRTB? | what influenced? |
| Yes, or affected your practise of it? Is it the love you have for patients, is it the fact that you are paid for any investigation you do as a professional? | Yes, the love for the patients is very much important though for the fact that we are paid for the services we still love our patients for them to at least get well so that they will not infect other people and they will not you know |
| So what actually influenced you is the love for your patients? | Yes exactly |
| But are there challenges that you have encountered doing this work? Have you encountered any challenge? | No, not really; no any challenge because the work is easier. No challenges. |
| Okay. Well let me now ask you to compare the decentralized DRTB services with the routine existing programme. What I mean by routine is that former way of doing itor usual way of doing it whereby the patients now come to the centre for investigation, they don’t get text, the only person that get text is the programme manager, they are not giving an form of support, they are not even counselled as a team by supervisors, all these things, all those components you know that is in DRTB is not there so can you compare this DRTB now that has all these components with the other one | Now, this one is very much okay. This one that we are doing now is very much okay than the other one because the other one some patients may not get information even some patients will not even come |
| There will be lost to follow up; is that what you are saying? | Yesso but this one because of the follow up then they are not going far, they are still around so they can easily come to where you asked them to come and they can be attend to. |
| Okay, so are there other advantages that you can think of that this one has over the other one? | Yes, the advantage I can think of is that for the fact that they are monitored, for the fact that they are tracking the patients because they are very stubborn, you know them very well. that is the most advantages |
| Yes, before now they use to track but the is an improved tracking using CBOs | Then again for the fact that we wave supervisors |
| State liaison officer, is that what you mean? | State liaison officer yes, that is |
| Is up and doing | Yes, he is up and doing and then again he calls to us we pest the result on the this thing for them to see, so it makes it easier and |
| Is it not there in the other one? | No is not there in the other one |
| Is okay | This one involves media something, so it make it easy for us to transfer the result to them even before they have the hard copy but then we use to go after we did the test we now take the result to them the hard copy but now even before they see the hard copy they have started treating the patients |
| So that means it actually reduces the time of making diagnoses and the time of enrolment is shorter now | Yes sir |
| So, they are the advantages? | Exactly sir |
| Thank you very much. Let’s also look at what changes you needed to make in your facility in other to be able to implement this programme | Yes, the changes that we have, before we use to run everything together but now we have a special one they label as a DRTB patients so we have special wrap for them so that is one of the arrangement. Then we have a special register for them |
| Okay, these are things you change, you acquired in other to implement this programme | Yes sir. So we have special register for them, a special wrap for them to know that this our TB patients with them, whenever they are called to collect their sample we make it fast or whenever they are around we attend to them so that they won’t waste time to infect other patients around |
| Alright, now looking at all the components, is there any one of them that you think should be changed | No |
| Or be altered as the case may be | No for me is well okay and well but the only I said should be the last is tracking whenever others are and off cause you didn’t see the patients |
| So everything is okay as they are | Yes Sir |
| Okay, and how complicated is this program? | Is not all that complicated, is not all that complicated because is very easy for us to go there whenever they have their OPD, we go there and collect their sample and run it fast and give them, so is not that complicated. Not that we will be going from one centre to another but whenever they have their OPD whenever they are together we just go there and collect their sample and is very-very easier for us to do that |
| Alright, does this programme I mean decentralized DRTB, does it serve the need or meet the need of your patients | Yes exactly sir. It help, it meets the need of our patients. Sometimes the patients you know the transport they will be given to them to come and take their treatment is part of it |
| Okay | Then for them to even take the treatment without any financial support from themselves is also part it |
| Medical bill? | Yes |
| Okay, does it meet their psychological needs? | Yes |
| The counselling? | The counselling from other survivors to them whenever they see them that they survived, they will have faith that since that person can survive it I too I can survive it. So that one also help them too |
| Okay can we say that in your opinion; there has been a great need or a strong need to increase or reduce decentralized DRTB services? | Though let me not say you should reduce it but you can increase it. Don’t reduce it |
| Why do you think we should increase it? | Yes, you will increase it because is a help, is a great help for other patients who are there. For other patients too if you can increase it, it will be very helpful because there are some other patients dying outside there and don’t know what is going on but once they know all these things they will be able to come so you can increase treatment for us so that at least we can save many lives |
| Is alright, so now let’s talk about the support that you have to be able to adopt this program in your facility, what support are available for you that you can think of? | The support available for us is that for the fact the they have their OPD to call us to come and collect the sample is a support because they make it easier for us to be going as in to be going from one place to another to look for them they make it easier for us just to go there, they gather them for us and we just collect their sample like that and release their result immediately instead of them because sometimes you cannot make it to be going one by one to look for them in a day and their result will not be coming out at once like that but once are gathered and you collect their sample their result will come out once |
| Was the WhatsApp platform of any support to you, the WhatsApp platform that you have? | Any platform? |
| That WhatsApp? | Yes, we have the platform that we paste the result. So, we collect the sample and we run the result even before we go with the hard copy we send result to the State liaison officers so that they will now get back to the patients, they will start treatment with the patient before we bring the hard copy |
| So you are working with the state liaison officer is it of any support from what you are doing? | Is a support because sometimes that result issue he stands as an intermediately between us and the patients. Is a very supportive programme for us |
| Okay, thank you very much. Now let’s look at the incentives that are available to help ensure that the programme is well implemented, what incentives can you think of? | Okay, like transportation to the patients, because some patients if ask them to come they say I don’t have transportation. We don’t have transport far but once they gave them the transport they will come |
| They will be encouraged | Yes, to encourage them to come around and again the communication, they call them, there is communication allowances |
| for the CBOs? | Yes, for the CBOs to call them to come around and there is transportation to the survivors to come and counsel them in their centre |
| Is okay, thank you very much. So, let’s now look at how you think that your facility’s culture or setting affects the implementation of the decentralized DRTB in your facility |  |
| Setting or culture, the way you do your things | It don’t really affects us like that but it really |
| It helped you to improve | Yes |
| How | We have special place to collect the sample of the TB patients but before we don’t have something like that but now |
| Then when we were discussing the. interview you said something about time management that you people are very conscious of time here that it is part of your culture here? | Yes, we are conscious of time |
| You talked about time; I think you also talked about infection, prevention and control? | Yes |
| You mentioned something like that? | Exactly because if patient is around we make sure that we collect their sample very fast and not delay them because of other patients. We collect their sample and ask them to go and we transmit the result to the |
| So why will you collect their sample immediately and ask them to go, why? | Not to infect other patients and again not to delay them |
| Okay, now what has been your motivation in implementation of this program? | Is the love of the patients. We love our patients too much so don’t want them to die, we don’t want them to infect other people so that motives us. The love of patients actually though the work service and the money is there but the love of the patients is the priority |
| So how confident are you about been able to implement the decentralized DRTB services regularly in your facility? | Yes, we have the confidence, we attend to them. Anywhere we have the confidence to manage the patients |
| I am asking; why are you confident? | Is our job to collect the sample and run it, so we have been doing it before so for the TB patients is part of our job to run the test for them too as we are running it regularly |
| Which is what you love doing right? | Yes exactly. |
| Continue? | So, as we love doing it before, our job is to collect sample and run the test so for them to even be around come around is our confidence to collect the sample and run it for them so that they can collect the result very fast |
| Okay let’s talk about the confidence of your colleagues, are you confidence also that they will be able to implement this program regularly in your facility? | No, not really like that. Not all of them can handle the sample of the patients like that. We don’t really have confidence in other workers so few of us are the one handling the samples |
| Okay because they are not trained scientists? | Yes, because they are not scientist and they have not been trained for it so we that have been trained for it are the one that are handling it |
| Okay, now let’s think about how well you think that this programme that is decentralized DRTB services that it fits into your existing work processes and practises. The things you do, your lab investigations now this program does it fit well into it? | Exactly, it fits well because it is our job to run the test before and this patientsthey are to collect their sample and run their test so it fits in exactly and is what we are running now is the sample we are collecting from them so it really fit in sir |
| So is not like is time consuming or challenging? | No, no. |
| It aligns well | Yes exactly |
| Because is your practise? | Yes, is what we practise, what we are used to it so and it really fits in |
| Okay, I want to ask you whether you think that this decentralized DRTB services delivery that it should replace the routine one or? | No, no-no, this one is very much okay. This one we are doing now is very-very much okay |
| So, we should leave the other one and be doing this one or we should be doing them together? | No leave the other one and be doing this one. This one is very much okay |
| Okay you said we should leave it? | Yes Sir |
| because is better? | Yes this one is better |
| Okay, can you tell us how you think that this service delivery, the decentralized DRTB service delivery how we can make sure it is sustained then also scaled up to other places because I know there are some other local Governmentthat don’t have it and is not in all the states of the federation? | Yes |
| So what can we do? | We will just want you people to help us to spread to other states and local governments so that’s what I said it the other time they are some people dying somewhere that they need this programme so if you can do something about it, we need it because we don’t want our people to die |
| Is okay. Alright thank you very much | You are welcome sir. |

**KII/A9** 2

IT; this interview is taking place in XX State with XX and it is going to be conducted by doctor X

The focus of the interview is going to be on the interventions to decentralized DRTB services, TB reaches Wave 9 interventions. That is what we are going to be talking about today.

IT: I want to ask you ma, what do you think about this DRTB intervention services?

RESP: A well TB reach wave 9 intervention is a good DRTB services is a good one. It is one that has helped the improvement in the enrolment of the DRTB patients in XX state, so the intervention is quite a good one. We expect that it will continue, since we are seeing result of what has been done so far.

IT; so, what do you like most about this intervention?

RESP; Well, it is the availability of the services near the patients, such that they do not have to travel far to access some services before they start treatment.

IT; and what do you like least?

RESP; the program is okay so let me not say there is something that I really do not like about it.

IT; what has affected your practice of these decentralized services in your state?

RESP; affected our practice? Like what has changed in what we have been doing before?

IT; yes, what I mean is now the implementation of this service, there must be things that has either influenced.

RESP; that is what I’m saying, you see for any services that is not concentrated at a place, when it is decentralized what you are saying is that you make it available for the people at a reasonable time and in a less expensive way.

IT; so, what has helped you most?

RESP; what has helped us most is that some of our services has become available real time to the patients instead of them waiting, it has reduced travel time, it has made the patients to be willing to access the services since it is now nearly at their door step. Instead of saying you will have to move from somewhere and come to this place.

I remember before, though even before TB reached here, we have been trying to be decentralized but when they came home, we were able to do more decentralization. You understand what I’m saying? We were able to do more decentralization. Even when we still do not have enough services, they were still able to help to provide transport services to people to make it available to them more, so that the patient is willing to access these services because the cost is no longer borne by the patient alone, it is now being borne by the program. So, it makes them willing to want to go to access the services the services that are being provide. So, one way or the other, it has influenced the success of treatment of patients.

IT; Are there challenges you have encounter?

RESP; with the decentralization?

IT; Yes

RESP; Yeah. We have challenges and some of those challenges are when we do not have, you know in some of our rural areas where we do not have comprehensive services, it makes it difficult to want to decentralize to such areas because the services provided. We have laboratories that cannot provide comprehensive services which have become some of the challenges. So, we are not able to decentralize services in to those places especially in the rural area, we still need to bring them to town but one way or the other we have had support to provide transport allowance where the routine transport allowance is not enough, decentralized services has still backed us up to support them, to bring those people that are affected to where they

IT; now, how does decentralized get resources compared to the routine problem

RESP; well, in any program where there is no decentralization you have more access, people are able to access the services better than when it is concentrated in one place. For example, when we have only, I remembered when we first of all started when there was only where we can access based line services, it was only in Ibadan. Patients will have to travel all over the state to come and access the services but as time went on, they were able to decentralize to some other local government. When TB reached here, we were also able to decentralized more to some other areas where patients can be able to access the services. So, when we compare the decentralized services to the routine one, you will see that it offers the patient access to treatment on time, access to services that can enable them to access treatment more than the time passed and it also help us to recruit more patients. You understand? Patients don’t have to complain, I kept traveling I don’t have money, I don’t have this. So, they do not have any excuse to give more than to access the services because it has been brought nearly to their doorstep.it helps more than when it was not been decentralized

IT; at the clinic lab, can you give comparison?

RESP; it is the same thing. Services whether at the clinic services are mostly offered at the clinic then at the local government. You understand? When a patient is diagnosed, we first all go and does the base-line test at the clinic, then the facilities at the TBNS get the result and that patient is able to be place on treatment on time. They don’t have to wait for us to send the results from Ibadan to them because they have access to result real time.

IT; okay, thank you very much. I know you have mentioned some of the questions I’m going to ask you now but just to encapsulate it. I will want you to tell us the advantages of this over the routine program.

RESP; the main advantage, the very main advantage is that the patient starts the treatment on time and that is what we’re looking for, that we put our patient on treatment within a specific amount of time when they are diagnosed, no excuse, no reason why the patient is not put on treatment and that is what we’re looking for. Within 14 days, patient has been diagnosed and that patient is put on treatment and that is the main advantage that we have in there. We do not have gaps in our enrolment. Patients are not lost to follow up. You know sometimes when a patient says, it is because I don’t have money to come for services, it is because I don’t have money to come for base-line, it’s because I don’t have money to come for this, before you know it that patient can be lost. If we are not able to recruit the patient for treatment, before you know it the patient can die and we are not able to put the patient on treatment, so we have had lesser gap, we have had reduced time in enrolment. The gap in enrolment has been able to reduce and then our patients have access to treatment on time.

IT; is there comparison with the routine programs? Do you think decentralization has it disadvantages or its disadvantages?

RESP; yes. Like I have said, comparing the routine program, Number 1, patients don’t have to travel for a long distance before they access services

IT; so, what are the disadvantages? The disadvantages of this intervention.

RESP; let me say there is no disadvantages so much. The advantages far outweigh whatever the disadvantages we think there is. Well, the only one I can think of is maybe compilation of funds, before I only have one lab to collect receipt from and pay, now I have to collect from different laboratories, bring your receipt, bring your receipt, some will prepare on time some will not prepare on time but you have to wait for them. And then at the national will be asking you, we want to pay we want to pay. You understand? That is the only disadvantages. When you are dealing with one person, you know you are dealing with one person but when you are dealing with many people now, you know it has become, not everyone will respond to you on time. That is the only thing I can say is there that, their time of response for payment. That is the only disadvantages that I want to see that is there.

IT; it is alright. So umm, were there changes you made in order to faithfully implement these decentralized services?

RESP; yeah. There are changes and there are still changes.

IT; in your perspective

RESP; There are things that needed to be put on ground so that the patient can be able to get what they want. You know reading of results, transportation of result and many other things like that. So, we have to be proactive. You understand? Even everybody needed to be proactive to be on their toes. Okay we are dealing with a DRTB patient, it is different from any other person, this person needs to be on treatment on time and they cannot be delayed in anything that concerns them. So once a patient comes in, we understand that this patient is not just like any ordinary patient that has just come. It is especially a patient that needed to be deal with on time. So, people cannot be laxity in the attention given to such patient. So, everybody needs to up and doing as far as working and provision of services is concern. So, there were changes, in the attitude there needed to be trainings, there needed to be more counseling of people in handling issues and in handling matters. So are basically the changes that needed to be made.

IT; considering the components of this intervention, are there things that need to be odd out?

RESP; well, you see as we were going, we were improving and doing well. But when something comes in, not like that there should be any alteration but for any service that is being provided, you look at how it can improve, what can we do to make it better. So as we are going along the way, we were improving and okay what can we do to make these things better? Like I was talking about the support for provision of transportation, for people who the decentralized services could not even reach their places, you understand? Now the basic transport allowance is 3500 that is provided for everybody but there were people that we got to know that the 3500 was not enough for them. Even when you say, okay we have transport for you please come and the person will ask how much and I will say 3500, after some calculations we discover that the 3500 cannot take him and bring him back to his place. They will not tell you, they will continue to post you, so one of the things we did was to like at, okay what is the cost of transportation of bringing these people to this place and then the program was able to support, push up what the national has provided, okay I said how much is it costing them. Give, you understand?

IT; you mean there has been an improvement in what is been deposed to them?

RESP; no. the program supported, the wave 9 program supported in what is given, that is what I’m saying. To be able to bring more people to, you understand? We were able to be decentralized but you cannot decentralize towards the local government.

IT; Are you saying this aspect should not be altered right?

RESP; it should not be altered; it should be improved. You understand? That is what I’m saying because I’m not sure it was actually part of the project but because they were committed to making it succeed, they were doing it.

IT; What about the other components?

RESP; the other components are good, they are okay.

IT; now, how complicated is this decentralized service?

RESP; it is not really complicated; it only needs a good coordination system. It is not complicated; it is well coordinated well unlike the routine programme.

and it needs people who understand coordination very well to be able to put it in place. Just like I have said, you will need to interact with many more facilities, many more clinic as against interacting with just one, you will need to make sure that things are running well, you will need to be on your toes to know that okay something is not working well here and okay where can I direct my patient to work. Machines break down, things don’t work well and then when you say please this lab, provide these services for this person and the person will be saying okay we have heard its everything on that. You will have to know that the services being provided should not take more than 24 hours, after 24 hours that you asked, have you provided the services for the patient? Yes, we are still on it, why are you still on it? So, it needs a firm coordination, needs one to be proactive, it’s a good one, you understand because you needed to distribute reagents, you need to distribute drugs, you need to distribute drugs to different facilities and before DRTB drugs are not centralized, they are not decentralized, but they are not yet still decentralized but one way or the other we now passed to the local government. We are able to distribute the drugs and patients can access the drugs from their different local government that it doesn’t have to take you more than one transport to go there and access your drugs.

IT; So, if I got you correctly, it seems the coordination is not yet as you would have liked it.

RESP; It is. That’s what I’m saying, you were asking how it was different from the routine one and what are the things that makes it to be different and what can make it to work better so that’s what I’m saying that the things that the decentralization involves, those are the things that it involves.

IT; you are talking about the complexities.

RESP; so, it needs you to be on your toes

IT; so, the coordination is alright?

RESP; The coordination is alright but anyone that is there needs to know what it entails and then be ready to work as it is, as you can see in Oyo state we are doing fine. It’s a project that has really helped us one way or the other like I was sharing with somebody that myself and another partner we decided to sit down and look at the…. before the project came what was our dab, when the project came what was our dab and we had like 25% increase in the enrollment dab, you understand?

IT; increase?

RESP; increase yes. more than what we were enrolling before. We saw that between just a year one quarter to the other we’ve been able to increase our enrollment from what it was by 25%, so these are things that it has helped us to achieve, all the program put together as wave 9, you understand? Whether the putting up of one person to help, be working together, you understand? Everything that is what we have achieved.

IT; it’s okay, thank you very much. I know also in one way or the other you might have mentioned these questions about the needs of the patients but I want us to just look at it now.

RESP; the needs of the patient is enormous

IT; what I mean is how does the decentralized service meets the needs of your patient?

RESP; well number one like i have said it has reduced their transport, it has reduced complication in accessing services, you understand? It has made the services available to them at minimum time, at the minimum time that it is needed it has made the services available to them. Formally somebody will have to travel from probably Oyo to come and do even follow up, monthly follow up but now that we have decentralized our services to a lab in Oyo he doesn’t need to travel, just walk there take okada of 100 naira walk to the lab and submit your test and that is all, so it has increase access to services, it has reduced the amount of time that the patient spend to access services, it has reduced the amount of money that the patient spend to access services ,so it has increased the power of the patient to access their services and to own their own treatment, it has even made things available to them, it has made you know if you want to see the doctor, you will have to travel down but now you don’t need to travel down because a doctor has been made available in our own PD clinics that they can go and see either on a monthly bases or as the need arises. So decentralized there has to be in the sense that has made the services available to the patient at a minimum cost and at no time wasted.

IT; okay, thank you very much. So, the next question is, has there been a strong need to increase or reduce decentralized DRTB services?

RESP; strong needs?

IT; yes, in your facilities

RESP; yes, there is strong needs to increase decentralized services if we have the opportunity to do so, you know like have I told you there are still places where we are not able to decentralized to. So, we still need to go there, though there are some other impediments that are causing it. Number one is the money allocated for the services, the money allocated for the services, it has a fixed price which in some places could be far higher than the fixed price and which we may or may not be able to cope with so patients still have to come out of those places, because of the amount of money that those services are rendered in those places which is far higher than what we have and because some facilities they would not want to change, this is the amount of money and that is what we are going to take so those are the things that have actually caused us not to increase to as money places as we want to.

Another thing is the unavailability of those services in those places, yes, we get clinics but they will not offer the services that we want and they are not ready because the number of people we get there are not many so for them to incur the expenses of the services to them is not profitable so those are the things that are impediment are on their way making us not to be able to increase the places where we will be able to increase. If we have more funds to pay for those services then we will be able to increase to those places.

IT; it’s okay. So what supports are available to help you adopt this service delivery in your facility?

RESP; support?

IT; yes. Support those things you have found as supporting maybe that have enabled you to adopt

RESP; we have a supporting firm, there is human resource, you understand? We have human resource but they sometimes we do not have the other resources that we actually need, like if we need to decentralized drugs now totally, the DRTB drug need EC most of our facilities could not have it, some don’t even have far, you understand? So, it makes it difficult to actually decentralize the drugs and store them for a period of time so you cannot give, we don’t give more than one month supply you understand? The other time I was telling you the complications of that and you needed to be on your toes and look at which patient need drug this month you have to send, you understand? So those are some of the challenges that we could have as far as some of the decentralized services are involved.

IT; we were talking about support, remember there was a time we mentioned something like capacity building

RESP; yes. We have had such support, we have built capacity, we have had trainings, and like I have told you we have human resource that is basically the greatest support that we had. If there are no human resources, there is nothing anybody can do but the human resources we have there, capacity has been built, they have been trained to counsel, to provide services in one way or the other, to people and what have you. So those are the supports the program has provided.

IT; now, let’s talk about incentives.

RESP; okay.

IT; so, are there incentives to ensure that the decentralization works?

RESP; yeah, there are transport incentives to the patient, sometimes it is enough sometimes it’s not enough, like I said where it is not enough the program has been able to provide support for patient that the transport allowance is not enough to bring them to where they can obtain the services and then there have also been incentives for health workers for counseling services provided for the patient, you understand? Time spends, then there have been incentives for TB survivors, people who had had TB, they have taken the treatment and they have been healed, in fact they are one of our greatest supports that we have. They come and see other patients who are about to start treatment, they share their experience, they tell them what they have passed through, they encouraged them let them know that this thing will not kill you and that you will be healed, we have taken the drugs, we are okay we are here you too you can take the drug so it’s one of the support that has actually, it’s one of the incentives I want to say, you ,may look it like an incentives but it is an incentives to patients because the patient sees, hears, like the Yoruba use to say “has it ever happened to you before?” and they are able to tell the person “look it has happened to me before and here I am” so it is a motivation for the patient to also receive treatment so it’s a great support that the program is provided for decentralization.

IT; okay how do you think your facilities culture or settings affect the implementation of decentralized DRTB services?

RESP; I have said it, some of the resources do not support decentralized services, we do not have resources, I’m not talking about human I’m talking about, what do you call it the resources, like AC for drugs do you understand? If I’m going to keep drug in this place, I needed to provide AC so you can’t decentralized drugs to those places, do you understand? so those are some of the things that may not support the decentralization as we want it to be, that a local government officer takes control total control off everything that has to do with DRTB in that place, so they cannot really take the total control as we want them to take total control, they cannot store samples because there no fridges and there is no cold chain system okay if these samples are not able to go to the laboratory today, can we take it and keep it here. Those are some of the things, there are infrastructures that we do not have the infrastructures that are needed sometimes with some of these facilities to actually support decentralization as it wants to be.

IT; yes. And apart from that, you know you can have culture in some working places maybe among the staff how things are done here, how things are not done these are some of the things that can also

RESP; you know we have had trainings; we have had capacity building and we’ve been able to say what DRTB is, the facility staffs understand what they are in and physically those who are going to be taking care of the DRTB patient are people that have been trained on TB and are working on the program so our culture as people working on the program is how to actually take care of things so

IT; so, they kind of improve

RESP; so, there is an improvement, it’s going to be different from a health worker who has never been trained on the hanging of TB you understand? Such person may look at the patient no I’m sorry I don’t want to carry, you know there could be stigmatization but all those things we have been trained, capacities have been built and we have what it takes as health workers who are working on this program to provide services so our culture is that of people who understands TB and who can handle the treatment who knows what it entails who knows how to deal with patients so we have an acceptable culture as far as we are concern.

IT; so, I want to ask you a reasonable question, what motivate you to want to ensure that the implementation of these services deliberate is successful?

RESP; the care of people, the willingness to see somebody who is ill to become well again, the joy in seeing that you have treated somebody and the person cause you more than mummy don’t you know me again and the person who came to your clinic that day ok I’ve grown fat that’s why you don’t know me and you look at the person and say wow you have changed so the joy you see that you have been able to preserve somebody’s else’s life, you understand? so that these are things that motivate you and keep you going without getting discourage that you’re able to help others to live well and you’re able to provide health for people who have given up hope who think they are going to die, who think there is no life any longer and you have been of services to see that somebody else’s life is preserved along the way.

IT; apart from that just talk about services, just satisfactions.

RESP; yeah

IT; alright, so how confident are you about been able to implement this service telegram?

RESP; I’m confident.

IT; regularly in your facility

RESP; yes, we are confident, we have everything that we need, we have been trained, our capacity has been built, we’ve been working on this program for some times now so we know what to expect, we know what to deliver. We are confident of ourselves why? Not ourselves with the lord of our power, God gives all of us power but because we have received training our capacity has been built to handle issues, we’ve been doing it for sometimes we have learnt along the way we’re still learning and so we have this confident that yes, we can deliver the services that are provided for us to deliver.

IT; okay. How does decentralized DRTB service delivery fit with your existing work processes and practices? You may have answered this question in the cause of our discussion so what you will just do is to maybe give a brief summary of it.

RESP; how well it fit into….

IT; into your organization

RESP; when things change, it’s only a dead person that does not change, anything that has life will continue to change and changes bring improvement. If you don’t change you can’t improve and you cannot continue to want to do things the same way all the time and expect that there will be success all the time. Things are changing and if we need to get improvement, if we need to be successful in the treatment of our patient, if we want to see them live, if we want to record more success we need to change. Decentralized services have brought a lot of changes you understand? In a way we do things and we are changing with it because if we don’t change because we are seeing result, we are also adapting to go in this way. If we are not seeing result, we will be adamant and say what has changed? Nothing, why don’t we go back to what we were doing before. But because we are seeing changes, we are seeing result, we are also changing with it, we are adapting, we are changing what we use to do before to what is acceptable as far as the decentralized DRTB services is concern and because we are seeing result, we are flowing with it.

IT; so, in a nutshell, you can say it fit well into your organization

RESP; yeah. It fit well into what we are doing because our main aim, our objective is to see that people get treated and aware so whatever is going to give in to help us to achieve that objective then we will accept it, we will flow with it, we will fix it into our process and our plans and see that it works because if it’s not what we are aiming at then why are we doing it and if there is something that is better that we can do to still achieve our objectives in a far better way and gives us a better result and reduced death, reduced loss to follow up then we are up for it and that is what decentralized DRTB services has provided it has reduced death, reduced the number of patient that are loss to follow up, it has reduced the enrollment gap, it has reduced the amount of time it takes for a patient to start treatment on time which also has led to reduction in death so that is what we are looking for.

IT; so, talking about these processes, how have you been able to reflect decentralization now in your day-to-day work? How have you been able to capture those things needed in your daily work schedule and stuff like that?

RESP; yeah, everything is included in our daily work schedule. You see like have said when you are in a program, your routine begins to change, you begin to adapt, you begin to change the ways you’ve been doing things as far as you understand what I’m saying? so it’s all embedded in what we have been doing before you understand? Like when you’re talking about how receiving of alert you understand what I’m saying?

IT; yes

RESP; and all those things like that. Even if somebody receives alert and it’s not you, the consciousness that this is a DRTB patient there is a WhatsApp group that we have created and they bring it there. These things were not done before so the person sent it to the group and say look, I received this alert from this patient who is an owner and everybody sees it and say okay this patient belongs to this person so it helps us to identify where a patient comes on time and the patient is quickly tracked and placed on treatment. Those things were not done before but because our aim now is to decentralize services. Everybody is now on their toes and having understood what we are dealing with that this is a human being who can die if we don’t act on time.

IT; good. Now that you mentioned it, the WhatsApp platform does it have any kind of support to this implementation?

RESP; yes. It serves where? When a patient is diagnosed in one lab, that lab person put it on the platform and said yes, a DRTB patient have been diagnose for me and then we pick it, we see the name of the patient, we see the local government where the patient come from then you call the local government, you have a patient and so on, she will say I’ve seen it too track and up she goes to track the patient on time. Everything is done at real time instead of before when nobody sees anything, you first of all have to go to one platform, one back end to go and look for it. Okay we are a week because I may not go to that back end for a week but now everything is done on time as at when due so those are the advantages that we have from this and we were not doing it before but when it came we adapted and everybody took it up and started doing it so those are what I’m saying that it’s not a process that is out per say we are making ourselves fit into it and making it to work.

IT; alright thank you very much.

RESP; you’re welcome.

IT; do you think that this decentralize service delivery should be replace or complement the current program?

RESP; we are working together, you understand? A program is not really out per say there is always improvement so the decentralize service has not come to replace.

IT; okay

RESP; it hasn’t come to replace. All it had come to do is to improve what was on ground. We were doing it like this before, now let’s do it this way.

IT; what’s your own opinion?

RESP; if it can be improved better it’s good. You know I’ve told you some of the shortcomings what we build to make it work better so if those things are able to be provided for, it can make it better so it’s a program that it’s on the way and if it works more, it should stay if those infrastructures are able to be provided you understand? If not, we will still be working half you understand? We will stay with some of our routine program before while we work with the decentralization you understand what I’m saying? And see that okay the two of them are working if we are this one will still remain like this this one will go like this. We are available to decentralize services as far as it is concern, we have been able to decentralize OPD that is out patient department the patient comes to see the doctor in a monthly basis, it’s now in some local government but we have 33 local governments, we have it only in 5 places so we clustered you understand what I’m saying?

IT; yes.

RESP; so, if we find a doctor that can be trained and he is willing to work with us on DRTB we train them in some other places too you understand? If we have a facility where we can do AC and put drugs and employ somebody to work there, then we are off, we can do that too. You know that is still decentralization?

IT; yeah, but I’m saying if we were able to provide all the infrastructures as we have said made up all the challenges

RESP; yes, that’s what I’m saying. That when all those things are provided, it is the way to go.

IT; okay it can possibly reflect?

RESP; yes. And I’m saying it’s not replacing, we are only improving and getting better. The program stays there but what we are doing is that we were doing it like this before now we are doing it this way because its better it produces better results you understand?

IT; yes

RESP; so, we are only expanding our host so that what I’m saying is not wasted we are only expanding our host and see how we can improve the program better every day.

IT; thank you very much. I appreciate your time and your commitment

RESP; thank you very much sir

IT; you’re welcome

**KII/A10**

| **Interviewer/facilitator/questions** | **Respondent/responses** |
| --- | --- |
| Good day ma | Good day, you're welcome |
| So, the focus of this interview will be centered on TB reach wave 9 intervention/Decentralized DRTB services as well as the challenges and any other thing that would be imagined as we discuss.  So without wasting much of your time, can we know what you think about these interventions? | Ok thank you very much Dr. XX, this intervention actually; the decentralization is meant to help the program and it contributed positively over time. For example was it not last year that the project started and up to date it has helped us to track our patients as long as they are diagnosed even to the point of care. So it has been a positive contribution to the project. |
| is there anything you like most about the intervention? | Yes, the prompt diagnosis of receiving notification. You know there was this WhatsApp group that was created by that DRTB services and with the involvement of our laboratory personnel immediately they diagnose a patient, the DRTB they snap, they send details and they go out and if you're online you will see that there is a patient at so-so facility name, you contact the CPNS in case of the person is not online, the CPNS or doctor in charge of that Local government. In case he or she is not online, Mrs. So, so, so, check this. I've sent this to your WhatsApp, this and this, and from there tracking starts. OK, ma we have seen her ma, she said she's coming today, she said she's coming tomorrow and immediately the laboratory personnel will get ready and we have fantastic laboratory personnel. They will just say so to the person whose baseline is this and sometimes they will call us asking if we have a patient, is it your patient whose baseline is due except a few times maybe the baseline failed. What I like most about this program is the notification aspect of it. |
| Is there anything you like least? | Least, no, not exactly because there is a transportation fair for patients who go for baseline, the DRTB survival counseling, so everything is fine. |
| So, what has affected or influenced the decentralized DRTB services practice? | Yes, like I said earlier it influenced it to keep track of the patients. You can even have access to your patient folder, ok so, so TBLS, are you not getting him through, ok let me try number from my own end to tell them it is so, so TBLS. So, it has helped a lot.  It's not as if we didn't do tracking before, but because we are meant to since this intervention is already on ground, you can't say that there has been a notification since 2 weeks ago and you didn't pay attention to it. And also, I just mention, with the help of the VLO, the VLOs are doing a very fantastic job. In fact, imagine the VLO calling, myself calling, even the TBLS will be like what is it why is everybody just disturbing me? Ok, we are on our toes, we've sent the contact details go and look for them |
| Are there any other challenges? | Yes, with parents, sometimes when you call the TBLS and they call the officers and they say I've contacted a patient, he said he is not coming. Well, they will go for the baseline. I thought of baseline and I found out they have a challenge now, there is a patient from baseline care, baseline investigation, and we saw an overhead. Though we promised them already that the treatment is community-based, they don't come to the facility unless it is from us, there’s nothing that said ok I was ready to go and collect my drugs the decision was difficult.  Even after the doctor's review, the advice was like, to go to the principal at first to manage it at first. So right now patient is like, you said I will be receiving my drug at the facility in the community but now you're telling me I'm going on admission, with that issue of pestering that it was because of the result that you did that was what formed the whole thing and you know that some of them are not learned. You know illiteracy with being poor; everything is all joined together so the background won't be fair enough.  But most of the time what I do is that I invite people from outside Ibadan, I will call them, calling from the state ministry of health, office of the governor, that kind of thing and they will be like ok ma, yes ma, so that's it. |
| So how do the decentralized services compare with the routine in your office? | How is it right? |
| Yes, the decentralized DRTB that we are talking about, how do you think it compares with the centralized one? | Ok, centralized, we don't really do centralized actually in a way that is already decentralized. It's not as if there is a place you must go to before you access treatment. So, I can say that we've been practicing decentralization even before and not as if we are just doing it now. So, the thing is that decentralization is better because patients have access to treatment, you don't have to travel far to get treated or go for baseline investigation except there are other issues. |
| Are there some disadvantages of decentralized services? | Well, disadvantages, not really but from the example I made before about my patient, we've already told him that he will be receiving his treatment at the community level and now we are changing it. The only thing is that probably there might be a clause that in case after your baseline investigation if the result comes out it depends when you will go to treatment centers. So that's why I say little disadvantage and I must put that fact for someone that has a pediatrics issue, someone that has an OPC issue, and because of that they get treatment at the community level. |
| Are there changes you met at the decentralized services that you needed to make in your facility? | Yes, in terms of changes needed to be made, now most of our CPNS and doctors are more responsible to their patients. When notification pops out I call, VLO calls that change of behavioral pattern of not handling DRTB as if it's a normal DSTB. And according to the guidelines that a patient must be tracked for treatment within 10 days. You know that tracking system, so in a way it has helped us to change.  I don't know if I've answered the question |
| Yes, attitudinal, behavioral kind of thing | Yes, it is not as if all those things are not in place, but now that we want prompt treatment within 10 days of diagnosis. So attitude has changed, behavior has changed, even now that they have already finished their project, it is as if the effect is still been felt, that when you see a DRTB patient, the person has already gone for a treatment, even sometimes when we don't get the alert. So, it has changed the behavior of some persons like that |
| Ok, now you remember the components of this intervention are there anyone that has to be changed or not? | I don't think anyone has to be changed because if you check all at all the interventions, they are more advantages to the DRTB treatment care. So, there is nothing to change, the components are fine, and to add anything, for now no, I didn’t think, at least laboratories are fine, they are tracking our patients.  And to be improved in, let’s talk about the improvements. To be improved in accessing the transportation for the baseline, for our patients to go for baseline. In some places where the laboratories are far, you know we are trying to decentralized but we still have some challenges in some areas where the laboratory is not agreeing with the fact that the price they are giving them for their test so that forced us I think if we are in a zone now where we have DRTB patients we try to encourage them to come for treatment, why, because the laboratories around that place are not agreeing with the finding or amount for the baseline tests. So instead of going to do your baseline you go to the lab dozer and go back again before going to the treatment center. So like I said that they are to increase the fund and that amount needs to be reviewed for the laboratories especially in some areas. You know we have 33 Local Government and not all local government labs that have been without plan so that affected the laboratories to join us. |
| Do you think that decentralized services are complicated? | It's not complicated |
| How; in what place? | Fine, thank you. First of all, we want our patient get cured that's the aim. So out of the need and care if the person is sick, we are providing a means that is free and accessible. You can't tell Me it's not meeting their needs, it will meet their needs as well. We are talking about all that cares maybe financially or support because sometimes the ones that are on this care the majority are poor, not all came from a wealthy or rich background, we have some that are well to do and some that don't have at all. So, some of them even to come for a baseline investigation is a problem. |
| So, in terms of the objective of the interventions, like reducing or bringing treatment to their doorstep and bringing about a positive outcome and then their medical needs due to their condition. | Yeah, that’s why I said it will meet their medical needs. |
| It's alright. So now, has there been a strong need to increase or reduce the decentralized DRTB services in your facility, do you have that need to increase as the case maybe? | Yes, like currently we are decentralized but it's still in the pilot phase, like we need to increase the level at which we are decentralizing. Maybe create more laboratories that we could be involved in, more OPD sites that are what we need because you never can tell where a patient will come from. There are some local governments that don't have any patients from that place and we have 33 local governments because if tomorrow a patient comes from a place where it's very-very far, there is no way. So, there's a need to improve more in decentralization based on we are waiting to an access. |
| Is there any other reason why you think you may have to increase these Decentralized DRTB services? | Well, other reasons include maybe staff capacity, human resources. Human resources too is a big factor in decentralization because when you are telling us there is need for this decentralization and no available hands, well skilled hands to handle DRTB care. That's the need in terms of human resources |
| Ok, but what supports is available to help you to adopt the decentralized DRTB service in your facility? | Supports that are available? |
| Yes, to help you adopt this service delivery that you can look at things that will enable you and how you will adopt it | Well, no 1 is a good population of our laboratory personnel, some of them have private laboratories and they are cooperating well and that's a good one.  Then, in terms of our human resources too, you know most times some of them don't even want to handle DRTB, they will tell you that they will just push it to the local government. I don't like their wahala (problems) due to their peculiarity in the treatment, what is involved? But now in a way, what you are doing much like the TBLS, like doctors, will just make sure you call a patient and the patient will start coming and he or she knows everybody is monitoring her and notification and cooperation goes for baseline. Ok I'm ready to call them. So, cooperation of the laboratory staff and also the TBLS and doctors. |
| Have there been training logistics? | Training so far, not really and you know you have to be updating your training because knowledge of yesterday might not be useful by today. So, in terms of training, I can say they are still lagging behind but they have been trained before. You know we are doing dot expansion and we are trying to extend most of our dot facility and most of them were trained on DSTB normal TB management care not totally of DR. You know people are changing and we are changing regiments, we are changing drugs and other things, you know that kind of thing.  Training is still needed. They have been trained before just that some of the sets have left so in one way or the other training is needed. |
| Ok let's talk about incentives, what incentives are there to ensure the implementation of the DRTB decentralized services? | I don't think there are incentives.  Maybe in laboratories when they do their tests they will be paid, is that incentive? I don't think so because they are paid for their services |
| Are there other incentives that you may think of? | No, for laboratories, no. Then for CBLS and Dot officers, no, except OPD. OPD the way they do the OPD program, the last day of the month either Wednesday, Thursday, or Friday, the TBLS always support them with lunch that's why the doctor and the dot officers that coke the TBLS supports them with lunch and that's the form of incentive that's been given. So, the OPD incentive is there in terms of managing DRTB patients’ incentive paid as part of the OPD. |
| How about the incentive patients, does it in any way affects the orientation of this program | The incentive, that’s the social and transport allowance they give to patients. Most times we don't give them money we try to accrue to quarter and quarterly bases. They collect #3000 per month, that’s 3x3 that's N9,000. So, most times when they know they are now giving a loan, you see a lot of patients that will come.  But on a normal OPD maybe we will count like 15, 16, but the day members are called by CBOs they will come earlier than said. And, was it not last quarter? One of our doctors frequently saw that when patients come, they will be asking for transport even from doctors, from members of the team, sir please I don't even have money to take me back.  So, most times the doctor will be forced to give them out of their own pocket to please them. Because if you see some people there, there’s hepatitis, there’s HIV, there's TB, that kind of person you will know there's no how you will feel like and that person doesn't have anything to eat. Coming to OPD waiting sometimes, sir I need money to go back and they aren’t have a choice but to start contributing free will contribution from their pocket; the doctor, the TBLS, the Dot officers and when they saw that things were going they will even complain to us; please ma are you going to give the patients something since it's not a quarterly thing. If we come to see them, sometimes they will tell you to give us money too as if we brought money for them. So, in that of patients the incentive given to them we turned it to quarterly, so that it can be heavy. But if we pay it that N3,000 per month now might not accrue to positive bearings. Sometimes We do follow up and the doctor will see all of them |
| But how do you think your facility settings or culture affects the implementation of this decentralized service delivery? | Yes, I can say for instance the facility in Ibadan holds our OPD sites in two places, I’m just using that as an example, if you have anything core related to the chest that's where we go. Even in that hospital, some management of high bases here, they don't go to that hospital they feel like they may be handling their parents. So, anybody that passes that gate like this they will know is TB. It might even be TB so far as you're coughing there's a way like let them be on their own that's it. And another thing is that there's stigmatization, there’s discrimination there is this things. |
| Is there a culture maybe in the staff setting per say. Setting, the environmental setting | Yes, like the example I gave of that other hospital, It’s as if the nurses that comes from there the Doctors they will just do as if that's just if you are having anything cough and in some facility, some places too they put them at distance, like they should not come close to where other people are receiving treatment like normal general OPD site that they have decentralized space for them. So, that is not all but at least we can say that at the scale of 100% I can give them 50% because they are managing. The culture is not really affecting it but is just some cases. |
| Ok what in general setting per say? | General setting, setting of the hospital |
| Yes, about the facility, yes you can talk about the environmental setting | The setting is Ok, just few hospitals that recently they said where they were before they should go out sketch because they notice they are more effective with TB. Normally, the setting is ok |
| And that improves the implementation? | Yes, it will improve it because as you enter there's no discrimination, there's no stigmatization, my patient can easily access me not that go back to that back these are people we don't need to attend to. So, it has improved the implementation already |
| So, but what has been your motivation wanting to have assurance that the implementation was a success? | My motivation is that when patients get care, to me when they get care and start treatment and they start recovering and say Aunty thank you very much I'm getting better. You know that's one motivation and you will be wanting to do more |
| Something like job satisfaction? | Yes, like when one hot mail was calling me in fact that a patient from what they gave her share for us #1000 to say thank you very much, while some patients can do otherwise but my satisfaction is that they are getting care. I have a patient that was calling me to a point he finished his treatment. Aunty thank you, I'm feeling better, I'm feeling fine, thank you for the care, and it’s not as if it's anything just because we are trying to protect this organization in a way. So that's what I can say as motivation for me. |
| How confident are you to be able to implement this decentralized DRTB services regularly in your facility? | Ok, I'm confident about that implementation because it’s working. The aim is for many people to access care closer to them and we can say this is improving our working and time of patients starting treatment and being on treatment because being on treatment is very important to us and finishing treatment is totally cured. So, our confidence is that it has worked and it will work. So far as you're something and it's working, so that thing will work later |
| And your confidence is like you are able to implement that regularly? | Yes, at least we have started before now with the activities as you can see I have made several calls before now and DRTB is still DRTB work is our work so we must find a way to sustain it. Even in CBOs, people that are accessing their treatment have to keep doing what you've started so that it will not fall off along the line. So, I'm confident |
| How about your colleagues, do you think they were able to implement the decentralized DRTB services? | My colleagues, yes, we've been working together on this decentralization project for close to a year now and they have been doing fine. So, I have confidence that they will implement it. They have already implemented it and we are still on the implementation level. So, it’s going on fine and I have confidence they will do that |
| Why did you think so and why do you have such confidence? | Based on their cooperation so far we've been working for over a year not as if we just started working about this decentralization issue and the platform is raised, you see DRTB management even before they see it they start calling everybody that we have seen him or her.  So I'm confident that my colleagues are going to implement the decentralized services |
| So how well does the decentralized services fit into your existing work processes and practice? | Yes it fits in the existing work process in the sense that it has made the work easier and better for accountability and more recordings sake. Like now I know my patients it's not as if I didn't know them before but I know them better now. Like Now you’ve seen this patient today how he or she will get treatment today. So, in that way it's a normal process of work. It is just that it is more channeled today with the way you're doing it. It's as if it takes time but it's the same aim but now we've changed style to make it faster, quicker and better that's it. |
| But, so but it fits into your processes? | Yes it facilitate into the processes |
| Ok, let’s look at whether you think the decentralized DRTB services should replace or complement the centralized one? | Yes as I said earlier we have been practicing the decentralization process in Oyo state since.  So, according to the complement, it complements it and it's still complimenting it, like I said earlier it had the work move better. So, it's completing the work. Parents have access to care, they don't need to go round about before they could get and start lagging behind. And when you get them, you know that this is a matter of urgency and you start treating them immediately, and when there are issues they are calling me ministry of health and everywhere. So it has complemented the work so much. It has reduced our loss to Celeron, loss to phone number except for patients that doesn't have any way to contact them, no phone number, no way to access them. But it has reduced a lot of Celeron and we follow up. You know when patients are on treatment, this person is calling this person is calling, so I find it special that they are calling for this and that and they are giving me their N3,000 per month, and so it has complemented the process. |
| Are you saying we should complement or replace it? | No, it's not a replacement. We don't want the centralized because the decentralized is complementing. You know in terms like the example I gave when we started this conversation about the patient that came and did baseline investigation and we advised him that he should go to the treatment center because he was panicking. So that one is not a replacement but we are trying to get a baseline that can come this way and it can come that way. That is if it works fine, you're going to start your treatment at the community level but if it doesn't work fine you are going to a health center for 2 weeks. So, it’s not replacing it in a way but somehow, somehow there is a clause attached to it that based on baseline investigation patients might need to be admitted in a treatment center and it's also complementing decentralization. So that is it |
| So, the decentralized? | Yes, the decentralized one is what we want.  We want it to stay, but in some cases like the patients that are having cardiac issues or anemic needs to go to treatment center; but it not in all cases but few. |
| Alright, thank you very much |  |

5

KII/A11

We are at Ibadan South West Local Government and the interview is with the State Quality Assurance officer. This interview is going to be conducted by Dr. XX a consultant with XX, Nigeria.

The focus of this interview is going to be on evaluating the effect of interventions for decentralized DRTB services and the factors that have influenced their implementation.

IT; so, sir, I want to start by asking you what you think about TB reach wave 9 interventions aimed at decentralizing DRTB services.

RESP; is a good intervention TB reach wave 9 has been in XX State in the past 2 years, Oyo State and one other state in the federation was use as a parlor study or one of the first 2 years grant that was concluded a few months ago and I happen to be part it from the beginning.

IT; okay.

RESP; as the XX coordinating laboratory activities throughout the 2 years period.

IT; okay, what do you like most about the idea of decentralizing DRTB services?

RESP; yes, it makes the services to be closer to the people that really needs it, it’s more patient friendly than when than it not been decentralized.

IT; can you throw more light as in being more patient friendly?

RESP; yes what I mean is that like the when TB reach came, they encouraged us to add more laboratories that can be doing baseline to the number we have, before the TB reach program we had only two laboratories that were doing baseline investigations for patients that have been diagnosed to have multi drug resistant TB by G experts but when the TB reach came they encouraged us to involve more laboratories in doing baseline so that the turnaround time that it takes for the patient to get their results will no longer be delayed when all the patients have to concentrate on going to a particular lab or only two laboratories within the states and you know our state is a very wide state with a very large area. Oyo State is one of the biggest states in the country and it has about 33 local governments, it has 33 local governments not about and the local governments are so far from each other so before TB reach decentralization should came in, the GeneXpert laboratories when they diagnose the TB disease to be MDR to be multi drug resistant the result has sent to each of the local government and when result are sent to local government there is a need for baseline investigation before the patient can be put on drug at that time we had only 2 if I can remember but of laboratories that can do baseline but now we have close to about 6 laboratories in the state that are now doing baseline investigations for patient prior to enrolment of drug.

IT; is there anything you don’t like about the decentralized DRTB services?

RESP; there is nothing I don’t like.

IT; or something you like the least

RESP; the least?

IT; yes.

RESP; well, the only thing I may say I like the least is that more laboratories still

IT; or maybe we should start with something you like the most?

RESP; what I like the most is that at least the laboratories that have been registered to do baseline have increased from 2 to 6.

IT; okay

RESP; and that has increased the turnaround time of patient getting their result before pre-enrolment. Our patients are diagnosed within 2 to 3 days, their baseline is done, within a week they are already on drug. Those are good achievements.

IT; can we now look at what you like the least about it?

RESP; well, I will say more laboratories can still be identified to get enrolled in the DRTB baseline test investigations.

IT; it’s okay, so let’s look at what influenced your practice of decentralized DRTB services in your state.

RESP; what influenced our practice is that we now have more cases that get their baseline investigations done on time prior to them being put on drug as a result, we are having less cases that are lost to follow up or lost to enrolment.

IT; okay, so what has helped you the most in the implementation of this service delivery?

RESP; well, the fact that most of our G experts has now been optimized, though it’s a continue process the laboratories that does the diagnoses nowadays are working effectively though it’s not without challenges, occasionally we still have case times when there are challenges and some labs will not work for some days maybe due to power failure or maybe the weather is bad, the solar panel is not charging enough or maybe there is a breakdown in AC but things are getting improved.

IT; okay, so can we say that what you mentioned now are some of the challenges you have encountered?

RESP; yes exactly, yes.

IT; can you expatiate more on that or can we go to the next question?

RESP; some of the challenges, I can expatiate more on it because the laboratory is a very complex unit and it is the bedrock of DRTB or TB program in particular, without the laboratory there is no TB program because TB is supposed to be diagnosed bio technologically, 95% it is only 5% that is allowed to be diagnosed clinically so some of the challenges we have are some of our G experts cite the solar panels do not charge the batteries, batteries will not charge on time so when there is low battery or low charging the laboratory will not be able to work efficiently for the desirable number of hours so there are samples but when the battery is low the laboratory will not be able to work so we have to wait until when there is enough sun rays for the solar panel to charge and all the rest so those are some of the challenges we have.

IT; okay, so know let’s look at some of the intervention characteristic of this strategy, so how does decentralized DRTB services compare with the routine existing program in your clinic or organization as the case may be.

RESP; decentralize DRTB services talk more about baseline investigation, I’m I right? What exactly do you mean by, it’s not only done in one place, it is done in

IT; like decentralizing the baseline investigations to more preferable labs, devolving the Gx alert to now include TBLS and the patient as well as the incentives that is given to CBOs which is output based. These are some of the components and decentralizing the treatment initiation to the local government labs. You’ve mention some of those things which brings these things to, I think closer to people and emphasizing community-based treatment rather than facility base treatment, these are some of the things the issue that come up, some of the components. So I want you to look at this decentralize aspect now, the routine existing program that is the former way of doing things

RESP; yes, the decentralized program has really helped, has been helpful. But now we have more cases that are detected and once they are detected within a short time, they are placed on drugs which will also help or it has been helping in reducing the TB body in the state.

IT; compared to the

RESP; compare to how it was before it was decentralized

IT; so, can you talk more on the advantages of this decentralize aspect compared to the previous way of doing it whereby everything was centralized? At the state level

RESP; at the state level. More of the advantages is that we now have more DRTB management centers even in the community unlike before we had only 2 in the state, one is at UCH, the other one is at Jericho where I work but now, we have DRTB cases that are been managed at local government levels in the state.

IT; does the decentralization of the DRTB service have anything to do with improved treatment or lower treatment failures? Or even reduction in loss to follow up or increased cure rate, do you think it has anything to do with that?

RESP; it surely has improved treatment success.

IT; now I’m asking you these are also advantages?

RESP; yes, they are

IT; can you explain more sir? I talked about follow up, loss to follow up, I talked about increased cure rate, I also talked about reduction in treatment failure, so these are just some of the examples that I can think of.

RESP; you know, for reduction in treatment failure when patients are treated close to their facilities, close to their home, close to their place of work that are close to their residence, close to within their community especially in times of harsh condition of transport fare within the country, it makes people more likely to access treatment if they will not have to travel a long distance of about 50 or 100km before getting to a treatment center then patient do not need to get hospitalized anymore since treatment is now based within their community, the patient can access the treatment center daily and still go to their places of work and it is generally more beneficial, more encouraging, it doesn’t dampen the moral of the patient if they have to be hospitalized, before they have to be hospitalized for a long time in the hospital incarcerated within a community within a secluded place but that patient can on community treatment, they can take their drug in the morning from the health center, go to their place of work come back, interact with their family members the following day they can still go and take their drug again or if they refuse to taking it in the evening or when coming back from work , that is so helpful so it encourages better compliance and treatment outcome.

IT; okay let’s look at your facility now, were there changes you needed to do in your facility in order to implement this strategy of decentralizing DRTB services?

RESP; well in my facility the changes that we needed to do is we make sure that once patient samples are treated and the result come out on the G expert machine, we communicate with the requesting dot officer or TBL supervisor as quickly as possible within an hour by telephone call which before we don’t use to do, we only, result are ready pile it up until when the TBLS or dot officer cause back to pick the result which it has part, it has been when next he or she is bringing her new set of sample which can be about a week but nowadays once result are ready we document on our register within a week the information has gone within an hour the information has gone out. The TBLS is already aware that the DR patient diagnose

IT; so are you saying that the introduction of decentralization of DRTB services that documentation has also improved in your facility

RESP; yes. Documentation is improved as well and communication too, communication vice versa

IT; certain things you need to put in place or certain arrangement you needed to make apart from these processes?

RESP; we needed more, people generally need more as to work or more time now to stay in the offices and run samples so that patient result are no longer been delay, and that’s what we’ve been doing to the best of our ability and within the available resources

IT; okay. So, you know about the component of this intervention we’ve talked about some of them. Are there any of them that you think you’ve been altered or not?

RESP; what I believe can be altered it has to do with the communication allowance for people for laboratory officers to quickly disseminate information on patient result that could be altered a bit

IT; okay in that case, any other component should be left as they are

RESP; yes, for now

IT; how complicated is this decentralize DRTB service delivery?

RESP; it’s not complicated at all, it’s simple

IT; why do you say that sir? Can you explain further?

RESP; it is simple almost every local government now, we have 2 to 3 facilities DRTB center treatment in every local government. I don’t work at the treatment center but I know that’s what happens

IT; does that make it simple?

RESP; it makes it more efficient, don’t let me say simple, it makes it more efficient for patient care.

IT; but the bottom line is, it is not complicated?

RESP; it is not complicated

IT; okay. Let’s look at how the DRTB service meets the need of your patient. I mean the decentralize DRTB services, how do they meet the needs of your patient?

RESP; have said it earlier that the patient now gets their sputum samples done on time, and once it’s done, they get their result done on time, they get this time done and they are put on treatment on time so there is reduce loss to follow up, reduced treatment failure

IT; so, in that case their needs medically, I remember you also mentioned their financial need been met to some extent

RESP; yes

IT; okay. So now has there been a strong need to increase or reduce decentralize DRTB services in your facility?

RESP; to increase mean to increase the number of cases I mean number of facilities within the facility, it means to split it into more

IT; do you think there is a need for maybe decentralize DRTB to be increase? That means you have more of it maybe in your facility strength. Do you think there is a small need for that? Or are you of the opinion that rather than been increased it should be reduced?

RESP; when you are talking increment that means you are talking of outside the facility

IT; that is an aspect of it. But then

RESP; but if it’s within the facility it’s going on well within the facility. There are no challenges

IT; okay. But do you think you have a need for it to be enhanced

RESP; yes, it can be enhanced. Like they can be something like a community follow up to patient who may want to differ of the absent once in a while, a time for counseling should engage people to take time to counsel patient very well on the drug and the disease, and it should not be a one often, it usually done at commencement but it should be a continuous thing so that the treatment can be done successfully without it been interrupted on the way

IT; okay thank you very much. Now let’s look at the support that are available to help you adopt decentralize DRTB services in your facility. Any support? Any enabling factor? Anything that has been of support in your adoption of the approach in your facility or in your organization?

RESP; the support has been that materials are provided though at times we have an inadequate supply of some of the next few materials to work with especially things like PPA personal protective equipment like nose mask, N95 nose mask, gloves, conform gloves, disposable lab coat, cover shoes in the lab which are supposed to be supplied on a regular basis, then we have challenges of documentation in terms of the people working this encouragement to things like let me say electrical materials like data to communicate then good working environment, is that related to the question?

IT; yeah, you know we are talking about support, factors that are being of support to your adopting the decentralized service.

RESP; the factors are they that we have equipment that can work

IT; yes, you mentioned it, supplies and equipment

RESP; we have equipment but the supply, the agents are supplied on time, the cartages are supplied on time so we have things to work with but when it comes to only consumables, we have few challenges in that area.

IT; what kinds of incentives are there to ensure the implementation of decentralized DRTB service?

RESP; the only incentives that is there for the laboratory personnel is that when we have virtual meetings. The organization use to supply communication allowance for data, that’s the only incentives we have so far.

IT; are there other incentives that you know of even if it’s not to you?

RESP; well, we have WhatsApp communication, WhatsApp group

IT; is that like a setting?

RESP; It’s not like a setting but people work we through it to communicate within the facility and even outside the facility that this is the situation of things right, now we have a new case, we have a new sample coming, we have this issue, these challenges, these successes so we have WhatsApp communication.

IT; has the WhatsApp communication being of support to your adopting

RESP; of amen support

IT; good, alright let’s look at the culture or the setting of your facility, how does it affect the implementation of decentralized DRTB services, does it help to improve or reduce the implementation?

RESP; it helps to improve.

IT; how?

RESP; my facility is a specialist center for tuberculosis in the state, we don’t attend to any other disease apart from patients that are likely to be TB patients so it has helped because its special.

IT; okay, how do you think your facility. Okay alright. What has been your motivation for wanting to ensure the implementation of this decentralizes DRTB services successful?

RESP; yes, my motivation has been that it’s going to be helping my community because TB anywhere is TB everywhere so once people are getting treated earlier and on time, it helping me its helping everyone. It’s helping the whole community, so

IT; so, the motivation is in a nutshell

RESP; in a nutshell, when my neighbor is okay, I’m okay because if my neighbor is not okay, I may be victim

IT; so, you are looking at it from the perception of poor taking the population

RESP; exactly, poor taking the population

IT; and the reduction maybe in DRTB transmission. Well, let’s look at how confident you are about been able to implement this approach regularly in your facility

RESP; okay. I’m confident about it, I supervise laboratories within the state so I try to do my job with confidence and except an extra person access cannot be a judge. So, it’s you that will say or someone else whether you do the job confidently or not

IT; and you talk about your center been a specialized one for TB treatment and diagnosis, so that also should have an impact of confidence in yourself

RESP; yes sure.

IT; so, can you look at it from that angle and explain more?

RESP; like morning, afternoon, night, what we do in our facility is on tuberculosis so with that I don’t have a diversion of activities. I just think of malaria, HIV, I hardly think of other disease like hypertension whatever so all I think about more is on patient diagnosis treatment, follow up, everything around that circle since it’s a special center on tuberculosis where I work

IT; now how about those who work with you or your colleagues as the case may be? How confident are you about their been able to implement this

RESP; I’m very confident, they are all doing their job very well

IT; you have that confidence?

RESP; yes

IT; okay. So, let’s look at the fitting of decentralize DRTB services delivery. Do you think that it fits well in your existing processes and practices?

RESP; it does fit well. I know it fit well

IT; can you shed more light

RESP; like what? It fit well

IT; yes. I know it fits well, how does it fit well? Yes, granted your office is a specialized one in that area

RESP; yes

IT; now how does it enhance or facilitate suitability so to speak of decentralization of DRTB services into your facility? This are they are positive factors but can you explain more?

RESP; well, we are happy generally because it also helps to reduce the body of activity in our central laboratory because some of the overwhelming things that would have been overwhelming our facility are now done in other facility where they are capable as and capable equipment also doing the job. We have about 15 GeneXpert site, so what is done in our facility are also replicated in other state

IT; yes. I remember we are talking about your facility and the decentralization of DRTB services been well fit

RESP; have told you it fits very well. We get our patient registered on time, we get them diagnosed and we get them on treatment on time

IT; okay. And of course, it also aligns well with your practice

RESP; exactly

IT; well, can you describe how decentralize DRTB services will replace or compliment the routine program or process? Let’s take it this way, do you think that decentralize DRTB should replace or compliment the former way of doing things?

RESP; I will rather say it should replace

IT; why will you say that sir?

RESP; because it is a better, we are achieving better result now with this decentralize treatment services. However, in some circumstances where there is special patient to manage there will still be need for the central specialize hospital to TB in existence so it cannot totally replace, it should not totally replace, it should compliment.

IT; final answer?

RESP; final answer yes.

IT; thank you very much sir

RESP; okay thank you sir, you are welcome.

**KII/A12**

| **Interviewer/facilitator/questions** | **Respondent/responses** |
| --- | --- |
| The focus of the interview is going to be on the interventions to decentralize DRTB services, that is the TB Reach Wave 9 interventions and as a preamble sir I will like to know your opinion on these services? | Thank you very much. About DRTB decentralization services, that is what we are operating currently in XX State and I can say that is a very good innovation. It has actually helped us to actually cope with the large patients I mean the DRTB patients that we have in State and it also assist I mean in making sure that all these patients they were able to access DRTB services at a place that is very closer to them. I mean in their Local Government Area or in their zone in the State as it may apply unlike before at when we are not doing the decentralized DRTB Services. People will have to travel I mean many miles and kilometres to get access to I mean to be diagnose and also to access treatment but with this decentralization, it has really assisted us especially now that we not even you know, we are not encouraging TB treatment at I mean on admission that the people are no longer been admitted except on some few cases. So, this time that we practise mobile I mean DRTB treatment it has really helped. You know this decentralization aspect really helped. |
| Alright sir. Are they things you like most about the decentralized DRTB services? | Yes. I mean this decentralized TB services what I like about it is that I think I have mentioned part of it. The beauty of it is that people can get his treatment I mean both diagnose and treatment I mean they can do their test and they can access treatment in a place that is very closer to them. Some of them they may not have to travel miles and kilometre to access treatment so first of all I like that. Then secondly, those people that are monitoring them you know since they are in their facilities, I mean they vicinity they really able to manage them to see how their taken their drugs and all those things so and that helped us to get the information about them as per the, I mean as per their treatment. How they are complying to their treatments. So, all these things put together there are the things I like about the decentralization and is also prevent something like out of pocket sense you know instead of…people need not to pay huge sum of money to go and all though the treatment if free but if the treatment is free and people are spending a lot of money to go and access care at a very far place is something else so that aspect is no long there. They don’t need to spend huge sum of money to go and do test or to access treatment. |
| Okay. Now are there things you like the least about the intervention? | Sorry the question is? |
| Things that you like the least? | That I don’t like? |
| Yes | About the intervention? |
| Or you like the least, you have talked the one you like the most? | Okay, things that I don’t like about the intervention is that you see we are talking about decentralization because we talking about those patients are been treated in the community. You know before everything use to be in the facility they will be admitted so but later it was letting to do community and treatment in the facility now the problem is that people that even supposed to come to Facility for treatment at times that supposed may be to admit for some time at facility because there is an option of community they will say know they want to stay in the community. That is just the only thing. |
| But is that in any where a disadvantage to the decentralization services? | Well, is not really a disadvantage but is part of the thing like somebody that may be when that person does the test you realize that that person has cardinal issue, you know that person did not know but we know that this is cardinal issue. This kind of patient maybe we should first of all manage the patient for some time in the hospital before we decide to the community but the patient will say “I am okay there is no problem with me since other people are taking treatment in the community let me also stay in the community” |
| What has influenced your practise of decentralized service delivery in your state? | Okay, well just like I earlier mentioned, I think is a very good development and I really like it, for instance in Oyo State now we have different zones in Oyo State like Shaki, Ibarapa zone, Ikeogun Upper Ikeogun, Lower Ikeogun, Ibadan Ibarapa zone, Oyo, ogun and oshon. So, you know before respective of where our patients are, they will have to travel to Ibadan and there are some of those places that are very far that may even be to five hours journey so in the past they will have to travel all the way from that place to come and do investigation and also to access care but now wherever they are in this decentralization they can actually not only do their test but can actually access free treatment |
| So that factor of bring it closer to them is a factor | Is a factor |
| Are there other factors we can look at? That helped in the implementation or influenced the implementation. The enabling factors something like that. | The enabling factor okay, well.  Some other factors like I mean those enabling factor is that we have health care workers that can actually handle them at various zones. So, some of our TBLS not really all of them, at least most of them are actually been trained on how to handle I mean these patients at their various Local Governments so the issue of capable hand is also another factor |
| Are there some challenges you have encountered? | In? |
| The implementation? | Okay, Yes there are some challenges that we have encountered just like I mentioned, anywhere that one it has to do with the issue of social support to patient and also transportation support. You know, if there are been treated in the facility in those days, there are going to provide food for them and even when they will discharge them, they will give them money but now in the community they are having challenges in that aspect of social support where by some of them if you ask them to take the drug but because they are at home they don’t have money to buy food some may just take ones a day some may not even in a day and that has been at times affects I mean their drug intake, their compliance. So those are some of the issues that we encounter practising this decentralized care |
| Sir, thank you very much. Let’s look at some intervention characteristics | Okay |
| So let’s compare this decentralized DRTB services with the routine programme as It affects your State now not clinic though you can also look at it at clinic level | Okay.  I will compare it with the routine services? |
| Yes | Okay, now is it like DSTB and DRTB? |
| No what I mean is decentralized DRTB services vis a vis what it used to be when for instance before the TB Reach Wave 9 I remembered that once there is extension of the farmacine resistance, there is an automated sms message that is sent to you and the state DRTB who will now inform the local government TB supervisor but now I think they has been a change no longer things like that there is this interventions nowmake it possible for not only you but the local government TB supervisor as well as the patients to get that and then what I’m saying is that now and then that is this decentralized against when it is centralized at the state level they had to come for all those services at state level such as the investigations and all that but let’s talk about the reduction in financial burden and all those. I don’t know if I’m cleared | Yes, I understand what you are saying. That is  Okay, well the issue of GXalert, the programme manager DRTB focal person use to receive GS alert in the past but there was a training that was carried out I think for DTBLS, DRTB focal person, M&E officer whereby they talked about this GS alert |
| I think there about 9 interventions incorporated in this effort to decentralize DRTB, what I want you to do sir is to compare this decentralize service delivery now with the routine existing programme just the disadvantages and advantages of one against the other | Okay, for the non-decentralized one whereby patient come to a particular place in the past I mean the advantage of that one is that in those day, all of them will do the text and we make sure they do their test and they go back before we put them on treatment but for decentralized one instead of coming to a particular point whereby they do their test which is really an advantage. So is an advantage to them because they won’t have to send a lot of money to go and access care. Then apart from that under the routine one I was trying to talk about GX alert the other time that DRTB focal person, programme manager will receive their alert then will call the local government TBLS but I think of recent my DRTB focal person can also, there are they was a time she told me because I myself let me be frank I have not confirmed that, they said that patients also are receiving alert but is it true? So but up till now I don’t think patients are still receiving that alert so but programme manager, DRTB focal person, M&E Officer receive the alert and I think Local Government TBLS in the recent time they receive so this one actually helped us to monitor the patients because the way that GX alert has been structured they have actually talked on that because they will only tell us the name of the patients and where they carry out the test so those are the major information that are there. The name of patients, result of the test where the test was carried out I mean the laboratory. We will now look at our hub and school pattern we will know those facilities that gave sample to that lab we will now call TBLS who send this sample then with that we get the name of the patient but that they are receiving GX alert now they also will know, so we can also work together as we are telling them this patients has been diagnose o they also will know that that patients has been diagnosed and the patients with that they will have to go and do baseline I mean baseline investigations for that patient before we commence treatment. So that one is also an advantage. Well the disadvantage of decentralized DRTB care may actually be in term of the test that is been done at times you know at times in some area it may be difficult to actually do all those test in some zones like the challenges that we are having at for instance I think Ibarapa Zone like global fund approved certain amount of money to carry out the test for instance in Ibadan they are able to see some laboratories that we talked to do this test for us this is the amount of money we have that have been approved and they do it for us but outside Ibadan some of them are not complying with that price so at times some test may be done here, it may remain one or two that they may need to send to where you know they comply with the amount that has been offered for that kind of test apart from that the advantage of decentralized care is much more compare to that of non-decentralized. |
| Is alright, so but what kind of changes did you need to make for decentralized service delivery to work effectively in the state | For it to work effectively in the State the kind of changes we make is that we were able to approach some facility both private and public facility so as to be rendering OPD services for our patients and also laboratories, we have to reach out to more laboratories you know so as to engage them to help us carry out baseline investigations and follow up investigation and apart from that even during some of our meetings we enlightening our TBLS and some DOT officers on how to manage DRTB patients and just like you know that DRTB management keep on changing from time to time. That one also we are doing from time to time to actually inform our TBLS and DOT officersso that they will be in tune with the current management of DRTB patients. Those are some of the efforts that we make |
| You mean in the area of capacity building | Capacity building? Not only that I have mentioned capacity building, I have mentioned the issue of engaging more laboratories, engaging more facilities. |
| Is okay sir, looking at the components of this interventions which we have talked about the beginning of this interview are there ones that you think should be changed or altered or not be changed as case may be | Okay thank you very much. You know part of the component is this issue of transportation, you know they give them transportation fare, and they give them social support you know I think some of these things need to be reviewed honestly speaking. Like for instance currently I think transportation is N3000 I mean no social support is N2000 and transportation is N1000 so but with the current economy that things is ee, is too small, it should be proved upon because it should actually be improved because there are some people even transport that they have to take to get to that facility that we said is even very close to them may even be more than that N1000 and for social support in term of you know they have to feed and all those things that money is too small for them and just you I have mentioned it under challenges that there are some people they may not take that drugs regularly because they don’t have food to eat so that area of transportation I mean the area of social support, in the area of ee I think I have mentioned that of Lab, that of laboratory is by the way but may be if they can actually may be if they can actually do it in such a way that money for the test will not be fixed that may be for one zone and another zone the thing could actually you know be done in such a way that what operate in one zone will actually comply it. Then this issue of engagement of those ones that have been treated for TBS TB survivor is actually a welcome development which has really helped us a lot because when those people when they are given a post-treatment I mean pre-treatment counselling because we do engagement them to do a pre-treatment counselling so when they do that it will increase the hope of our patients that this is somebody that useto drug before and that person is okay and if they also too if they use it they also will be okay. Then the issue of that aspect of you know we do more of pre-treatment counselling, that post-treatment counselling most of our patients when they are okay like this, they disappear but if there is a way also we can attach something to that, that once you are okay may be in so, so-so |
| Incentive | Ehe, when you come for follow up we will give you transportation something or one incentive, I think that one will also encourage them you know to come. Okay I think those are the ones I will like to talk about |
| Do you think the decentralized service delivery is complicated? | Is complicated? |
| Is complex yes and how complex do you think the service delivery is | To me I don’t think that service delivery is, is not complex o, honestly before the make it general something, we have been trying it in Oyo State at some level before they now make it general something to be done in Nigeria. So is not complicated infact it should even be encouraged honest speaking. It should be encouraged. |
| Okay but do you think that the service delivery I mean the decentralized service delivery that it meets the need of our patients | Well, actually it meets their need but however there are some area to be improved upon |
| How does it meet their need? Let’s start from there before where we can improve on. | Okay, it meets their need in that I mean they have easy access to care |
| Yes mention some of these but a kind of adding because I know in cause of our discussions we have mentioned some of those needs including financial needs and all that | Exactly |
| So may what you will do now is just to summarise and talk about how? | So all those things they needs are been met however I think we should improve on that area of financing that transportation and social support and not only that you see let me also say this one here all though it may not be for all the patients but for most of our patients in this state it applies to them. Most of them are poor like this aspect of when they want to pay their transport support and social support they say it should be transferred to their accounts. Do you know that some of them are poor to the extent that they have phone. In fact some are even worse than sometimes they don’t have phone they don’t have account, in fact when you even call them to come and do, that you want to do general something for them to open account for them when they are even asked for their name, they don’t even have name,is so terrible.Soa mega amount like N3000 I don’t why somebody that comes for transportation why you will now pay N1000 into the account of that person to first of all go and cash that money before that person will go for treatment, is really an issue I must be sincere with you, is really an issue because some people where they are living even if they are able to open account they will have to use transport to withdraw that money, you understand me and some do not even have accountthat is another thing |
| Thank you very much sir but has there been a strong need to increase or decrease this decentralization | Repeat the question I don’t understand |
| Whether it has been a strong need to increase or decrease or rather reduce the decentralized DRTB services in facility or in the state as the case may be. | I think, actually there is a strong need increase it, to increase the decentralized TB care. There is a strong need. Reason been that, I have mentioned some of those reasons. You want me to emphasize on those reasons? |
| Yes | Okay. Some of those reasons I mean easy access to care is number one. So the issue of even finances I mean the amount of money that they spend of transportation that one will also even be reduced and even to monitor such patients at Local Government Level even become easier and even for them to get their test done become easier for them too. So those are the things I think should be encouraged |
| So let’s talk about some of the support that are available to help you adopt this decentralized DRTB service delivery | Okay, the support that are available, well let’s, we have laboratory I mean this issue of free laboratory services are available, free treatment services are available, logistic system is in place to ensure that drugs get to our bushing also we have transport and social support for these patients although we had seen that it is little but at least it is the foremost support for them which is actually helping them and also the facilities that are there, that wehave been able I means some of our facilities have been upgraded by the government even those ones they are even a kind of support I mean for this decentralized TB care and also the availability of human resources is also another form of support |
| You don’t have problem of human resources in terms of the | What I mean by that is you know DRTB treatment is a specialize care so in that regard is not that we really need so many people to actually do it but however when it comes to usual challenge, we still need to train more people so that we will be able to expand this decentralized care because if it is possible to have in every Local Government area is also good but presently now we don’t have it in all LGAs. You know in Oyo State we have 33 LGAs, if you want to go by LGAs it means we are going to have 33 OPDS sites |
| So let’s say that it is still in the pilot stage that we need a scale up | A scale up yes |
| Is alright now let’s talk about the incentives that they have to ensure the implementation of the decentralized services | About the issue of incentives are presently are those people that are I mean the medical officers or those people that are operating to decentralize TB care, I think there are some incentive that are been given to them. There are so as to assist also for the laboratory I don’t really think, that one definitely they been there even |
| Ok those at that level they may be in a position to address this particular | E those one like the medical officer I think they give them something like is it lunch allowance or.. is it lunch allowance that they give them but the problems is that now there are two forms, one that is been carried out by the global fund and there is another one been carried out by USAID so there is disparity in how it is been implemented from those two programmes I means for those two partners that are supporting TB programme. So in that area of incentives in term of the resources |
| Yes I think you have addressed that, how those incentives now, I think the essence of the incentive is to see that this implementation becomes a success so having mentioned those incentives as there were, can you tell us how your facility’s culture or setting affects the implementation of decentralized service delivery | Okay, our facility culture actually the issue of facility affecting this e, now you know in our facility, there are some facilities whereby we have so many clinics and whereby we have so different kinds of patients coming to that clinic. You know the culture of facility like there are some facilities once they hear somebody has tuberculosis you know they put them aside. This issue of stigmatization is still something that we are fighting against. You know currently in some facility they have to pull our name I mean our DOT officer from the office where he use to attend to patients and move her out of that hospital premises a place near garage to actually be seeing our patients, you know to them is that culture that they don’t want TB to spread and to us is a form of stigmatization because we have been doing it for a quite number of times and it is not really affecting anybody |
| I don’t understand, are you saying sir that that culture of say stigmatization is the kind of hampers the implementation of the | Yes, that is what I’m saying. Although we are just experiencing it in…….facility but in that facility is actually affecting the implementation of the decentralized DRTB service but in some setting the facility culture actually improve the implementation in that in that facility as they are offering OPD services, they are also have in place the laboratory services so which actually help in the implementation of DRTB care. As patient comes there as Dr is seeing them, they don’t have to go to any other place to go and do test they do test right inside that facility |
| Yes and nobody distinguishes who is who as in nobody knows what a patients comes for | No nono, that is not what I’m saying, you know what I’m saying is that you know this is not common to all the facilities, I’m saying in a facility whereby they see patients there and patients done their investigations there rather than some facility to see them the patient still has to go out to some laboratories to go and do test laboratory investigations |
| Okay that facilitates the implementation of the programme | Yes |
| Is alright. So let’s talk about the characteristics of some individuals. So what has been your motivation for wanting to help to ensure that the implementation of this decentralized services is successful | Yes what has been my motivation is that first of all number of lost to follow up. Actually is been reduced. Number of patients that arelost to follow up is been reduced. That’s number one secondly we wereable to monitor our patients very well compared to what it used to be in the past. All these things put together there have been a kind of motivation that if they doing this decentralized TB care our treatment outcome they are actually been a good one. We won’t been having a lot of lost to follow up, they died and all those unfruitful treatment outcome. So with the treatment out that we are having now, I think is good thing to practice, is a motivation for us that if they practise it the more we are going to improve in our treatment, our treatment outcome will actually go higher |
| Okay sir. So how confident are you about being able to implement these services in your State? | Okay, actually is something that we have been doing for quite some time, we are confident that the thing will keep going on, so as we are implementing it and even with the support of ……that we have, we believe the thing will be able to stand. It should be sustained, we are confident |
| How about your colleagues, can you vow for their own confidence? | My colleagues |
| Yes, your staff so to speak | My staff okay, yes, I think we will be able to sustain it with the current staff that we have and some of those people that have been managing I mean DRTB patients the only thing that we just need to do that will just encourage is that we will do training from time to time. Man capacity building so that one is also very important. From time to time. So I believe with that I’m confident that out this thing will be sustained in the state |
| Alright sir. So how does the decentralized service delivery how does it fit into your existing work processes and practise? | Well, I think gradually the decentralized services is being integrated into the normal service delivery. Gradually we are doing that. Although not in many facility but we believe with time we will still be able to increase to other facilities. although there are some you know you were asking me about the issue of human resources the other time about do we have enough human resources and I said well since this DRTB presently moves as if there is not like drug sensitive TB that we have so many of them that we can still cope with them but the issue of human resources will still be a change in the nearest future as we keep on expanding this decentralized care, just like you also you might have seen in the pages of newspaper how people are leaving you know for a greener pastures |
| Now you are saying that it’s well fitted into the processes, the work schedules the as in there is a good fit | Sorry |
| I am saying now this decentralized service delivery thatit is well fitted or that it fits very well into the existing structure in the processes, practise, work schedule and I stopped like that. May be how, even workload in the ease of practise of one’s profession | Yes, I think that one we have fits very well in our work processes and practises. Just like our DRTB centre OPD, we do it every month and they have a particular day whereby they do it, some centre choose Wednesday may be last Wednesday of the month, some last Thursday of the month, some last Tuesday of the month. So, we have a fix day how we run all and people that goes there and all the TBLS that will go with the patient to that place, all these TB survivors that will come to the clinic so we have this structure on ground and which everything is actually going on well |
| Alright sir. So finally, do you think that this decentralized service delivery should replace or complement the current programme and process | Now what do you mean by current programme? |
| You know we have talked about the one this is decentralized and the centralized form | Okay. |
| So my question now is do you think that this decentralized should replace and if yes can you talk about it or if not | Okay. Well right from the onset I am not in favour of centralized care you understand it, because of what is attached to it. The only one that I’m in support is that when the patient need special care like when the patient need admission, I think that patients should actually be admitted. So, I believe decentralized care should actually replace centralized though after all we don’t have centralized care or do we have no. our own is decentralized care so is not the issue of |
| So, it has come to stay? | It has come to stay. Is not issue of replacing it. It has come to stay in the state and I think it should actually continue like that and we are witnessing the benefit of it. So that is just it. The only one that we can just say we want to complement is that of hospital based care so ones patients need hospital based care, patients should actually be allowed. We should not fizzle out that aspect saying that everything should now be decentralized but for centralized something whereby all patients in community they will come to only one facility, we are not in favour of that. |
| Is alright. That’s the end of the interview sir. | Thank you very much |
| Thank you sir | You are welcome |

**KII/A13**

| **Interviewer/facilitator/questions** | **Respondent/responses** |
| --- | --- |
| Thank you very much. The first question I will ask you is what do you think of Reach Wave 9 Interventions to decentralize DRTB services; what do you think about the program ma? | The think of TB survivor is good for health and for body and for our health sir. |
| Okay; how? Can you explain more ma? | What I can explain is when somebody has cough; so if person want to know whether it is TB or not; he or she is supposed to go to hospital to check and know if it is ordinary cough or is TB cough. |
| Okay; you remember that we are talking about decentralized DRTB services; that now certain interventions have been put in place; they can now take their treatment at the community level, they don’t have to go to the centre again; it is no longer centralized and now they are given transport supports even the CBOs get theirs and there are so many interventions; I am asking you what do you think about those interventions?  Are they good?  Do you like them?  Let just hear what you want to say? | All the treatments they have been giving us are good and if person have cough he or she goes to the hospital nearer to him or her and if you can afford going to the farer ones you can go. All I know is that TB medicine is good for the body because it cures our pains. |
| Okay; what do you like most about the interventions? | Like what? |
| About these interventions I have mentioned; I have given you examples; I said that they give transport supports to patients; even the investigations or the tests they do they now do it at the preferable laboratories; that even TB survivors provide them with counselling services (team counselling) and even the CBOs get supports for tracking; I am asking all these things now; is there any one you like most? | I like them they way they are doing it. |
| Which one? | The transport supports.  The treatment they have been proving for us. |
| Okay, for local government? | Yes. |
| Do you think it is better than the time they use to ask you people to go to Ibadan? | You mean the treatment they are giving to us at the hospital? |
| Yes, I am talking about getting the treatment at the local government and going to Ibadan which one do you prefer? | The one at the local government is better. |
| Now, what influenced your practice of decentralized DRTB services in your state? | It is because when I was taking my own treatment the whole thing worked well for me and if I see somebody having cough I help the person by talking to her; I encourage the person to better go to hospital for diagnosis and treatment. If such person refused because of fear I will continue until the person will accept to go. |
| Is it because you have had the experience? | That is it, I have had the experience and also to enable them not die with sickness in their houses. |
| Do you have any challenge with what you have been doing to TB patients so far? | I have not had any challenge because most patients that I have counselled usually come to me for thanks, testimonies and appreciation at the end. |
| Okay, between the DRTB decentralized services and the existing centralized services at Ibadan which one do you prefer?  Any advantage with the one we are doing now? | The one we are doing now is better than the one we were doing before. |
| Why do you say so? | It is because the medicine and small supports for the patients’ transport. Another one is also the food. Those supports encourage us not to be thinking as patients. |
| Okay; would you like any of the components as mentioned before to be changed? | Do you mean the ones they were doing before or the ones they are doing now? |
| The ones they are doing now. | When I got my own treatment, I spent four months before I get cured. I should I advice to come up with drugs and medications that can cure TB patients within the shortest possible of time. |
| Now, we are talking of those interventions like transport supports, communication supports, tracking supports to the CBOs and all those things which are up to 9 if counted; so, which one do you want to be changed? | They are not supposed to change any of them because they are all good and helpful to the patients. |
| From your experience so far; is this work difficult or complicated for you? | It is not difficult oh. |
| Okay; why do you say it is not difficult? | Like what? I said it is not difficult for me. |
| Please I want you to explain? | How do want me to explain it? |
| You said that the job is simple; how is it simple, please explain to me? | It is because I provide the patients with counselling services without any difficulty. |
| Okay, I am listening. | “Cough” |
| I am listening! | Each time I provide counselling services to the patients I do it without stress because of the passion I have for them to get well like me because I was in their conditions before now. |
| Were you trained to do this work? | Yes, I was trained and it is also one of the reasons for my passion for the job. |
| Does the decentralized DRTB services meeting the needs of your patients? | Yes, it is meeting the needs of my patients especially on my area which is using the TB survivors to provide counselling to the patients. |
| How? | Because it has been helping me to talk to patients on the importance of taking their drugs and keeping to other medical advices and rules as provided by health care workers to them when they are on TB treatment and services. |
| Which means if they listen to you and take their medicines they will be well? | Yes, they get well each time they listen to me and follow the medical advices. |
| What of the psychological needs that you made mentioned of before? | You know if a patient doesn’t have money to pay his or her medical bills; it makes the patient to start thinking so much of the condition but if he or she is told that the treatments and services he or she is going to receive are free including feeding and transport; such patients will start getting better even before the receiving the services. |
| Do you want these interventions to increase or decrease? | XX people should do more because their interventions have helped a lot of TB patients in our state. |
| What are those things that motivate you or encourage you to do your work? | It is the love I have for my people and because I was in that condition they are now and others helped me out. |
| Do you fill any form for the job? | Yes, we do fill form and each time we fill the form they must provide financial support for us. They usually provide us with transport money and they equally pay in some amount to our account numbers. |
| So, they provide you with supports? | Yes, especially on transport. |
| Are there others supports that they are providing to people in order to make the interventions work well or continue? | They should continue to give us now so that the work will continue. |
| What are those things that motivate you to do this work? | It is because I have passed through medical experiences and I know how bad it could be for someone to become sick especially TB or cough.  So, now that I am well, I want the good thing that has happened to me to also happen to others who are today in the condition I was. |
| Is this your motivation? | Yes, it is my major motivation because it encourages me a lot to talk to others especially when I remember that I will tell them that the services are free |
| What of your colleagues; do you think they can sustain they interventions like you? | Yes, they can do because all of us have been doing the work very well. |
| How\has the decentralized DRTB services fit with your existing work procedures?  Does this your counselling services to the patients disturbing your work? | It does not disturb my work.  It is not a big job now; it does not do me anything.  Talking to another person does not disturb me. |
| You said that the decentralized DRTB services are better than the centralized one? | Yes, the services are very much better. |
| I want you to tell us; should we use this one and replace the other one or do you want two of them to be working side by side? | I told you before that this one is better than the other one because this one provides patients with relief and better cure. So, this one is better and your supports are highly needed as always. |
| Thank you very much ma. | Thank you. |

**KII/A14**

| **Interviewer/facilitator/questions** | **Respondent/responses** |
| --- | --- |
| So sir having explained the purpose of this interview and obtained your concept, I think we will like to know sir what you think about TB Reach Wave 9 interventions to decentralize DRTB services? | TB Reach Wave 9 intervention program, when it was introduced we did not first understand, but after the operation started we discovered that it was a very good program because we discovered that the project helped so much especially in putting our patients on treatment timely and it also helped in so many other areas of placement of treatment then supervision of treatment of patients on treatment rather so and at the end the treatment outcome.    Initially we use to have buck number of our patients who are diagnosed DR I mean DRTB patients who are diagnosed but are not on treatment. So a number of reasons were given – this one is not living here, he has changed address; he died before result came out and a number of excuses but this project had really helped. It really helped |
| How has it helped? | It has helped like we evaluated during our last quarter meeting it was projected we considered the second quarter of last year 2022 and the second quarter of 2023 and we now compare and saw that the project has helped; 1. It has increased our patients worth not only patients, treatment outcome. It has improved our treatment outcome DR treatment outcome in Oyo State |
| Is alright, thank you very much sir.    What do you like most about these interventions? | About the activities of this interventions there are so many things I like about it, so many things but the one I like most is what our officer use to do calling us and finding out about patients. He will be mentioning the name this so so-so patients what happened to him so |
| You mean the state liaison officer? | Yes state liaison officer. SLO abi? |
| Yes | Yes, He will be calling, in fact that has pushed we TBLS to once to do more, to once to do what is needful about the follow up test for DR patients ensuring that everything is put in shape, ensuring that the result has entered into the treatment cadre and a number of those, so our SLO always call us to mention the name of, he knows the name of patients off heart self |
| So that means his activities are highly upgraded? | Highly commendable, Highly commendable. Oyo state we have that to say about him because he calls us intermediately. |
| Are there other supports that you can talk about? | Regarding this activities? |
| Yes | Yes, aside of those, the payment that is made for our patients especially when they go for follow up test. You see a number of our patients use to complain that we don’t have money to eat, somebody who doesn’t eat is it that person you say go and do your follow up test? But there are little little amount they pay them, infact one of the patients told us that each time they remembered the date they told them to come for, they know they will go and come back with money so it really help, it really help |
| Ok but now, what influenced or affected your practise of decentralized DRTB? | What affected it |
| Or influenced your practise of it? | Ok, ya, decentralization like we use to ask before, we started pushing years back that well, look at our state, our State Oyo State is a disperse state, is not in a point, is not at a point is not only Ibadan, Oyo North, Oyo South, Kinnira, Okogwu, far away Okogwu.  So, now say okay if we have patient and we say that patient should now come just one place. When a patient pays an up board of about 5,6, N7,000( five, six, seven thousand Naira) to come for treatment or for baseline investigations.  It is discouraging, so many of our patients; diagnosed patients have been lost because they will say who will make the payment, who will do this but the decentralization like in my Oyo we have another laboratory, it was decentralized so they go there “with ease-with ease” |
| So in that case, that must have affected a lot of patients to follow up with? | Yes that previous one |
| I mean the decentralization | Yes it has improved. It has brought lost to follow up down |
| Are you saying that what actually influenced you is the fact that things are done better? | Yes things are done better |
| The result you see? | Yes the result we get. When you treat people and there is no improvement, when you treat people and there is no progress, you be discouraged. |
| Alright, so are there challenges that you have encountered? | Yes there are challenges, there are challenges. One of the challenges is well that will be at the managerial level the laboratory because of the turnaround time, the result this time around but each time the result comes out, anyone we diagnosed of DRTB the GX alert will alert us but the most important thing is that this DRTB patients as we have support for them in the follow up in all this in all these.  I think there should also be a social support social economic support for them. Most of them, those who would want to be on treatment and there are not having food to eat, when you find out you see that all these people who are DR patients most of them are what now |
| From poor background sir | From poor background, an indigent. So some of them they can’t find something to eat not to talk of, so some times we discovered that in the past we have so many patients that they will collect their drugs but they will not take the drugs because they know if they take the drug, they must eat and there is nothing to eat. |
| But the intervention already have a little incentives in terms of | Yes it has, it has a little incentive |
| What do you say about that? | The incentive you know the incentive usually come on quarterly basis and that quarterly incentive payment well looks like not been too realistic. If possible if the incentive can be coming at the right time, timely may be the TBLS treating the patients will be paying weekly, be paying monthly you understand but quarterly you know |
| Okay that’s what you see as a challenge? | That’s what I see as a challenge sir. |
| Okay. Can you compare the decentralized DRTB services with the routine program? | Yes, yes we can compare but the one passes the other. Decentralized one is better, it has performed excellently more than the routine one. In the routine system we had perpetual problems and challenges. Perpetual problems and challenges as in we had a number of patients who are not regular on treatment. We have patients who will just determine not to continue treatment even at our places of work there are people, there are workers that are not encouraged by the behaviours of patients so there are not encouraged and so things are but this one is better, is better why because it empowers the TBLS, it empowers the patients. |
| Does it also empower the CBOs, survivors? | Yes it empowers, of course the CBOs are on top now there are the one, it empowers everyone, and it empowers everyone. |
| Alright, so it empowers them on what sense? | Okay, like it empowers we the care giver in the sense that it gives us a sense of responsibility. We know that if we don’t do the SLO will call on us. So it empowers us, it also cause us to have a sense of appreciation, knowing fully well that okay the state is taken note of what we are doing because most times when they call from the state asking about our patients Mosa Ibrahim for example so they know I am taken care of Mosa Ibrahim. Three months after, four months after they still call and that patient should be on the fifth month now. has he gone for the test, has he gone for the follow up test. So all these things sense of appreciation is been created in us |
| Who calls to make these enquires? | Our SLO |
| Alright. So, are there disadvantages? | Of this scheme? |
| Yes | Disadvantage, disadvantage of this scheme I have not seen any |
| Okay now can you tell us the challenges if any that you made in your facility in other to implement this? | In other to implement this, we know that the patients will be treated at the community level, we know that there are number of steps that will be taken especially our environmental approach like initially we place like a shade where patients will sit down but when we know that we have DRTB patients in community at the community level that community TB care we now discovered that there is a need for rearrangement of our place, rearrangement in the sense that we rearrange okay if any DRTB comes he sits here, DRTB sits her then there is a need for garage such that the DRTB patient that comes for drugs does not waste time. So there are a number of changes that we have made. In fact on protecting ourselves too on wearing protective devices, it also improved of course those things are supplied. The protective equipment were supplied and the sense of making effective use of it was done on us |
| Okay, now are there component of this intervention that you think should be altered or not? | Yes I have seen all these components about 9 of them and all these components are excellently designed. I have not seen any one to be altered or, I have not seen any. These are excellent structured components so there is none sir. |
| Is alright, so how complicated is this decentralized service delivery? | Well complication, complication, complication. Okay we have a number of issues like when you go to the patients house at the community level, when you go to a patient house to visit a patient there is a need for caution because of cause you know the patient would want anybody in the community to know that I am, so if we go there officially, officially it hurts patient we have so many that have said no I don’t want you to be coming to my house or if you will come at all don’t wear those shirt you have been wearing. TB is curable shirt we used to wear, don’t take all these bikes with government number with you. Yes, so is one of those things, is one of those things. So is good but I think we have a patient who died eventually because there was a time she was visited and after we left their own community, the landlord now came and asked her why did they come, why this why that and she now said they came to see me now I’m receiving treatment I’m coughing the man said okay leave my house, the woman left the house and eventually she did not come to receive drug again so we started so we started calling I think that should be about 2007 or 2008, we started calling where are you now you don’t come, I will come I will come and we couldn’t track her in that house again so eventually one we heard that the woman died so I think is one of the of the, is community approach that must be so programmed community in such away so that people are not embraced. Thank you sir. |
| Who are not embraced? | I mean patients are not embraced |
| I understand but can you make a suggestion on how to go about it? | How to go about it, the patients or the treatment support will be invited to the facility, Okay we want to see you next tomorrow by so so-so time will you please come? |
| Okay considering the fact that finance is the major issue | Ya if finance is the major issue then we have to, there have been a case like when we say okay take our project motorcycle go and carry that patient but it will not be around the area of that patient, a little farther from the house, the patient will come and... |
| Okay, so you think that is what makes the programme a little bit complicated? | That’s my own opinion sir |
| Is alright. Now do you think that the services of the programme meet the needs of you patients? | It actually met, it met because they commended it, each time they are paid they will come back and say we receive so so-so much o may God bless them oh! |
| So let’s say that is their financial need | Yes |
| Is there any need? | Ya the clinical need too. The clinical need too when they come to take up their drugs and we will tell them even if you don’t want to take we have been doing much now this one we are doing is ok is good now, he will say okay even though the money is not more than may be N3,000 per month is something now if I see somebody that will be given me N3000 in fact somebody has to analysis it and said is N100 per day if somebody is given me N100 per day it’s okay to find something to eat it’s okay. So the scheme actually meet the needs of the patients |
| Does it meet some of their psychological needs? | Psychological yes |
| In the area of counselling using survivors? | Yes, yes-yes, when we have patients like that you know patients are different.Some patients do not want you to know anybody with them because of the stigma. They want their husband, they want their wives |
| But do you think that use of survivors goes a long way in outlaying their anxiety? | Yes, yes you now tell okay, who do you trust, who do you think you can talk to, who, whoever, who do you think, may be your friend o, your landlord may be anybody and that really helped, that really helped |
| You mean the use of? | The use of survivors |
| Okay, so it helped? | Yes it helped it actually helped |
| Meeting their psychological need? | It helped in meeting their psychological needs, because they feel secured with that person. There are some other people, some may not be secured with their husband or their wives and because of their marital stuff |
| Okay, so now let’s look at if there has been a strong need to increase or decrease decentralized DRTB services in your facility, has there been a need to decease or increase? | Don’t decrease it o, is rather been increased |
| Eheeh? | Yes it should rather be increased the reason why is because one all this contact tracing scheme increases the chances of detection of TB then the active care search project all around by the CBOs, organizations increases the chances of having DRTB cases, and in so much it is like that. I think it will be needful to increase the power the effectiveness of the scheme instead of reducing it |
| Is alright. Okay although you have stated before that the activities of state liaison officer has been of immense support, are there other supports or factors supporting you that you can think of about as of now? | Well we have what we call OPD meetings that we hold with our patients at the end of every month. That OPD meeting is locally centred in the facility; all patients in that facility will go and meet their medical officer for clinical assessments. |
| OPD meetings? | OPD yes, OPD meetings at the end of every month so it is there a patient can now complain to a medical officer, I’m having this, I’m experiencing this, I’m doing this I’m doing that so there are some other basic vital signs taken that they will take, they do the BP, the check the pulse every other thing the way the patients were and they will now ask the patients do you have any question and it will also help the OPD meeting also affords us the patients to share experience about themselves if there are anyone who has issues they raise it this is my own case o, is what I’m experiencing the other one will say ha it has happened to me before now don’t worry everything will be okay, somebody who doesn’t want to take treatment before or who is not ready to be regular on treatment when he hear somebody who gets well, he will be encouraged. |
| Okay, apart from you the TBLS and the patients and the doctor who else are usually in the OPD meeting? | We have the patients, we have the contact person of the patients or the survivor of the patient, then we have the medical officer, our DOTs officers too, DOT officers that are managing the patients will be there and that is all. |
| Okay, and such meeting has been of immense support? | It has been of immense support |
| Alright. Any other support you can think of | Aside of the social economic support I suggest sir. The social economic support that I suggest should be improved not for me but for the patients |
| Ehehe okay, but you know I was talking about support | Okay, you are talking about support from this scheme |
| Yes, that are available to you | okay, okay To us, |
| Yes you have mentioned two and I’m asking if there are other ones | No other one, no other one |
| Alright. Okay so you have also mentioned incentives but let’s use this opportunity now to know the incentives that are available I’m asking you the question on what kinds of incentives to ensure the implementation of the decentralized DRTB services successfully? | That what kind of incentives are available |
| Yes | I think the financial incentive is very-very important |
| You have mentioned some of them | Yes I have mentioned them. This one is about what makes this decentralization successful abi |
| Yes | The incentive that really, that made them successful like I mentioned the financial incentive is the pivot one |
| The financial incentive to the | To the patients and treatment supporters |
| Any other ones, to the CBO, to the TBLS? | Yes the CBOs are just partners they are agents supporting partners that help us to, maybe you that the project pays though them and the TBLS of course well there is nothing but the encouragement of the success we have. Job satisfaction when you see whom you have treated and he is getting well that’s enough an incentives. |
| Right we will get to that. Let’s look at how your facility’s culture or setting affects the implementation of this program. | Like you see my own facility is located, is integrated into PHC services and of course is within the compound of PHC facility and when you see somebody in that facility, when they see anybody coughing they will say aa go to them o don’t cough here o go to them don’t cough here so we took step and we let them know that everybody is a potential TB patient off cause everyone who breath in oxygen atmospheric oxygen is a potential patients so that nobody should radicle anyone until you have gone into the laboratory and you come back and the test say you donot incubate even if it is negative you are not still free. So that in a way re-oriented them. It actually helped the movement of our patients in and out. So we also discovered like I mentioned the other time that our arrangement in the facility before we started this scheme, the arrangement changed just to accommodate our DRTB patients |
| Okay should I say that your consciousness of infection, prevention and control informs why you are, part of the reasons you attend to DRTB patients | Yes Sir |
| Immediately | Yes sir |
| And let them go? | Yes sir, you are right, you are right sir |
| Can you explain? | Yes I can explain on that our infection control mentality |
| Was that forms part of your culture? | Yes is part of culture.infection control mentality actually got improved and that helped us to conclude that it is needful for us to attend to our patients on time, quickly and trying to separate them from all other may be DSTB cases to prevent further infection |
| Is okay, so what has been your motivation for wanting to make sure that this programme is a success? | Ya, our motivation like I mentioned, the first is job satisfaction |
| Okay and the second? | And the second is because our patients are being paid. Our patients are being paid our month too can talk say shabi they pay you this so so-so amount what else do you want them to dounlike if nothing is paid to them and we expect them be regular on treatment |
| And you say is a motivation to you? | Is a motivation to us; even paying them is a motivation to us because it helps us to be able to handle them. They paid them they have no excuse and off cause is a motivation to them too |
| Is okay, so how confidence are you about been able to implement the DRTB decentralized services regularly in your facility? | We are confident of it, because it improving our output so we are not embraced by it at all. We are confident because we knowit is result oriented, we have been getting our result. the outcome treatment outcome changed so is encouraging |
| How about your colleagues sir are confidence that they are able to implement the program? | Well, well, well, I can only talk of my own but generally speaking like I mentioned during the last quarterly review it was projected and we see that well centrally the treatment outcome the whole condition of our programme management really improved following this scheme |
| Okay | Yes you were asking about my colleagues |
| Yes, are you confident that they are able to | Yes I’m confident as a result of the presentation we had, it means they also worked. |
| Now does the decentralized DRTB service delivery fit with your existing process and practises? | So perfectly sir |
| How? | So perfectly. Our existing practises was that you see when, okay it was much on DS, but now in the case of DR you know the routine thing is just to send the patients here for treatment to the treatment centres but now because we already have DS patients that already coming for treatment before is not a new scheme the only thing is just that the infection control mentality must be improved on. So it fits perfectly, it fits perfectly well. |
| It’s okay, is not like is challenging or | No is not o is not o, our management is daily clinic nothing has changed |
| Has that increased the volume of work in any way? | Is still the same work now, well it may increase the volume of work but it is still the same work |
| The number of forms you fill | Ya that is expected now, is expected but is still same work though increase the volume any way. |
| Okay, However we can conclude that it aligns well with you processes and practise | Yes, there are separate scheme, they aligned, it increases work volume but there still go together |
| Okay, can you describe whether you think that this decentralized DRTB services whether it should replace the routine one or whether it should be allowed to complement each other? | Well I cannot say that it should replace the routine one because there are severe cases of infection of DRTB infections that may need the attention of the treatment centre so except for those I think the scheme should be rolled in to replace the routine one. One it will increase treatment nearness, it increases treatment uptake, it increases programmatic achievement and a number of those |
| Is okay, how do you think that this programme can be scaled up and sustained? | scaled up and then sustained, this a very big question, scaled up and then sustained ya |
| So let’s start by scaled up | scaled up Ya it can be scaled up by organizing or having a period of reflection with TBLSs and DOT officers on the central aim and objective of this scheme such that they will be carried along in the penates of the scheme and they should come to realization and they should follow the standing order, then sustaining is will be that will be majorly supervisory. Sustaining through regular supervision, regular evaluation of our activities I think we can get there |
| Any other thing you may need to say? | Myself, ya the only thing I want to say is that the organization has closed up their project I would know if possible they can extend for another one more year in Oyo State, yes it will definitely help us. The one has helped us but if they can extend it by one more year |
| Thank you very much sir | You are welcome sir. God bless you sir |
| And bless you |  |

**KII/A15**

| **Interviewer/facilitator/questions** | **Respondent/responses** |
| --- | --- |
| What do you think of TB Reach Wave 9 interventions to decentralise DRTB services? | TB Reach wave 9 intervention is one of our partners in MDRA programme, so they are here to help us with the routine things we have being doing before like the design of scrotum requests form, before we don’t have a contacts person and their phone number include, but now they have redesign everything so that we will be able to track the patients when we get the results. And there so many interventions, like before the TBLS don’t use to receive any alert, except the VLO the accessing officer that will inform us, but now everybody gets the alert on time so that the patient will be able to start his/her drug in a short time. |
| Is there something you like most about the programme? | Yes, what I like most about the programme is decentralisation of everything. Before we have it in one place, but now there is so many labs are helping us so that patients won’t be delayed in treatment. |
| Is there something you don’t really like much, or like least? | No, everything is ok. |
| What affected, or influenced your practice to decentralise DRTB services in your state? | What I can say is that, if you can’t get patient on time. Because patient they like moving from one place to another. So, ones we get the patient, I don’t think there any other problem. |
| What has helped you the most? | What has helped me the most, is the collecting the specimen from the patient, and with the help of linkage officers when they collect the sample they will take it to the laboratory, and when they get to the laboratory without wasting anytime they too do their work, and send us results through our WhatsApp, and GSM alert. |
| Have you had any challenges in the cause of implementing the programme? | There are no challenges, I don’t have |
| Compare the decentralise DRTB services, and the routine existing programme in your clinic? | You know before we collect sample of all the patients needs, or we treat them as presumptive suspects it is later that we, when the results come out as three positive, then we now go for DRTB, but now in as much as we ask the patients have you been treated before, or from somewhere else before coming to the facility. If the patient can tell us the true something, with the help of GeneXpert. So there is no problem, we can diagnose them. |
| Though you have mentioned some few advantages of decentralise DRTB, can you talk more on the advantages? | The advantages is that we get our results in time, then given of transportation support to the patients in as much the patient, we inform the patient come, and do other investigation we are going to give you transportation fee so they will answer us in time, and then patients can get their treatment in the community, or been admitted. That one depend on the our own initiative, whether the patient is bad on the onset he can go for admission, or if the patient is stable to be treated in the community we can treatment in the community, but if the patient is a sturborn one we can ask him to go for admission. Counselling is part of the advantage, we invite the survivor to come and tell them. So, when they see that he/she has been treated before, and that’s one can helps us in our counselling that you are not the first, and you are not the last person this is what happened to me when I started my drugs; able to be fine, counselling is really helpful. Improved tracking with the CBOs is an advantage too. |
| Can you think of any disadvantage? | I don’t think we have any disadvantages, in as much as we have good counselling. |
| What kind of changes do you have to make in your centre, or clinic in order to implement the programme effectively? | Sitting arrangement, we ask the MDR patients to come as early as possible in the morning, or we schedule in the afternoon, because we don’t want them to meet together you know that some cases are more serious than the other. That how we arrange it in the facility. |
| Are there some of the component you want to be altered, changed, or modified? | The only is their financial support to the CBOs they should be given them when necessary. You know what we are practicing now is that they pay them every three month, they should be paid every month to encourage them. becausesome that is the only thing they rely on, considering the economic condition of the country they should be paid monthly. |
| How complicated is the decentralise service delivery? | No complication. |
| Is it easy to implement? | Very easy, when we get our patient from the counselling on set. You know we have counselled them and easily they adjust. with my own patients now I don’t have problem, because from onset I give them good counselling, and I invite TB survival, one of the TB survival will come and talk to them, and they will adjust, and attending OPD meeting every month. |
| How well does service delivery meet the needs of your patients? | From the onset, from the counselling we do follow up investigation. We have told them from onset that every month we will be collecting scrotum sample for culture, and blood test. Instead of them to be going individually to collect it, the laboratory officials will come to their OPD clinic to collect it, they meet everybody there, and there will be no need for the patients to go to the laboratory. So, it is stress free. |
| Does the counselling help in meeting any need? | It’s meet psychologically, we invites the reductive to support them in nutrition, they should not leave the patients, they should move closer to them now that they have started their drugs. It is not contagious again they should not isolate them, they should give them support in feeding, that’s for other nutrition. |
| Has there been a strong needs to increase, or reduce decentralise DRTB service in your facility? | There is no need to reduce, it should continue like that. There is need to increase, because it makes everything easier for both the patient, and TBLS. I have told you their follow up is done when necessary, you know everything is decentralised, and this people are really trying, and laboratory too, you take your sample there they do it on time, and make our work easier.it makes our work easier with the help of decentralisation. |
| What supports are available to you to adopt decentralise DRTB in your facility? | The support, giving of form, the provision of tools for the patient we don’t lack, everything is available unlike before. Recording tools, scrotun request form, all the investigating materials needed by the patients are available, for follow up we give them every month to records in the treatment cards, we don’t lack drugs. |
| Can you tell me about incentives that has been put in place for the programme to be a success? | Financial support to the CBOs for the tracking of patients in case of any defaulter thank God I don’t have any defaulter. Then the financial support for the patients during their OPD clinic for transportation so that there won’t be any complain that there is no money, that’s why I don’t come for OPD clinic, that one too improve the programme unlike before. there are patients that will be complaining that I don’t have transportation fee, but now they are collecting stipend for the transportation fee they will be eager they too or the treatment supporter will be calling to ask when is our treatment day is because they are getting something. They are actually motivated by this financial incentive. |
| How do you think that culture, or setting affects the implementation of this programme in your facility? | Everything goes hand in hand as we are running it before, we ask them monthly for their follow up, so everything goes hand in hand.it does not affects my facility service at all because we have been used to it. What we have been doing before is to improve more on this. |
| What have been your motivation in ensuring that that service delivery is successful? | My motivation is the use of USSD, that is my motivation, giving of money for calling the patients, they are giving us stipend for communication allowance, and when we are giving communication allowance there is no room for excuse so that makes it much easier. That’s motivates me. |
| How confident are you about being able to implement the programme in your facility? | Am confident because it’s part of my job. I have been doing it before; I have been doing TB management since 2006. And I have gone to so many trainings so am use to it. That gives me confident. |
| Do you think that your colleagues are able to implement it regularly also? | Yes, they are able because I give them step down training and they too go for training too, so if am not around they are able to continue with the job. |
| How well does DRTB services delivery fit with your existing work practices, and delivery in your facility? | It is not burden, and it’s not hard to my work. You know from CD to DR the only thing that is different is the months. You know that one is six months regime, and the other is nine months regime, it’s not burden it still part of the work. So, I don’t count it as a burden. |
| So, it aligns well with what you do? | Yes, it aligns well. |
| Do you think that decentralise DRTB should replace the routine programme, or it should complement it? | It should complement it, they should work side by side. The routine one you give proper counselling, and you cannot say if you do follow up under the routine one, you do your normal follow up and you prepare the patient as you are taking your drugs, if things go well, you will complete the six months, but if things didn’t go well you will continue that’s why I say they should complement the each other. Before if you are sick the family will come together to rescue you, the family supports but nowadays there is no family supports. But with the help of decentralise service giving transports allowance, giving financial supports to CBOs, it’s really motivate the patients to continue their drugs. Under the routine one where there is no financial support you have so many defaulters. This one should replace the routine one, because of the reasons I have given. |
| Thank you very much ma for your time and responses. | You are welcome sir. |

**KII/A16**

| **Interviewer/facilitator/questions** | **Respondent/responses** |
| --- | --- |
| So Sir, Having explained the purpose of the research and having accepted to participate in this; I will start by asking you what you think about DRTB with nine interventions aimed at decentralizing DRTB services. | Good afternoon, I'm XX called by XX for the Ibadan south west Local Government.    What you just asked now, in overall what I think the TB wave 9 interventions actuality it's a very good interventions that helps a lot of patience but here at Malete here; what we normally and forcefully do is to send them to the facility so that they can try and get some things they need at the facility like understand the drug very well, because based on community we have challenges with the patients that we treat in the community so that is the first point that I want to raise. |
| You say it help the person? | Yes |
| How? | What I did with the help of the patients in this intervention was that most of the patients were fully welcoming with their families. Most of the families we see as if they are still part of them. Because in some places once you say you want to take someone out from his home, maybe to some facility, some people see it as maybe the person can't get better, maybe that's why they want to take the patients away. |
| Ok, you are talking of the fact that there is now community based treatment | Yes. |
| So that's an advantage? | Yes |
| That's how it has helped the patients? | Yes |
| Ok, is there any other way? | It also helps us to have a full interaction with the patients by going to their houses making friends with the family, then they see that what is happening with the patients is not something that is not welcoming, anybody can come in to see them at any time. Play with them, so most of the patient's family have been thinking of it. I don't think this person will be cured again, and they just see this person that he's getting well again, they are also happy with it. |
| So, it gives you the opportunity to create awareness? | Yes, with 5he family by the time we go to their houses. Then another one of the disadvantages that I see, is the economy factor of the country currently. You know most of the patients need a good diet and we can see now, most of the people that normally come down with DRTB, I mean Drug resistant tuberculosis. They are poor people; they are so poor they don't have money to take care of their family. |
| Ok, maybe there will be a point where you will talk about that. But before we get to that point can you tell me what you like most about this centralization of DRTB services? | Actually, what I like most is, it gave us full courage to have good interaction with my patients. Because most of them will be fearing that is it always that we will be going to the facility, they will not even come to our house, or is our people not supposed to know what is happening with us, so by going to the community we try to enlighten them on what drug resistance is so most of the people now understands especially the family and relatives closer to the DRTB patients. They understand what it means when we talk of drug resistance tuberculosis. So, it's one of my joys, most in the decentralized program. |
| Is there any aspect of it you would like to list or you don't seem to like? | The only thing is that at times the patients will tell us that they don't want us to be coming regularly. |
| Why? | thank you, some will be saying in the community some people will be looking at them and saying is it only you that they will be coming from hospital to come and look after his illness. But I always tell them , don't look at others, focus on your own life. It's your life that is important to you. Don't listen to those people that are trying to wave you away from your well-being. So, to me there are some like that that will tell us that they don't want us to be coming, they prefer to come to the facility. |
| So, what has influenced your practice of this service delivery, I mean the decentralized DRTB in your state? | Well actually, here in Oyo state this program we welcome it. I can say we are the first state to talk of community DRTB treatment because we see that it makes us to more popular in the community, it makes us to get closer to the leaders in the community. So, by doing so it's a very welcoming program in Oyo state and we love it. |
| So, what has helped you? | So, my own, it's some community where I think people don't know me, at times I will just be driving or going nearby, I will just see people shouting my name. |
| So, what has helped you in the implementation or in your practice of this DRTB? What has helped you? Any factor that has helped you? | Well on my own part, the only thing I can say is that it brings joy in my heart, about participating in the program. And also it makes me feel more happy when I see patients I trek in the community get well. |
| have you encountered challenges in the course of the implementation of this intervention? | Yes |
| Can you tell us? | What I have encountered, one thing was the reaction of the drug on the patients when they started the treatment, you know some do vomit and other things like that. So the time some patients will call you will be the time you can't even think of. Someone can be calling you at midnight that this is what is happening to me what would I do. So it's one of the things that makes me feel somehow when it comes to this DRTB decentralization. Because what I saw was that if the patient was in the facility he could easily get the nurses or doctors to get them what they needed at the moment. We do lose some due to the cause of not being able to get what they need instantly. So it's one of those things. |
| So the next question I will ask you is actually the number one question. I will want you to tell me how decentralized DRTB services are compared with the existing program in your clinic? | As I've said earlier, the program is very good, in terms of having good rapport with the patients directly, because when someone is admitted we cannot have access to that patient as we normally have it, in the community treatment. You know we are free to go to the patient's house but In the facility they will tell us this is the time you can do the visiting. So, it's one of the thing that I see most on my own part |
| Are there other advantages? | Another thing I can say is that the patients are always free to say their mind with us. They are free to tell us what is happening even in their family. Like some patients will tell us maybe the wife or the husband is ill, the other partner will be telling us that he can't stay with that person because it's an infectious disease, all that stuff. Part of it is that we have tried to bring back so many houses, so many families together. When it comes to this type of decentralized system than clinic. |
| Such incentives to patients are they advantages? | Yes |
| What I mean are they advantages of decentralization of DRTB services? | Yes, most will tell us that they don't have money for feeding, they may not have time to go to work. So most will say that money is helpful. But they will be talking about how the money is small but the time they got is helpful to them even to some families also. |
| Can you think of any disadvantage? | Actually, is that money should stop |
| No, I mean disadvantage of DRTB services decentralization | Ok, the disadvantage that I can talk about is the prompt contemplation. If a patient was admitted to a facility, you know the nurses and doctors are always there 24hrs. That is one of the things that I see there so they can easily get access to anything that happens to them. But in terms of community there is nothing like that until we are called and we get to the place. So that is one of the disadvantages that I see in comparison with the community and clinic for admission. Then the issue of the money, the incentive that we talked about. What I see there, if someone was admitted also they have an access to this incentive, but most people love to stay at home to receive their treatment and they will use that money within their family also. |
| Ok. Now what kind of changes do you need to make for these services to work effectively in your facility? | Ok thank you, the first thing I can talk about is the good welfare of the patients. |
| Were there changes you made here in order to implement the decentralized DRTB services? | Clinic |
| Yes, facility. Were there changes you made so that you will be able to implement this DRTB service? | Yes, there is. Visitation of the patients regularly is one of the things that we introduced, because at time we do that once in a month before but now once we have a patient we try to know the family, i try to tell my doctor's officials to go to their houses to know where the patients is living, how conducive the environment is. |
| So, you visit more regularly? | Yes, we do that before. |
| Are they under this kind of changes? | Yes |
| Whether infrastructurally, or among the staff but things that you have to do so that this program will be effective | Actually, another thing that we normally do is that here we do get some group of people that come, maybe they want to do charity. So most times we call on the DRTB patients to come, they give them tokens, like incentives, Like raw food and other things like that. I've introduced that one also here when I come to this local government. So we normally do that. Then also from our own end here if any patient comes and tells us that he doesn't have anything we do contribute within ourselves to help the patients because it's good to help others. So we are trying on that also here |
| Now, let's talk about the components of this intervention, are there some of them that should be altered? | Presently? |
| Yes, that should be changed or not | Presently now I don't think anything should be altered. |
| You remember those components like the fact that there is a revolution of GS alert | Actually, what I said then was… |
| That is one, of course, there is support given to the CPOs, financial incentive based on output, there is also the use of issued, as well as the decentralization of treatment initiation at local government level community based treatment. So, my question is all these things that make up the intervention are there ones u think should change, you can tell us. | To me presently, the component is well ok. Then what I just want to bring in is the access of drugs for the patients. To be getting the drug at the facility than looking for maybe, going to the state to get the drug for the patients. So, if they can try and do as they normally distribute drugs for the DRTB patients, if that one can be incorporated it will also be helpful. But in terms of others, it's well ok. |
| You mean the other components? | Yes, it's well ok. |
| Let's talk about the complex nature or otherwise of the service delivery. Do you think that it is complicated? | It is not |
| Can you explain why you think it's not complex or complicated? | In terms of delivery, to me what I see is that, you know I said something earlier that if the drug can be sent down just like we normally have our quarterly drug that was distributed on the DRTB, drug like a third party logistics. So, if the drug come earlier, it will be helping us very-very well in starting the treatment of the patients one time, but another thing that I look at is, you know there is what we call WHO standard of treatment. And We implement the treatment in Oyo state and the patients are doing well. So, if we want to base on that standard, we can be on that standard alone. And that's what we are practicing now presently with the patients. |
| That's ok, you said that the process or rather service delivery decentralization of their services is not complex or complicated meaning it's simple. So, I will want you to shed more light on the whole process, changes and all that. | Actually, what I mean 8s that by the time we discovered the patient most of the time we got the alert late, at times it's early. I don't know whether it's based on the treatment diagnosis center. Yes. Most of the results that come from UCH Gelike, those are the places that we normally use and the Ring Rose teaching hospital at Oyo. We got some results but there are some that will just come late. |
| So, are you saying in a nutshell that it is simple? | It is simple and also not as simple as that. Simplest way of it is that, once they got the patients, once they discovered the patient's drug resistance, the state team got alerted before us and we were the one that took the sample to the facility. Which is supposed to also have that alert on the same day not for the state logistics officer or the GR focal person to start sending the message to us. |
| No, but the idea is for you to get the message directly from the automated machine | I don't |
| Ok you still get the message from the state | From the state DR focal person and even the Mr. Shittu that is in charge of the organization. That last patient that we have is the one that called me and sent the message to my phone. I used to call the patients and they come that same day and we start the treatment that same day. |
| So, your opinion is that it's not complicated? | It's not |
| Ok, how does decentralized service meet the needs of your patients? | Okay, actually this very question. The simplest way I can, just let me say it this way. You know when I talk about the treatment, they got their drug, because most of the time we use to tell them that they know you come for your drug and it is going to be a week that the drug will run off from you. You tell us then we tell them as stated, that so many people will need drugs at so-so time. So, most of the patients at that moment will get their drugs one time. But also in terms of the, that's part of the needs that the patients need from us to get treatment on time. |
| Yes. What I mean is that this DRTB services delivery that is decentralized now, is it able to meet those need that is the services they want | Yes |
| Explain more | That is the reason why I first talked about how we get their drugs on time. It meets their needs because it's one of the most essential things they need and also by having OPD meetings with them. They see doctors at the end of the month, they will tell them what and what they will have as challenges based on their illness. So, from there they will still tell them some other thing that we need to incorporate in their treatment for them. So, it's helpful and also the incentive that was given to them monthly also is also helpful and especially the treatment supporters it's goes a lot with the situation of the country now. So that's what I could say about that now. |
| So, in your facility has there been a strong need to increase or reduce decentralized DRTBs services? | In terms of increments. |
| Yes, I said, has there been any need? | Ok, I now understand. Actually in terms of increments you know what they. The situation of the country presently in term of fueling and some other things. So I mean in terms of transportation to some D.R patients' houses. How long it stays now. Like now that the fuel has increase we don't normally go as do before, so that one is if they can do something on giving us incensement on visitation on someone that will go to access the patients at home. At the patient's convenience time then for the reduction. I don't know if there is anything. |
| Ok you have explained that there is a need for increase, maybe there may not be a need for reduction. So let us look at the support system. Has there been any support that is available to help you adopt the service delivery we are talking about in your facility? | Actually, what I can talk about, is the financial capacity of it as I have said with the last question that I just talked about. So if there is any support I can render for us to help us to make the visitation to be more than we normally do it could have been better in terms of the DRTB service delivery in our own facility here so if you have some little support in terms of finance. |
| I understand what you are saying but my question is what support you have had that is available? | Like the one I have said earlier, those people that give them incentive you know I made mention of that. |
| Which has made you adopt a decentralized system? | Yes. So, if that one can also be done. If we can be getting little things for the patients that will make them feel that it's not only drugs that we blaze on. Actually to me I can say from us here once the patients come if we have anything, we give them. |
| Ok. Remember what I ask is the support you have had or the available support for you to adopt this service delivery in your facility. Are there factors that have been useful to you that have been supportive to you in this facility for you to adopt decentralized DRTB services delivery in this facility. That is the question. You may have mentioned one or two of this support but I will like it, if you will encapsulate this point. | With the support that are available to help the adoption |
| Have they been capacity training? Things like that. Has there been things like a WhatsApp platform? Can you talk about things like that, have they been helpful, you know such things. | Actually, let me say in terms of training capacity and all the national people with some organization, like your own organization, will train us on WhatsApp groups. It helps base on the treatment. Because of the new system that we have now on the treatment of the D.R patients. There are some that we go through the newly trained virtual training we did on WhatsApp, then some side effects of the drug also. The support in terms of the training is helping us in this facility because there is something that at times, we think is normal for patients but by the time we go to refresh for the training we see that that is not normal for the patients to be telling us. So that part is helpful in terms of a decentralized system in our own facility here. So, in terms of training. Then, let me basically base on the training for now. |
| Are there some incentives to ensure that the implementation of DRTB decentralization is successful? Are there some incentives you know about? | For the patients or to the health workers. |
| I'm sure you may have mentioned one or two | Yes, as for the patients, what they normally do for them as have said earlier is helpful to them and it's working successfully. Then in terms of the visitation team most of it we do on our own. And that one also is also successful for us to go to the patients houses, have an interaction section with them, ask them their problems, the one we can solve we solve it, the ones that we cannot solve we do counsel them on it. So that is just it. But the incentive is helpful for the patients. |
| Ok. Let's talk about the facility setting or culture so to speak, you know maybe the setting of the facility is there anyway, it has affected the implementation of this service delivery that has improved or reduced. | Yes, I can say I have improved. We don't have anything reduced |
| Ok, but can you explain how it has increased. | Actually, by the time we had the D.R patients we did screen the family also. So, in terms of that, many people by the time they get to this facility will just mention that, this small place, this is the place where many people are getting well. To us it means all joy and makes us proud of the facility where we are. Well, another thing that I see is that by doing that In terms of culture we do let many patients. We have good interaction with them to know their spiritual status at times. We do counsel them, you guys need to be prayerful. |
| Let's say the culture you are talking about here now maybe in this facility you have your way of doing this. | Yes, I'm not talking about the religion or the culture of this place. What I'm trying to tell them is that they need to be prayerful because in everything that comes up with anyone either good or bad someone needs to be prayerful. So, this environment or let me say this community as a whole we have so many things that I can say can be a problem in this environment. The environment is not conducive. In terms of overcrowding. You will see so many houses lump together. No fence, window to window. From house to house. It helps us to get more patients because by the time we started doing community mobilization screening in the community we do get more patients. So, it improves our work in the facility here. By getting more patients from the communities. So the number of patients we are getting is getting higher. Because of the system of moving through the community street awareness screening activity so it helps a lot now |
| So now, what has been your motivation for wanting to help and ensure that decentralized DRTB services are successful? | My motivation. Hmmmm, the only thing that I can mention is that the drugs that we are using should not be out of stock. At the federal level, state level and local level. Yes, they should not be out stock of DRTB patients in Any of their drug, they should not be. |
| I mentioned motivation. Are they things that have motivated your interest? | The only thing is getting the patients and get them well. That is the only thing that I see there, that is my joy. I don't have anything motivating me other than getting the patients one time and get them with prompt treatment. |
| How confidence are you about being able to implement the DRTB services regularly in your facility? | Ok what I want to talk about is the implementation of this in my own facility here, the confidence that it give me is that 8t makes many people look at me as someone that once they got to the person anything that is their problem he will solve it for them |
| Okay. Yes, that is understood. But for you what is your confidence that you are able to implement the services regularly in this facility? | Well, if we are not confident in the community level  Treatment. Most of the patients we treat in the community there will be a lot of red alerts at the national level maybe the patients are not getting well, maybe a lost to follow up |
| So, in other words you are saying you are very confident? | Yes, confidence in community based treatment for DR |
| That you are confidence that you are able to implement this service delivery regularly in this facility? | Yes |
| How about your confidence in your colleagues? | Same thing because my doctor officers and the other people that we are working together. I have confidence in them because when I'm not around they are the ones to take care of the patients so if they did any wrong management I could have known. So, I have confidence in them also. |
| So let's look at how DRTB services fit into your work processes and practices. The work you've been doing here the practices I mean the work you have been doing here the processes, the practice talking about the schedule, whether the service delivery DRTB decentralized service delivery. Does it fit well into those services? | It is |
| Ok, can you explain how it is? | Because to us we see DRTB treatment as a DS like treatment. Because the only difference is the number of drugs they are taking, the way we manage our DSTB patients is the same way we manage our DRTB patients. We don't see any difference there. There is but it's not like other ailments. So, the delivery is fitting with the existing working and practices system in our own facility. We don't have any issue, the only thing is you know their own culture and here sample collection is monthly so we will be practicing that we are fix in to heat. And the other normal things that they normally do when they go for OPD they do the other necessary thing with them. So, the system we are ok with and the process is also ok. So, it fits in with the system we are using and we are practicing with this facility. |
| Ok we have integrated the | We see D.R treatment as D.S treatment also. The only thing is that we give more care to the DR patients. You know DR patients don't normally come daily. They don't come as frequently like DSTB patients. So that is the reason why I talk about visitation of the patients at home at times. Most times they do call if they have anything and we answer them on call. If it's necessary but if it's necessary to come down we tell them to come down so we talk face to face. |
| Ok. Can you describe how decentralized DRTB services will replace or complement the current program or process? Do you think it should replace the current program or it should complement it? | Actually, you know what I am talking about, I firstly mention the disadvantages for some of these patients. When they are at home in terms of drug reaction, then in terms of feeding also, maybe two weeks or anytime at the facility or most people will just tell us,” ahh oga see oo the food wey dem dey give us there I no fit get that kind food for house oo” (sir the type of food they are giving us here is rich and we cannot afford the same diet at our homes oh!); and they can't be there for the rest of their treatment. So that is one of the things I can see as challenge for most of these patients with the present program in terms of decentralization of this program from facility to home base and mostly we talk about the care that they get at the facility. That they prefer it than staying at home, You, know before we started the treating of the DRTB patients you know they went on four months admissions. So, most of them the way they were fed they really love it. |
| So are you saying that the decentralized DRTB service delivery should replace the former? | Yes, that is if, there is if there. If the incentive was given to the patients, It is going to be one of the things that can help them because most of the patients financially are down and they don't have anybody to support them in terms of feeding. |
| What of in terms of out born in terms of the reduced gap between treatment diagnosis and in follow up in terms of reduction in numbers or lost in follow up. What of all these other ones are the reasons why they should replace it. | Actually, with the number of patients we have. Let me talk about the facility now. Most of our patients got all their patients and they are all ok. Last year we lost only one patient. Then all the remaining ones like six or seven in this facility. They complete their treatment. So being admitted, being in the community even to be in the community to be I prefer it to being in the facility because they have access to their family at any time. We can even talk to their family at any time they want. So. it's better than to get them admitted or isolated from their family. |
| So, are you saying we should complement? | Yes, we should complement it. |
| Or replace it? | No!!!. Let it continue the way it is going, it is well okay. |
| Thank you very much. | You're welcome sir |

**KII/A17**

| **Interviewer/facilitator/questions** | **Respondent/responses** |
| --- | --- |
| As an ice breaker, what do you think about TB Reach Wave 9 intervention to decentralize DRTB services in your state? | First of all, I want to thank the organizers of this programme. Before the programme started, we were trained at the state level” As the adage says no knowledge is a waste’’. We went for the training, and started the implementation after the training. |
| What do you like most about the intervention? | This intervention is like what we call delegation. What I like most in it, is that the programme, treatment, and service were given to the DRTB patients at their door step. The door centre, the facility where the patients will be receiving treatment is very near to where the patients are residing. The door centre is a trained DOT officer, and instead collating all the DRTB patients at the same point decentralising the service at their door step is very effective, and we are appreciating it. |
| Is there something you like about it, from what you said you talked about taking the treatment initiation to the community level? looking at the other ones such as GS alert to TBLS,and patients, financial support to CBOs, use of team counselling with the DRTB survivals, and also decentralising investigation to peripheral facilities. Not forgetting transport support to patients for baseline investigation, use of USSD, then verbal autopsy for investigating reports of a treatment tent, then the engagement of liaison officers. You have mentioned the one you like most, which one do you think should come last? | All the plans are good initiative, and a welcome development, but the one that I want you to do most,for example in my zone XX there is no baseline investigation laboratory, and it affects us most. |
| Remember I listed all these, and I said which one do you think should come last in the other of importance? | The one I think is the use of USSD and the use of verbal autopsy, but transport supports to patients on baseline investigation should be put into consideration, because it helps a lot. |
| Now the transportation support to patients, and support to CBOs, and taking the treatment initiative to the communities, which one should come first? | Taking the treatment to the community level is still the first, and most important. |
| What affected, or influenced your practice of a decentralise DRTB service in your state? | It affects our work positively, because decentralising the services helps to reduce the heavy work in the office.by the time some DOTs officer attending to their patient here, I will be busy doing another thing in our office, so is like a delegation, when you delegate you have a lesser work to do, and the work will move on. |
| Is that what affected, or influenced your own practice of decentralised service delivery? | Ideally, it influenced. |
| Can you explain how it influenced? | It influenced in the sense that, people appreciate good things, and am influenced by that. When you have good products with lesser price people will be rushing to buy it. |
| Is that what influence you the most? | Yes |
| Have you experienced challenges in the process of implementation of this approach? | Thank you very much, the only challenge that I have concerning our zone, and all the facilities under that zone is that there is no baseline investigation laboratory, we use to come from Ibarapa to Ibadan for baseline investigation laboratory. I have to tell you the truth, even now. |
| Let talk about some of the characteristics of the intervention. I want you to compare the decentralised service with the routine programme in your clinic? | The decentralised services is very good more than Routine service. Because for our own it saves our time, and patients are receiving drugs/treatment at their door step, for the patients it reduces their transportation cost, reduces stress, and even adherence to treatment has improved. I can easily walk down and get drugs instead of taken bike, or motor down to routine area. |
| Does it also affected the lost to follow up? | Yes, it also helped us to follow up patients. we don’t have, the degree/number of lost to follow patients is coming down. |
| What about the time between the treatment diagnosis, and treatment? | The treatment diagnosis, and treatment is higher. Delay for treatment has reduced. |
| Are there some disadvantages? | The advantages are more than the disadvantages. Pertaining to transport, treatmentdiagnosis everything is moving smoothly/forward. The programme is very good, and the initiative is a welcome development. |
| Is there any disadvantage you can remember? | In our zone, the disadvantage is that when we need DRTB drugs we may need to call them, we find it difficult to get the drugs at the due time, because of bad road network, and security challenges. |
| What are the changes you made in your facility to be able to implement decentralised services? | The changes we made is that, for the DRTB drugs/patients, I appointed a desk officer that will be in charge of the DRTB programme/services in the facility. But before the decentralised service came, routinely we use to gather the patients together, and talk to them, but now we split the work accordingly, delegation reduces work as I said earlier. |
| Is there any other changes you needed to do then in order to implement? | What we needed, there are something like hand washing facilities we provided, then the structure was dilapidated. |
| Do you have to touch your structure to be able to implement this programme effectively? | Yes, we want God to help everyone of us, including the organization to make the environment conducive for officers that are working there. |
| Are there some of the components you want to be changed, or not? | The entire component is good, but we have to put it in order of priority as I said earlier. Just to summarise what’s needed to be changed is USSD, and verbal autopsy. These ones are not all that is important to the programme. Since we have concluded that the patients of DRTB, the baseline should follow, after which transportation to centre to the patients, then the liaison officers should be encouraged by giving them the best financially, and otherwise to ensure commitment. |
| How complicated is decentralise DRTB services in your opinion? | It has no complication, the system is very simple for the implementor, and the receiver. Because it has advantages more than disadvantages. It benefited both the service provider, and the service receiver. |
| How does it meet the need of your patients? | It meets the need of our patients in the sense that taking food to someone that need the food, that’s how it is. You are taking treatment to the door step of a person who is seek, reduces stress, reduces cost, and reduces waste of time, and it very good to both service provider, and the receiver. They compliance to drug administration. It meets 95% of their medical needs, by getting cured from their illness after taken the drugs. |
| Does transport support to some extent meet their financial needs? | The transport support that the organization is giving to the patients helps a lot, because it meets their financial problems, and they also make use of it. When they get the money, they use it to go where they suppose to go. |
| Does taking treatment to the community meet their need? | It helps a lot, when you take drugs their door steps, there will be no need to stress them, and it encourages quick recovery from the diseases. |
| In your facility, has there been a need to increase, or decrease the decentralised services? | There is no need to decrease anything. Like Oliver Twist we need more, because we are enjoying the services, because it has more advantages more than disadvantages, and you need more of anything that has more advantages. We don’t need reduction, we need improvement. |
| What support is available to help you adopt this approach you have being talking about in your facility? | There is a platform that comprises all the DRTB laboratories, and comprises all of us. It is form of support because from there we can know a facility, or somebody who is providing the DRTB services in Ibadan. Through the platform we can be able to detect patient who is receiving treatment in another facility. It has been helping us, and can still help us in future, and it good. |
| You still go to Ibadan for investigation, how will you overcome it, What support do you have in overcoming that? | That is what we want in future; we have been managing to come from, and the organization supports from our end here where baseline investigation is done. There is a form of support for the movement. |
| What kind of incentives are there to ensure the implementation of decentralise services? | There are so many incentives I can talk about, transport support, CBOs, the laboratory officer have great support from the organization. The transport support for the CBOs, and patients must not be removed, unless if anything is done like bringing the Doctors, and Laboratory officials down to the communities for the baseline investigations. If not, the financial support must go on. |
| How does culture affect the implementation of decentralise DRTB in your facility? | It improves/smoothens the implementation of the programme in the sense that, before the decentralisation, we use to gather all the patients in one place for health etiquette/ patients conducts. The culture has been helpful in the implementation of the programme. |
| What has been the sources of your motivation in ensuring that the implementation is successful? | What motivate me most is that decentralising the treatment, encourages delegation, and reduces stress on the part of the health giver, and the patients. The Patients compliance to drug administration, and quick recovery is quiet motivating. |
| How confident are you about being able to implement the programme regularly in your facility? | There is a great confident, my patients knows me very well, and before you can treat anybody you must know the person’s background, and having confident between the health giver, and the patient produces better results, and smoothens the implementation of the programme. |
| What about your colleague, are confident that they can be able to implement the programme in your facility? | I have 100% confident in them. The reason being that even if am not around, there is no hiding thing,they will do the job as if am around. This programme encourages delegation of duties, when you delegate you have time to do another thing. But if you don’t have confidence on your workers in the facility the programme will fail. I have confidence in my colleague/staff in their ability to implement the program regularly. |
| How well does the decentralisation approach, DRTB service delivery fit with the existing practices? | It does well. Is like we are using one stick to kill many rats. That decentralising services do not interrupt our regular activities, it did not affect each other, it helps. If the programme gives us something (money) to buy things, it will be useful for other programme in the same facility. |
| So, in order words you are saying that the programme fits well into your practices? | Yes, it fits well into my practices, they allaying well. No cause for alarm. |
| Do you see it as increasing your work burden, is it time consuming? | No, it doesn’t increase our work burden; it is not time consuming, because there is delegation of duties. It does not have negative effects on the existing procedure, or process. |
| Do you think that this approach should be replaced, or complement the current programme? | Thank you sir, we are begging you, don’t remove the programme. We want you to put more efforts to sponsor it, to complement the existing one. |
| What do you mean by existing, do you mean the routine programme, we should complement, and not to replace? | Yes, the routine programme. The decentralise programme is better than the previous one.It should replace the old one. Base on my previous response. |
| What are your final words? | My final word is to appreciate the programme organizers. |
| Thank you very much sir. | You are welcome. |

**KII/A18**

| **Interviewer/facilitator/questions** | **Respondent/responses** |
| --- | --- |
| Good afternoon ma | Good afternoon sir |
| So ma having gotten consent after you have looked through all the aspect including the purpose of the interview and understood everything, I think I can now proceed by asking you what you think about TB Reach Wave 9 interventions to decentralize DRTB services? | The TB Wave 9 interventions the supporter of the program and they will make it easy for us to do the job |
| Ok, how does it make it easy for you to do job? | Because before the decentralize, the work is more difficult but as it is now is make it easy to do the work effectively |
| Can you explain it? | I mean before, before the decentralization everybody from all the Local Government we have to come to Ibadan |
| Ok | But for the opportunity given to us now, so |
| Ok now is taken to the Community | “Yaa” |
| Taken close to | To the people |
| Alright, what do you like most about, you know about the component? Is there any of the component that you like most or the one that you like the least | The issue of supporting the patients is one of it in which is motivate the patient even to cooperate with us |
| Ok, you mean the transport support to patients for their baseline investigations? | “Yaa” then the issue of getting their drug in the community so is also help them to cooperate and to adhere to their treatment |
| So they’re the ones that come uppermost in the order of importance, these two you have mentioned now? | The issue of supporting, come closer to them, so |
| So of all the components now; which one do you think should come last? | The two is important |
| Yes, there are other ones, other components, you’ve talked about the ones that should come first and I’m now saying which one should be given the least? | The issue of financial support |
| Should be given the least? | No, the least |
| Remember you said, you already said that the financial support, are you talking of the financial support to the CBOs? | To the patients, I mean to the patients |
| That is the transport | Support |
| You said that one that the fact that the treatment is taken to the community are the ones that you like most | “Ya yaya” |
| Now, I’m now saying the one that you like the least. If we say arrange this thing now, from one to nine for instance which one should come last | The use of verbal autopsy? |
| Verbal autopsy, ok, is alright that is for investigating the pre-treatment, the report of re-treatment test | “Long pause” |
| Let’s look at what affected your practice of decentralized DRTB services in your State or let’s say in your facility as the case may be. What it affects you or influenced you | What? |
| Yes, what helped you most? What helped you to be able to be a part of it? | The issue of been the same platform getting the alert is one of it then. |
| Is something, ok it has helped you? | Yes. |
| So been in the platform that is the WhatsApp platform? | Yes |
| Ok is a source of encouragement? | Ya, it helped us to know as early as possible if you have patient. You can’t be waiting for the lab man to call us until you get there but as soon as result is our you get the alert, you be able to know ok, one of my patients is DL so you will be able to contact the patient as early as possible |
| Ok, in the process of implementing this program, have you encountered any challenge or challenges? | Challenges, in my own Local Government anywhere, the challenge I can say is the issue of laboratory. So we don’t have lab in my own local government |
| Ok, so can you explain more | What I’m saying is that for us to do any sample to diagnose, no laboratory, no laboratory |
| So what do you do? | We take it to Ibadan |
| Ok, you take the sample to Ibadan | Ee, but recently we have one at Ibarapa |
| Is it central, is it Ibarapa Central? | Ok, the issue of baseline, No we don’t have any laboratory there to do something, nothing. |
| Ok, now let’s look at the intervention characteristics and I want to how decentralized DRTB services compared with the routine program, as in I want you to compare this decentralized approach we are discussing now compare it with the former one? | You know before if you have any patient the patient will come to Ibadan but now we can you know take care of that patient in my place now |
| Are there other things you can say in comparing the two | This decentralize to me o is better |
| How? | I have said it earlier that it makes it easy for the patient to receive their treatment |
| Ok, because it is taken to the community | “Yaa” |
| Ok, what about the alert | Yes it help us that is what I said, it helped us a lot. |
| What about the counseling with the TB survivors? | Yes it also helped. This issue of decentralized also help. |
| Are there other ones? |  |
| Engagement of liaison officer? | Liaison …. |
| Yes the liaison officer for coordination of the program, in this case is Mr. Shittu that does that. Is that also is an advantage or an aspect, a good aspect of decentralized process? | Yes, ok-ok-ok, even at times like myself maybe I have patient, even before we go through our WhatsApp Mr. Shitu will surely call. He usually call me |
| Is ok, are there disadvantages associated with decentralized DRTB service? | Disadvantages, for now I don’t think of anyone. I don’t think I have for now. |
| What kind of changes do you need to make decentralized DRTB to work effectively in your facility? | That is one of the, the issue of challenge so if there is. |
| I said changes | Changes |
| You made in your facility, that is the place where you work for you to be able to effectively implement the decentralized DRTB services, where there things you have to put in place | Ok, like the issue of washing of hands, the issue of peeping. |
| Did you get all these things, did you provide | Yes-yes we have oh! |
| I’m saying did you have to get all these things in order to implement our program. Just tell me |  |
| You mentioned washing of hand | Ee washing of our hands |
| Did you have to may provide washing hand basins for the process | Yes, in fact for the XX have they.. |
| No I’m asking you the things you did in your facility? | We have washing hand basins now given to us by our Local Government and we have been using it |
| Ok, are there other things you did? | Then hand sanitizer, Chair, we don’t have chair |
| Ok, | So that is part of our challenges anywhere, no chair |
| Yes we will come to that but you know all that I ask you now are the ones you put in place so that the program well done |  |
| Ok, let’s look at now this component we have been talking about, are there any of the component you want to be changed | Any of the components to be changed? |
| Yes or altered or improved on as the case may be or are there ok the way there are? | They can still improve on it |
| On which one | The even the issue of finance, the financial support |
| Ok, financial support to the, is it to the | To the patients and even to the TBLS self |
| Ok why do you think the finance should be increase on | Due to the situation of this country self. I think they should improve on it |
| Explain more on this, can you explain more ma? | Like the one given to my one patient now, for somebody to come from Igorgor down to Ibadan, the money given to them is not ee, let me say it is not enough because from Igorgor to Akpayite that is my Local Government Area, if you want to come to Ibadan now; your to and fro is N8,500. At least they will come for their follow up tests. |
| Ok, how complicated is the system, I mean the decentralized DRTB service delivery, how complicated do you think it is? | It is not complica, no complicated as e…. |
| As in the process of implementation for instance is it so complex? Is it complicated? | No is not |
| Ok, so is it simply or easy to implement? | It is easy |
| Can you explain ma? Why you said it is easy | Though if you want to look at it by my own perspective |
| Yes, yes let’s look at it by your own perspective | It add to our job but we are able to you know cope due to the passion we have for this program |
| Ok | Let me say due to the passion I have for the program |
| Ok, is alright |  |
| So how well does the decentralized DRTB services meet the needs of your patients | My patients? |
| Yes | I have said it |
| How, the needs of your patients, how the program, I mean the decentralized DRTB services delivery meets to them. The demands of the patients may be their medical unit, some of the | Yes like one of my patients now she has you know gone to many places, I mean the mother of that patient, the patient the age is around 12, 12years so is a child. So the mother has gone to several places but when she came to us and she got the treatment and the baby is fine now in which she has loosed hope before but now at least she is doing fine even the mother and the patient herself so medically the patient is ok now. She can join the fellow in the school. So even concerning that financial aspect because before she started this treatment nothing, nothing but by the time we gave her that little amount she really appreciated it. |
| It ok, thank you very much |  |
| So in your facility has there been a strong need to increase or decrease decentralized DRTB services | To increase it |
| Yes | In my own centre or LGA as a whole |
| Has there been a need strong need to increase the service delivery | Yes |
| Ok why, why do you think there is a need to increase the services | So as to motivate you know our patients |
| Ok | I think it will serve as a way of motivating the patients even we that you are taken care of this patients too |
| Ok, you mean the intervention will help to motivate both the patients | And the staff, both the TBLS and the Doctors |
| Ok, what supports are available for you to adopt the approach in your facility? I mean this approach we are talking about the decentralized DRTB services | Supports |
| Yes | From e… |
| Support that are available in your, that is to you, to help you adopt it | Ok-ok the issue of training |
| Ok | So we have been trained so as to do the work effectively. So is part of it |
| Is there other support that you can think of that helped you in the adoption of the program. | Apart from the one I have said |
| Ok if there is none you talk of let’s about the incentives | The … |
| Incentives, what incentives are there to ensure that the proper implementation of the program. | Incentives from |
| May be incentives from the program for instance we have been talking about the incentive now the transport to the patients for instance there is output based financial incentives to CBOs to enhance the tracking of patients, they may also be an incentive to TBLS, so lets | The one that motivates us? |
| Yes I’m talking of the one that are there, the ones that we have which will help in the implementation of the program. I just mentioned a few of the incentives and in cause of this our interview we have mentioned two of them, I just want you to like summarize them now | Ok, you mean the issue of taken the drug to the community. Is it part of it |
| It could be. May be I just mentioned the transport fare to the clients | The transport that motivate them to receive their treatment |
| No. this I mean this incentives are meant to help the implementation of the program so that whatever things we are doing, the implementers are doing; they are doing it well so that the decentralized DLTB service will be going on well. Is that clear? So that it will be going on well | You mean incentive to give or the one they have given to us, which one? |
| Yes the one that they have been given to you. The one that there already | Ye through the CBOs abi? |
| Yes | “Ehe”, I think the CBOs |
| Yes the CBOs | The CBOs, the have given the incentives to the patients |
| Ok | And there are doing well |
| Alright, any incentive to CBOs? Are the CBOs given any incentives | Yes |
| Good, that’s what I ehee… | Yes |
| Any incentives to the TBLS |  |
| Ok, so how do you think that your facility’s culture or setting affect decentralization of DRTB? | Facility setting? |
| mm… | Affect the |
| Implementation of this program |  |
| For instance in your office, in your facility there has to be a way of doing things there. You have been in TB program for a long… | Yes |
| There are things you do, you have earlier mentioned something like washing hands, and how you attend to you patients and the general culture or the setting of your facility for instance whether it helps to implement the program well or not? | The setting of my facility helps the program. |
| Ok, how? | Because where the patient will, where we will attend to the patient, I mean the setting |
| How? | There is a place that we attend to our patient, so that very place that is where we put our washing basin |
| So now all those things do they help to improve the implementation, mm  Do they help to improve the implementation? | It helps |
| How? How? Can you explain? | I think it make is easier for us to attend to our patients |
| That is good. How does it make it easy for you to attend to your patients? | The place is not congested |
| Ok, in that case, it helps you to implement this program? | Yes |
| Effectively | Effectively |
| Is alright, now I want to know what your motivation is for wanting to have or ensure that the program is implemented successfully. What motivation do you have? | Motivation |
| Yes | That I have, what motivate me abi |
| yes | Is you know I have passion for it |
| Is it for the programme or for | For the program general, even for doing |
| Why | These are my people |
| You mean the programme people are your people | I mean the patients |
| Ok your passion is because you want the patients to get better | Yes |
| You are happy when you see them get well | Get better, yes |
| Yes, is ok so your lust for your patients and the program helped you to get good treatment outcome | Ehe to get good treatment outcome, yes  Because the way the program is packed, I said it earlier, it helped and motivated our patients to receive their treatment in a place very close to them. So and most of them are feeling now. |
| It’s ok, |  |
| So how confidence are you now about been able to implement decentralized DRTB services regularly in your facility? | I have been trained. So I know what to do and what I should not. So there is no problem for me in taking care of this DRTB |
| How about your colleagues, how confidence are you? | They are |
| Why did you say that? | So whenever I did step down training for them, so they know what to do and what they should not do |
| And they are committed to | They are committed, very-very. honestly they are committed |
| Is ok, so now let’s look at how the program or the decentralized DRTB services delivery fits into your existing processes and practice in all office or facility as case may be | How it fits? |
| Yes, whether does it align well with what you use to do before now, before the program came on board? Is it so much of a burden to you | Mhuuu ok. Anywhere is a burden but at the same time we try as much as possible to cope because it is part of our job. |
| How is it a burden? | You know the issue of this spacemen request form, you fill-fill-fill, they have add something to it so but notwithstanding, we are trying our own best at least. |
| Because of the passion you have for the work you have to cope | Yes. |
| Ok are you saying that it fits well into your processes and practice? | It does. |
| Alright look at whether you think DRTB decentralization, I mean the decentralized DRTB service will replace or complement the current program. Whether it should replace it? | Replace it with what? |
| I mean whether the decentralized approach we are talking about should replace the former way of doing things? | No this decentralized is ok, I said it earlier is better than the former one we are using |
| So should we stop the former one now and be doing only decentralized of | Yes let’s continue with this decentralized |
| Ok and forget about the former one? | Yes |
| Why did you say that? | I have told you that it makes the work easier for us. For as many of us, you know for you to tell one of my patients that you are going to Ibadan, you are going to stay there for so so-so months it looks somehow to them but telling them you will just go there and do your test and come back to the community, you receive your treatment in the community, you know, so that one helped them. They will not run away. So they will come and take their treatment in the community and they can go back to their job |
| So what is your final word? | My final word is to continue with this decentralized program we are doing now. To me o, it’s ok. |
| Thank you very much | You are welcome sir. |

**KII/A19**

| **Interviewer/facilitator/questions** | **Respondent/responses** |
| --- | --- |
| Having explained the procedures and all the things that you need to know and answered our questions and obtained our consent I think we can now go on to the interview proper and I will start by asking you what you think about TB Reach Wave 9 interventions to decentralize DRTB services | TB Reach is a very good programme that really improve our DRTB work and Services in Akinyele local government in the sense that it creates more laboratories for our patients not to be moving far away before they will access their baseline test and we have two new laboratories more with the one existing before like Favour and Dakura laboratory. It really helped our patients not to go far before accessing their baseline test. |
| Alright, can you tell me what you like most about this interventions | What I like most is about this decentralization and investigation |
| Is it the decentralization of the baseline test to the local government | Laboratories to the local government level. It make it easier for us |
| What do you like the least about it? I mean about the programme | Is the transport support given to our patients when they are going for their baseline, it really helped them |
| I said what do you like the least | The least? |
| Yes. As in not that you don’t like it but in hierarchy in the order of importance it comes last | Okay. |
| Did you say is transport allowance or support given to the clients? | Yes the transport support given to the clients |
| Okay, why should it be? Why do you like it the least? | Because, you know most of them they will say they don’t have transport money and when you support them though the reason why I like it the least is because they accept it but they still complain that it will not meet up to the |
| Is not adding weight | “Eheeee” is not adding weight.Is not up to the standard but they still manage it so if they can increase it a bit |
| Is alright, what has affected your practise of decentralized DRTB services in your State? I mean what has helped you the most to practise or get involved in the practise of decentralized DRTB service | What helped us most is this support I have been mentioning it, is the support given to patients and this issue of GX alert given to us by your organization TB Wave 9 |
| Okay, are there the things that influenced your being a part of the decentralization process? That is something that influenced your practise of this approach of decentralization | Is the passion that we have for the patients |
| Okay that is what moved you | That is what moved me |
| Have you experienced any challenge or challenges so far in the implementation process | Yes. About the GS alert your organization need to improve because most of the time when we receive the GS alert the patients name will not be included even the facility name. what we normally saw was the Ref resistant has done so-so-so geneXpert side they will mention the expert side but no facility name no patient name so it should be difficult for us to track the patient so they should include the name and facility centre for more easier to do our work |
| Okay, thank you very much. I want us to look at some of the characteristics of the intervention now and we will start by comparing the decentralized DRTB services with the routine way of doing it that is the routine programme. So I want you to compare how, tell me how there are compared to each other | There is a little different from the existing one and this newly one because their stratum request form is a little bit filled up with the address, the date and every information that will make it easier for those laboratory personnel and the DOTs and the DTLs to trace the patients is inside that photo request form more than the formal one we have been using |
| Okay so can you talk more on the advantages that decentralized DRTB services | Another advantages is that we are you know before we just fill one form but now we are filling more forms even if one get lost they still have another extra to work on |
| Okay is it duplicates or? | Is a duplicate; before it was single form that we normally fill, now are filling like two. So if one is missing they will work on the other one. That one really helped us and it helped our work |
| Is okay. Are there disadvantages you have observed in the decentralized services delivery | I don’t really observe any disadvantage. The advantages of it are more. there don’t have any disadvantages |
| Okay. Now let’s look at the changes you need to make for the decentralized DRTB services to work effective in your facility. Changes, so what changes did you make, where there things put in place, any adjustment in your facility in other for the implementation of this approach we are talking about to be effective | We don’t really have need any changes than this programme to continue |
| What I’m saying is that at the introduction of this approach that is decentralized DRTB service delivery where there certain things they changed may be arranged in your place of work so that the implementation will be smooth | Okay, we normally offer our patients a place to sit. Then we give them like nose mask, hand sanitizer, most of time we do ask them to go and wash their hands before given them the health education |
| So before we started this decentralized DRTB services you were not doing these things | We suppose to do it but there is no enough PPE |
| Okay but now you have more | But now we have enough PPE |
| So can do those things now | With ease |
| You talked about where the patients stay, did you arrange the place for the patients to stay | We have shade |
| Okay like | We have shade. Is like the OPD |
| Okay, you tried to provide a place for | We normally provide a place for them to sit. Relax a bit before we come up to give them health education |
| So these are the changes you introduced | Yes |
| Okay, now let’s look at this component we have talked about them before but I want you, the component we have mentioning, all those components are there ones you think can be altered | There is no one because all, there are very important and it help our work. It help our services the more so you don’t need to alter any one |
| Or change any one? | No we are satisfied with it but about the transport support if they can help us to increase it a bit it would have been better. Transport for the patients |
| I want to know how complicated this decentralized DRTB service delivery is. Do you think is so complex or it is complicated to implement? Or is it simple? | Is simple |
| Why do you say that | It is simple now than before. Now this is a support from your organisation to patients even to we both the DOTs officer and the TBLS that are going to render the services for them. So the supports to the patients and to the DOTs officers or TBLs with the means of GX alert the communication allowance though it’s not every month or all the time; so once in a while we are receiving such. |
| Is okay. Now let’s look at how it meets the need of your patients so I want to ask you how well does decentralized DRTB service delivery meet the needs of your patients | Thank you sir, it meets their need a lot because before the commencement of your service they do complain that they cannot go too far-far place to go and do their baseline test. Most of them they will say they don’t have transport money to and do their baseline but now there is no complain again. They will do it at their various communities with ease so no delaying of result then they will still give them little support for transport though is still within their community but your organization are still supporting them so is advantage and is really help them a lot. So we don’t receive any complain of I cannot come or I cannot come from my place alone, is reduced than before |
| Alright. So what kind of incentives sorry. Let’s look at now you have talked about how it meets their needs let’s now know whether there have been a strong need to increase or reduce this service delivery in your facility. What I mean is has there been a need in your facility to either increase or reduce decentralized DRTB services | We want your organization to increase DRTB services in our local government because it really helped us. It helped out patients.We the service provider it make our work easier because since there are receiving support no more complain again then if you expand it a bit at least we will still have more laboratories where they can access their test again so that they won’t be going far-far place before accessing it |
| Okay, has it in anywhere affected lost in follow up may be by, has it increase it | No it has decrease. Our lost in follow up has decreased and those that are accessing their treatment they have increased it has jack it up than before |
| Okay, that seems to be some of the reasons why you need an increase | That is why we need an increase and want the programme to continue |
| Alright. So now what support are available to help you adopt decentralized DRTB service delivery in your office | We have been mentioning it like this transport support for the patients, then GS alert, then communication incentive for the TBLS though we want our DOT officer too if they can extend their hand to them it will be better so those are the areas |
| Okay, communication support? | Communication support. During the OPD clinic your organization do support our patients with transport money then when they are going for their baseline, they do support them again so but we want your organization to extend this communication support to our DOT officers so that you know this work you can’t do it all alone. If TBLS is not available the DOT officer is there to attend to the patients |
| How does the support of the patients for instance that you mentioned help you to adopt this approach which is decentralised DRTB service delivery, can you explain how it helps you. | Before we find this difficult in getting our patients to the laboratory centre but now with the support of your organization this issue of decentralization, it make it easier for us. No more stress again. When we call them, Okay go to this so so-so facility in your community do it, so they find it easier than before |
| And that has encouraged you | Eheit has encouraged us we the service provider in rendering our services to the patients |
| It’s okay thank you very much. Although we have talked one way or the other about the incentives now but I will like you to talk about just the different kinds of incentives may be to summarize it now because you have mentioned it in various ways. Those incentives that is we have been talking about. | Like support being given to the patients when they are going for their OPD meeting |
| Yes the transport support | transport support when they are going for their baseline test, most of the time the support given to all those TB survivors, so the communication allowance given to them, so they do call those that are about to access their treatment or those that are still on their treatment say okay we have experienced this thing before and we have come out of it with ease with love and with much care been given to us so it really helped our work. They speak on our behalf. So when the patients now come to our facility it make it easier for us they have been hearing this things before. Is not new again and people are really getting better, getting well out of DRTB |
| Okay thank you, well done. Let’s look at how your facility culture or setting affects the implementation of this service delivery. The setting or the culture, now in your health facility there are ways you do your things and such ways had, so we want to know how those ways that you do things whether it has anywhere improved the implementation or whether it has hindered it so to speak, whether it prevents you from implementing it as it should or whether it is useful | It was useful and it was enhanced our service to the patients your programmes so it doesn’t affect us or bring us down. It improved the way and manner we offer the services to the patients |
| How does it improve it? I want you to explain to me | It improves it because there are more forms. You know your organization provides more treatment forms then we have all this PPE, we have it plenty like nose mask, hand sanitizer |
| Does the programme provided or you already have it | Your organization add to it |
| Okay, added to it | Added to what is on ground before |
| But remember my question is the way you use to do your things which you can described as your culture or the setting may be setting of your office, the way things are there, whether those things for instance how you use to attend to people may be you usually when somebody comes in, you give the person a nose mask or you ask the person to go and wash hands or you take the person to a sitting place that is however you do things my question is the way you do your own things do they of did they have improve the implementation of this service delivery or where they imputing the implementation | Okay is improving |
| I said how? Which one for instance? Okay let me give you an example. That somebody says well I have always been in TB programme, usually when somebody comes you ask the person to wash their hands in a certain place may be when the programme came now, it was easy to accept the programme because and because you have been doing all those things it helps in implementing the programme properly. Can you in your own words now explain to me how the way you do your things has been able to help you in the implementation | This TB Reach Wave 9, since it has been come into an existence. It improve our work, is not affecting what we have been practising before, it improve it because what we are needed to render the services to the patients is available |
| Okay, but the way you have been doing things has it been improving the implementation of the programme | It improve, it improves it |
| Alright thank you very much |  |
| Okay, what has been your motivation to ensure that implantation is successful | My motivation? |
| Yes what has motivated you | Is the passion that I have for the patients and I have for this TB programme, is the passion that I have for it so that’s what motivate me in attending to my patient in rendering the service to the patients with ease |
| As in when you see you patients get well | Ehe when we see our patients getting well it motivates us a lot because most of the time when they come for the treatment most of them they might not be looking well but after the counselling health education, okay do this eat your balanced diet this and that so after some months we see that they are getting well, they are looking good and better that’s what motivate us, okay we should not relent we should keep on doing the good work |
| Okay, I want to know how confident of been able to implement this service delivery we have been talking about regularly in your facility | I have confident in doing it because we have been dealing with DS patients before so when it comes to the DRTB patients so it makes it easier because we have been trained for itand we are doing it regularly we are doing it almost everyday so we have use to it and the confidence is there,we are competent to do it. |
| How confident are you about your colleague also being able to implement the services | They are qualified the do it then because when we receive the training we trained them also and when we are rendering the service they are also there so we do it together |
| I want to ask you how the decentralized DRTB service delivery how it fits with your existing work processes and practises. The processes in your office and your practise does it I mean does the programme of this service delivery fit well, align with things you do or is it too much of burden or you find it challenging or time consuming that is what I mean does it stop or disturb your workprocess or it’s okay, it fits into since you have been doing drug sensitive TB treatmentthat this one now that is the DRTB it fits into it perfectly. | It fits in perfectly because we have been dealing with TB patients before then this DRTB it makes it easier for us because we have use to it be it counselling being to educate them on what they should eat, on what they should do and the precaution so it doesn’t affect us it makes us improve more and more. |
| Okay, does it affect your volume of work or your burden of work? | It doesn’t affect the burden |
| Do you fill more forms | We fill more forms, it doesn’t affect, you know we said it help us though we are filling more forms I have mentioned it earlier that we are filling more forms more than before but it improve our work because there are some columns that are not on the existing form but this present one, it’s more, it’s complex but we are used to it and we are enjoying it and it improve our work |
| Okay I want to ask you now whether you think that new method which is decentralized DRTB service should replace the current programme the routine programme that’s the way you are doing it before the centralized one or whether they should complement each other | They should complement each other, there should not stop it |
| Why? | We as a service provider we have been enjoying it |
| You enjoy the two programmes? | We enjoy it together because we are on existing protocol then with the new one are still in line. It should continue |
| Alright thank you. So what is your last word because we are gradually coming to an end | Okay last but not the least. So I want to say we really enjoyed the DRTB Reach Wave 9 services in our Local Government and it really helped our services so we want it to continue and we pray that God will continue to increase and enlarge their organization more and more in the Mighty name of Jesus. |
| Amen. | Amen. |

**KII/A20**

| **Interviewer/facilitator/questions** | **Respondent/responses** |
| --- | --- |
| What do you think of TB rich wave 9 interventions to decentralise DRTB services? | It is good, and people have being getting better, the services we have being rendering through TB wave 9 intervention. |
| You mean you get better treatment outcome? | Yes, we get better treatment outcome than before. |
| And your Patients, do you still have as much lost to follow up as before? | The lost to follow up has reduced; we no longer have lost to follow up again. |
| What do you like most about the idea of decentralising services? | It is better to decentralise the services. |
| What do you like most about the intervention? | It helps us in tracking our patients, unlike before. Before, we normally have lost to follow up, but since the intervention of the organization the lost to follow up has reduced drastically. |
| Is there something you don’t really like about the intervention or which one do you think should come last? | The intervention is Okay, all is ok to me. Engagement of state volunteers should come last. |
| You know we talked about decentralising treatment at the local government level, verifying the treatment tent using verbal autopsy, and counselling of the patients. | The one I like most is improving supports to patients for baseline investigations. |
| Why? | Because it will help us to place our patients on treatment at the normal time, immediately the results are out we should be able to place the patient on treatment if the result is ready. |
| You really like it well? | Yes |
| That still doesn’t answer the question of which one do you like least. May be before we will finish you will remember it, let continue. | ………………………. (silence) |
| What affected, or impacted your services of decentralised DRTB services? | Initially our patients use to come to state headquarters for treatment, but now with the help of Tb rich it is community base now, so initially some patients don’t like going out of their community to receive treatment, they use to feel as if they will not come back again. With the help of this decentralisation now, it makes them feel at home when taking their treatment unlike before when they go outside their community for treatment. The treatment is brought to their door steps. |
| How does that influence you? | It influences me in the sense that my patients take their drugs at their door step. |
| They now access care | Yes, they access care as it should. |
| How does that make you to be interested in what we are doing, or to be part of the programme? | They get better on time, and we normally give them home visiting, so they have sense of belonging. It influenced me to be part of the programme, and to practice DRTB. |
| Have you had any challenges while implementing this programme? | There is no any challenge. |
| Looking at the two programs, the decentralised program, and the routine (existing) program; Can you compare the two? | This TB rich decentralised DRTB service is better, because the treatment is taking to the door steps of the patients. Unlike the other one we have been practicing before, where patients needs to move out of their community to travel down to state for treatment, so this one is better. There is no need of going out of their community for any treatment to access care. |
| Is there any other thing you can say in comparison? | To the best of my knowledge, the decentralise DRTB services is the best. The patients are be given transport fair, and CBOs given tracking allowance, we do training too, and have liaison officer at the state at the level; they trained us on the new form given to us by the organization. |
| Are there disadvantages you can think of? | No |
| In your facility did you need to make any change for decentralise DRTB to be implemented? | There is no much changes needed in the facility, all because in our facility we don’t separate DRTB from normal process, though there is a particular area they normally sit, and we normally give them face mask to cover their mouth. |
| Did you have to introduce that particular place when you started this program? Did you have to make adjustment in your sitting? Is there any other thing you did? | There is adjustment in the sitting arrangement unlike before. We normally give sanitizers to our patients, and we give them face mask too, then there is a corner we arranged for veronica bucket for hand washing too. We made all this changes when the programme started. |
| Are there any of the component that you changed, or altered, or may be improved upon those components, we have been talking about. GX alert and patients now gets, the improved tracking pattern, counselling, there is also decentralised baseline investigation, so transport supports, USSD, decentralised treatment initiation to the local government level. There are investigation treatment tent using verbal autopsy, engagement of state liaison officers, all these things I listed now; which one do you think change, or modified?  How? | I can say that verbal autopsy should be modified. In the sense that, pre-treatment tent verbal autopsy should be modified. There are some patients that died, as it is one of the components. By the time we were asking the relatives what really happened, a times they normally feel as if you want to stigmatize them. |
| What do you think we should do to remove that problem? | We should try as much as possible to make sure that the laboratory for baseline that is not available is readily available at our LGA. |
| Tell us more on how you think we can avoid that stigma? | We should try as much as possible to educate the community at-large on the importance of early signs, and symptoms of cough, so that the patient can report to the health facility on time, and do the normal thing so that the sample will be taken to laboratory on time. Awareness creation is very important. |
| How complicated do you think this services are, decentralised DRTB services? Why do you say it is not difficult? | It is not difficult. It is not complex, because the decentralise service is being taken to the door steps of our patients, so there is nothing difficult about that. |
| Is it easy to implement, the forms you fill, and other roles? | It is easy to implement. They not cumbersome in the sense that I have an assistant, and all other health care workers to participate in given out drugs. Even if am not around they normally assist me to dispense drugs. I have a desk officer too that is assisting me in the facility. The work is not cumbersome. |
| How well does decentralise DRTB services meet the needs of your patience? | The decentralise DRTB services meets the needs of my patients in the sense that, like the issue of GS alert immediately the results is out we will be alerted, and even including the patient too. We normally get it with the help of the organization and we commence treatment immediately, or place them on baseline investigation before treatment. |
| Does given money meet their needs? | They are given them transport allowance, and CBOs for tracking allowance. There need, financial needs of the patients are being solved with the stipends given to them. |
| Does the counselling address their psychological needs? | It addresses their psychological needs, in the sense that some patients feel that anybody that is having TB, the patient cannot survive, but with the help of counselling their next actions are stress free. |
| Do you think that there is a strong need to increase, or decrease decentralise DRTB services? | There is need to increase, because the already analyse services has helped the patients a lot. So, with the efforts of TB rich, if there are something they can bring out that can easily help our patient we will be much happy. We need patients to be attended to more. |
| What support are available to you to adapt decentralised DRTB service delivery in your facility? | There are supports sir, like the issue of incentives that are being giving to the clients for transport, it use to motivate them to come back, and it makes our work easier, unlike some patients. If there is nothing to support them to come to the hospital, it will be difficult, there are people leaving in a far area that is not close to the hospital environment they need to be transported for them to access care. |
| Tracking is easier now? | Tracking patients is much easier now, it part of support too, and it makes our work easier. |
| Are you part of the WhatsApp group? | I am, and it very supportive. |
| Are there any other support you can talk about, the form that is given for you is it helpful, is it supportive? So it supportive? | Yes, it it supportive, because at times when we are less busy, I use to gather my general health workers, and train them on hospital requests form given to them by RIDHEC.  Yes, it is supportive. |
| What are the incentives that are available for the implementation of decentralised DRTB services? | As I have earlier said, they normally give the CBOs incentives for tracking the patients, patients are receiving transport fair too. |
| What about the TBLS, are they given any incentive like communication incentive? | Yes, they are given incentives. CBOs and TBLS are collecting incentives. |
| What about your facility culture, and setting, how does it help in the implementation of decentralisation of DRTB service delivery? | It improves it, in the sense that when the patient comes in, you welcome the patients, and ask about his family unlike then when the patients comes in all the health workers will be fidgeting as if they will be infected immediately DRTB patient comes in. with the help of TB rich now, things have changed totally through the training given to us. |
| Is there anyway your culture, or setting helped you in the implementation of decentralised DRTB Service delivery? How? | It is of help because there is no more stigmatization in the facility with our DRTB patients, Unlike before. It affects it positively. If the patients come now, we cover face mask. |
| Where you used to be given facemask? | Initially, we don’t give face mask, but now any patient that comes in we give facemask, with an introduction we make with the help of TB reach. The way we do things in the facility is helpful to the implementation of the programme. |
| What is your motivation? | What motivated me most is that on the issue of baseline is been given by TB reach, if not for that our patients will not be able to go for baseline test, but with the help of TB rich they have done a lot to motivate me. Like a patient that absconded before he was persuaded to go for baseline test, it was a thug of war. I persuaded her with the family before she can agree to go for the baseline. Despite the facts that money for the transport was not enough, it was TB Reach Wave 9 that added incentive to the money given already for the transport for baseline, and she went. When she came back, we placed her on treatment, and she took the drugs for just two weeks, and she absconded. I tried my possible best with my own money to look for her, despite the fact that her parent said that she has travelled to another state, I went there with my money because I know the implication of what she did, yet I couldn’t get her. |
| How is it a motivation to you? | It is a motivation, because if not for the money added by TB reach she wouldn’t had gone for the baseline test. The help that the TB rich, the little incentive that was added to the transport fair for her, made her to go for the baseline test. It is that acts that motivated me. |
| How confident are you about being able to implement decentralise DRTB services in your facility? | Yes, to the best of my knowledge, I will try my possible best as I have been doing earlier to make sure that all the DRTB patients takes their drugs as expected, since there are no more going out of their communities to get their drugs. The drugs are being giving to them at their door steps. |
| How about your colleagues, are you confident they can be able to implement the services? | Am very confident, because already they have been assisting me in given out drugs to my TB patients even to my DRTB patients, that makes me to believe that they can be able to implement the services. |
| Do you do step down training to them? | Yes, I do step down training, this also the reason why I have confident in them even, I normally do on the job training for them on the filling of request form every month because some of my general health care workers, when they are filling the request form, they may jump age that is why routine activity to do on the job training for them. |
| How well does DRTB services fits into the existing practices in your facility, does it increase the burden of work, is it time consuming? | It is not, the burden is not much. For me I don’t think there is a burden in as much as I have subordinates. It aligns well with what we do, it is also because I have been into TB management. for a very long time. It is not challenging |
| Do you think they should replace the routine programme with decentralise DRTB services or should they complement each other? | What we are doing now is better than what we are doing before. Decentralise DRTB services is better, and preferable because they access care at the community level unlike travelling, and leave their families for two to three weeks before coming back. |
| Is there any other suggestions, or recommendations? | To the best of my knowledge the other suggestion or recommendation I can give to the intervention is that there is no laboratory in my LGA, GeneXpert machine is not available, is like they brought used one the inverter, and the battery is not working, so samples is being taking to another L.G.A for processing. If TB Reach Wave 9 can bring another GeneXpert machine, we will be very happy |
| Thank you very much for your time. | Thank you sir. |

**Title of Interview/discussion: KII BI**

**Date of interview: Monday, 7^th^ August, 2023.**

| **Interviewer/facilitator/questions** | **Respondent/responses** |
| --- | --- |
| **Good afternoon sir.** | **Good afternoon.** |
| On the overall, what do you think of TB REACH WAVE 9 intervention which focus was to decentralized DRTB services? | Yea, it was a good concept and still a good concept because of the ease of accessing medication from our patients logistically actually. |
| Any other thing? | Well, I want to believe it also help to improve our compliance in terms of accessibility to OPD side. |
| Okay, so what do you like the most in that decentralization process? | The interaction, patients-counsellor, patients-pharmacists interaction because from there you can generate our pharmacistular reports. |
| Okay, that is the biggest catch to it. What could be the least aspect of that concept? | The least? What could be the disadvantages of the concept? |
| Yes, or things that could have been not so wonderful as these ones you have mentioned. | I wouldn’t really say there is something about that. |
| Okay, but we are just talking about the concept generally; so everything about the concept is wonderful? | Yes, it is okay; the concept is okay. The other thing is the kind of palliatives to patients were not enough. |
| They weren’t adequate? | Yes, initially they were but now with the high cost of things and also logistically, the logistic team were not even gets through. |
| Logistic team in the concept of DRTB decentralization? | Yes, the support was almost coming from federal level. |
| Okay, what could you have advocated? | Well, I think there should have been kind of partner to give more supports to the logistic unit. |
| So, what you are saying is that the logistic units are not adequately taken care of in that process? | Yes. |
| Okay; okay. So, what affected or influenced your practice of decentralized DRTB services? | What influenced? |
| Yes, what influenced it or what influenced your practice of it? | Yea, it was the accessibility to medication and services by patients from the hither-lands, going to the centre was a bit cumbersome; that is, it finds a way of getting things closer to them; that kind of thing you know can lead to treatment failure seriously; if patients want to get services or medications and they don’t have way with that; and ofcourse you know what that means; they will treatment failures, service failures and so many things. |
| Okay, so what has helped you the most in this decentralization process? | “Long pause” |
| What has helped you the most in the practice of this decentralization? | It assists us to get the drugs available to the patient at OPD on one point and from there we generate our earlier services and data. |
| Okay, then what challenge did you passed through; or the challenges that confronted you in that process? | “Long pause” |
| Any challenge? | Well, sometimes it was just temporarily actually but the temporarily thing was sequential, that is the availability of materials for PEP; I mean for self protections such as face mask, sometimes no gloves. |
| So, those were the challenges? | Yes. |
| Okay, let me go down to the nitty-gritty; so, does the decentralized DRTB services compare with the routine existing programs? | I think DR-TB decentralized is better. |
| In what way? | It is better in terms of; like I said initially “ease of accessibility of service to medication” |
| What did that help to achieve?  Did that help to achieve anything? | Positive outcomes. |
| Okay; what are the advantages of decentralized DRTB services? | Like I said more patients been enrolled.  Less non-compliance.  More patients taking up medications.  Turning up the services.  Fellow up and all that. |
| Okay, any disadvantage of decentralization? | Well, disadvantages; I wouldn’t know. |
| You think there were no disadvantages at all with the project? | I wouldn’t want to look at the disadvantages; I can’t pin-point some; I think the positive outweighs the disadvantages. |
| Okay, so what kind of changes did you want to make for decentralized DRTB services to work effective? | More funding. |
| No, what did you made for the effectiveness of the project. | What I made? |
| Yes. | Actually, I made sure that the drugs were actually packaged and also ensure the drugs get to the number of patients that were supposedly enrolled and then get the actually number of patients that received drugs TB.  Then also I interviewed the patients one-on-one to know if they have any reactions from the drugs; you know these things can also discourage patients’ compliance. |
| Okay, so those were the changes you made? | Yes. |
| Any other one? | “Long pause”  That was just that. |
| Okay.  Are there components of the interventions (decentralized DRTB services) that should not be altered? | “Long pause” |
| Okay, I will mention the interventions so that it will help us in that one.  Number one intervention is devolving of GX alert.  Number two intervention is improving tracking of patients by supporting the global funded Community Organization.  Number three is improving counselling of patients through introduction of structured free treatment-counselling and team counselling to DRTB survivors  .  Number four is decentralized Base-line Investigations.  Number five is providing transport support to patients for Base-line Investigations.  Number six is employing mobile connectivity solutions to improve turnaround time for Base-line Investigations.  Number seven is verifying reported deaths using verbal autopsy.  Number eight is decentralizing treatment initiation to the Local Government Level.  Number nine is engagement of a State Volunteer Liaison Officer to coordinate state wide activities | I think they are still okay. |
| Which one of them would you want to remain the way they are for now?  For the intervention; during that intervention because the intervention has ended? | From what you have enumerated; I think they are okay. |
| So all of them should remain? | Yes. |
| Is there anyone you are ranking higher than other? | Apart from the state liaison issue; I think we should have more than one. |
| We should have more than one liaison officer for the state? | Yes sir. |
| You mean that one should stay? | No; all the rest should stay but there should be improvement on the number of liaison officers from state. |
| Okay, I have taken note of that one.  How complicated is the decentralized DRTB service delivery? | Complicated? |
| Yes; Is it complicated? | I think the way it is structured is okay; although I am not actually deep in the programmatic running of it; where I am mostly is in logistics; for the program I think the program manager should speak better on that. |
| Okay, how well do decentralized DRTB services meet the needs of your patients? | Yea, I think from the feedback we get; it is okay because they don’t miss their medications and also the service delivery; like I said maybe they have personal challenge for them to access the OPD sites; outside that I think it is okay for them. Apart from that you know we have good network in Akwa-Ibom State, so you can always get to wherever you want to go even when it was at central level but now it has even got closer to us; I don’t see any challenge for them to access services. |
| Okay, so you are saying that it made them ease especially on access to medication and services. | Yes. |
| Any other thing? | I think that is that; apart from like as I said I don’t know how much that is been given to them; those patients coming in for these services but the way things are; I would want to suggest it should be increased. |
| Okay, so in your state now has been a strong need to reduce or increase decentralization of DRTB services? | Well, in Akwa-Ibom State, usually we have some big Local Government Areas bigger than others; but for now, we have four zones for this decentralization; it is only if the programs look down and see Local Government is big; you get what I am saying? |
| Yea; yea. | Even getting to their points of OPD is always a problem; so, there is for more decentralization of those areas but that will be based on the number patients’ turnover on DR-TB activities from that locality; otherwise the four can remain. |
| Okay, what supports are available to help you to adopt the decentralized DRTB services? | Logistically no; apart from the one from the Federal Government. |
| What is one from the Federal Government? | Well, the payment to take drugs to local governments; yes on OPD days you take to the centre but on other days you have to take it to different Local Government Areas; personally the ways things are now; it is totally inadequate. |
| Meaning that the supports are not enough? | It is not enough and we were not even having partners for the support. |
| What do you advocate then? | That they should be some intervention to support the logistic unit on service deliver and also on the day of OPD let there be lunch. |
| Okay, so that is what you are advocating? | Yes, and also, I mean sometimes drug become scarce in the store; yes, sometimes we run out of the drugs and we to get to the zonal stores to get them; you understand? |
| Yes. | Sometimes we run to the zonal stores for the drugs especially when OPD is coming and you need to get the drugs for the patients. |
| So, what kinds of incentives are there to ensure the implementation of decentralized DRTB services? | We, for us there is none so far. |
| Do you advocate? | Yes, financial supports. |
| Okay; okay, how do you think your state culture or setting in the state has affected either to improve or reduce the improvement on implementation of decentralized DRTB services? | “Long pause” |
| Does the setting in the state support decentralization of DRTB services? Or does it work against the decentralization? | No, it supports; it is seriously in support of the decentralization. |
| In what way sir? | The location of the Local Governments; it is usually on geo-political zones; you know we have at the national level; at the state level we have also; so and health wise, you can’t rule out politics. So taking these things to different zones; I think it is good; so normally we say “I am marginalized or my patients are not been taken care of or things like that” |
| So, are you saying that the state setting supports the decentralization process? | Yes, it is okay. |
| The working practices in the state; did it affect the decentralization? | It supports. |
| In what ways did it support? | Let me get you again? |
| In what ways did the setting the state; maybe the geographical setting in the state; in what ways did it support the decentralization process? | “long pause”  Well, because in the different zones of the state; like we have Oron zone, Ikot Ekpeneg zone; we have very fine hospitals there that; in fact, there are almost like tertiary hospital because they have doctors, nurses, midwives and all that; so we have to push cases for increase medical supports from doctors and nurses. |
| So, it helps? | Yes. |
| What has been your motivation for wanting to help ensure the implementation of the decentralized DRTB health care and treatment services? | My motivation? |
| Yes. | It is just to make sure patients get access to medications as at when due; that there will be no delay in the process we have compliance; we have better uptake of services and medications at the end on side of the patients. |
| That is your own motivation? | Yes. |
| How confident are you about decentralized DR-TB services in the state? | We have been doing it; just that we have to improve for any little challenge; we have been doing it and we are fine. |
| That means the state has been in favour of the decentralization for a long time? | Yes. |
| So, how confident are you that your colleagues are taking up that position too towards the decentralization? | Well, in every situation, there must be apathy; there must be apathy to issues; even not all doctors are interested in TB services not even DRTB services; when they listen to what it can lead to and all that; but we need to motivate; we need to encourage; you are not going to beat anybody; you have to sometime appeal to their sense of duty; the Hippocratic boldness; pharmacists’ bold; nurses’ bold and whatever that this thing has to be done; and you know that this is all about wash my hands let me wash your own; they think that in a project like this; there is so much to eat from it; so sometime we give something to them for motivation. |
| Which people do you have in mind sir? | The health officers; the people at the facility level because at the end of the day they are the ones to spend more time with patients doing medication and administration, service registration and all that. |
| How well does decentralized DRTB services fit with your existing work processes? | Well, it is not as if we take it as an adhoc service; it is continues as per the work I am supposed to do; so treat it holistically just like other services. |
| Okay. | Yes; it is a daily thing; it is a regular job schedule. |
| So, you are seeing it as job you are supposed to do? | Yes because the government is paying for my services as a pharmacist; so it is part of the job schedule, yes. |
| Okay, so with the decentralized; what do advocate that with the DRTB decentralized services will compliment with existing routine services that you have been having? | It compliments and improves it. |
| So, what are you advocating; does it replace or it remains to complement? | It compliments because if you are talking of replacement; it means it is taking another, replacing something that was not in the system; these things were there but it compliments them. |
| Okay, we have already done the interventions; you are just particular about the liaison officer otherwise... | The other ones are perfectly okay. |
| So, is there any other one you need to comment on? | The liaison of a thing is it not for the entire state? |
| Yes. | If you are decentralizing to zones, there should be zonal liaison officers to be able to coordinate what is happening in their areas of operations; that makes it a bit robust than one person coordinating the entire state; take for example; one person should be at Ikot Ekpeneg zone now and focus on the area; there will other people in the zone looking for treatment failures after initiation and all that but if I am in the entire state the performance will not be optimal because the work will be too much. |
| Is there any other thing you need to comment? | Well, just that you people should assist logistic unit getting some of the materials that we need; actually, because most of our things come from national, sometimes from IHP and none from other organizations. |
| Okay, and you feel that their impact should be felt? | Yes sir. |
| Okay; thank you very much. | You are welcome. |

**Title of Interview/discussion: KII/B2**

**Date of interview:08/08/23**

| **Interviewer/facilitator/questions** | **Respondent/responses** |
| --- | --- |
| What do you think of DR-TB wave 9 intervention which was aimed at decentralizing DRTB Services? | Okay, TB wave 9 intervention was a good intervention to Akwa Ibom State and to decentralize our lab is a good thing because for now we have four OPD sites. We have one standalone Lab and for baseline test we have about………… (paused)  We had two, it was in quarter 2 that we are trying to have another one. So, if we have more, ifwe decentralize to have more labs. It will really help especially those patients that are far that they have to pay much before coming to where we have Lab for their baseline test. So, if we can have a lab that is very close to them that they can just walk-in or they pay a little amount to get to that Lab it will really help. |
| Okay, what could you call as the best or what do you like best or most of the whole intervention in that DRTB wave 9 project? | What I really like most or that impress me was the aspect of choosing of DOT Officers in terms of writing accurate prescriptive address in the request form. That’s is the aspect of DOT Officers because the aspect of DR-TB what I really like most is the aspect of prompt alert the SLO will intimate the TBLS and other Team members to be able to contact the DR-TB patient on time when we received the alert and inform that so-so person in your LGA is DR-TB patient. So, this person has to go for baseline test so that was the real something that really- really impress me during the wave 9 intervention. |
| Is there any other thing that you did not like about the project? Is there any aspect of the project that you did not like or it wasn’t so wonderful? | Anywhere, in all projects we have ups and down, for wave 9, I don’t really see ……………….. they tried.The only thing was for us to work with them so we have a result. |
| So, what really affected or influence your practise in supporting this decentralization of DRTB care services in the state? What is it that helped you or that motivated you or influenced you that you supported the project? | Okay, as a CBO working in the Community, implementing wave 9 intervention coming to work,what really influenced me that I really keyed into the programme was the aspect of getting to the patient, receiving the diagnoses alert, the TB alert and other partners. So with that since we have a time frame duration that we had to after diagnose we had to enrol the patient, we have timeframe so that aspect that they were coming in to help us as a CBO sometimes it was difficult for them to come in and help, that really impress me that I was able to key into the programme and also from the beginning of the programme as a CBO we were involved because even in the training of the survivors we were there and they told us our own contribution that will help the programme to be successful. So, through that we were able to work with the team to ensure the implementation was a huge success. |
| So, what helped you moving well in supporting the project? What helped so much or what was the major thing that made you to support the project? | Okay, like I said before, it was involvement of the CBO from the inception, my Madam from day one that she came into the State and Program manager make sure that we have a CBO that is working with the DR-TB patient in the community that manages the DR-TB patients in the community and she invited us we were there from the beginning of the training and being that we in the community so we had to key in and ensure that the programme was successful. |
| Ok, did you have any challenges? Did you face any challenges in your involvement in that decentralization project? | We did not really have challenges. The only challenges we had was working with the survivors because,we picked survivors in all the senatorial districts of Akwa Ibom State we supposed to be calling them to counsel newly diagnose DR-TB patient but sometimes being that they live in the community we may try to reach them, their number may be switched off, not reachable for some days and because of that that part wasn’t really really effective to some senatorial district but in some it was really really effective, so I will say that that was a challenge that we had in that aspect but in this decentralization of Lab, since we had about four centres for OPD and at the beginning we had one for baseline when wave 9 came we had another one in Ikot Ekpene and before the end of the programme we had another one in Eket so there was no challenge, the only challenge was that you know some of these patients, where they live was very far from the lab so the challenge will be may be that they don’t have transport to come and the little money ARFH is supporting them with which N3500 may not be enough for them to come out to the Lab for their baseline test. So those were some of the challenges that we had. |
| You mentioned ARF, what is ARF? | Okay, Association for Reproductive and Family health, they are the one supporting Angels Home for Babiesin management of the DLTB patients in the Community. |
| What is their support like? | They support the patient for transportation during their baseline test, they also support them with social support where the patient can use the money and buy anything that they feel like buying. They also support the CBO, ok we have what we call treatment supporter for each DR-TB patient where they also support. They also support the CBO to visit this patient monthly, quarterly to know the way about of these patients, how they take their medication, if harking to their medication. So that’s what ARF do |
| Ok, thank you. So how does decentralizedDRTB services compare with the formal method where by everybody was coming may be to Uyo or the state capital to come and receive services? How do you compare the two? | I think having many lab now is better than when we had one because let’s say for example we have a patient at Mbo or let me say Isinobolo, Isinobolo I will say is the extreme when we talk of location of the LGAs in Akwa Ibom State and from Isinobolo toUyo will take at least one person to and fro about N6000 to N7000 ok so if the TBL and the patient are coming to Uyo that means they will spend may be up to N14,000 to and fro but having a lab at Eket now will reduce the cost. Soif I compare what we had before and where we are now or what we have now,I will say decentralization of lab is very veryok, it’s good. Because at least it will reduce cost for patient and supervisor. |
| Ok any other thing? Is it only that? | Accessibility, now the proximity between the LGA to the lab is also a good thing and what else, before we were having OPD in the four sites which was closer to Isinobolo because someone from Isinobolo cannot come to Uyo for OPD rather that person will go to Eket since Eket is closer to Isinobolo than Uyo. |
| Ok, so the services are closer to the people? | Yes, except you want to expand the OPD sites now to may be 6,8 or so |
| So do you support that? | It will be a little… what will I use…, because you know, since we have one lab now that is doing follow up test, so now I’m just thinking if we decentralize OPD may be we will use the lab that is available in that location for a follow up test because if we are to still use the one lab that is still doing follow up test it will be so so hard for that person to move may be we have three centres in a day or so but it’s ok if the programme can support that. |
| So that means you’re in support of further decentralization? | Of OPD sites? |
| Yes | Oh yes, I will say yes. |
| What do think is the advantages of decentralized DRTB services? | The advantages one:   1. It will help quick access to services. 2. It will reduce cost |
| Cost on the part of the patients or? | Cost on the part of the patient and it also help our patient in terms of patient will tell you I don’t have transport to go and get my drugs or because thisso so money you use to give to me I use it to eat, I don`t have transport money. So I think those are some of the advantages that will really help the patient. Even may bewe engage some labs that are around the area in terms of each visit for, may be for patient to collect his/her drug or may we do follow up test. Is one of the things I think it will also help. Instead of waiting for OPDS because you know sometimes this patient may have complains and if they don’t have access to a doctor they will wait for OPD and before OPD, we don’t know what would have happen to the patient. |
| Ok, so any other advantage? Any other advantage? | No for now |
| Ok, then any disadvantage of decentralization process? | The only disadvantage that I will talk about will be the aspect of the Lab now knowing what to do and on time, because if we engage a lab that doesn’t have all these equipment that we use in doing all these tests, it will be a problem. In that a patient can commence treatment without doing all the necessary baseline tests. So, we must look for a lab that has all the things that we are needed for those baseline tests so that it won’t be like a patient will pay his/her way to a lab today and they say I will do fasting blood sugar today, tomorrow come back for your x-ray or come back for another test. So, we engage Lab that will do all the test at once so it won’t be at lost to the patient or the supervisor because some of these patients, if they go today, tomorrow they will tell you they don’t have transport again, so I will say that, that’s the only disadvantage we had we don’t engage a lab that will give us services that we are need or may be...I think that’s the only advantage I have for now |
| The only disadvantage now, I think is the disadvantage that we are talking about | Yes is the disadvantage |
| Okay, any other one apart from the lab being unable to do the test? Any other one? | I don’t think of any other one for now |
| What changes did you make for the success of this decentralized DRTB Services?  What changes did you make either to your work pattern or to that of the Agency or the organization?  Did you adapt anywhere or was everything just the same? | I don’t really understand that question. |
| Ok, the question is this - is there any adjustment your organization made in other to ensure that this decentralized DR-TB Services worked very well? | We did not really do adjustment rather we key into the programme. Yes, we key into the programme. We go to OPD site on the days that are fixed and we worked with the team to ensure…  The only adjustment we did is during the baseline when we, in a component of our implementation we did not have a counselling section but through wave 9 intervention we were able to follow up with the patients through counselling, pre-enrolment counselling where wave 9 project helped us give us materials that we used during that pre-enrolment counselling where we could not go, may be is very far, we do that during the OPD service and we ensured we do pre-enrolment counselling before commencement of drugs. So that was, I will say that was the way we adjusted our own into the programme. |
| Ok, So Who was doing the counselling, is it the one by…. which group were doing the counselling you were talking about? | Ok, the Angels Home for Babies Staff used to go to baseline site and counsel the DR-TB patient before enrolment. The counselling was on side effect of the drugs, adherence and being consistent. |
| So, the counselling you are talking about is different from the one by DRTB survivors | Is still part that’s why I’m saying where the survivorswere not able to reach, the CBO now key into the programme to help. |
| Where you people doing such counselling before the wave 9 project? | No, we were not doing. It was when the wave 9 |
| So, you people added counselling into your own services? | Yes |
| Ok, that’s good |  |
| Are there components of this DR-TB intervention that you want them to remain as there are continuously? Can you remember the components? You know there is a GX alert, there is decentralization of Lab, there is this counselling by DR-TB survivors, there is payment to collect baseline investigations, then there is another one, I can remember all of them. | Transport for social support |
| Good, which one will you want to remain as like that without alteration? | Ok, thank you very much sir, like you talked about GX alert, that one should be there because without that we will not be able to know which and when the patent is diagnosed because I have been asking my colleague since wave 9 project ended, there was a group that we had that we normally have those information. I was even telling the DRTB focal person that, is it that you don’t DRTB test again or you don’t have DRTB cases again or is it because wave 9 project is no more so we are not using the … again people are not posting again. I don’t know, so that should be there.  The transportation for baseline should also be there but looking at the amount is very little compare to the high cost of fuelnow. Also, the baseline test. Before now we use to do audiometry but after a while that was removed from the programme so if we can bring that in. |
| You want audiometry to be back? | Yes |
| Why? | Because some this patient, they use have earing problem and when we don’t do that test we don’t know the effect at the beginning, we don’t know what it can cause so if we can bring that back into the programme it will help and also social support should also be there but we should also look for an increment because of as far as DRTB is concern, out of 100% of DRTB cases that we have, 70 to 80% are people of the poor not the rich so sometime these people are those that could not even afford a square meal to eat so some of them self like the one we visited one time, if you see where this person live in short you shed tears for this person. so that should alsobe there. Counselling because they need counselling a lot but for now the counselling shouldn’t be the pre-enrolment counselling, it should be intermediate may be quarterly, bi- quarterly I mean semesterly or so. So, we will be able to encourage this patient. So that it won’t be as if when you just tell them,they will just, It will be only one. It should be a long term something. Since we have now to eleven months of treatment so if we can make it three times during the duration of the treatment, I think it will help. Then e… |
| The use of liaison officer | Yes, is a very good thing because she was working and but I think one is not enough depending on the number of patients we have in the state. Because like Akwa Ibom we have 3 Senatorial Districts so sometimes when you want to do TB, we also consider the three Senatorial District, So, if we can have two in a state is not bad. So that this one can do this, this one can do this other one |
| Is there any other one that you think it should continue? | OPDs, morning OPD |
| There’s one that concerns CBO, you didn’t even say that. | I have talked about counselling which CBO was involved. You know since the project wasn’t for CBOs we were just keying into the programme for the success because the CBO. because we are working with DRTB patient so we cannot work outside the programme. So,If they engage CBOs to also work we will do more like in terms of visit to the DRTB Patient yes we have the money that now if you want to go monthly it won’t carry. So if we can have more support that will go may be we engage people in each Senatorial District to visit this patient do this do this, it will help. Because that really help sometimes especially when you visit a patient at the beginning of the treatment like one we had at Eket, the girl had to drop out of school, they chased her away. She was living in the church. It was because we visited her on time and we were able to be calling, visit her every time that’s when she was able to come out of the depression because she was depressed. So that also will help. |
| Ok the one that concerned the CBOs is the one they said improving tracking of the patients | Yes and follow up. |
| Should that one continued? | Yes. Then counselling as well |
| Ok, is alright. Then decentralized DRTB treatment service do you think is complicated? | Is not complicated, the only aspect I will say that look like is the aspect of how we try to fixed our OPD site. May be now we use to have it two days in four centres. So now may be we will try to extend the number of days because if you want it to happen 2days in three centres or in four centres it will not help because before you will get to Eket from Eket now let’s say we have Eket and we have may be another 2 at that axis before leaving there to this other place, to this other place, the patient might have gone home so we look for may be 3days or we have more team that this can move to this and this people can move to this so that we can be able to accommodate all the sites. Ok, but in terms of baseline, is not complicated because if it is something that you can just walk to a site and do your baseline or you pay a little amount it will really help so is not complicated. |
| So how well does decentralized DRTB services meets the need of your patient? | How well? |
| Yes | I will say this decentralization of Lab really help the patient like I was talking about the transportation, transportation now looking at the country we are and the amount of transportation we are spending every day, it will really-really meet well for a patient. When a patient is diagnosed and you tell the patient you can have your services done there or you can just pay N100 to N200 to do your baseline test. The person will not tell you I don’t have money. The person can even borrow N200 to pay his/her way there but when you now mention a place that is very far, the patient will even the relative will say do want to go and sell my person. So, it will really meantwell for my patients. It will, it will. So, the decentralization of lab will really help. The only thing is that we have to structure it in a way that it will really, like I was talking about the labs that we have to involved. We have to structure it in a way that the lab we will involve will be on the know of what and what not. |
| Ok. Any other thing on that? | mm and the doctors if we decentralize OPD site, we will have to engage more doctors that will be at that site and we have to train them so they will know how to check these patients, know when to stop intensive, continuation and other how to manage the patient. |
| So we are coming into almost what we were saying in the first time, is there need to further decentralize or reduce the decentralization? Which one do you support to further decentralize or to reduce decentralization? | I will say to further decentralize will help. Me on the aspect of the things I mentioned if we will consider those things, decentralization of this Lab will be good. |
| Why? | Ok, like I talked about access to the lab. Yes, because my patient will be if they are diagnosed on time, we have 42 to 72 hours from diagnose and baseline. So, if decentralize lab and we have patient in that area we will meet up that time frame. So, decentralization of lab, I will say it’s ok to go ahead with it but we should structure it that it will favour every one. Because if we say we should reduce from what we have now, looking at the transportation cost, that’s the aspect I’m looking at now. The transportation cost, if we reduced, we still have to look for a way to increase transportation cost for the patient and the supervisor because this patient even when you ask them to come alone, they won’t come. Because some of them they will say I have not been to Uyo before. So, coming to Uyo I don’t want to miss my way or something so the supervisor will still have to accompany this patient and because of that even the N3,500 that ARFH support for transportation now is not enough. So, if we reduce it now, we will, that money in short, I don’t know how we are going to work with that. |
| Ok. That’s good. So what support, we are coming to support now, I know you have been mentioning them. What support have been able to help you to adapt or adopt decimalized DRTB services | Ok, like I was talking about transportation, the support like now that we are working for ARF in terms of transportation to OPD sites, we did not empower on that but from all the money we use in management we also bring out some to pay our way there. So, if we now decentralize, we will need more support to also move around those sites during OPDs. In the aspect of patients they have support like I said is N1000 for them during OPD. So now in short that money is not enough. So if there is a way we could help to increase the money for them like I was talking about social support is N2000. In a month they have N3,000 from ARF which this N3000 like I ask one when we visited house in Ora, I asked him, they paid you N9,000 for the quarter, have you removed your transportation for the three months? He said ma, my house rent was due for over six months and I was unable to pay, so when the money is paid, I used it to pay my house rent. So now coming to OPD may be a problem. So, you know before now they have been paying some of them N15,000 monthly. That was when we were doing injected on.  So now is N3,000 so if that money can be, if we can increase a little, it will help.  Doctors I know KNCV support doctors at their site yes if you still want to support more is still ok. |
| So, for the CBOs is it only ARFH that support your activities? | Yes, is only ARFH that support our activities.So, if we have other groups coming in to supportuswe do more like I talked about in terms of home visit, we increase the number of home visitswe do throughout the period of treatment |
| Ok but you do home visits now? | Yes, we do home visits to the DRTB patients |
| Once a month, once a quarter? | No, at least two times a quarter depending on the distance |
| Ok, any support, what about incentive, what are the incentives that are available? | Ok, for the CBO we have incentive during intensive phase and during continuation phaseof the treatment so ARFH pays us everything like fifteen-fifteen thousand when we manage a patient during intensive when we manage a patient during continuation and like you know we don't have salary everything is imbedded in that money, office running cost and everything. So that’s the only incentive we have as a CBO, there is no other incentive that we have. So, if we have more we do more. |
| So even incentive is still ARF that support? | Yes,its still ARF that support |
| How do you think that your setting, the setting of your organization or its functionality? Did itimprove or reduce the implementation of this project, I’m now talking about your CBO? The working formula of your CBO, did it improve or reduce the decentralization of this process? | Please sir, explain. |
| Ok now, how your CBO functions, how your organization functions, you say you fitted in into the DR-TB project that is decentralization, what I’m now saying is, everything about your organization did it help to improve this decentralization or even hindered the process | No, it helps to improve |
| In what ways? | It helps to improve because as an organization, we are working with the setting, working with other groups where we also be part of looking at the lab we think will be ok for the programme or decentralization of lab and also contribute our quota to the programme. So, it really helped. Is not reducing, is improving. |
| Okay, is there specific way you will point out that it helped you to improve? Are specific angles that it helped? | Is finished sir. |
| Okay, let’s go to the next question |  |
| So, what are have been your own motivation for all the support you’ve given for the implementation of decentralization of project? | My own motivation? |
| Yes, what motivated you to give out this support | Ok, like I said before, in DRTB project you have to consider the patient. So,what motivate me is looking at the patient and trying to see how we can help them and when we don’t help them, they don’t know anywhere that they will get this help. So what motivate me was the aspect of seeing a way all these patients can be…, can help access quality services and on time. So, looking at that aspect I will say that’s what motivates me to key into the programme and ensure is successful. Because the DRTB is more or less patient programme because without the patient we will not do anything. |
| Thank you |  |
| So how confident are you that this project. How confident are you about being able to sustain this DRTB decentralization process | How confidence am I that the decentralization should be sustained. In a ratio of or in a percentage of how many, I will say I’m confident it should be sustained in a way that. |
| What is giving you that confidence? | Because we achieved more when we started and where we are now, we are still achieving so I know if we maintain the decentralization of lab we will also achieve more in the aspect of access to their drugs, access to doctors, access to may be OPD sites, access to baseline investigations. So if that is sustained, all these services I had mentioned we will be able to have it on time. So I’m confident. |
| What about the other members of your team, how confident are you that they will consistently support this decentralization project? | They have been supporting so I know they still support because if they don’t support, because the patient is what we are looking at. If you don’t consider the patient first, that means you are not doing anything. I’m confident that they will support and adapt to the decentralization of lab. |
| Okay. How well does this or did this decentralized DRTB services fit with your existing work processes and practise? | How well? |
| Did the decentralization fit into your own work processes and practice? We are now talking about your organization again. | Ok, sir, how well does my organization fit into the… |
| Yes, the procedures and processes of your organization, how well does it fit into the decentralization process? | As Angels Home for Babies, working with DRTB patients,because we are working in the community, we have to throw our policy as an organization may not really, anyway as Angels Home for Babies we support decentralization of the lab because is part of our work to ensure quality services are given to DRTB patient |
| Ok, you didn’t describe how now? Did you people care for everybody that… | Because why I said so is since we been working as ATM now is TB so we have to also ensure quality services are given by ensuring patients access these services and on time. |
| so from your own opinion with decentralized DR-TB services, will need to replace or complement, is it going to replace or complement the already existing services that not decentralized? | Is it going to… |
| Now we have this project not decentralized, what in your own mind, is it that we should all adapt to decentralized or make it move at the same pace with the one that is not decentralized, that centralized now. That’s what it means. Do you like the one that everybody come to Uyo to do test. You want it to be replaced with this new approach now | Is this new approach oo that we are talking about that we will decentralize the lab. Not the one we have before. Like I have been saying that we should also consider the structure who is involved in the decentralization, the lab, we should consider the lab we are using but to decentralize is ok. I was looking at the transportation aspect when I was discussing that that is a very key when we are talking about whether we should be where we are or whether we should support this decentralize session, because the transportation cost, if we say we should maintain where we are now, the patient may find it difficult to come to Uyo to access or may be from like we have it Itumekpo to Ikot- Ekpene now is also very far distance, so I will say we maintain the decentralization of Lab. |
| Ok,So you don’t want the centralized process to continue any more where everybody was coming to Uyo to do one thing or the other for DRTB services. | Yes  No |
| So, you want it to replace it automatically? | Yes |
| Is there any other thing that you may want to say about the decentralization of DR-TB services? | Ok, is still on what we were discussing about that when we are looking at the decentralization of lab, we should also consider if we are decentralizing OPD site the doctors at the site, we should also consider the team going to the site, the movement, we will have motive, we have the doctors consider the lab that are involved and also what we are doing now, the pharmacist is always with us. So all the team members that are supposed to be with the team should be there in case of anything we will not be found wanting in anywhere. So in terms of that training, we should also consider training. I know Eket what they do, one thing that really-really impress me is the aspect of their training. They do trainings a lot, for DOT officers, the TBLS and others. So, I know when we do all these things, we consider training as well because is the key. Because when people are not informed, they are deformed maybe they don’t know what to do. They will give you anything and you will take it because they don’t know. So, we have to tell them this is what we want you to do. Give them terms of reference, this is what we want you to do so they will do it the same way. |
|  |  |

**Title of Interview/discussion: KII /B3**

**Date of interview:**

| **Interviewer/facilitator/questions** | **Respondent/responses** |
| --- | --- |
| **Good evening.** | **Good evening sir.** |
| So, thank you for the opportunity for the interview. | Thank you sir. |
| Tell us about Tb Wave 9 project intervention that we talk about that focused on decentralization of DRTB services in the state because before now the concentration for service was only at Uyo but now it has been decentralized into three zones; Ikot- Ekpeneg, Oron and Eket. So, we are just trying to find out is on this project and the intervention that follows; what and what could be done whether they should be strengthened and continued. Okay, just tell us what you know and thank you for the opportunity for the interview. | Thank you sir. |
| Tell us about this TB Wave 9 intervention that we have talked about that focused on decentralizing DRTB services in the senatorial centres? | I think it has made DR-TB services to be serious and to be more effective in Essien Udem. |
| Okay, how did it help the patients; how did it help health workers; how did it helped DR-TB program? | It helped the patients because the facility is close to their houses and some of them do trek down to the facility to receive their drugs even though they cannot go; we health workers do go to their houses to deliver their drugs. |
| Okay, this idea of no more going to Uyo; do you think it is good? | Yes, it is good sir. |
| Do you have other reasons to show that it is good? | Yes, it is good because sometimes, you know before we even get to them; some of them or most of them have even suffered from TB for long time before diagnosis; so when we diagnosed them; for them to use their time going to Base-line, going toUyo or OPD before delivering their drugs to them; now that the decentralization of DRTB has come it is quiet close and easy for us to get them to Ikot-Ekpene before the Base-line when the results are out; their drugs will be out and immediately without wasting time. |
| So, those are the good aspect of it? | They are the good aspect of it; yes sir. |
| Was there any challenge?  Do you think it came with any challenge? | Challenges?  No! |
| Okay, now let us go to the components; just tell us the advantages and disadvantages of DRTB decentralized treatment? | The advantage is that patients are close to the facilities.  We have good relationship with them and close communication with them; so from there, they can easily get better faster; in that mood, we communicate with them and tell them that will recover quickly hence the doctors, the nurses and other health workers are there with them and show concern for their health. We communicate with them; we draw them closer to us and keep encouraging them in all aspects both for taking of their drugs; living good lives for them to be well and fully recovered. |
| Okay, is there any disadvantage? | Yes! Some of the patients don’t have transport money to come to the facilities but because of the closeness as the result of the decentralization we do go to their houses and deliver their drugs to them. |
| Now, let’s talk about the components; are components of this DRTB intervention that you are not aware of? | No. |
| Okay; there was a time you were inviting DRTB survivors to come give counselling? | Yes, we counselled them. |
| Do you think that one worked? | Yes, it worked; it encouraged the present patients that since the DR-TB survivor is alright now, me, I will also be alright too, since te DR-TB survivor is alive today, I also wil also live. |
| You think that is enough for that to; if government is here again; do you think that should continue? | Yes, that should continue. |
| Do you think there will be any problem if you want to implement that in almost all the facilities or LGAs in Nigeria? | It is good. |
| Is there going to be any problem? | No..no...no..no...no. |
| Do you think we can find enough DRTB survivors to be able to do or perform that? | Yes. |
| Okay, how often were you calling the DRTB survivors to come? | Before the ten months of DRTB treatment, the survivor can be free from the treatment. |
| Does the survivor go to their houses? | Yes, they do. They go to their houses even though if their numbers are not around; we inform them during the OPD then. |
| Okay, that means survivors were picked based on those regional lists? | Yes. |
| And you think that if you decentralized that you still be seeing people that will be doing those work? | Yes; yes; yes. |
| So, you think this one should continue? | Yes, it should continue. |
| Which other aspects of the program do you think should continue? | “Long pause” |
| Okay, you are not a TBLSLO, you wouldn’t know GX alert? | Yes. |
| Doing investigation again in Ikot-Ekpene, do you think it was of help? | During investigation? |
| Doing Base-line investigation in Ikot-Ekpene, do you think it was of help? | Okay, okay. It is good for us to continue it than for us going to Uyo. |
| In carrying VLO role, did you ever interact with the VLO for this project?  Do you think it worth it that we should have VLOs? | Yes, yes, we should have. |
| Why do you think so?  Why? | The VLO did their best in all aspects; once in a while she with other Team members will go to the houses of the DR-TB patients one by one and ask after their health; knowing how their health is, asking them questions like; have you been taking your drugs?  How is it doing you?  How have you been feeling? And others; so it really helps and it is good. |
| Do you think that it worth for them to continue? | Yes.  They should continue; it is very good and is encouraging. |
| Okay, is there any other thing you want to tell us about this DRTB decentralization? | Yes, this DRTB decentralization is really good because we have one language; we speak one language and if you go to where you can meet another language; it can make them not to feel comfortable like having Hausa or Igbo doctors for the patients can make them not to feel very comfortable. Here in Uyo both the patients and the health workers understand ourselves because they speak, they speak and we both hear and understand ourselves; yes we understand each other very clear and better understanding and this is one thing very good about DRTB decentralization. |
| Okay, so apart from using this DRTB Focal Person; DRTB survivors for counselling; is there any other one you would want it should continue? | “Long pause”  Okay, punctuality there; for us to be punctual as health workers.  Be on your duty post and at your facility on time; being there on the days of work for the patients to receive their treatment and drugs whenever they want, any day, anytime they need their drugs. |
| Okay, now we have started the decentralization; do think it should continue? | Yes. |
| That means activities from Ikot-Ekpene continue and you can create other centres from Ikot-Ekpene?  Do you think it is good like that? | Yes, it will be okay and nice. |
| Why? | Because it saves cost of transportation, this is one and most of the parent of the clients, it encourages them to come because it is closer.  They discourage them from going to Uyo because of distance but since it is now closer, they encourage them. |
| Okay, do you know whether they will have enough health workers to work in those centres that may be established? | Yes, because before the establishment of those centres they should make provision for enough health workers to work in the centres. |
| So, you are in support of the decentralization? | Yes, I am in support of it. |
| Do you want this decentralization to replace the old model one or do you want the two to remain side by side? | This DRTB decentralization should be maintained; they should continue with it; Uyo should go to Uyo as usual; here should also remain at Ikot-Ekpene. |
| So, you want people from here to remain here? | Yes. |
| You don’t want this one of bringing patients from here to there? | Yes. |
| Okay, do you think that this decentralization should continue?  Do you think it should be sustained? | Yes, it is life matter and it has been encouraging me a lot.  It should continue. |
| What you said is encouraging? | The DRTB intervention program because it is life saving. |
| Okay, is there any other thing you want to say about DR-TB decentralization? | I want to thank God for all your supports and encouragement to us and on our people who are the patients or our clients so far.  Thank you very much for that; we are very grateful and God will continue to give you life and bless of all you in Jesus name.Amen. |
| Okay, Amen!  Thank you very much. | Thank you sir. |

**Title of Interview/discussion: KII/B4**

**Date of interview: Tuesday, 8^th^ August 2023**

| **Interviewer/facilitator/questions** | **Respondent/responses** |
| --- | --- |
| **Good morning.** | **Good morning sir.** |
| So, like we have said, our focus is on decentralization of TB services(DRTB).  So, we start at once; what do you think of TB Wave reach 9 project which focus on intervention geared towards decentralization of DRTB services | The decentralization of DR-TB services is good because of the cost of transportation; like there are some Local Government Areas that are close to Uyo, so they go to Uyo to access their treatment services; and those close to Ikot-Ekpene also access their services at Ikot-Ekpene. |
| So, of that DRTB projects which part of the project do you like most or that appeal to you most? | The part I like most is the support; like the transport support to come down to this centralized site or the laboratory to access treatment and services. |
| Okay, what about the part or the role you played being a counsellor? | I liked the role I played; I felt happy coming to talk to diagnosed patients that want to start receiving treatment. |
| Why is it that you did not mention this at first? | “Long pause” |
|  | I forgot. |
| Do you think that one helps the patients? | Yes. |
| How do you think it helps? | Because I (DR-TB survivor) took the drugs and I am okay now so I encourage them to take the drugs so that they will be okay like me. Because my doctor told me that there are many TB patients dying because they are not taking the drugs. |
| Okay is that why you were interested in being part of the project? | Yes. |
| Now, which aspect of the decentralization didn’t work well for you? | None sir. |
| What made you to be part of this project? | When I had TB, I was receiving treatment from chemist shops which never solved my problem, because I still coughed out blood until when somebody directed me to health centre and I did and was diagnosed of TB after a medical test proved that I was positive for TB. Then, I commenced treatment for TB and the drugs really helped me. |
| Now, for the work you are called to do, did you have challenges? | Let me start with an instance, my brother was infected with TB and people around said that I am the one who gave it to my brother because the sickness started with me first; they insisted that I should be the one to take my brother to hospital for treatment. So, this was a very difficult situation for me. |
| Okay, in the project what were your challenges as a counsellor? | I don’t have any challenge. |
| Now, compare the processes of taking the treatment during your time and to those who are taking it now and tell us which one is better? | When I was taking the drugs, it was almost oral and injections but now it is completely oral with non-injection. |
| Apart from the drugs, we want to know if you prefer your time when patients were coming to only a centre than now when patients are going to different locations as the result of the DRTB decentralization to access drugs; which one do you prefer? | The decentralization is better. |
| Why? | Decentralized DR-TB care is better. There is a reduction in the cost of transportation because patients can now have access to treatment and services in locations that are closer to them unlike our time. |
| What do you think are the advantages and disadvantages of DRTB decentralization? | There is no disadvantage from the decentralization and I have already told you the advantages earlier in cause of this discussion. |
| So, no disadvantage? | Yes. |
| It is okay.  What are those changes or adaptations you made that helped you fit into the counselling services? | I managed to fit into the counselling services for DR-TB patients through the knowledge and experiences I derived from the health workers that offered treatment and services to me of which counselling was one of the services I received. This experience has been helping me to encourage my brother to take his drugs and follow up with other medical services because he is on TB medication now. |
| Okay, what components of the intervention did you want not to be changed? | There is nothing I want to be changed among all the components of the intervention. |
| Okay, could you mention one component that you don’t want to be changed? | One of the components that I want it to remain as it is should be counselling component of the intervention. |
| Okay, you remembered your constituency.  How well was the centralized DRTB decentralized services meet the need of the people? | Like now that there is high cost of fuel and the transportation is high; I solicit that transportation fare should be added to the TB patients in line with the current situation to ease their hardship as the result of high cost of transport fare. |
| Okay, more money should be added to their transport? | Yes sir. |
| Okay, do you want the decentralization to continue further or do we reduce it. | “Long pause” |
| Do we continue DRTB treatment closer to the people beyond the three zones they are now? | Yes. |
| Yes on what? | The decentralization should continue. |
| Why? | It is because of the transport cost. |
| Okay, what is your motivation in supporting the implementation of DRTB treatment and services? | “Long pause” |
| What made you to join or support DRTB services? | It is because I have seen the need for counselling people and I have also seen the need for the decentralization. So, as it stands now when people cough; they are quickly referred to me because people noticed that I was in that situation before but now I cough no more. So, they come to me to know how I got solution for my condition to enable them follow the directions and I have been directing them accordingly. |
| Okay, how confident are you that this process will continue? | Which process? |
| Decentralization now? | I am very confident. |
| Why are you confident? | It is because people are still coughing and the continuations of the process will bring solution to their condition through treatment and services. |
| Okay, how do you think decentralization of DRTB services are of need to the patients? | The decentralization is okay but during OPD clinics; if you want to see doctors, they now take another sample from you to go and do follow-up test to know if the treatment is progressing or retrogressing. |
| Now, this decentralization and the formal one; do you think that having one centre where TB patients come for treatment should be maintained or off the track or should both the old method and decentralization work side by side? | I like how it is decentralized. |
| Why? | It is due to high cost of transportation; so, if it is not decentralized, is not everybody that will be able to take treatment if one centre is maintained. |
| Do you have any other thing to tell us? | I have nothing more to say. |
| Nothing more? | Yes. |
| Okay, thank you. | Thank you sir. |

**Title of Interview/discussion: KII/B5**

**Date of interview: Monday, 7^th^ August 2023.**

| **Interviewer/facilitator/questions** | **Respondent/responses** |
| --- | --- |
| **Good afternoon.** | **Good afternoon sir.** |
| Okay.So, like you know we are working on; this interview is centred on TB Wave 9 Intervention which is on decentralizing DR-TB treatment services on service delivery.  What do think TB Reach Wave 9 Intervention to decentralize DRTB treatment services? | I will say it is a good intervention which is already in the state; we already decentralized DR-TB services in the state. so Tb Reach Wave 9 Intervention is really a good one, it has been helping us; I mean, it has taken the treatment and services closer to the patients and indeed, it is a good one; yes, it is a good one |
| So, it was a good intervention? | Yes, it was a good intervention. |
| So, what do you like most about the intervention? | Everything about it. |
| Everything? | Yes. |
| Okay, what should come top most; what should come number one among the interventions that made the intervention so unique and wonderful? | Like the counselling that made it possible for DR-TB survivors to provide counselling services to the new TB patients was a wonderful one; it was really a good one. Let us start with this example; that I am sick I see another that has passed through my condition talking to me; it build one’s confidence, makes one more open to receive treatment and that was a very good idea. Survivor counselling approach. Another one that is also good is having a VLO in the community which also helps fast-track the patients and bring them for treatment; so it was also a very good one too from the intervention. |
| Were there any odd aspects of the intervention? | The odd aspect of the program was the GX alert because me in person do not get alert. |
| You don’t get alert? | Yes sir. |
| Why? | Until the lab. Focal Person gets and now post it on the general platform; that is when I now get and I don’t know |
| So, from time immemorial if I may use that word, you have not been having GX alert? | Yes, maybe most times the PM could forward it for me or when they put it on platform that is when I will say “oh someone has been diagnosed” but as for me, I don’t receive the alert. So that one didn’t work well for me. |
| Okay.  What has affected or influenced your practice of decentralized DRTB treatment services? | Affected? |
| Yes. | In terms of? |
| What influenced your own practice of decentralizing DR-TB treatment services? | “Long pause” |
| Okay.  What helped you most in supporting the decentralization or what is the reason you are in support of the decentralization? | Okay.  It helps to bring the services closer to the patient; so it helps in that aspect, like we have some; you know having the different centres; is now like a cluster while the other people from the far Local Government Areas; so decentralizing helps those from the far Local Government Areas to access the services. It reduced the financial cost still high but not as higher as coming down to the state.. |
| Were there challenges with the implementation of the decentralization processes? | Yes, we have some challenges and that would be with the mapping. |
| How? | We have people conducting the investigation tests but their prizes are not friendly; so like in Oron, there is no lab there for now, so we still have challenge in that area, so if you have any patient in Oron you still need to bring the patient down to Uyo for the investigation test. |
| Apart from that, any other one? | Sir if I do remember any other one I will tell you |
| Okay. | And also, another challenge is the documentation. |
| Documentation, how; explain? | There are those forms we normally interview; they send no samples to the lab. Though RedAid has been trying doing zoom meetings like capacity building meetings for the Dots but even with that we still have those challenges; improper documentation of patients’ data is actually one of the challenges. |
| Okay.How does the decentralized DR-TB services compare with the routine one that is not focused on decentralization? | I won’t really know because I came on board to meet with decentralized services in the system. |
| Okay.Under the non-decentralized, if I may use that word; it means everybody will be coming to you to access treatment and services; how do you compare it to this one whereby services are decentralized? | Coming to you could be challenging; quite challenging to one; the patients; let me say to the lab. Scientist and the health workers for all over the state to be coming to one place; it is quite challenging in way. So this decentralized care system has eased workloads for the health workers and it ia also very favourable to the patients. |
| Okay.What are the advantages of decentralized DR-TB treatment services? | Patients’ easy access to facilities and services.  It assists patients to get services at ease.  It assists health workers reduce workloads and it equally builds their capacity because whenever we go or when we have opportunity especially the key officers to know what they don’t know before.  And also it reduces financial burden on side of the patients. If I should use Oluga; oluga now is a big Local Government; so for them coming down to Uyo; even from Orong-nam which is at the interior or out sketch of the Local Govcernment; so if you now tell that person to come down to Uyo to do Base-line screening, you will lost the patient on the road; so this has reduced their financial constraints and also, it has help them get their treatment without much stress. |
| Did it affect outcome? | It is positive. |
| Any disadvantage? | Anything that has advantage will definitely have disadvantage. |
| So, what are the disadvantages? | You know, like decentralizing; you need to build capacity of health workers, so some of these health workers like in Eket and others places we have visited have little or no capacity; that too is a disadvantage; you know we didn’t train them on DR-TB; I mean proper training on DR-TB but because it has been decentralized to those places; we need somebody to stand in; at least a doctor to stand in to see these patients and attend to them; so this is one of the disadvantages of this decentralization. |
| Okay.  Any other one? | “Long pause” |
| Any other disadvantages? | I don’t think so. |
| Okay, thank you.  What kind of changes did you need to make? | “Ehae”...another disadvantage; for the state people touring around it...”laugh”...though we have a support from KNC but that support only takes you to one decentralized place; so you going to Oron, or any other place...”.laugh”....you know that one. |
| Okay, thank you.  So, what kind of changes did you need to make, you as a person for the decentralized DR-TB treatment services to work effectively in your state? | “Long pause” |
| Do you make any change?  Do you make any change; it affected no scheduled because all those touring are also part of the changes you have done? | Already I know these are the days I am going for clinic and I will plan myself ahead. |
| Okay, you didn’t make any special changes on your plan of schedule? | Yes sir. |
| Okay, that’s good.  Are there components of this intervention that should not be altered; I felt I should write out these interventions because there are few ones on my head but the other ones may feel cheated; so, the interventions are;  GX alert which we have talked about.  Then improving tracking of patients by supporting the Global Fund funded community organizations (CBOs)  Improving counselling of patients through introduction of structured free (three) treatment counselling and team counselling and DRTB survivors.  Decentralizing base-line investigation to more peripheral lives.  Providing transport support to patients for Base-line investigations.  Employing mobile connectivity solutions to improve turnaround time of base-line investigations using unstructured supplementary services data (USSD). | Sorry, this last one you mentioned here are they doing it?Okay. |
| You know all the components to large extent, not all of them were fully done.  So, it depends whatever you are seeing.  Then verifying quarterly treatment death using verbal autopsy; this one is not well captured to a large extent.  Then, decentralizing treatment initiation to the LGA.  And then, engagement of a state’s liaison officer.  So, which one do you want unaltered? | I don’t want them altered. |
| Everything? | Yes sir. |
| What about your GX alert system? | That one; maybe the fault is from the person that normally sends it; you can alter that one to meets everybody |
| Okay; yes...yes...yes; they will know.But the other ones should stay that way? | The VLO to me, you know Akwa- Ibom is a large state, I will suggest they will have at least one in each of the zones. |
| Because they only have one VLO in the whole state.  One moving round the whole state? | Yes; going Mbolo, Mbong, so, we need two or three VLOs. |
| In that case you support all the interventions? | Yes sir. It is helping us a lot. |
| How complicated is the decentralized DRTB treated services? | It is not. |
| It is not?  What makes you think it is not complicated? | It is not because we have been doing it; to me I came in to meet it and it is not complicated. |
| What could have made it complicated? | If they didn’t decentralized it? |
| You are saying it is not complicated any reason for this? | Yes, because it makes the work easier not cumbersome. |
| Okay.  How well do the decentralized DR-TB treatment services meet the needs of your patients? | “Long pause”  It helps them access treatment and services easier because it brings care closer to them; also you know; decentralizing it, having supporters to tell them the dos and don’ts; making them to have somebody to call; helps them to this quick movement to move; you know if have somebody around you that tells you “take your drugs oh!” it works; so, for me I wouldn’t have been able to do all that alone. So this decentralization helps to get someone who asks them to adhere; “ have you taken your drugs?”; “how are you feeling?”they can easily call that person and that person will reach to us; it helps to take their treatment; it helps them to take their drugs and also, the financial aspect , it ease them with that hence they can easily access their drugs instead of coming down here; for example, if I am living in Eket, I can easily get to Emmanuel hospital to access my drugs and treatment instead of coming down here. |
| So, in any case it is a very good innovation? | Yes sir. |
| Alright.  In your state has be strong need to increase or reduce the level of this decentralization? | Yes, there is need to increase it. |
| Explain more on that? | We still have some LGAs that are still far; we have an LGA that shares boundary with Abia state; we have the other one that shares boundary with Rivers, so there is need to decentralize to other LGAs because we have some patients in those LGAs who could not come out to where we have the GOPD sites because of transportation; most times what they do is, the TBLOs might go there and get samples from them and get their medications. So, sometimes we need to decentralize to those other far LGAs. |
| Okay.What supports are readily available to help you to adopt the decentralized DRTB service delivery in your state? | We were able to adjust to the decentralization because we have the structure, also we have the man-power. |
| What did you have in mind when you said structure? | At least we have DOTS sites which we can use and in each of those Dots site there are human personnel there which if we do a little capacity building they will be able to handle the patients out there. |
| Any other one?  Any other form of support? | Yes, some partners like ARFH provide support for the social services like transport fare and all that for the patients. |
| Which partners and what did they support on? | We have “ARF”; they support a little bit on transportation for the patients. |
| Okay.So, apart from that, any other supporter?Any other support? | “Long pause”No. |
| So now let’s go to incentives; what incentives are available? | Sorry, KNCV do support. |
| KNC do support? | Yes. |
| Which type of support do they provide?What did they support on; transport? | KNCV provides for tracking of the patients; they support the health works to track these patients within the localities. |
| Okay.Any incentive?  Are there incentives attached to this to ensure this decentralization is successful? | “Long pause”  Sir, can you explain more on that? |
| Incentives like tips, any form of help, assistance and monetary reward? | “Laugh” Not really. |
| Enyeeeeh? | The only incentive is that KNCV assists a little more on transportation to do all those tracings. |
| Okay.  That is an incentive. | Yes and the one I mentioned that ARF does. |
| Okay.  So, KNCV supports the state team while HAV do support patients. | Yes, KNCV supports the state team while the HAV supports the patients. |
| Apart from the monetary support on transport, any other thing? | There is no any other thing. |
| Okay.  How do you think that the structure in the state or the setting in the state has affected the implementation of DR-TB decentralized treatment services either for good or bad? | The state structure? |
| Yes, the state structure, what is obtainable in the state or the culture of the people in the state; did it influenced for good or for bad on the decentralization process? | It has influenced the process for good. |
| What is the good; what do you have in mind? | Because these people are our people and its influence to me is for good because the structure...sorry, let me get it right; the structure like human structure? |
| Everything. | Okay. |
| Okay, let’s leave political structure. | “Laugh”...we don’t even have political structure |
| Administrative structure, the way the state is organized, event the tiers of government; local government; how did it affect either for good or for bad the process of decentralization? | Whether they have affected it negatively or positively? |
| Yes, we want you to expatiate on that? | Before now some group believed that this is not a treatable disease and that is where it affected it negatively because some might refuse and some might not even come out for the treatment; but positively with one of the intervention then that some of the survivors do come out to receive the treatment and the state and the health workers are here for the good of the patients. |
| What have been your motivations for wanting to help ensure the successful implementation of decentralized DR-TB treatment services? | My motivations? |
| Yes. | My motivation for a successful decentralization is seeing a sick DR-TB person accessing this treatment easily and at a close by location and you know, that after some weeks the person becomes healthy coming out on his/her own; it motivates me a lot and that means that what I am doing is good. They assess treatment easily, you know close to them because it is close to them they will easily access treatment and services and you see them coming up healthier,; it motivates me the more; so, if you can work for this patient for the positive change another person close by can come; so seeing getting better is my motivation.  So, seeing them getting better at the close by locations becomes my motivation for the decentralization. |
| Okay.How confident are you about been able to implement the decentralized DRTB treatment services consistently in your state? | We can do it as a state. |
| What gives you that impression? | Already, we have the man-power and they willing to work; we can work. |
| Okay.  Any other thing? | No. |
| Alright.  So, how confident are you about other members of the state’ team about being to ensure the continued decentralized DR-TB treatment services continue to function? | How confident am I with them? |
| Yes, how confident are you with your colleagues towards the sustainability o the decentralized DR-TB treatment services in the state? | Working for the success of the decentralization, I have enough confidence because almost all of them are permanent staff, we don’t use ad-hoc staff for the process; so, they are state staff, local government staff and they are there; definitely, it is part of their job description and I am confident enough. |
| Okay,Alright.So, how well does the decentralized DRTB service delivery fits your existing work processes? | It is our work and that is what we have been doing. |
| It fits you well? | Yes sir. |
| Simply because you are doing DRTB decentralization treatment services? | Not only DRTB decentralization services but that is my major work here. |
| This decentralized DRTB treatment services, do you want it to continue, replaced or complement the existing services or do you want it to replaced the non-decentralized approach or compliment it? | It should complement it. |
| Why should you say complement? | It is because when we don’t have decentralized, we have non- decentralized; so, it comes it should complement with the existing one before it comes. |
| So, that means you want the two to be operating the way they are operating? | Yes. |
| Any other thing you want to say about decentralized DRTB treatment services? | “Long pause” |
| Okay.Nothing; so, that means we have come to the end; about those interventions, which one did you rank first? | The one I ranked first? |
| Yes, the one you prefer most; first, second and third; I think I heard from most people; GX alert, improved tracking of patients using global funds supportedCBOs, improved counselling using the DRTB survivors, decentralizing Base-line investigations, providing transport support to patients, improving mobile connectivity solutions, verbal autopsy, decentralizing treatment initiation in the LGAs and engagement of a liaison officer | GX alert is number one.  Improved tracking of patients is number two. |
| So, which one is the best to you? | For me these three components of the intervention are excellent, decentralizing base-line investigation,  Improved counselling using DR-TB survivors and then the engagement of the state liaison officer. |
| Okay.Thank you very much. | Thank you sir. |
| Bye-bye. | Bye! |

**Title of Interview/discussion: KII/B6**

**Date of interview:**

| **Interviewer/facilitator/questions** | **Respondent/responses** |
| --- | --- |
| Madam, like I told you, we want to discuss about that project that you usually go out to talk to TB survivors, what do you usually tell them? | What I usually do is that I will first of all introduce myself to the DR-TB patients as one of the DRTB survivors, I will introduce TB to them and tell them that it is not a death sentence, that it is a curable disease, that if they take the drugs correctly and complete their treatment, that they will be cured and that they will not die. That TB is not like HIV that you will continue to take the drug for a very long time or throughout life.but for this one, when they take it properly, you will not have any problem. I will tell them that TB is curable, that the government is taken care of the bills for the drugs and give them free of charge without asking them to pay. That there is no injection again.  During my own case, I was taken injection before I started taken drugs, that they should not drop the drugs because of the side effect, that they should take enough fruits or other things like milk so that the side effect will not be much on them.  I will also tell them that the government will give them some stipends for their transportation allowances. |
| In your mind, do you think that these messages that you pass across to them do work? | Yes! It worked very well |
| What made you think that they work? | Because there is one of the patients that used to call me whenever she takes the drug, she begins to describe the side effect for me, I will tell her to eat fruits, vegetable and milk, that she should not take alcohol because it will affect the drug. |
| You are sure that the woman completed the treatment? | Yes |
| Then, if you have chance to advice government, will you tell them to continue this kind of thing? | Yes |
| Why do you think that they should continue it? | They will save lives in they continue the treatment. |
| No, not the treatment. I mean using people like you to talk to people that want to take treatment, will you advice the government to continue doing so? | Yes |
| Why do you think the government should maintain it? | Because, if you do not see somebody that will advice you and encourage you to take a certain drug, someone will drop the drugs because of the side effects of the drug. In my own case, the drug tormented me so much, if not the CLO that advised me, she calls me every time to take the drugs. But, if you do not have somebody that is advising you, you will drop the drug in fear, thinking that the side effect of the drug will kill you. |
| Okay. Now, if government want to continue using people like you to be talking to those with DRTB to continue to take the medicine, what problem do you think the government can face? | There will not be any problem |
| Do you think there will be DRTB survivors to do that work? | Yes |
| How many DRTB survivors do you think that we have in this state now? | I don’t know |
| If the government is able to locate them, do you think that they will be willing to do what you did? | Well, I don’t know. This thing is a personal decision. If someone decides to go and do it, he or she will do it. Its not a matter of forcing the person, if you force the person, he or she will not be able to do it. |
| How many times did you have any contact with them? How many times did you see them and talk to them? | Its usually when they come to the hospital, the CLO will call me |
| Okay | Yes |
| Because they cannot get all of them at once, whenever the get any patient, they will call you, then you move from here to IDH? | Yes |
| Were they given transport to go? | Yes |
| They were given you transport? | Yes |
| Even if its one person they will call you? | Yes |
| How many times do the call you to see one person, will they call again to come and see the same person? | No |
| At what point do they usually call you? | Before they give out the drugs to the person. |
| Okay. How do you know that they do finish their treatment since you do not see them again? | They do contact the CLO |
| Okay. You usually get your information from the CLO? | Yes, I also use to give them my contact details |
| Did any of them call you? | You |
| What did they ask you? | About the side effect of the drug and that they will not be able to continue taken the drugs again because of the side effects. |
| How many people call you? | 2 people |
| What did you tell them? | I advised them to continue, that this one is not worst like injection. Like me I took injection before tablet. I advise them to take the drugs, they should not take alcohol, they should take enough fruits and take milk, even though its costly, they should take it once in a while. |
| Apart from Ikot Ekpeneg, did you go to Uyo to talk to people who has DRTB? | No. Another person is there. |
| Good. That time that they treated you, where did you get your treatment? | The first time I went to IDH to collect the drug. |
| When you did the laboratory examination to commence the treatment, where did you do it? | At Uyo |
| Assuming you were told to go to Ikot Ekpeneg that time, would you have agreed? Maybe they will say, you should go to Uyo instead of going to Ikot Ekpeneg, would you have liked it? | No |
| Why? | Ikot Ekpene is near and they have enough equipment for the laboratory investigation here in Ikot Ekpene. |
| Okay, because you do not know whether they have what you want? | Yes |
| Do you think that going to Ikot Ekpeneg where nobody knows you than going to Uyo where somebody knows you, which one do you prefer? | Hahahahahahahaha (laughter). I don’t worry myself about whether somebody knows me or not. |
| You don’t bother about those things? | Yes, my concern is my health |
| You will worry about the fact that people will know that you have DRTB? | Yes |
| So, there is no problem having DRTB? | Yes, if you have DRTB, just make sure that you are treated. |
| Assuming that I have HIV, do you think I will prefer going to ………… | Hahahahahaha, that one is another case. |
| So, the fear of HIV and TB are not the same? | Yes |
| Okay. | Yes |
| For HIV, somebody can go to Uyo? | Yes |
| But, for DRTB, you can go to Ikot Ekpene, you will not mind? | Yes |
| Okay | Yes, because it is curable and it does not last for a very long time, you will be treated and you will be okay. |
| Okayooooooooooooo! | Hahahahahahahaha (laughter). I don’t knowooooooooh! |
| So, you are believing that DR TB and HIV are not on the same level? | Yes |
| At all | They are not the same |
| So, you would have preferred going to Ikot Ekpeneg to get all your treatment? | Yes |
| If they ask you, will you advice the government to stop making people to go to Uyo? | I don’t know. It depends on the facility that is close to them. |
| Assuming there is somebody in our community will you prefer that the person goes to Ikot Ekpeneg not Uyo for the treatment of DRTB not HIV? | If the person sees that everything is complete at Ikot Ekpene, I prefer Ikot Ekpene to Uyo. |
| You are seeing that everything might not be complete in Ikot Ekpeneg? | Yes |
| So, you will ask the government to make everything in Ikot Ekpeneg to be complete……….? | Like Uyo, so that it will be easy for people. You know that from here to Uyo is far and transportation is costly. They should make provision for Ikot Ekpene. |
| Is there any other thing you will like the tell us about the work you did that time? | Like what? |
| Anything you can say about it | I will advise government to produce more drugs and give more transportation stipend because the amount they are giving is not enough |
| That is for the transport? | Yes |
| Any other thing? | Government can produce more drugs for them. |
| Is it new drugs or what? | Anything that will work for them |
| More drugs | Yes |
| You know that the government is trying, you said that you too injection and people are no longer taken injection now? | Government is trying so much. Do you know what it will cost you to go and buy those drugs on your own. It is very costly and many people cannot buy it. I thank God for the efforts of the government to have been producing these drugs for patients. Government should do something about Ikot Ekpene so that they will not be taken their samples to go for test in another place because it usually takes time before the result will be out. |
| But if everything is in Uyo and working properly, things will begin to work very fast? | Yes |
| Is there any other thing you want to tell us? | No |
| Okay. We have come to the end of this discussion. Thank you very much. | You are welcome. |

**Title of Interview/discussion: KII/B7**

**.**

**Date of interview: 10/08/23**

| **Interviewer/facilitator/questions** | **Respondent/responses** |
| --- | --- |
| Okay! Good morning | Good morning, Sir |
| Today is Thursday, 10^th^ August, 2023, and we are discussing with the TBLS from Etim Ekpo L.G.A Akwa Ibom State, Nigeria. So, ma, thank you for agreeing to talk to us and also that the interview will be recorded, we appreciate, like I told you before, we are discussing TB range which the aim was to decentralize DRTB Services. By decentralization we mean, before everybody de come to you to do everything, but they kind of said people stay in Iket-Epene, State at Eket and also obtain the same services, so that what we are discussing. Ok so we will start with this way so what do you think of that TB reach an intervention, how do you see it? To decentralize DRTB Services. | Okay! First of all, it helps because before we came to Uyo, everybody came to Uyo then but they now moved us to Ikot-Ekpene, but still it is very far from Etim Ekpo, still it’s far from us, we coming from Etim Ekpo cause Etim Ekpo is not just the Secretariat alone, it is inside. Some of my patients are coming from inside Etim Ekpo, the border of Abia and Etim Ekpo and now, when things are coming far like that its difficult for them to come to that kind of place, it is far before they can reach to Etim Ekpo Secretariat, they have spent enough money and before they can reach to Iket-Ekpene, much for them the patient so it’s good to be at Ikot-Ekpene but it’s also good for it to be at Etim-Ekpo. If you can find one point for it to be at Etim-Ekpo also then this Etim-Ekpo, Eka is close to Etim Ekpo, Ukanafo is close to Etim-Ekpo and Etim-Ekpo is the middle of Eka, Ukanafo, we can, we ourselves can join together, while Ikot-Ekpene is far (spoke language) close to that place and they will not spend a money and it affects our patients here in Etim-Ekpo to Iket-Ekpene was so also, the same thing at Uyo so that my feel. |
| So, you like the project but you are even saying even Iket-Ekpene is for people coming from inside. So what do you suggest? | I suggest that we can draw it close to Etim-Ekpo to help us in Etim-Ekpo, Eka and Ukanafo. |
| Why do you want them to draw it closer to Etim-Ekpo? | To help the patient not even my very self, the patient because sometimes they complain of money and I see it with them that from hmmm, I have two on ground that are taking treatment now mother-in-law and daughter-in-law very far, before they can reach to where PAC is, is 3000 Naira. I don’t talk of now oh! Then, the 3000 Naira before they can come out from where they live to where PAC the secretariat is, was 3000 Naira, then before they moved from that secretariat to Afian junction, is 1500 Naira, then before they can move from that to Iket-Ekpene 2000 Naira that to know the whole thing was and it’s painful to them they don’t have money, I don’t talk of myself. I can fuel my bike then 4 litres to and fro and fuel another one and came back. But for the patient it’s very difficult for them to reach to that place because when we talk of Etim Ekpo, Etim Ekpo is not just place like this, very very, as I am talking like this, that place they are living is boundary with Abia, and Etim-Ekpo, that’s the challenge. |
| Okay! Thank you for that, so we will still come back to this. Lets go to TB Reach Wave 9, what about that project that you like so much, that the project, that decentralization movement, pushing than making it possible for people to access DRTB Services outside Uyo. You understand if you look at that project, all together, which aspect of it do you like very well. | Moving from Uyo to IK? |
| Yes | That IK was still okay for those patients that are not living inside Etim-Ekpo, it was okay. |
| What we are looking at is the major thing that we are moving away from Uyo. Do you like that project, do you like it that way? | I like it. |
| Now, let’s see that DRTB Reach Wave 9 in totality, what about it that you like so much about it, the decentralization. What aspect of the decentralization do you like so much? | What will I say, what I like so much there is, they now move this thing to private lab, the baseline is where I want to start. They move from public lab to private lab, which anytime you have a patient like that man, that royal pathology lab to me oh, anytime you call the man, he will answer your call, if you say am coming today, am coming tomorrow, no argument, you go, service is on until. That is, because you people attached private, is private but not public, I don’t think of the public, the service will be on like that, that is the only thing me I see always and I say oh this man is very good, you call him even by 12:00am in the mid-night, he will answer immediately, the test is done then he will now call you. If it is public lab, they will not call you |
| Are you not seeing it that it is because of that man as a person or because it is private, how do you see it | Although it may be from that man as a person but it is also because, its private, he knows his gain inside of that thing. But one’ though he may collect some incentives, but not to say it’s for him alone he may have HOD, that everything is going to the HOD,not specifically to the man that is doing the test, but this one is his own office, he can control and |
| Yes, because we were with that man yesterday, and I can agree to what you are saying about him. Okay is there any, you don’t finish, is it only that private lab na in sweet you pass | No, it is not, it’s not, this people that are working there are like deliver their services, they will now call every time they look that patient will be free, how is that one, how is the other one, I mean the patient, this our local people in the state. I know they can work by calling, I know it is not easy. |
| Okay you are talking about the state, ok this people | You ask me of what is another thing you like, I now just bring it to this people, that are working in the state that they cooperate very well they don’t just say after all it is not my brother it’s not my sister, they will now bring the patient out, they want to see, they want to know and that is the first service we will like, times if they call the first time you will pick na, that’s always what happens because we are loading with something, so they call, you did not pick, but at the end of the day will pick the call, but phone Ring;;; |
| So that means you are happy with the state team or the whole community base organization or both | Everybody |
| They are doing well. Ok any other aspect, now is there any bad side of that intervention | Uhm mm the bad side, I have already told you |
| Ehn that one is a process, we will come to that is there any other bad side | Uhm, I think the only bad side is the transportation fare that is the only thing and in fact supervisor, want something supposed to get something. You know when we are going to baseline investigation, is me that will carry the patient to the laboratory but the transportation will be given to the patient not the supervisor. You go for baseline investigation and may not complete the investigations. |
| Okay, thank you, so what influence your practices of decentralization DRTB services, is it the type of sacrifice you are making, what influence you to do all those ones | It is the help for humanity that comes first. For example, if a DR-TB patient is in the community for a long time, he/she will affect others so if we get an alert in the platform that someone has DRTB, we will try all our possible best to bring that person for treatment starting with baseline investigations. So we will ensure that the individual commences treatment so he/she does not infect others in the community. |
| What makes the main point for you is to help humanity abi? | To make the program move forward |
| Okay, now are there challenges with this decentralization of DRTB services? | As I told you, I have said it already, the challenges are what I have just said |
| That money | The transportation is a problem. So the decentralization should continue up to my LGA- Etim-Ekpo so that challenges with transportation will be lno more. We in Etim-Ekpo LGA we know how much we spend before we go and bring a patient for OPD services, in fact you can call this patient on phone and she will say I don’t have money, you will not just leave him alone because he/she say he doesn’t have money. No. You cannot leave that person and go your way, you have to find way and you see you take it as a risk for you to set down on top machine with that patient because you want to help, no money to give him transport, this machine carry him, you enter this machine, you take risk, you cover your nose everywhere so that you move with that person to OPD. That is the challenges even when I come back, I will pray a lot. |
| Okay, let discus the component of this intervention, because you have talked about GS and ART, cause the number one of it is expanding GXalert to supervisor, where you getting alert then? | Alert in phone on this in telling us, I mean the alert that just inform us that so so andso so government. |
| That is the WhatsApp group. | Yes, the WhatsApp group. |
| Yes, that is the way, we modified that alert. | Why now let us talk of money. |
| Okay, okay | What am interested in is money to do the work efficiently, alert of ………… |
| Let talk about that GX alert, how you take see am. | The information will be in the platform immediately they detect, immediately any lab detects a DRTB case. They will put it in the platform (WhatsApp group) that so so and so, they will bring out the name of the patient from the address you will see everything about the patient if he/she is from your LGA. Then people will start calling you about the case. Several calls will start coming in from the PM, DR-TB FP, SLO, everyone. The aim of the calls is just to make sure that the Supervisor identifies the patient and take him/her for baseline investigations. |
| Now that means that component is good | Yes, it is very good oh |
| Do you want it to continue? | Yes, sir |
| What did you think will be the challenges that may come up if it, if there is need to scale it up to national | The challenges are, if the people are not paid, if those lab people are not being paid, if they are not paid, I don’t think they will just drop it in the platform, immediately but may be because they are given something so that immediately they have this one they will now put it in the platform, everybody will see it, even if you say you did not see it, so you don’t have data. If you have data to enter into the platform, you will see. |
| So, if that one is supposed to be scaled up then, that means they should be ready to be paying the lab people so that, or to ensure they continue dropping the messages. Okay, number 2, that’s component one that we talked about so now, improving tracking of patient by supporting the global fund supported communities and organizations. | Just break it down let me. |
| Okay, the people, this community base organization, they track DRTB patients, like all those ones where you say they are calling you and, all that is part of making sure that things worked well, so that’s, the CBOs. How you see that? | As this one de track the patient of my local government not even once, but not monthly oh! They came, we now move from community too community where the patient is. |
| And you join them? | Yes, I join them, the last one, we now moved from community to community, we visited about 4-5 people that day from morning till that evening, they left. It helped because when they see another face coming like that with car, you see, this patient, they will know that it is important. |
| So, you think this one should be scaled up to? | Yes, sir. |
| Is there any challenge in the scaling up? Do you see problem if the opportunity comes for it to be scaled up? | No challenge, no problem. |
| The third one, decentralizing baseline investigate to the referral centres’, that is what they are doing by going to do that test at Iket-Ekpene. How you take see that one? | Doing that test at Ikot-Ekpene |
| The baseline test | If the baseline test is coming to Etim-Ekpo will there be any crime, if it comes to Etim-Ekpo. |
| What do you think will be the problem if the lab comes to Etim-Ekpo? | I don’t think there will be any problem. |
| You think that lab that can do all those things can be sited in Etim-Ekpo? | Since you people are using private lab. |
| Is there a private lab, standard in Etim-Ekpo, you think they can do all these tests. | I think oh, although, I don’t really know, just thinking. |
| Okay, you see, that is why we are looking at, this is an option, however these are the challenges that we are going to meet because that man at Ikot-Ekpene. There was one test that he was not doing so he has to go and find money to go and buy the re-agent to start doing that one. So, it means that, if we decentralize further into Etim-Ekpo or any other place, the chances of seeing a lab that will do all these may be very, very low. | Okay, let us do like this, it can still be at Iket-Ekpene for baseline, but where we talk of OPD that is monthly, let it be because that man is a good man. |
| Now, let us talk about workers that will see this patient. Do you have enough workers to see the patients? | We have general hospital at Etim-Ekpo doctors are there, if we do all arrangements at Ikot-Ekpene now, we could talk to them, they can do it. |
| Okay, now the fourth one is improving patient counselling by using DRTB Survivors. | No, I don’t want DRTB Survivors. |
| You don’t want them again? | Yes, the reason am saying this is last, I called a DR-TB Survivor with my credit for the purpose of counseling of DR-TB patient. He said I don’t have money, I have not eaten since last week, madam give me money to go and eat if you want me to come, give me transport money, me I will not be able to do all these things instead of that, let us use the State Team for that counseling since pre-treatment counseling is very good |
| So, you agree counselling is good. | Very good oh! |
| But you don’t want to use DRTB Survivors. | Ehn, because I heard that they were not paid. |
| So, if they are not paid, it means they are not paid na, you should have been campaigning for them to be paid na. | Campaigning from where? |
| So, you believe counselling is good abi? | Very good, sir. |
| So, you want that one to be scaled up, but you don’t want DRTB Survivors. | If they are no problem, if they can do it no problem, but for someone to come and request for food and money and every other thing, we will not be able to provide, because you people are not giving me the money to give to him. |
| Number 5, providing transport support to patient for baseline investigation. | So, we already do that but as I told you, is not, but that of Etim-Ekpo will not be okay, they can spend up to 7000 Naira before they can get there and came back-same not all oh! |
| So, what do you suggest? | Increment, to increase the amount. |
| Okay, employing mobile connectivity solutions to improve turn-around time of baseline investigation, that man, he said anytime that he does test, he said, will put in a platform and everybody will have access to it immediately that is it. | Yes! |
| So, you are witnessing it, you are seeing it. It is an app abi? | Yes! |
| Do you like that one? | Let it be, it helps. |
| Do you think of any problem that will follow this, if it scaled up. | That one is very important, but another phone call to the supervisor because not everyone of us will enter into it, no data sometimes, but phone call is supposed to have the number of all the supervisor that he is working with. Anytime this is from Etim-Ekpo, he now call me, this is from Ohorukara, he will call that is it. |
| Verifying reported pre-treatment death using verbal autopsy, did you do this one? | Autopsy. |
| Do you ever do it. | Okay, explain, whether I have done it and I don’t know. |
| Verbal autopsy is just all about going to find out and verify, like if Chijioke died under you, it is to go find out if it is true that Chijioke died, you have to ask questions. | No, I know go there the last one that died I called him and asked him if he died, it was the day of OPD they now put it in the platform that OPD is so, so, so day, I now begin to call all my patients, I call this one he did not pick, I called again and one of his brothers picked the phone and told me he died last week. |
| But it is a confirmation, this confirmation is more for pre-treatment cases because most times we find out that, most patients especially that time that they were all going to Uyo, it takes time before they conduct the baseline thing. They will be reported dead, while in real life, they may not have died., that’s why you should start going to verify, than just asking questions  Engagement of a state volunteer Liaison officer. | I like that one. |
| You like that one, wetin make you like that one? | You know one man cannot do this work alone, if another person is coming from the state you know, he is bigger than us and he knows. |
| Okay, how do they function and what makes you to like them? | Already, I have said it, the reason I like them is because they will also push the work. |
| So, do you want that to be scaled up? | Sir! Let it be. |
| Now, what difficulty or challenges do you envisage when the component is scaled up? | I don’t know of any challenge than I think for it to be closer to us. |
| Okay, of all this component wey I talk, which one do you think is the best? | All is the best. |
| Just name one nah! | Tracking. |
| Okay, we start again, we want make you compare this decentralization with the former one, how do you see the two, where everybody dey go Uyo? | No, the former method wasn’t good, everyone going to the capital for OPD was hard. You know that of Uyo, we didn’t use bike, its only Keke and Ikot-Ekpene we can get bike and move, but in Uyo, we don’t go on a bike, it was hard. Sometimes we just charter a motor, which was difficult, we have done that before, we charter a taxi, it was very costly. Its difficult but Ikot-Ekpene is okay. You can charter a bike and move to Ikot-Ekpene and come back. |
| So,its better that we decentralize. Okay, what are the advantages of decentralization. | The advantage is that, it help us. It limits our expenses and our time. |
| Any other thing? How does it helps the patient? | It reduces expenses for the patient. It saves time, the patient will not stay for a long time without baseline. At Uyo before, they will waste much time, they don’t go for baseline, talking of Uyo. Some people don’t know Uyo, they will think its very far, There are advantages in decentralization. It helps the patient. Ikot Ekpene is very close. . |
| Okay, what about disadvantages? | The disadvantage is that Uyo is very far from Etim-Ekpo, from Ikot-Ekpene to Uyo is very far. |
| Okay, we are talking about the disadvantages of decentralization. | No, no, no! |
| No disadvantage? Okay with this decentralization is there any type of changes or adjustment that you have made to make you cope with it process? | Yes. |
| What are the changes or adjustment? | Going to Uyo was very hard, you can go there and not see the Lab man, you will be calling and calling and not see him but this Lab man at Ikot Ekpene if you go there, he is a civil servant but if you call, he will say come by so.. so-. so- time. If you go, you will meet him that’s why I like him. |
| Okay, but did you make any other personal adjustment with managing the part of this decentralization? Any adjustment you made just to make sure that you fit into the decentralize DRTB services? | The adjustment is that I don’t go on my own time. I will adjust my time to go with the time of that man when he fixes, when he says come, I will adjust my time to meet the time he fixes the time that I should come. |
| Apart from that, are there any other changes? | There are many changes, but the few that I can mention is what I mention.  Okay, this component where we discuss, is there anyone there wey you want make him remain as he be, make them no change am patapata. Those our GX alert, DRTB, Counselling from DRTB Survivors, Tracking of Patients, Decentralizing Labs, Support to go for Baseline Investigation, that one of using number connectivity, you use the app, you see the test, Community Volunteer Exam Officer, is there any you want make he remain, make them no change patapata.  All of those, ones are good. But I said something, that to the patient for baseline, that we should increase transport for them, added something, not that they should remove anything. Its okay, tracking is okay, everything is oaky, but to add something is to increase at least transport fare for them not for me the supervisor but for the patient. |
| This decentralize DRTB Services, do you think its complicated or easy and simple? | It’s easy and simple. |
| What makes it easy and simple? | It is very easy and simple because everybody are going to Uyo from everywhere. Very hard before they will attend to your patient but since Ikot-Ekpene has their own, it is good. |
| Are there support that is available to help this decentralization, maybe form NGOs and other decentralization. | Well, I cannot say, I don’t know that one oh! |
| Support no be from NGOs? | I don’t know oh! |
| But they use to give money to those patients? | Okay, its from NGO, I think its you people that pay them, we see transport support 3000 Naira every month. |
| Yes, that’s the kind of support I am saying apart from that, is there any other support. | No, transport to the supervisors monthly 2000 Naira. |
| From which agency? | ARFH, I did not even ask them, they know they are supposed to give. |
| Any incentive? | For? |
| You, for patients, you know. | Except if it is that two thousand |
| Did they train your workers? | Yes |
| When | Before we started this Wave 9 something, something. |
| Where? | Uyo |
| How many days? | It was about three days. |
| Which people train? | RedAid |
| Do you think we should decentralize further or even reduce am, is there anything that is making you think that they should move it from Ikot-Ekpene to Etim-Ekpo. | Yes sir, yes sir. |
| Why did you want that? | I will prefer further decentralization. It will cut our expenses, for us and remove stress, because going to Ikot-Ekpene is very stressful, so if the activities will center at Etim-Ekpo, it will help us reduce stress. |
| So, you want it that way? | Yes |
| So, all this thing you are doing, what is your motivation? | What motivated me to do it? |
| Yes! Cover mouth, cover nose, the same bike with a patient and all that. | I said same thing when we started this, not because of the money, it’s because of the patients also, love for humanity because if the patient stays in the community for so long, he or she will infect others. This was what motivated me to go and plead with them to come out for baseline and treatment also. |
| We don talk decentralization, you dey even talk make we decentralize further, make we come to Etim-Ekpo. Are you confident that we fit sustain what is on ground now? | That one, I don’t know, is you that know. |
| Yes, from you own mind. | Yes, sir. |
| What makes you think it can be sustained? | Because you are coming out to help, so the thing will still move further. |
| Okay, okay, okay, we will tell them that, it is what you said.  What about your other colleagues, what do you think? Do they think this thing can also be sustained and they are confident? | Yes, I think so. |
| This decentralize DRTB services, how well did it fit into the way you practise at TBLS? | Better. |
| Like now, before when you don’t need to be moving people for baseline and all that, now all of a sudden you do call the pathology lab at Ikot-Ekpene. How did you treat it, or everything you started making adjustments to be able to fit in into all these? | I don’t know, how can I put it? |
| Now, all of a sudden, this is a new approach, if it before, when you are not part of baseline investigation or do patients go or you people still accompany them? | Yes, we do accompany them because some in the village, they have not gone to Uyo before, when we are going to Uyo, you accompany them to make sure. |
| And also, do you still accompany them now that it is not at Ikot Ekpene? | Yes sir, yes sir. |
| Okay, Okay | Unless otherwise, that person is a learned person; we just give address of the Royal Pathology man and his number. Then we communicate, after everything, they tell me its okay, at the end of it the man calls me for other results. |
| So what you are saying is that even when this decentralization came in, it fitted the way you do take work before, okay.  Now, do you think we should……, there are two processes, first one is the time people do go to Uyo go do test. Now, you can go to IkotEkpeneg, now do you want the one that we have decentralized, the one going to IkotEkpeneg to replace coming to Uyo, make them cancel anybody from your end coming or you want them to stay side by side? | Let the two systems be. Let Uyo be and let Ikot-Ekpene be, if I have a patient and he says that he is going to Uyo, if I explain, if he can go to Uyo, good. But, Ikot-Ekpene is closer to us than Uyo. |
| That’s what I am saying, you will not follow that person Uyoat all again? | If he is a learned person, somebody that can go, is only those that you see cannot go, cannot move on their own, we come in to help. |
| Okay, you want the two system to remain if a patient say he wants to go Etim-Ekpo, let him go. | Yes, but they will not go to Uyo since we have Ikot Ekpene close by. |
| So, your own is, you want it to come close to where you are staying? | Yes, that is what I am pleading for. |
| Is there any other thing you want to say? I have finished my question. | I want to say, thank you for your effort. Let me just say it like that, that even the effort of this people that are helping our patients, I am thanking them, they are helping us and we now it is somebody. There are people that are helping us. It is not our money and its not Nigeria’s money. We know that one, that, is helping us. I am thanking them now, thank you very much for that, but continue to do the good thing because it is the Lord that will give them reward, our own is to, if we have patients, we just move with the patient, and counsel them. Because some of them, may think that it is the end of their lives, just tell them something, encourage them, the other thing is for you to tell them to come for baseline and start treatment, they will be okay; they will be alright. I don’t have much to say, I have said a lot of things so thank you, sir. |
| Thank you! | Thank you for coming to Akwa-Ibom. I saw you at Enugu, I don’t know you will come to Akwa-Ibom. |

**Title of Interview/discussion: KII/B8**

**Date of interview:9^TH^ AUGUST 2023**

| **Interviewer/facilitator/questions** | **Respondent/responses** |
| --- | --- |
| Sir I go straight to the question like I said before we are focusing on TB wave 9 project which centred on decentralizing DR-TB services | What do you mean by … |
| Okay, there is decentralization, you before patients who are accessing DR-TB treatment need to go the State capitals for baseline investigations and for every other thing that is associated with it including initiation of treatment and all that but part of what this project did is to make it possible that some centres will be involved in the treatment. | We are doing it already here now. |
| Yes, the State Program Manager made us know | We are doing it already |
| But then that is actually the major focus of this work because I think this centre has a model already. In some other states, you must reach state capital before activity will start.  Okay, so it helped us insight in discussing the matter based on the fact that to large extent, the practise has been on-going here but we are now viewing a situation whereby there is a…, we are now comparing with the situation where everybody will be moving to Uyo for all activities concerning DR-TB. I think what your state has done have been a step forward. So, thank you sir for the opportunity. |  |
| Okay, we will just start at once; we will want to know…, what do you think of the TB reach wave 9 project which centred on decentralizing DR-TB services? | Okay, so like I have said earlier, that this facility is like the hub or the centre where we do TB activities in the whole of this senatorial district, so I think we have already decentralized even before now because we do that once a month. We have a team here where all the patients from the whole senatorial district come here and we are the one that see those patients. The State team normally joins us. So they come the one that needs investigation, the one that need….. whatever, all of them, the lab people will be here on that day.  All what the patient need is to arrive here. We review the patient, if they need other investigations, because there are some TB patients who have other problems. They have high blood pressure, they have diabetics, so if they need to do those tests on that day they will arrive here, once a month. That’s what we do. So, they come here we review them put them on treatment if they have other diseases apart from TB |
| Okay, because it’s good for me to read out some of the components in the TB reach wave 9. I think there are 9 components I will just read them out. One is devolving GX alert to include LG TBNs. Two is improving tracking of patients by supporting the global fund funded CBOs. Three is improving counselling of patients by using what they call or what we call DRTB survivors. Number 4 is decentralizing baseline investigations to more … qualified referral centres. I’m sure that one have already been, is part of what you people have been doing. Then providing transport support for patients for baseline investigations, employing mobile connectivity submission to improve turnaround of baseline investigations, verifying test using verbal autopsy. Now decentralizing treatment initiation to Local Government level and then the ninth one is engagement of a state volunteers…, so these are the way we will do x-raying to see how well they impact on the programme and for the once that you have… I mean your centre or your state have already been practising, you also emphasize because we will want to know the challenges that involved in most of this and perhaps, we may expect you to make some recommendations assuming since you people have already been practising this, what recommendation do you think you can give to the National TB Control Progrm assuming they want scale up some or most of this component to national level. |  |
| What was so fantastic about this decentralization? Let me focus on that now since you are already an experienced hand in that ever before we came. | We tried that once when we had the former the previous Program Manager on tuberculosis, there was a day he called me that I should come for training in Uyo, so everybody came to Uyo. We were overwhelmed because where we used the Divine Health Centre did not even have where to give patients to sit and It was only two of us before the whole patients in the East, so we were overwhelmed. From there we started thinking, can we decentralize this thing. So, we made a trend, you know it was only me and him inside that waiting hall so we needed to arrange so we can join other people. So we now took centre to ……..Oron General hospital where it will be cover the Senatorial district, One here and one in Uyo. We now gave them different days so we don’t clump and the staff will also not be over worked, because like you also said today, today is Eket…. We now form a whatsApp group where the, all the workers were in the whasapp group. So when we to do our OPD days…… 7^th^ of August Uyo centre district, 8^th^Ikot Ekpene, 9^th^Eket at the different centres so that when the team is moving, the team moves to Uyo on different days and join the doctor, on the second day they also move and join the doctor in the other group, the 3^rd^ day they will also move and join the doctor. Now why do we move to join the doctor because doctor will be able to identify some of the …. You know all other health staff do normal, they will just go and display, but there is a way you see a patient as a doctor and you see something… you just ask her and she will say doctor I urinated hundred times this night. At least you know that she has diabetes issue with this DR-TB so you needed be a doctor to help these different centres. So that is how we were able to decentralize but it did not also come with no problem. So on the flip side of that, a lot of these patients don’t even have transport to come on their clinic days, now the doctor themselves you can fix a particular date, and that day the doctor will have another engagement. May be if not a programme, infact many doctors are doing another programme may be online or they have exams or they have lectures, you know and so you won’t stop him from going to his lectures, that also gave us some issues. So thirdly, issues of the civil service. You know there are some staff that have been trained that have been following us. You know how civil service do when they want to transfer they will not call us say should we transfer this person so the staff that have been trained and have been following up the programme, they now transfer them and now since the programme don’t even have a way of training people again, when they transfer those people now, when they bring new people who does not even know anything about the programme, who trains them because you people are no more doing training but before now you people use to do training once a while you update people’s knowledge. You train and retrain. So when the government transfer so they now bring new people that do not know anything about TB. Some who knows even some health workers when they hear anything about TB they will not even want to be involved so how do you convince them to be involved since there are no special incentive no hazard allowance or something because some of them feel like if I should be involved in TB I should have hazard allowance which I think they concept are …, some of them will say what will I gain from that your TB, please give me another work. I don’t want to do your TB and you can’t force the person. So that is some of the small-small challenges that we have been having. |
| Okay, but is there, still in this decentralization aspect, is there anything that made you happy with that approach? | Beautiful sure now you know let me give you an example like Ikot Ekpene here where we are, ugworokara is close to…, Aba is close. So is closer than going to Uyo so patients like where that is close to them and most of them speak the same language because they like to interact with people that speak the same language and apart from that we were able to have what do you call it, treatment focal persons in this local government. All the local government have somebody a DR-TB focal person in all the local governments, so there are the one that we’ve been sending to go and check if the patients are taken their drugs as prescribed, check if there are reactions to the drugs. They are the ones that will report back to the centre. So, these people are also their people. They interact better with them so it was a very good thing. You know there are some people that if you tell them to come to Uyo, the way they look to Uyo high… you know, they feel that when they come people will come and speak the language they will not understand so they don’t even want to come. So, some of them don’t even like leaving their village, their farming, they are doing one thing or the other. So, it is convenient for them to be in their village, you send your own focal person to interact with them, they come and take drugs and go and give to them. So once a month they show up here and also sit close to them for review so is a fantastic idea, and it also help to give the health staff allowance. You know because the TB… can go round the three centres when we decentralized. If you say today is Ikot Ekpene, the programme manager can be there. Tomorrow he can be in another place, so you cannot do it in one day because he cannot divide himself to be in the three centres. |
| So, but the closeness, people were saying that the closeness did not matter much because at the end of the day stigma came in. that if they come to a place that is closer to where they live that people will now accuse or know or become aware that they are DR-TB patients | Well, I did not experience that. No patient told me that. So, I have not experienced that. |
| If I may focus on all these components which one is the most fascinating to you | There is one that we are not doing o |
| I know, I know that’s why I know that…………. | Counselling, I mean everybody does counselling |
| They were using the special touch in this is that they were using DR-TB survivors, people who have had DRTB and they are cured, you understand. So, they were brought in to be able to speak with patients before initiation of treatment | Yes that happened but was not sustained because when we came to this place what we use to do is that before we start clinic, we gather the patients, a health officer will talk with them about the DRTB, encourage them that is not anything, that there are drugs and so on, when they finish talking to them we also tell them the does and don’ts like when you cough, cover your mouth, don’t bring out stupum, you should not pour it anyhow, if you are in a house, living with people if you are coughing be careful. We do that. So, after that we now call a survivor who will also |
| So, you people have also been practising it? | Yes, we did that now even with HIV and the team we used to do that initially |
| This decentralization in the State when did it start? | Is up to three years |
| Is up to three years ok | So that is what we use to do both in HIV and even our TB. In fact,there was a time Kings Foundation took some of us doctors in TB with survivors to go and do these things that we are talking about in Netherlands |
| For training? | Not training, we were discussing stigmatization you know it was the issue of HIV, some survivors said they took drugs, they were not stigmatized, they survived and all of that so we travelled out with those people |
| Just for that purpose? | Yes, for that purpose. You know the thing was so good you know things were happening but at a time everything just died, people were not visiting again so survivors were taken. |
| Okay, what can we say affected or influenced your support of this practise of decentralization because is already up that you have already started even before this project started? | The need to decentralize, Yes. Before that we were bringing everybody to Uyo, we observed that a lot of patients will miss clinic for one reason or the other, they don’t have transport, rainfall, so they will be lost to follow up kind of. We also noticed that we were overwhelmed, overworked. The health workers were overwhelmed by having all the people in one place. So, when we decentralized patients became more comfortable. So now, patients can access these services close to where they live and health workers have less stress. Decentralization is good. |
| Okay now, I think you have touched it to an extent but we may need it for more emphasises the challenges involved in this decentralization. If you can list it out for us. Yes, the challenges that you people faced when you are initiating, during that initiation process | Okay, the number one challenge was that most of the people that we trained on DR-TB were in Uyo so that challenge of health worker who know, who have knowledge about DR-TB because you have to get people who trained to send to where you have a referral centre. Yes you have to send them there. So, we had that problem. Apart from that we had the problem of people not accepting to be involved in anything TB or DR-TB. A lot of health workers don’t want to be involved. They don’t want anything TB. Because of the stigma involved, they will say carry that your TB something and go. The third thing is that the patient also used to complain about transportation and all of that. They don’t want to come. I think basically those were the challenges that we had during that time. |
| Okay. | Another challenge was that they were some facilities that complained that they don’t have enough time so how do you now come and give us another work, more responsibilities. So now the one that had time, her staff are not trained on DRTB. So, there were all those kinds of issue that stand as constrain. Because we want even decentralize the more but we had that challenge we had to stop and we say ok let’s even use one officer for a particular senatorial district. We wanted to do further decentralization but we met heavy war on all those things and those transfer that I mentioned. Sometime it is like me now that are here imagine if they transfer me now who will do the thing because most people doesn’t even want to do because one it is not lucrative, the stigma involved so a lot of people want to be involved in something that is lucrative you know. They will say no let me go and find one CS, and make my money because doctors want to work clear cut and make money so when you say I want to come and discuss TB the person will not come out. So how to convince somebody is not that easy to convince a doctor to come and stay and do this work. |
| Okay, thank you very much. So how does decentralized DRTB service compare with the routine centralized format that you people were practising before | From my observation, it has improved the issues we use to have with loss to follow up patients we don’t really have that because by then we use to have about ten people that were lost to follow up the truth is that you will not see them again but now if you check may be one or two. So that loss to follow up is not really there again. It also helped us to manage co-morbidities because most of those days that we used to come to Uyo, all of them may not be seen by a medical doctor. Sometimes other health workers will just issue drugs and will not ask those clinical questions to unravel other co-morbidities. So, it has helped us to manage other things so that the patient who has DR-TB is taken drugs but he has stroke and die because it was overlooked. So, it helped us in managing co-morbidities. It has helped us in retaining our patients. We don’t have much patients to follow again. |
| Now I know that you have spoken so much on this matter but it means that the other questions will just be to be emphasizing on what you have already said. Okay the advantages of this decentralization process | The advantages of decentralization. One it has created a lot of awareness. If we are still going to Uyo, a lot of people here in Ikot Ekpene will not even know that there is such a program, DR-TB program,  Number two a lot of health workers now have knowledge of DR-TB. This is because when the patients come and we are interacting with them the health workers are also learning.  Now the third advantage is it also helped the patient to access drug easily without need to come to Uyo because it is closer to them. The whole point is that it also helped the patients to have interaction with those that will help them in some other conditions apart from DR-TB. Fourthly because we attached each treatment personnel to each local government, it has helped us with drug interaction reactions, adverse effect. Our knowledge of adverse effects of drugs used for DR-TB treatment has improved. Sometimes they will report patients said he took this drug and he having so-so-so. We can now so this drug used to have so-so adverse effect. It has also helped us with that |
| Thank you very much sir.Now disadvantages of decentralization | One of the major disadvantages is that it gives so much burden to that particular facility that is been used as hub site. If anything happens to that particular facility, it means that patients cannot access for that period. Do you understand now like the time I was not around so the whole thing you know, so like that time may be the patients will be going to some other place may be like Uyo. It was a serious issue. That was why I was telling the Guy “you have to train more people”, you have to train one or two persons so that if I am not around they can swap. So, this is one of the major disadvantages. |
| Thank you very much, let’s go to the next question.  Now what kind of changes did you make to ensure that this decentralized DR-TB treatment worked effectively? | What? |
| Type of changes or adjustment did you as a person make in ensuring that this decentralization process worked out very well | When I was informed that they want to use this health facility as a DR-TB center, I came down from that meeting, I called all the staff and informed them that they want to make this place a DR-TB centre for this senatorial district so when the patients come, attend to them. I informed them of all that will be needed for that development. I delivered a lecture on infection prevention and control and then appointed a focal person for DR-TB. I created a WhatsApp group for information dissemination.  Give me somebody in TB unit. There is already a TB Unit that already know about DRTB because they are in TB. Put the person in DRTB for me. So that when they are going for DRTB meeting that person will be the one, the person will be doing everything recording but at one point as God will have it all my Nurses even the health attendant started getting involved so when I go there all of them are recording something, you know everybody was just excited, you know I was able to calm down the tension. So, when they called for the meeting, I will say so-so will be coming, expect them.  You know like when you called me yesterday, I have already told my people that you are coming so that when you see a visitor you, know and I have a whatsapp platform in this office for all staff where I relate information. So they already know that you are coming. So that is what I also did that time when I got that information, I communicated with them when you see this kind of people., I also did something to make sure all the patients have their face mask so when the patients come, I make sure they have their face mask, if you don’t have your facemask use your handkerchief and cover your face. So that’s what I did and it was easy because when you carry your staff along, it will be easy for them to cooperate with you unlike when they wake up one morning, and start seeing DR-TB patients in the center, they may disregard that activity and the thing cannot flow. |
| Ok, any other one? | The next thing was that channel of communication with the State, to the Uyo now, to the state Programme Manager we were able to communicate and we were able to tell him the problem, the few challenges that I had which is that training. So sometime he used to come and do a small training here for my staff. We kept training and re-training our health workers at the local level. On some occasions I will invite the State Program Manager to join in the training. This is because I noticed that when I talk and he as one from the State Team talks it brought more confidence to the health workers concerning the program. So, he usually comes sometime I may not be around, he will call I’m in your facility I will say talk to the health workers. So, he will talk with them, engage them. So that was what we were doing, everything about these things is just get people to be involved and give them that confidence. That helped us very well. |
| Okay, now we are coming to those components are therethese components that you may want it to remain unchanged | Which of them |
| You know we have 9 components of the intervention project, is there any one that you want them to remain functional let be use that word without any changes you know I will still read them up again. devolving GX alert to include DRTB | Let me take them one by one  devolving GX alert to include DRTB |
| devolving GX alert what they wanted you know now the DRTB focal person and the state programme manager usually receive what they call GeneXpert alert anytime a DRTB case is diagnosed from the GeneXpert machine. Now even though what they did they adapted this, what they did eventually was because they wanted the AD TBNS that’s the local government supervisors to be able to be getting this alert in addition to the state programme manager when they were because you know is from all this companies that do GX alert when they couldn’t do this what they did now is that once a DRTB patients is diagnosed they place that result in the whatsapp group so all the LG-TBLS that are involved they will now know, so is like a real time communication if I may use that word so that’s the first one. | Okay |
| Do I continue or do we treat this one? | That’s the first. |
| That’s one of the components not as if is the first. | I think what I think personally is that thing is ok. The only think personally is that when that is done the patient needs to be informed. |
| Yes, you because most times, you know is the lab that are doing the test. The lab doesn’t know the patient but when the result is now put in that Whatsapp group the LG-TBLS will now call | Yes, its important they try to know where the patent is because there are some when they drop sample they travel, some when they drop the sample they will go back to village and if you didn’t follow them they will continue to say God will heal God will heal so If they know where they are you can now connect with the Local government person of that location they travelled to. So that should also be involved. |
| Ok. Now the other one is improving tracking of patients by supporting the CBOs. You know there is what they call Community Based Organizations. So what they do is to track the patients and monitor their activities. So, what the intervention did is that they provided extra support to CBOs to ensure that this is done. | So,in that case what I think is that you know if the prophet doesn’t have the honour in his home, now for those CBOs, if they don’t have honour because of where they live they are so use to them that when they talk they will not even take it serious. So I feel that the State will once a while if we have a case in the community like there was a time we had DR-TB around Gbademi in Akwa Ibom State, so I was telling them we need to go to that place, we need to codon that place and try to go inside do health awareness, speak to religious leaders, speak to traditional rulers or all those chiefs, at least when people like now when you come as you are coming now it creates more impact that it’s not only us. So, I feel like beyond that people, the state team should also join them to create that impact when they see new faces from the state team it creates more impact. |
| Okay, number 3 is improving counselling of patients, the use of the DR-TB survivors | Okay. Is very-very important but some the dangers is this patient after treatment they are gone. Just that they also convince the survivor to come out and speak |
| Thank you very much, you are even putting some of the challenges that may come up with that I appreciate that sir. |  |
| Now decentralizing baseline investigations to more peripheral qualified centres | Is one of the major issues. In short one of my major problem is that I don’t hope if there will be changes after now apart from baseline when they come for culture, I don’t think so, we send our culture that time we do not have one in Akwa Ibom State and that time we need to wait and wait-wait and wait. So is a major issue, anytime we go for the state meeting I will be the one to shout and shout even my director just charted me put my hands down but you know in my mind I was like how much is it that the state government cannot afford that we will keep suffering, so is a major challenge, that is an issue. Now the baseline investigation is a major challenge. This Ikot Ekpene a place where we do baseline investigation, Uyo, then other place should also have may be 3 or 4 locations so that we can have this result quicker than we used to have. Before now, when we diagnose DR-TB before we have baseline investigation results it will take time. This is because we use to get baseline results before we initiate treatment and by that time the DR-TB patient is still coughing and he/she may spread the disease in the community before we start treatment. So it was good that that baseline investigation is decentralized. In-fact, it should be more decentralized. Then we must have … culture in the state, all state in Nigeria should have not just 3 states will be sending to one, it doesn’t make sense. |
| Okay, the forth one is providing transport to patients for baseline investigations | Yes, I know that you provide but the patient had been complaining that it is not enough. You know that there is nothing we can do but let me tell you from personal experience, 90% of these patients who have TB are very poor, I don’t know if other people have other submissions but I as far as I’m here and from what I have seen, I used to work here alone for three years. You know I was … Doctor for three years before they send me other doctors; I was here alone for three years so anything that has to do with this TB something I know it. So, for the patients I don’t know how we will handle this issue of transport. A lot of the DR-TB patients have complained that the money given to them for transport is not enough. This is because most of them if not all the patients are poor and you cannot blame them. Almost all the patients that have been diagnosed of DR-TB are poor. So this transport matter is a real problem. |
| So, what do we do? | I think from personal experience if you go to community, you go to community you don’t work independent as we worked you have a small-small cluster may be one small group headed by one village head. So, we need to work with all those people and through the religious leaders because here they like church well-well. So, if a pastor in her community say “don’t do”, they will not do. So, you need to go to that community use the pastor and village head or whatever to talk with them you see how we can improve. This one is not even you people if Government can have their own measure on how to improve the economic status if we engage government because if they don’t have money they don’t have, if you didn’t have money to put for here you won’t have been here is straightforward. So, a lot of them complain that they don’t have money not just that they complain they don’t actually have. So, it has been disturbing us with letting them adhere to their medications. There are some months they don’t even use to send the money how small you know those things. the compliance. However, there are many other newer technologies. I hope you know that people are coming now, to deliver drug to these patients |
| Even some African countries | Yes. Bayelsa. The Ekoyinabo centre person is my friend, Bayelsa have started producing drug that they give to their patients. Newton so we can see how we can deploy drugs now, I think we cannot be doing one thing - one thing for ever. There are newer technologies so that if patients cannot come to the hospital, we can reach out to them. Okay if we cannot deploy drugs, we can have one or two utility vehicles so that if we notice that these patients. you know we can go to those patients Another thing is can we have an app that we can use to track compliance that if the patients have not come that app will give us an alarm or something that this particular patient in this so so centre, we can have that kind of app that can track to know when the patient has taken his/her drugs and when the patient stop we have an app that can repeat the alarm. It is happening everywhere we can have that here. |
| Okay, thank you. Now the next one is employing mobile connectivity solution to improve turnaround time of baseline investigations. Connectivity means that once the test is coming out is just like an alert to some people will have access to the result so that the turnaround time for that result is reduced | What does it mean?  I have complained about this one already now |
| Exactly! Exactly! | So, when they finish, is for the lab people when the result is been carried out to know where the patient is coming from, they will just send it via e-mail or something. Like if I send people to lab in Uyo now I don’t go there to collect the result again. Like took my mum’s sample to lab they just sent the result to my mail. So, people have developed now. Like my friend in US told me I need to study a result I told him, send it to my mail, he said in Uyo I said yes. So, people are also moving forward so we can deploy that. Result can be sent to where they are needed so that people can initiate treatment on their patient sooner than we use to do before. |
| Ok the next one is verifying the reported pre-treatment test using verbal autopsy, what they do in this one is that you know in the programme when they say most people die before the initiation of the treatment but there are no cases of reported dead so what they just need is verbal autopsy just to verify. Who did you say die, they talk, you just go there and confirm from the people, is not as if you are doing any real autopsy but by asking questions you verify because what they found out is that most patients may change location and the programme will report death so they want to be verifying this death. | Verbal autopsy is ……………., I have heard about it but is a very sensitive one, very sensitive in the sense that motion and all that |
| Okay assuming its through treatment that………………. | You will now go and start asking the relative how the relative died some of them will even say that you killed him/her. So is a very sensitive one. So, we don’t deploy verbal autopsy like that until you have done the proper things on the people that are going to carry that verbal autopsy done. Are we going to do it through phone call, are we going to do it physical appearing at the scene, the address of that patients so need to a lot of them that will be involved need to be trained on how to approach the relatives of those who have died and get information from them you will not go in that grieving period if you go in that grieving period you will not get anything from them so you need to know when are you going is it before burial or after burial even after, how many days or how many months. Who are you going to talk to,to lead you because there are some family members that if there are the one leading you, they will allow you but if you go directly they won’t even. So, a verbal autopsy is very sensitive, is a programme on it’s own but is a very good that we should do because it will also help us to unravel a lot of things that are the cause of the death of DRTB it may not be the DRTB itself it may be other things so that verbal autopsy will help us to unravel that, that is not just this TB that kill these patients there are other things. This can do that and it is not just one light thing, is so deep so we need to. I think is a very good way of getting information of the cause of death but we need to do it very well. |
| Ok, thank you. The last but not the list is the engagement of the state volunteer liaison officer | What does it mean? |
| That’s somebody was kept in the state to be able to reach out to the other members of the State Team in all matters related to DRTB | We have now |
| Yes, that’s the DRTB focal person but what the project did was to have a liaison officer | Yes  Who was the person? |
| Mentions name | That lady now |
| So that’s her work so she was now liaising with all members o the State Team in all matters related to DRTB | So why are we asking about the person now |
| I’m not asking about the person is about the office now, is it worth sustaining? | Now my own is that is she not overworked? She is the only person because I remember there was a time I was the one going round the state, the State Program Manager was not around so he told me to be going round Uyo, Ikot Ekpene and the other centres to see DRTB I was only seeing that particular person. You know in my heart I say is this person not overworked. The person is so needful now and she needs an assistance self |
| Thank you thank you very much for throwing good light into all these. |  |
| Sir do you see this decentralize process of DRTB services can you say is complicated or is simple enough? | Well to me I’m not seeing any complication. Is simple enough |
| What justifies your position that it is simple? | Now from my own point of view, immediately I see that message because they sent message, I am in many whatsapp group so when you still post it!Postit on that normal team whatsapp that we are all of us, you send me individual message so that I can remember and a day before you remind me that’s what I tell them all of them and what I do I call back my TBLS and ask how many patients do you have in Ikot Ekpene that will be coming for tomorrow or next tomorrow DR-TB clinic day, he will tell me, how many male, how many children. I will start preparing my mind on the number of patients I’m coming to see so it made the whole thing go smoothly as far as I’m concerned. Before me I come, I know that there are number of person I am coming to see at least when I’m arriving the clinic, so I can do other things I want to do before I come but if all of them are coming to clinic I can do other things that I want to do but if all is coming to clinic the same day it will be too much now I cannot do that if it was the whole state going to one place so it helped me. That was the thing that helped me to manage the patients and from the record that I have also seen I have seen a lot of improvement. There are lots of patients that when I see I check the weight when I will come back they ‘ve added 2 or 3kg other things, so if I have been having that kind of result, that means you use that medium to look at them more. The one that have high blood pressure but before now we don’t use to measure those things. I’m the one that started it. When I sit down here you just bring them for DRTB so one patient told me I have not been sleeping, this your drug? I brought BR apparatus and measured and systolic BP was 220. that’s why I then told them to always measure blood pressure. So that is what helped me to manage all those other things because if I have time to interact more with the patients but when they are many like that you don’t have time, you just rush but when it is decentralized the number is reduced and they have more time to interact with the patients to ask more questions why did you stop your drugs. I had even used my phone to call a patient’s because the woman stopped her drugs, she said she went to visit her husband. When she went to see her husband she stopped her drugs so when she now came back after three months, I told her you are going to restart from the beginning she said sorry I went to see my husband, I said where is your husband’s number she brought it and I called the man and said please give her some time because she cannot miss your drug for 3 months I don’t understand what you are doing so you will start from the beginning and I spoke with the husband. You know all those kinds of time and the passion that also helped me to interact more, it wouldn’t have happened if we have crowd. It won’t happen. Now, another thing I have also seen is that it helped us to know our team. So now if I just see a call now I will know is from Igborokara TBLS that is calling me, I know everybody now in my phone so if just see your call, I know that may be probably a DRTB diagnosed so it helps that team to be functional. We have a small cluster of team, where they report back to the state so is a very good thing. |
| Thank you thank you.Now so how well did the decentralized DRTB service meet the needs of your patients? | The decentralization has helped the patients have more time to interact with their caregivers. It has also helped the patient to access care easily, no complain about going to Uyo. Again, we have even been very flexible in the sense that there was a time that we fixed DR-TB clinic and they were to have a masquerade ceremony, you know from the culture of this place when they have masquerade days, they said we will not come out on masquerade days so we were flexible enough that we were able to shift to another day to help them let finish their masquerade ceremony. Because sometimes in fact you won’t come out if you are a women, you cannot come out because if you come out there will be a serious problem. In some part of this Aniakara, you don’t come out at all. I don’t know why government has not abolished those nonsense, if you allow me with those culture, I would have cleared all those things, just carry bulldozer and enter into that their masquerade playground. In fact, primary school would have been there. |
| Okay, that’s a replacement therapy now | Yes. So that’s one, you know that flexibility is there, so if somebody was coming from Sinudo to Uyo and you now start discussing masquerade of Uyo it doesn’t make sense now, but if it is us you say, how we will do it now, even the TBLS cannot come out, so we need to reschedule TB clinic fixed on the day of their masquerade. |
| Now I think you have almost touched on this one but I will still ask you.Sir do you think that there is a strong need that we increase this decentralization, either to go farther to the hitter lands or even start bring everybody back to Uyo? Do you understand so which one do you, do you think there’s a strong need to? | We should even decentralize further instead of reducing but I think for now before we do that we should train those people that will be managing the patients because is not just decentralization but the quality of care is also important. Is just like if you want to repair a car, the mechanic will send an apprentice to you who will come and spoil that car, you know make the car worse than it was. So, when you are decentralized who and who are you sending there? Do they have the capacity needed to deliver? So, for now I think it should remain where it is until we are able to train more people, build capacity and we can now decentralize further but for all to go back to Uyo. No. It never worked, it never worked. It was so stressful. |
| Okay, thank you. Now I don’t know whether you have been aware of this what support available to help you to adapt to this | To help? |
| Support either for the health workers or for the patients to help in this decentralization process. | The only thing we know is this small-small transport now |
| Okay from NGOs | Yes, from NGOs |
| Okay | Though recently, they was so much complain both from the health worker and from the patient, before now when fuel issue did not come, but this days if you give me N10,000 now to go and put in that my car, how much fuel is that now? you know it doesn’t do anything. So that transport support was a bit okay before but this fuel matter has worsened the whole thing. Now a lot of patients are also hungry that if they do not have money they will prefer buying food to eat with that transport money and forget about the care for DR-TB, that’s why I was saying that to further decentralize it will be good but there should be training of more health workers. So the quality of people that will attend to them should be taken into consideration. Because now that they cannot come because of the economic crises now, those people that would be attending to them how trained are they? So that they will not go and do more harm. |
| Okay, we’ve talked about support, any incentive? just close to support | Nothing, nothing now. I told you that before now we use to have training on TB and that makes people to be happy, but it has been very long we have not had any form of training. It is just that some of us who has been doing the work for sometime now, are the ones who try to train the health workers at the local level so if there are newer health workers included you won’t even know. But before now when there are new staff, they are trained immediately on DR-TB care. They had training on DRTB before we started but these days when we have newer staff they will just send us some whatsapp text we just read but there is no formal properly arranged training and a lot of people learn better in that training. We do a lot of sample, a lot of group work, a lot of simulation training, role play it makes the thing to sink but people will say zoom meeting, zoom meeting doesn’t … I have told them zoom meeting. There are some people that cannot understand with zoom because by the time you go for training they simulate a scenario, they do role play, they do group work, you learn better but they do not do these things again so we are just like that. |
| Okay. How do you think your facility routine setting or structure affected, did affect setting, how did it affect either to improve or reduce the implementation of DRTB process? The facility setting or culture of the Facility or the mode of service delivery, did it improve, did it help the decentralization process or did it reduce it. | It helped, you know is so easy to do that here now. Do you understand what I mean, this facility has already been known for TB care diagnoses and treatment, so DRTB was not much different. So, with the facility that has been doing TB care for years when the DR-TB treatment came in it was almost the same. I don’t know what is happening is other facilities that have not been doing DRTB I don’t know how they were able to initiate the process but here it was not difficult to implement. Here is not difficult anything TB is not difficult that is because that’s what we normally do. So, any changes that comes we quickly switch and key in. so there was no issue at all, there would have been issue if the superintendent doesn’t like TB and other staffs, They may want to get another doctor and that is where the issue would have come. I will have nothing to do with TB, what I will do is to take one of my doctors into your programme TB and I will be sending him for that programme for that period, but if me I’m part of it then no problem. |
| Okay that’s a very good one Ok, I’m still in a close one, what have been your motivation for supporting or ensuring the success of this decentralization of DRTB services | Okay motivation, I have been doing DRTB programme since, I don’t know even before I was transferred here so I have always been doing it.  When I came here, I have being doing normal DR-TB programme diagnoses and treatment and I have also being seeing good results from the treatment, you know when you see good results from what you do that is a motivation. So you see a patient who come with HIV and you see improvement there is no way you will not like to see the next patient, so the results I got gave me motivation. Then finally, when we have now decentralized DRTB, I now saw that I was a little bit relieved of that stress you know because during those days I was the only doctor here so the DRTB period I will be in Uyo and nobody here to attend to patient. The only free period was during the COVID but before then I was the only doctor here,so during that DRTB period I will be in Uyo and they will be calling me here, so stressful, you can’t concentrate, Dr.there is a patient here So, when the rationale came I will be here seeing my normal patients here till when the DRTB patient come I will also start seeing them so it makes the thing very seamless you know all those calls and all that with you. |
| How confident are you that this decentralized DRTB service will continue? The sustainability | Well, is a big issue, a big issue that I use to be afraid and disturbed because when I start hearing people’s complains, that they don’t have transport even from both the health workers and the patients that they want improvement in their transpor. So is a serious issue, if it must work that area we must be looked at. How they will go and come you know because a lot of them don’t have transportation is a very serious problem because a lot of them don’t have money so there nothing you can do, they don’t have money. So, if you can look into that they will come. Some of them are excited to come, some of them pay their transport to come, some don’t have. |
| What about your colleagues do you think they think the same way that this should be sustained? Do you think that they are aware or that they also hoping that this decentralization should be sustained? | I have not really interacted with them like that but I know that a lot of my colleagues if you mention TB the person will ask you “wetin” I go get from that, you know they will say why do you need to go and take that risk. treating DR-TB  So, a lot of them when you just mention it, they will say I’m not interested, they feel that is not lucrative so nobody wants to come and waste their time. I have refused to look at it that way, I just enjoy doing it because of the results I have been getting.. |
| So how well did the decentralized DRTB treatment services fit into your existing work processes and practises? | Very easily now, you know I have said it before is the nature of services. We treat TB patients just that we had a plan that may be workout plan that may be once a month DRTB patients will come so we try to fix it because things has been working here we know the days that are busy day our normal busy day for TB so we check those days that are not busy so that when they want to fix they will fix for those days so that we just see the DRTB and one or two more none DRTB so that it will be easy for us. So, we were able to |
| Ok | And we have staff. What I do is that I delegate here so when I came I see some people you know I have a book here I delegate a lot, different day I put the people that will clean say in front of this office so-so-so person so that when I come and that place was not the way I want it I just called that person who is supposed to keep it clean because I know the person that should do that.  So, I delegate, with the DRTB stuff I also delegate. Say okay, so-so-so staff will be in DRTB, so-so staff will be in HIV, so-so-so staff will be on this, so is easy for me if am not around they will call, I know whom to call. So, I delegate it makes my work to be very easy |
| Okay, in your own opinion should decentralized DRTB treatment services replace the already exist old model or should it just be a complement? Should it complement the old model? | I think it should complement. The two approaches, centralized and decentralized should be co-existing. Let me tell you why. There are still DR-TB patients who don’t want to see doctors that are too close to them so they want to go far. So, if you remove completely the old model those patients will not be captured. You need the two to co-exist to happen side by side so that there are patients at Ikot Ekpene here but they will want to access DRTB Services at any other place they want to go to for reasons best known to them, so you need to allow them to make that free choice. You don’t strangle patients. allow them to have that liberty of choice and there are some patients that also have doctors in Uyo so may on the DRTB they may like to see that doctor for another condition. So that if you fix DRTB on 10^th^ of August, they can call the Dr and say I will come to hospital on 10^th^ of August so that they will do both. Because some of them have other things you know Uyo is like a central….. so some of them have may be relative to visit, event to attend so will allow it so that they can also fix they are own plans to do all at once so if you remove it may deprive them of that. |
| I think my questions are through, is there any other comment you want to make on this decentralized DRTB treatment services | Okay, I think the model is something that we’ve come to enjoy. We like it so far, so far. I think is doing well but the only issue we have is that we have not been training so because the same set of people will not be the one doing it forever. So, we need to keep training so that if this people are not around this other people can still do the same job. Another thing that I want us to do is that issue I talked about tracking of treatment. I don’t how you may want to do that so that if a patient stops taking treatment,w e should be able to know on time because we used to have such issue once a while when a patient will stop treatment and stay for months’ time and be back so you know he has to start from the beginning is not that easy, so it used to pain us so we need to see how we can track that. Because all what we are doing now if the patients are not coming, we are wasting our time, we are wasting our time. So, we have to look at that process very closely to know how to get patients to be on treatment and how to identify the once that are not taken their drugs on time. Now the next thing is the issue of laboratory. The time line between when the baseline investigation comes out when we initiate treatment are always too long so how do we get to bridge that gap. So, you can have baseline done so that we can deploy certain result may be through e-mail o whatsoever. so that we can also have time to decentralize the lab. |
| Yes its part of this | Decentralize the central laboratory also, let us have the lab that we can be using to do sputum culture in the state because it also gives us much trouble waiting for result for long periods of time. So I think basically those are. Then incentive and encouragement of the people that are working in the programme. Make the people to be happy because a lot of people are not happy so we have to also make them to be happy. Let people know that this programme is being appreciated you know they will also do well. |
| I must thank you for this very interesting interview, I appreciate. Just that key word, “thank you” .Thank you, its a joyful and stimulating interview. | Thank you Dr. |

**Title of Interview/discussion: KII/B9**

**Date of interview: Wednesday, 9^th^ August 2023.**

| **Interviewer/facilitator/questions** | **Respondent/responses** |
| --- | --- |
| So, what do you think of TB Reach Wave 9 interventions which focused on decentralizing DRTB treatment services? | I think it will make the services to be more effective. |
| How? | In the sense that taking it down to the grass root will make those DRTB patients to come close to us and be able to access those services. |
| So, what did you like most about that intervention? | What I like most about that intervention is that, it makes work easier in the sense that if they come in, I believe more funds will be added so that it will also make it easier for us. |
| Is there any aspect of that intervention that didn’t go well? | Is it already on ground? |
| Yes, it has been on ground already and has ended but we are assessing it. | Okay; it has ended? |
| Yes, but we are assessing it.  So, I explain the intervention; were you in any way involved in the project? | That DRTB Wave 9? |
| Yes, that DRTB Wave 9. | Yes sir. |
| What was the level of your involvement? | I think it was just taking the DRTB patients to do Base-line investigations; taking them to OPD and then follow up; taking them to OPD as at when due. |
| Yes, that is the intervention, pulling the DRTB patients from Uyo to Ikot- Ekpene; to Eket that was what the project was.  So, do you think it came with challenges? | Yes. |
| What were the challenges? | So many challenges.   1. Some of our clients find it very difficult to travel from Essien Udem to Ikot- Ekpene, so, transportation was needed. 2. Another thing is that some of them cannot come down to the facility so we used to take drugs to their posts to make sure that they follow all their treatments so that they will not be any lapses. |
| Okay.Any other ones? | No. |
| Okay.So, let me discuss some of the components of that intervention, so that it will help us to deal with questions.  These were the activities that were included in that intervention.   1. Devolving GX alert to include local government supervisors; were you getting any alert? | Yes sir. |
| So, that is part of the project; so that supervisors should be in the know because by right the GX alert did not include all from the way they positioned it from the beginning; they did not include the supervisors; so, they included DRTB Focal person and even the state program manager; so, this one do you think it is good? | Yes. |
| You think it should continue? | Yes, it should. |
| Why should it continue?  As a supervisor, how did this one help you? | It helps in the sense that they said if those ones cannot come, I can fuel my bike with just a token and take the drugs to them. |
| We are talking of even before starting the treatment and even diagnosis, because they were doing; were you part of that WhatsAPP group that any time there is DRTB treatment they will put it in that WhatsAPP? | Yes sir. |
| Is part of this project. | Okay. |
| So, how do you see that one? | Is fine. |
| It should continue? | Yes. |
| Why do want it to continue? | Because it makes somebody to know it earlier, they are times when we dictate it; initially it takes time but through the help of the whatsapp group immediately it is diagnose; it will be posted in that whatsapp group and we know it and follow it earlier instead of doing it pending. |
| Okay.Do you think there will be a problem in implementing this, even on the national scale? | “Long pause” |
| Like if they want to implement this in the whole country; do you think they will be a problem that will be associated with it? | Since it is a body, I think it will not. |
| Okay.   2. Is improving tracking of patients by supporting the global fund supported CBOs.   Are you aware of the activities of the CBOs? | Yes. |
| What did they do? | CBOs? |
| Community Based Organizations; they help in tracking patients. | Okay. |
| Do you think that is good? | Yes it is, because we ourselves cannot be able to do the work without their help...alone! |
| So, you think that one should also continue? | Yes, it should. |
| 3. Is improving counselling of patients using DRTB survivors; are you aware of that? | Yes. |
| How do you see that project from that angle? | Because been a DRTB patient that survived DR-TB will encourage the other person to know that people suffering from DRTB are likely to be saved if only they adhered to the treatment. |
| Okay.Then, would there be any problem in scaling that up? | I don’t think there would be. |
| Do you think we have enough DRTB survivors to fit in into that assignment? | Yes. |
| Like in your LGA; how many DR-TB survivors do you have? | I have up to eight (8) of them. |
| How many of them featured in this project? | I have two (2) of them in Utubong. |
| Two featured? | Yes. |
| So, you are convinced that this is a workable formula? | Yes, it is. |
| So, it could be helpful? | Yes it could. |
| In what way do you think it could be helpful or it helped? | Like that site in Utubong will come to this facility any time we have DRTB services; talk to DR-TB patients, also hehimself was the one that was always monitoring the treatment; takes the drug to them and counsel them like “it is always fine oh”  “it will make you to be fine” and you know in a situation like that; it makes others be fine and be open in free communication. |
| How many times do they come in contact with the patients throughout their treatment period? | The survivors? |
| Yes. | It was up to 4, 5 times |
| That is for each patient, how many times do they come in contact with each patient along the treatment period? | It should around 3 times. |
| 3 times? | Yes. |
| Okay.So, you think that is a very workable formula? | Yes. |
| Okay.   4. Is devolving Base-line investigation; decentralizing Base-line investigations to peripheral centres; like in Eket, Ikot-Ekpene, how do see this one? | Fine, because it makes it closer; initially we used to pay transport to Uyo but now we can pick up a bike down to Royal Pathology and do the test and go back. |
| Okay.Were there problems associated with this? | Apart from transportation; there was no other problem. |
| Do you think that the labs. in Eket, Uruan and co.; do you think that they were standard compare to the one at Uyo? | It is still the same lab. |
| Were they doing all the tests that are needed? | Yes, they do. |
| Okay.You feel also that this one should continue? | Yes. |
| 5. Providing transport support for patients for Base-line investigations; how wonderful was that one? | It was fine; it was okay. |
| It was okay; so, it should also continue? | Yes, but should be added or increased because now things are very hard.  I think the transport should be added up to seven or ten thousand Naira. |
| Okay.   6. Is employing mobile connectivity solutions to improve turn –around time of Base-line investigations. | I don’t understand oh! |
| This one was the way the make the investigation results available to people immediately the results are out? | After they diagnosed? |
| Yes, I think they have focal persons they sent rather than waiting or going to the lab, so they were able to send result immediately; how do you see that one? | Fine. |
| So, all of is fine; is there any one that didn’t meet up to your expectations? | No. |
| Okay.   7. Is verifying pre-treatment; reported pre-treatment tests using verbal autopsy? | Okay. |
| Are you aware of that; you know a lot of patients died before they start treatment? | Yes. |
| It was fined out that most of them didn’t even die; so, this verbal autopsy is going round to check; if they said that they didn’t die; somebody will and check if it is true that they didn’t die; that is why they called it verbal autopsy not autopsy that they conduct on the dead persons. | Okay. |
| How do you see that one; did it help in any way in this project? | Since we did not experience it; we did not have it here. |
| 8. Is decentralizing treatment initiation to local government level; that means making patients to start treatment at the local government level; how do you see this one? | Fine; it is very fine. |
| 9. Is engagement of state liaison officer who is a volunteer; did you worked with the volunteer? | “Long pause” |
| Are you aware that there was a volunteer during the TB Reach Wave 9 project? | I don’t know.  Angel love for babies? |
| No, she is your friend and her name is Aniebeth; do you know her? | Yes I know her. |
| She was the volunteer; that her work.Did she work with you during that period? | Yes she did. |
| Did she introduce herself as a liaison officer? | Since we have working together; one-on-one;so.... |
| That was her work.So, which one among all these approaches is the best one? | In all? |
| Yes. | 1. Involving the TBLAs in the program. |
| In the GX alert? | Yes. |
| That is what you ranked no.1? | Yes; another thing is making it easier for the DRTB patients to go to IDH without going to Uyo.  Another is notifying him that this person is a DRTB patient for the patient to take drug there immediately. |
| Okay; let’s continue now; is there any of this project you think that have some challenges if at all we are going to implement them? | I don’t there is any that have problem; they all straight forward. They should all be carried along. |
| Now, we are comparing DRTB treatment services whereby patients don’t take treatment at Uyo but come to Ikot- Ekpeneg, Eket and Uruan with the old method that everybody was moving to Uyo. How do you see the two; can you compare the two? | There is a very big different because of low transportation; like mine all of them use to come because the transportation is not much. This is going to Uyo for treatment and coming back with the transport will not be enough. |
| Okay; okay.Any other thing? | “Long pause” |
| Any other way to compare the two? | And another thing is; doctor assessing them as well also gives them confidence that they have somebody to confide on. |
| Okay; okay.Any other one? | And another thing is that this DR-TB treatment drugs are also brought down at the OPDs; so, it is also easier for them to continue their drugs; to adhere to their treatment; “not saying my drugs have finished” so, the TBLS can now go and pick the drugs for them to make the whole easier for them. |
| Okay.What are the advantages do you think this project came with?These options what are the advantages they came with? | The advantages are that; it is close to the facility. |
| Okay, | Another thing is that it builds good relationship between the patients and the health care providers as well as the doctors. |
| Okay. | And there is also a close communication because they are free to communicate anything and any problem. |
| Okay.Is it okay or want to add more? | It is okay. |
| Okay.Did it come with any disadvantage? | Yes; it comes with disadvantage. |
| What is the disadvantage? | Transportation. |
| Even to the nearby facilities? | “laugh”...because all of them don’t have money; like one patients we have in Okon; from Okon down here takes one thousand five hundred Naira (N1,500.00), the day they come to see; the distance is far; is one thousand five hundred Naira coming and one thousand five hundred Naira going back; so I think that could be the challenge. Another thing is that sometime, the way the drugs do torment them at the first time of treatment. |
| How does it torment them? | It makes the vomit, some feel dizziness. It makes them to vomit easily.  Immediately after the first line of treatment which is two weeks, they will be fine. |
| Thank you.  Did the decentralization helped in that one? | Yes, it helped. |
| How? | In the sense that the way they feel might make them come fast to the facility instead of going to Uyo. |
| Okay; what kind of changes do you need to make to make sure that this decentralization process work out very well? | Changes? |
| Adjustments you needed to do? | For myself? |
| To yourself, your office and your facility? | The first one is Punctuality to service; coming to work early so that any time any moment they see me.Another thing is if the intervention will be able to create a better place so that if the come they make themselves comfortable; you can see this place is hazardous. |
| Come again? | I said if the intervention can; one thing there is for me to come early; that is the adjustment so that any time they come they will see me.  Another thing is, if all of us here are sitting in this environment; I think you people should come and create a better place for us so that when they come, they make themselves comfortable; although they always feel their PPE but there are those ones that may not be comfortable with it. |
| Okay; is that the only adjustment you people made? | Yes sir. |
| Now, these components I have listed for you which are nine in numbers; which one do you should stand the way it is; that government should just buy it and continue with the same thing without changing it? | I think the nine should remain the same. |
| The nine should remain the same? | Yes. |
| All the nine? | They should; I think so. |
| Do you see verbal autopsy as the same as DRTB survivor counselling a patient? | Yes, because it motivates them. |
| No; which one do you think should stand? | The DRTB survivor counselling patients should stand. |
| That is what I am saying; are there of the same rank with verbal autopsy? | They are not. |
| So, which one do you rank the best among the interventions? | The DRTB counselling. |
| The DRTB counselling; why? | Because it makes the patients to have confidence that if this person is fine, that I too will be fine. It is just like somebody having HIV now and the condition; maybe the person is in stage three or four; if she come across another HIV person that says....”ha my condition was worse than than your own but today see how I am today; I am fine” the only thing I can tell you now is to continue to take your drugs; there is nothing that will make that HIV patient not to have confidence that one day I will be fine. I think that one should be number one for me. |
| Okay; thank you.This DRTB intervention;thisdecentralization; do you think is complicated? | “Long pause” |
| Or is it simple enough? | It is not simple. |
| So, what makes it not simple? | Seeing it; I think it is not simple. |
| What makes or what made it not simple? | What I think is that ......”laugh”....I don’t know what to say.....”laugh.....laugh....laugh” |
| Okay.So, how well does this decentralized DRTB treatment service meet the need of your patients? | It meets the needs. |
| How? | It makes that patient to know that “my drugs are there I will take them” and it makes it easier for them to know that these drugs they are not buying them; that they are free; so, giving out such drugs for interval of ten months free makes it more reliable. |
| Okay; any other thing? | No. |
| Okay; now is there anything that you think that can make this decentralization to continue; like going beyond Ikot-Ekpeneg? | Please come again? |
| Do you want this decentralization to continue beyond this level? | Yes sir. |
| You know now, we just have three centres; Uruan, Eket and Ikot-Ekpeneg; do you want it to beyond Ikot-Ekpeneg now? | Except it will be local government by local government because my people will be paying transport if that happens. |
| So, you want it to be further decentralized? | Yes. |
| Why? | Maybe making it easy for them; for instance, people from Oburu-Okara to Ikot-Ekpenen; they find it difficult, so if they can stop it here Eserendi, people will move from Abark, Ika and join them here, it is closer now. |
| Okay. So, you feel it is better that way? | Yes. |
| What do you think that will help? | It helps them to save transport cost. |
| Any other thing? | “Long pause” |
| Any other thing? | No. |
| The patients that are on this decentralized DRTB treatment services; are there supports that they receive maybe from NGOs or Agencies? | I only know of “Angel for Babies” |
| That is CBO? | Yes. |
| What did they do? | Whenever they go for DR-TB clinic; at the end of the day; maybe after two months; I don’t know but they will receive the alerts. |
| Okay; they will pay them money? | Yes, they pay them some money. |
| Apart from that; is there any other support? | I don’t think there is.  The only support I can say is that the team always come around to see them; to see how they are feeling but no other support apart from the one they have during their OPD. |
| Okay.Any incentive? | For them? |
| Attached; like you talked about support; any form of incentive: training? | For us? |
| Anybody? | No. |
| Okay; how do think your facility culture or setting affected the implementation of decentralized DRTB treatment; maybe either making it to work better or not to work better? | Since we are using the same language; it helped. We use the same language and punctuality to duties. |
| These are the aspects that helped the decentralization process? | Yes. |
| Any other thing? | I don’t think there is any thing again. |
| Okay; what has been your motivation in being part of this DRTB treatment services; the decentralization that we are talking about? | My motivation? |
| Yes, what has been your motivation? | If I see my clients; all of them because I have about eight of them even those that on treatment get cured and fine; that motivate me.  And, also seeing the state team on routine supervision or visit; coming to see them; having concern on them; giving them those free drugs; giving them that token transport; they motivate me to do the job. |
| How confident are you that this program or this decentralization will continue? | The confidence is that hence it is team work and the team has come thus far that they will continue with it. |
| So, you are confident that it will continue? | Yes; to help the masses as they are doing. |
| How confident are your colleagues that this thing will also continue? | They will appreciate as well if it does. |
| So, you think they are confident that it will continue? | Yes, they are confident; are you not? |
| Because you know that; is alright.  How well did the decentralized DRTB treatment service fits with your own existing work processes and practices? | Since we are health care workers and we are already in TB unit; everybody, let me say all of us have TB...or how am I going to put it?  All of us are immune to TB except it materialized; that is doing the job of TB makes it easier so that you will be able to see both those ones with TBs and DR-TB when they are diagnosed through geneXpert. |
| Okay.Now, this decentralization; do you want the old method of going to Uyo be replaced or do you want it to say people shouldn’t go to Uyo except people within Uyo as well everybody around here should be staying Ikot-Ekpeneg or do want the two to continue the way they are? | “Long pause” |
| Do you understand? | No. |
| Do you want the old system scraped for the new system to continue or do you the two of them continue going side-by-side? | But how can somebody stay at Ikot-Ekpene and think of Uyo for his choice of treatment; you need to go to where is closer now. |
| Do you know whether people are stigmatize when they are diagnosed of DRTB positive? | That one is true sha. |
| Has there been any case of that? | It depends. |
| Or are you aware that such a thing could happen? | It could. |
| Have you heard any experience of such? | Though I have not had any experience of such; sha! I think I had once experienced such; that the family was like “DONT SHARE THINGS WITH BECAUSE YOU ARE COUGHIN” though they don’t know it was a DRTB case but because of that persistent cough; the person was stigmatized; kept him in a particular place; they did not share things with him till after the interval of three to four months that the cough reduced and he was fine. |
| So, which one do you prefer; or do you want them to be going side –by- side or everybody should go where that is closer to them? | I think everybody should go to where is closer to them. |
| Why? | Because it is more economical. |
| Okay; apart from that? | It saves time and the stress. |
| So, you prefer the old method to go? | Yes sir. |
| Is there any other thing you want to tell us about DRTB Reach Wave 9 intervention project which centred on decentralized DRTB treatment services? | Okay; what I can say first is; let me start by thanking you people because you tried; yes implementation is also about caring of others; it is not easy; first, thank you very much.  But another thing that I can still add here is that; sir please, you people should look into their own transportation because these people find it difficult to feed, let alone taking the drugs; so if by any way through the state PM to support so that their transport could a little bit better than what it is today; it will be really appreciated. |
| Thank you.Any other thing? | Another thing is if they can be giving them token because there was one program that came; they called them “Brookly” during that time when they came newly; if they see the patient; though they were basically for under age HIV clients; they will go there with beans, rice and other things as token to those patients. |
| So, you want such to be extended to them? | Yes sir; to be extended to the patient. |
| Why? | Because, some of them when I went there, was it last week; according to him he has not eaten for the past three days; so, I have to give out money from my own bag for them to go and buy food for the children; so I think they should be supported. |
| Okay; any other thing? | “Long pause” |
| Alright; so, thank you very much for your time. | Thank you sir. |

**Title of Interview/discussion: KII/B10**

**Date of interview: 8/8/2023**

| **Interviewer/facilitator/questions** | **Respondent/responses** |
| --- | --- |
| So, I will start at once. So, what do you think TB reach wave 9 intervention is to this centralized DRTB services is? | It's a very good policy. It's very-very good because before now we knew when we had the DRTB they just take it to you like that, but now when they come, they access care very close and it makes the work very-very easy. Sometimes on the part of the patients, because we have the problem of bringing them to a central point but now that it is closer, it's very-very simple for us and for the patients to access their treatment and even for us also. |
| What aspect of that project fascinated you most or did you find much interest in? | In fact, it is all, I can't single out one particularly |
| Can you single out one you may call the best approach for you that you picked interest in? | Okay, the very one is, no. 1. Changing the cost of that treatment because before now it was very-very difficult to manage the patients on this inject table so some patients before the end of the treatment they will just abscond, so they came up with this policy that is an oral treatment that is very-very simple and as well, there are some critical conditions that you move the patient to a treatment center with that, the patient can comfortably come out of the house to access treatment at a very centralized place which reduces the cost also. |
| Okay,if we focus on the components of the intervention which one did you pick interests the most? | Like the components, can you through more light on that? |
| Okay, then we will come to those components but let's see the whole activities, you know there was a GX alert, there was a provision of state zonal officer, there is a decentralized gap and then support to get lab services, and also support to get CPU for parking of patients and then there is patient counseling using DRTB consent form for DRTB survivors. | Yes, well,let me put it that in our area I think the patient support is upgraded because I can remember vividly that somebody told me that this is my cough is it not the one they use to pay people money so I told him no, it's not that your type. So, you know, somehow like the patient we have before now, sometimes when they collect that stipend, they are so happy, and that makes a lot of them adhere to their treatment. And that component was so good, the GS alert equally, it was so good that before now, before you had a patient now, they are getting the information was very difficult but with the GS alert, it was very-very possible so that you can just within today abs and tomorrow everybody will know what happened. |
| But I've learned that since the project stopped the WhatsApp activities stopped, why? | I don't know why because we are not the people who managed or initiated it, so that is it |
| Okay, so what aspect or what component of it don't you like most? | Okay, since all the components are beneficial because there is no single one that was meant to conflict with one another. All the components were good, the only thing is it is not sustainable like the GX alert you just mentioned is not ongoing now. |
| Ok, so what affected or influenced your practice of this Decentralized DRTB service? | Well, the issue is distance especially in terms of taking the patients to llugbe's line, in fact my greatest bump string is in that line because like we in Mgbo LGA like as I come today now, coming here now I will spend something like 4,000 Naira and that time fuel was not like this but now fuel price is setting us up. The only challenge we have is bringing the parent, sometimes the relative, some families are educated which they will not allow you to go along with the patient, sometimes they will like to come and know exactly what you intended to do, they send someone to come around to moving patient from the home down to Oron before you now come here is a very big challenge to us because sometimes even if the program sometimes subsidize their transport it's not enough, so that's the only issue. |
| So that means some could say that it's even people like you that will appreciate the decentralization process? | Well, yeah |
| Okay did it come with some challenges? | The challenge as I mentioned is that one |
| No, I mean the decentralization itself did it come with any challenge?  What about the peripheral lab were they challenges? | Okay that one like the peripheral lab the issue is that we at Mgbo LGA we have our lab, and we have a truenatmachine the only challenge is that we need a geneXpert machine that is not a truenat because the lab person has been complaining. Having conducted the test you need to do another one to roll out the least tag so it takes some time and then another thing is getting to a peripheral lab here now like Oron because we have been making use of Oron. But now you see Oron is jam-packed with so many samples which like the turnaround time in the lab has caused a lot of issues |
| So what challenge are you hitting at? | So now had it been that we have a similar lab like that in Mgbo LGA because now in Oron it serve like 5 LGA including Sliket and sometimes Eket but here it would have been easy for us to do our own there, although some other will join us, we get our result and then the turnaround will reduce |
| Okay, so how do decentralized DRTB services compare with the old ones where things were centralized, can you compare the two? | Comparing the two there is a great difference, the first one before you have OPD, let me start with OPD, before you bring the patients, the OPD was to bring the patient down here to you or eket, but coming down to decentralization policy it's done in Oron so the distance barrier has been completely reduced. Another thing is even the lab, I think I could remember I normally bring my sample to that Teaching hospital so with the lab in our side and this one it helps us and the patients’ treatment equally reduces. So, there is a great difference. I can say with the old pattern it was difficult, then with decentralization it is simple just to put it in a very short sentence. |
| So, what are the advantages of the decentralization process? | The advantages are one: it saves time and it cuts down some kinds of barriers and some things like the lab you can walk in easily and do your test and come out. Like that GX alert it does help because once it drops in everybody gets it instead of waiting to be doing a lot of calls and ends up being channeled to lack of network and all those things. So, it is very simple and even the patients themselves feel it because sometimes some of the patients are allergic to all these distances because when you put their health, you compare health the way they take treatment but now I think it maximize everything. |
| Did it come with some disadvantages? | The disadvantages? Well, for everything that has advantages there must be disadvantages but the disadvantages are not much. |
| Can we know one or two disadvantages it came up with? | The only disadvantage I can say is only that the program does not sustain the people. For example, I will still mention that GS alert was very ok and another thing is that since the section of that decentralization policy, there is nothing like reflection, mentoring, because you know we have so many things on our head sometimes we even forget all those things. So at least there should be a re-training like that |
| So, you would havepreferred those components to be sustained beyond the intervention period like the GX alert that you are talking about? | Yes, it's supposed to be because even moving down to Mgbo LGA you know what happens with immediate effect. |
| Ok so that's what you're seeing as disadvantages now the nonsustenance of those components, ok?  So, what kind of changes did you need to make for the sake of this decentralized DRTB cure? | Thank you so much, I think what I need to do as my own contribution is on the part of bringing the baseline test. Like we in the zone, we have a DRTB patient and you still need to bring the person down to you for baseline. I don't know if the program can equally look at it. |
| Even with the decentralization? | Yeah |
| That's part of the challenge, inability to find a lab there to serve in your own zone. Ok, you're seeing it as an issue? | Yeah, it's an issue Because you're still moving the patients down to Uyo for treatment, it's one of the challenges also and I think the possible solution is to suggest a lab of that nature to be sited on that side to subsidize the long distance journey. Some of the patients like I have one like that refused to come but the other one I managed to pay from my pocket. Although the program subsidizes it, it becomes a problem if the lab are not closer. |
| Any other changes that you made? | You know in finance I know but personally I wasn't in support of placing the patient on a monthly stipend. That is not enough because like that some of the patients were after the money and not the treatment but I think a little support still goes to them. |
| It's important | Yes, it is important to support them because most of them will tell me that your drug makes me too hungry.  And like I've said the lab also, the condition of the lab, power was the problem of using the Truenat machine and we installed the solar light then the issue if power is gone. But if they upgrade the machine to GeneXpert it will be ok.  Another thing is in Mgbo LGA complete two wards are in the river line with about 20-something communities which sometimes we can't go to because no provision of these funds to access there so it's another challenge. I think in either quarterly or periodic support which we can go and do a kind of sensitization which will give us another long way to go because me personally I know that we have patient there which are not traceable |
| So okay, are there components of this intervention that you would want it to remain as it is that don't need to be changed?  You know we talked about the GX alert, patient personnel using DRTB survivors, decentralization of that lab and whatnot, and then the position of a state liaison officer, so which one would you want to remain without being changed? | All are essential. Instead of changing, I will advise them to do a little adjustment like that the engagement of DRTB survivors was very very good because it helps the patient at least the current ones to access it. But the fear is that survival is another problem because something must be done in that area because those that were treated and know they were cured, how can we carry them along so that they may be part. So that anytime we have a new DRTB they may be involved in it to be there. And another thing is, like to mention counseling is very very important. I think that one has been sustainable till now. Before any DRTB patient is enrolled, you need to counsel him. So, all the components are important and I made all of them to be sustained including the GS alert. |
| Do you think that the decentralized DRTB components is complicated? | No, it's not |
| So, it's simple enough? | Yes, it's simple |
| What informs your position as simple enough? | It's simple enough but I'm still at the casing, if they do it like they do in DSTB treatment combining the drugs so that it will not be so much I think it will help.  Because there are some persons once they remove this and the pills are so much they will be afraid to take the drugs and another thing is, I know they are equally on that, engaging the training supporters like we used to do is equally important but how do we know because sometimes we are not spirits to be there to be sure they are doing the right thing, their role as specified. |
| So, you're saying that the decentralized DRTB services is simple? | Yes, it's simple |
| Okay, So, how well did the decentralized DRTB services meet the needs of your patients? | Sir can you come again |
| How well did you meet the needs of your patients? | It is met because now it is closer. The patient may comfortably at the comfort of his own house may come out safely. Sometimes, personally, I am somebody that used to stay in the field for a long time, I have a motorcycle so that sometimes I may visit them and doing that it's simple because before when Ijokere was there I know what I lose but with this one now there is no complication |
| From your own view or from your Local Government view do you think there is a strong need to further increase or reduce the decentralization process? | To reduce it? No |
| No, what I mean is, increase or reduce, okay you're in the………. | I can only say in terms of the pills that they are given |
| Decentralization is what we are talking about | Okay decentralization, so there is a need to increase it. |
| Increase it so it should go beyond 3 zones | Yes |
| Why do you need that? | Because we in Mgbo LGA we are in the river line setting and we share International borders with Cameron. So, we have an influx zone with people from Ghana, Cameron, and Fernande po, like that. where we are now my focus is to extend to Ibaka which is the border line because that is the most populated community in our local government. So, I think we should increase it. Although the workforce is equally another problem because even if they do it, I think they won't equally see the need to engage man power. |
| Ok, what support do you think is available to help you adjust to these decentralized DRTB services. | Yes, is it for? |
| NGOs, anywhere | Well, I know the NGOs like those working without the effect are now Echews. I could remember when I had an issue like that, I approached the HS there and I solicited for money when there was no fund to move the patients down here that should have prone the issue because they have Keke to move the patients down to here but there's a limit that their Keke should load.  But now we have written a lot of complaint to the Local Government, the Local Government are adamant but for when those people the Echews had fund they needed to support us at least carry out like taking the person down here for baseline, I think that was once and since then when I went back again the lady was telling me there is no fund for that again so there's no support again and you know you cannot even surcharge the patient because you know they don't even give. |
| So apart from that AQs, are there other NGOs that supported the DRTB treatment in your LGA? | There's no other person |
| Were there incentives? | No Incentives |
| So how do you think your fascinating culture setting improved or reduced the implementation of this decentralized DRTB treatment? | Our facility, well, with what we are doing we are trying to test as many people that came up with the history of coughing like that and to support the decentralization that we were able to do in accordance with treating it very well. Most index cases that we have we have been able to trace them and comb the environment to ensure that nobody is left there. And equally, we maintain, we notify, sometime we mentor, we do undecide method for our staff because some of them will be like what happened in another facility not even in my base, and when they went there for supervision and I saw a woman coughing directly to the health worker I just ask madam how long is this your coughing before I know she say 2 weeks and the health worker was interrogating with the woman, because in my bag I had a spectrum cup so I collect the sample and I went to sample it without them, the sample came up positive, high range. So, I went back because I got the address, called the woman and placed her on treatment and I now have to mentor the health workers and say see what you're doing in front of your patient and she coughs like this. So, I told her that any issue of cough should be reported and get the sample for me. I think by doing that we tried to increase the high level of our own suspicions in the service of DRTB. |
| Ok, let me now look at the location of your LGA, did it support the implementation process or did it hinder the decentralization of DRTB services | It did not hinder anything,it did not destroy |
| So it enhanced it? | It enhanced it |
| Based on the distance? | Yes distance, it enhanced it |
| Also, you can say your LGA is hard to reach so to say? | Because like I told you before, two communities completely, you can't access it on foot like this, you need to enter the boat before you reach the community. And the other one is on the high sea, and the cost of leaving Ibaka to that side is about 2,500 naira by boat. So, it's an hour drive by the sea. I don't know because the challenge of getting to that place is still a big problem |
| So that means the location of your LGA favours decentralization? | Yes, it favors it |
| Ok. So what has been your motivation for supportingand ensuring the successful implementation of this decentralized DRTB services? | Actually, the motivation I have, like I said before, the distance was completely reduced, fine, so it helped me and equally the patients reduced the problem we face. It helped equally all the patients also to get well as soon as possible because it being their services nearer. |
| So how confident are you that this decentralization process could be sustained? | Well, that one had it been that our government shares in the problem we have, it should not be a heavy thing to the partners because that's where the issue lies. I am confident in the fact that they all know what they are doing but how I wish they would engage the Local government chairman because on our part we have tried to do that but these people I don't know what to say about them. It's like they don't get the picture of what the partners are doing. I think the government needed to be fully engaged in this so that this would not be what happened to this state some months ago was so awkward. There was an out stock of immunization vaccines in the state but it was either in a zone or so, for a state to give money so that they can go and convey those vaccine down to the state for onward distribution to the LGA and they said there is no money and this thing was out of stock of immunization for like about 2 or 3 months, yes, it was the banners that still go and approach that houses down. So, it was terrible, as at the time banners you know, let them know the rules we have in this state, after all what we are doing is their people, yes. And what they don't know is that TB anywhere is TB everywhere. Their family is not affected, if this family is affected, their own will equally be affected. So, I think they should own up to these things. Anything like these political commitments will save the issue. |
| So, you think once they start exercising ownership it will help? | Yes, it will help because they on their own would be to sensitize. There was one chief, I think I've never seen a chief like that, about 2 months ago who was telling me that somebody had cut a wound and the person was there in their house treating it using these local herbs. Before you know it, the person died. So, the chief has to come to the hospital and meet with my director to say that he wants to collaborate with the facility to help in sensitization like that in the community. He wants the hospital to subsidize the cost that if you have any issue like that you should report to the facility. So those are the type of people we are supposed to have, at least I would have loved to tell the people around for there own good. |
| If I may go back to that question so you're not confident that this will be sustained in decentralization? | I am not saying that, I know it will be but a tree alone cannot make a forest that's my opinion. Let them equally push also, let them play their own parts at least if they are engaged with the efforts, they are doing it will make it a success and it will be sustained. But if the program wishes to I know they can equally do it. |
| For your other colleagues minus yourself, do you have that confidence that this DRTB treatment services decentralization is sustainable? | Yes |
| Okay you're sure of the position of your colleagues and not that of your own? | Yes, I'm sure of my own and I'm sure of them also because most at times we have been discussing on this |
| Ok, so how well did the decentralized DRTB services service in or fit with your existing work processes and practices? | Well, it is hectic sometimes but everything is passion and some people that came to the program because of money. If you put the money first you will not do it. So, we need to integrate it because what is really interesting is somebody that is like you suffering from a sickness that can be taken care of. You just leave it like that because of money, no now. I think if we did not integrate it, I will do it willingly and passionately |
| But did it fit very well into your work processes? | Yes, very well, it fits |
| Okay, but how did it fit very well? | Well, it fits very well because that's our own call of duty. That's what we are trained for and we have passion for it |
| Okay, how do you want the decentralized DRTB services in place or compliment the old system? | No, I don't like it to be complimented |
| It should be replaced, that's the old system should be replaced? | The old system should be replaced, then the decentralized one should remain |
| Okay not compliment anyone? | Yes |
| Why do you want it to remain? | As you can see it's simple compared to the old one so it makes it possible even for us and even the patients. So, I like it. if there is something they need to do they can upgrade it instead of collapsing it. |
| Okay, so is there another thing that you need to say about the TB wave 9 decentralization of DRTB treatment and services? | What I need to say is that it's a very good policy like I said it helped our people, it even helped us TBLS. So, my little contribution I have to say is that those areas that need to be amended |
| What do you mean, which area do you want amended? | Like the establishing of a lab close to our people and another thing is providing funds, little funds for hiring machines. I don't mean us because we are doing our work and another thing is frequent or periodic mentoring or on site training of the health workers at least should be sustained.  Another thing is you know this thing was meant for the health officials in the TB sector, I think other health workers need to know all these things also, they need to be carried along. |
| Okay, is there any other thing that you want to add? | Well for now during the work we need the system to be sustained. That is, you know what you do well, like they say to whom much is given much is expected we are trying our best and let the program equally try to sustain it if available. As you can see the world is changing, as you can see people are doing a lot of research and serious intervention innovation this innovation can be added to upgrade the system. |
| Thank you very much.  So that's the LG TBLS from Mgbo Local Government in Akwa Ibom State. | Thank you Sir. |

**Title of Interview/discussion: KII/B11**

**Date of interview:Wednesday 8^th^August 2023**

| **Interviewer/facilitator/questions** | **Respondent/responses** |
| --- | --- |
| Sir, on the overall, what do you think of the TB reach wave 9 intervention to decentralize DLTB services | The TB wave 9 to decentralize DR-TB service is a welcome development in the sense that, it has helped us and reduced the rate of going to Uyo for example. It then helped to fix a centre in the three senatorial District, now decentralization has helped us, help the patient now the economy which is so biting so that the patient can come to a particular facility and get the treatment at the right time than bringing all the patient to one facility sometime the patient will not come because of distance, so decentralize of TB wave 9 is very good, is a welcome development. |
| What is the most fascinating aspect of this TB reach wave 9 | The… |
| What is the most interesting aspect of it | The most interesting part of it is that they have tried by at least giving the patient some transport money and the supervisor also get their three thousand. Sometimes when I will sit down I will say your account has been credited with some amount. Is a welcome development. That is incentive. That is a motivation on their part even though you are collecting your salary or whatsoever, then any additional money is incentive, is motivation. |
| Thank you sir.Now where there any, was there any bad aspect of this intervention?Any no side, let me use the word no side | No, I don’t see any bad side. |
| It has not, did everything work very well all through? | It worked all through provided you know, we the helpers sometimes fall the programme because of our attitude, because of the way we do things. If we work according to the laid down rule, there is no problem. It’s okay. |
| What affected or influenced your practise of this decentralized DRTB services?  What in you made you to support it and ensure that it worked | You see even though they support us, me as a person, it is good when you detect a patient, you move with that patient and make sure that you give that patient a comprehensive treatment so that that patient might not spread this disease to others knowing too well a TB patient, one single patient which has not taken drug will infect up to ten or fifteen people in a year. Between ten to fifteen people in a year if not treated. |
| Now this decentralization process, did it come with some challenges? | Challenges in the sense that…  Not too much, I have not seen.  Though then we create a date for the OPD clinic for the decentralized because OPD Clinic, DRTB clinic had a date and a day. It is the duty of the supervisor or a treatment supporter to make sure that he gives information to the patient so that the patient might come out on that day to get …, get the drugs and so on so that it will give you the ability for you to manage that person the way you want to manage. |
| Okay, let’s go to the component of that DLTB intervention, I will now name them one by one and we will discuss around them on how the workability if that should be adopted may be by NTBS and even if they are going to adapt, will there be challenges that will come with it.  Number one sir is involving GX alert to be include Local Government supervisors, just discuss on that one. How did you see that, did it work very well | It worked with me now because I do receive it. |
| Were you receiving alert or you were in a whatsapp group and you were receiving information on when the DRTB patients were diagnosed, where you receiving the GX alert from the GS bank machine directly | No I didn’t. |
| Okay, but you people formed a whatsapp group | Yes |
| So, it worked very well | Yes |
| So why is it not functioning since this intervention stopped? Any issues since you said it worked very well but you were not sustaining them or sustaining these other ones specifically.  Okay if we are going to advise the National TB continuous programme that adopts this,what is your view on that and what advise will you give them as they adopt it. | Well, they include the TBLS because anytime a DRTB patient is diagnosed in my facility I will see the result in the WhatsApp group and the focal person in Uyo will call me or send a message to me, .... so so so patient has been diagnosed, follow up and report to your program manager. I will get up, I will get to the Lab, I will get the result, the phone number, the addressed and start to find out where that patient is located from there, I drag down the patient, we start part two. Part two is for you to arrange for baseline after comprehensive health education with the patient because you will not just say come let us go to Uyo, let’s go to let’s go and do another test. It is you the supervisor who sits down with the patient, you give the patient a comprehensive health education. Ma this is what happened. This is what we are going to do before we give you treatment. You take the patient to baseline, after the baseline result, you take it to your pPM Or you give to ...who is here in IDH to prescribe for you based on finding and weight ban. |
| Okay, so you are thinking this should be sustained? | Yes |
| Do you for see any problem or challenges? | No |
| Number two sir, isimproving tracking of patients by supporting the global fund funded CBOs | That should be sustained because there are helping us to get what we want. They should sustain it because by the time this NGO did not come in, we find it difficult to do a lot of things, they are helping us, there are helping us that is why you hear the information of oborokara beating his child to help him to do the job. |
| Okay, Now the 3^rd^ one is improving the counselling of patients using DRTB survivors | Is good because by the time you finish baseline, you want to give DRTB patients, the survivor will be there and you as TBL supervisor will be there. The treatment supporter will be there, all you will do is sit down, you get a buck of that drugs and give, you start to advise the patient, don’t take this oo, example alcoholic, drink enough water, take enough fruits. This is how you are going to take these drugs if you noticed any contra indication tell the supervisor who will now call the PM for further information on what to do because you need to reach back to your PM say o this man is developing so-so abnormality, the PM will tell you or I call ....or the patient will come you take him to ....who will examine the patient and tell you what to do. |
| The next one is decentralizing baseline investigations toqualified referral centres | The decentralization of all these services should not be too far away from where the patient is staying like here in Ikot Ekpene, we have a nearby Local Government like Oborokara in the Ikonoland so on. It should not be too far so that you can bring the patients knowing too well that all these patients report late for treatment. They will be there in the village, they will be manager of, give me this drug, they will enter into a so-so-so and so chemist they will mix drugs, they will come here when the whole thing deteriorated. So, in that case let us decentralize so that if you want to pick a patient, I can remember when we went and picked a patient all the way from Ine Down to Uyo for baseline. I know how I suffered, I had to use my car |
| So, you want the lab to be closer to the people. | Yes |
| Are we going to have challenges with such service | Challenges in the sense that it depends on the lab. That’s why we need to inform those labs that is called Fusola. The supervisors in all the Local Government Areas are in Fusola. You cannot just come any day and say I know this, I know this. I am the person who will take you down there because we know all the lab. Not all the labs are labs. |
| Okay, that’s become a problem or was a challenge to that area | Yes |
| The next one sir is providing transport support to patients for baseline investigations. You think that should be sustained? | It should be sustained. Is a welcome development. |
| So, number 4 is employing mobile connectivity to missions to improve turnaround time of baseline investigation | It should be sustained. |
| Why and then any challenge?  Why do you want it sustained and could there be any challenge? Will there be any challenge in adopting that? That’s what I mean | No. |
| Ok, number 5 is verifying reported wave treatment test using verbal odours | Verify the….? |
| That’s people who die before treatment. Verifying verbal ….that those people actually died | It depend on the family now. it depends on the family. There are some people when they die, it has gone, they will bury it but some families that will say let’s find out what happened. |
| No, in this case, reversal autopsy does not mean you will go and do the normal autopsy that Doctors do. No, is like coming to verify. Is there somebody like Ikenna here, is it true that he died? Is just like asking questions. |  |
| So should that one be done? | Yes, it should be done to find out because I can tell you with the experience in this field, the supervisor on health personnel can just say oh this patient has died because you find it difficult to track down that patient. Because that patient has travelled to Lagos, you cannot find out, you cannot track down this patient. He will say oh doctor that man had died after three years that man come back again. |
| Okay, number…, the next number is decentralizing full treatment initiation to Local Government level. Initiation of treatments | You see by the time we get a DRTB patient after baseline, then we want to initiate the treatment, we sit down in a round table to counsel that patient. You know once a time you have miss this patient proper and improper health education because if you don’t give that patient proper education, see take three tablets in the morning if at all it’s so, 3 tablets in the evening. By the time you don’t give the patient proper information that patient will not do what you ask him/her to do. Automatically you have to give that patient proper health education will tell him the number of months that patient will take that treatment. Let it go into his marrow, let it form because is like in IDH, if we want to start treatment even DS-TB you will sign a consent form, we will collect passport from you, we will sign undertaken with you and you take treatment. Your pastor has to sign for you or your village head so that you will not run away because they are very-very trickish. That’s what we do all these at IDH. |
| Okay, number 7 is the engagement of the state volunteer liaison officer | The state liaison of officer involved? |
| Yes, the engagement of the state liaison officer. Did you not a liaison officer in the state? | I am a liaison officer. |
| Is it okay? | Yes |
| You want it to be sustained | Yes |
| Why? | Because that’s why I told you that we are the foot soldiers. We knew where and how to track down all these patients. If I give you a phone number or a case file of any patient here, say go and track down that patient, it is very-very impossible because you are not here. We have lived here for many years. We know who to use and get the patient so that we can track down and do whatsoever we want to do. So it is in every …. |
| Ok, sir but all these components of this intervention I have mentioned, which one do you think that carries much more weight during the intervention process? | You see I can tell you what happened since I joined this programme some years ago, RedAid invites me for 3days training at Uyo, the money this people give me which is motivation, they asked me go out and do more. Yes, because to whom much is given much is expected. That’s what I think. They have tried; let them come in let us work together in other to achieve our main objective. |
| Okay, Alright. So, sir you not told me the effect of this thing, Is component now, that’s my major question | The component? |
| Yes, which of them? these ones that I have named. Which one do you think is most wonderful, let me use that word. |  |
| Can you remember them or do I still call them again? | Call let me see |
| GX alert to LG TBNS |  |
| mobile connectivity to missions, |  |
| improving tracking of patients by supporting the global fund funded CBOs | That’s the number one. I take that one. |
| Not even the one that was giving GX alert | GX alert is ok and the other one is ok |
| Why are you taking this one as first | Because they are helping me to track down my patient down |
| So that’s what that TBNS is doing | Yes |
| Okay I now understand. Okay-okay |  |
| Did I asked of the challenges you encountered or the facility or you and your team encountered in this process of implementing DRTB | The challenges I have encountered mostly in this Ikot Ekpene and …. A patient will be diagnosed of TB, DRTB, you go to his house, because of one thing or the other the patient will say no I will not take the drugs. If you try to make sure that you go there 2 or 3 times to convince the patient to take the treatment. that’s the big challenge I’m having. |
| Why do they decline sir? | Some of them say no, it is not something where hospital can cure. I’ve been taken herbs, I’ve been treating this and that the thing did not go, how do you say you want to cure and want to cure me for free? Do you think that that drug is a good one?No, you cannot, people have been collecting money from me but they cannot cure me, now you say you want to cure me for free |
| Is either you don’t know what you are doing or | Is a big challenge. It will take you time before you will convince that man to accept like that man we went to his house. I take time to convince him the man accept but now he is healthy. He is very-very healthy. He was happy, we went to his house today |
| What? | Yes |
| So, the major challenge is convincing people to take treatment | Yes |
| Ok, sir how does decentralized DRTB services compare with the old method where everybody was going to Uyo for treatment | This one is very good. |
| Why | Because it is closer to the patient. Apart from being closer to patient, the patient will come in like Ikot Ekpene before the team from Uyo arrive here, we have finished all the counselling with our patients, the drug is ready, we send the patient to the medical doctor to examine, they will collect sample and other things. Very simple and very short, they will go. Unlike … charter a vehicle from Ini or Ikot Ekpene down to Uyo and other people will come. You see a lot of crowd. This one is ok because you will not have much patients and you will have time to talk with your patient, your patient will give you his or her problem and you are able to solve it |
| Sir, what are the advantages of this decentralized DRTB process | It reduces cost like of transportation. |
| Any other one sir | It is accessible for the patient. It is affordable in terms of transportation down here. Patient can have at least, compared to the little money RedAids or whosoever will give to the patient. I think they pay them N3000 or so. If a patient use N3000 down to Uyo and go back with the drugs is nothing but coming from his house after everything at least he will go back with N1,000 to buy something. So it is good. |
| Okay, any other thing sir |  |
| Are there any disadvantages associated with this decentralization process | Because DRTB patient is not the type of patient that will be coming on daily basis to pick drugs, let us fix an OPD clinic where all of them will come in a single day. Like here in Ikot Ekpene on DRTB OPD clinic I will inform my matron. We know how to handle other DR-TB patients. We keep one place for DRTB patients to stay we don’t mix them together and we make sure that we handle them like ….and we ask them to go. |
| Okay. What type of changes did you make to ensure that this decentralized DRTB process is successful? | The change that we made? |
| Yes, what adjustment did you make to ensure that this programme succeeded | My major adjustment to make sure that this programme succeed is for me to at least make sure that I am accessible to my patient, I give them information when I should give them and when they are supposed to be here, I will call them say come to the clinic. Come and take your drugs so that, because information matters a lot. |
| Is that all? | Yes |
| Ok, so we move on |  |
| What are the components of the DRTB treatment, those ones that I mentioned that you don’t want anything to happen to it, it should remain like that continually? | Engagement of CBO should remain  Because they help me to find be patients |
| Okay, any other one? | GX alert should be there. |
| Okay |  |
| Do you think that counselling of patients | Is very ok. Is good because if three of us sit down and counsel a patient is very-very helpful. Not everything that I will be carrying on my head, the one that I forget this one will tell me so that the patient will be loaded with information and go back. It is helpful. |
| Okay. What is the percentage you calculated? | The percentage? |
| Yes, I thought you calculated something | No |
| Then this decentralized DRTB treatment services can you say is complicated? | Is not, because it depends on the schedule of the officer. Because they will only send information to me through whatsapp, you are going to serve on OPD on so and so-so -so day, I had to prepare. I will inform all my patients that they must attend their clinic on that day. It is not complicated it depend on individual |
| How well did this decentralized DRTB service meet the need of your patients? How well? Did it meet the need of your patients very well? | Yes |
| How? | That is why I told you earlier that before the team from Uyo came in here, I will arrange all my patients the way I suppose to arrange and keep. When the team from Uyo come in here, they will call them by name, Mr. ......... will come then we check, come and sign, your phone number, your account number, orderly, orderly. They will start to ask them do you receive your payment from so so and so month, if you don’t receive, let us know. So, they will correct all those things. So, it is a welcome development. |
| Okay |  |
| Now coming to this decentralization, do you think we should decentralize further or we should reduce the decentralization process? | Like here in Ikot Ekpene, you should reduce it because the person coming from ....Local government and Ikono should stop at ikono. |
| That’s increase then | Yes |
| That means we should decentralize further | Yes |
| Any reason for that? | Because of the distance |
| Ok, the distance is the major issue | Yes, because if I sign N3,000 here then the man from Ikono or Ini will sign N3,000, it is not the same thing. It is not the same. |
| What support are available to help this decentralization process to succeed? | To engage more people now. |
| No, what support, support may be from NGO, partners and all that? | From RedAids? |
| Any of them, is there anything they are supporting to ensure that DRTB goes on well? | You need to increase their payment. |
| You need to increase their payment? | Yes |
| So, there is already a payment? But just that it needs to be increased. |  |
| Any other thing sir | Arrange for seminars |
| Okay that’s incentive now | Yes |
| Seminars or training | Training |
| But are there incentives that supporting the success of this decentralized DRTB services | I have not seen |
| Okay, but the support is the one they give to the patients. | Yes |
| Are support also been given to health workers? Any support been given to health workers? | The support that they give to health workers is the one that I may say if only you are a treatment supporter |
| Yes? | Yes, if only you are a treatment supporter. |
| Okay |  |
| Then your facility structure and setting, how did it improve or reduce implementation of DLTB. | They have no problem because the moment we have a day for DR-TB clinic, I will inform my matron and .... is aware of that. We know how to strategize patients that will come for that day so that they will not mix up. We create a room for DRTB patients so that we can see them and you are not to keep them for quite a long time. The moment they come, you counsel them and bring all the findings and send to doctor, they will come out and take their drugs and go |
| Okay, thank youSir what have been your motivations in supporting the success of this decentralization DRTB services | Apart from money, Passion, one must have passion on the job. I’m always happy if I do the work. Let’s forget about money, money will come when it will come. I collect a lot of gift from patients, not that I ask them to give me but the way I handle my patients. I will sit down with you, I will crack jokes with you, I will tell you what to do. Some of them out of joy they will come and say I come to thank you. Money will come when it will come. Passion. |
| So, the passion for the job is the main | Is the goal. |
| Okay any other thing sir | Nothing |
| Sir how confident are you that this decentralized DRTB service will be sustained | I am very confident, I know it will be sustained |
| What gives you that confidence? | The confidence is on the fact that it depends on the people on the top. By the time they will sign a budget and say come and work, you will not run away, you will come so I pray that they sign the budget so that we continue the job |
| So,you’re believing that as long as there is budgeting approvals, it will be sustained. | Yes |
| So, how confident are you that your colleagues will work towards sustaining the process? | My colleague? |
| Yes | That is why I said is dependent on individual. Me I know myself that I can work. |
| Are you confident on this? | I am very confident |
| Ok what do you think that your colleagues at work, what are they thinking about this decentralize DRTB services, do they see it as something that worth sustainable? | I look at 60% |
| Okay |  |
| Sir how well did this decentralize DRTB services fit into your existing work processes and practises? | You see some people need to have a work plan. By the time you don’t have a work plan you cannot accomplish what you want. You must have a schedule say this is what I’m going to do in a month even when you wake up from bed in the morning, you say I’m going to do five items, you have 5 plans, at least by the special grace of God you can accomplish 3 or 4. You must set a plan, set a plan say this is what I’m going to do this month, this day, this week. Not everybody have the same mentality |
| So did this DRTB services decentralize, did it fit into your work process? | Yes |
| How did it fit in? | Is fitting in the sense that I know like if I wake up I will say, I have five patients, these five patients not all of them collect drugs at same day o, same time o, because you did not diagnose the patients at the same time, same day. You must have a work plan. This is what I’m going to do, by the time they collect their drugs whereas you issue drugs to them two weeks because they are not going to live with you and depend on the treatment supporter that you are using to handle their treatment. Your phone is there as a working tool phone. You call Mr.,.......I am coming to you o, you supervise them now. you make sure that you see the drugs. Sit down there if they are not taking that drug in the morning let that patient take that drugs before you, you see o this patient has taken his drugs. You advise them. What problem, most at times all of them are very poor so as you are going there you must go with a little money in your pocket. You are going to see a sick person and say sorry o,.. at the end of the day, N500/N1000 take and buy groundnut. It brings you closer to the patient so that the moment you call that patient next time say o sir is coming o, sir is coming o. whatsoever you want him or her to do. He will wake up and do. These supervisors need to be given money so that you can do this job |
| So that’s the incentive now? | That’s the incentive |
| Ok so it fitted well. |  |
| So sir we have two processes now, that’s decentralized DRTB treatment services and then there is another one which everybody go to Uyo take treatment, now which one do you support? Do you support that it should replace that old one? | No. let us forget about the old one lets choose this new one. |
| Why? | Because it cut cost on transportation. Going to Uyo is a very difficult task, very difficult task. Take a patient here down to Uyo. What if the patient is coming from Isinobolo down to Uyo, what if the patient is coming from either Ikoli obasi down to Uyo, Nkari in Ine Local government down to Uyo, you know how much it will cost? Now it will cost you up to N10,000 but coming down here you will spent a little money at least the money you will give them for transportsay ok I don give money to pay your way down there to go and collect your drug than going to the old one where some during that period not all the patients comes for treatment- OPD clinic. Now at least I can assure you that we have 70 80% that come for OPD clinic if not all. I can assure you that because if it happened that you on OPD clinic at least you see nothing less than 70 80 percent. Very few will be minor complains when the supervisor will be calling the drugs. Take to his house and give. |
| My own questions have finished but if there is any other thing you want to tell us, inform us on the decentralization of DRTB treatment services sir let us know | I’m really happy for your visit today because anytime you have supervision, any supervision that happened in any facility, it is not a fault-finding mission. It is where that supervisor will go and correct the person at that facility, this is what to do and if you have not been doing it, do it well. So, thank you very much for coming down here. In fact, you have boast my moral, yes because next time if you say you are coming to me I will sit down, I will prepare myself. Had it been I prepared myself for you, well we thank God. I’m very happy |
| Thank you. So, this is the TBNS for Ikot Ekpene LGA Akwa Ibom State. Thank you very much sir | Thank you sir. |

**Title of Interview/discussion: KII/B12**

**Date of interview: Monday 7th August, 2023**

| **Interviewer/facilitator/questions** | **Respondent/responses** |
| --- | --- |
| **Good afternoon sir** | **Good afternoon** |
| Okay, so I will start with the questions. Thank you for agreeing to the interview and thank you also for making it possible for us to record this interview.  So, what do you think of TB Reach Wave 9 intervention to decentralized DRTB treatment services? | Thank you for that. I think the DRTB as the name implies is actually a welcome development in assisting the patients with less capital to help them get treated for TB.  Over time in Akwa Ibom state, we realized that we have an increased number of those who are diagnosed with TB, and for the benefit of either transportation, I think the decentralization will also benefit the patients more. |
| Okay, so what do you like most about the decentralization process, what makes it fascinating? | Well, probably it's the same process that we've seen at times, I think it's a way of bringing the treatment closer to the patients. So that's one of the benefits I can say otherwise, it's still the same process that has been before |
| So apart from bringing the treatment closer to the people any other thing?  Any other good in the project or the program? | Well, another thing that I discovered recently is the drugs that are being taken to the patients that's another thing about it, unlike before TBLs will collect the drugs and give them to the patients.  So, currently, I realized that the pharmacist in charge will also collect the drugs and go to the center when the patients come and also collect their drugs and that also helps them. Like I said it's simply a big help to the patients. It also helps us to monitor that every patient that comes will also get their drugs |
| So, the basic thing about it is that it brought the treatment closer to the people. | Yes |
| But is there any no side of it?  Does it have any upside aspects that don't gel well with the goods | No |
| So, all aspects of the decentralization are good? | Yes |
| What has affected or influenced the practice of supporting decentralized DRTB services in the state? | What actually affects you? |
| Yes, what actually affects you in supporting? | I don't understand the question |
| Okay, I said what affected or influenced your practice of decentralized DRTB services in the state? | Okay, one of the things that actually influenced my work as the DRTB laboratory focal person is seeing the patients with passion and the less privileged, and sometimes they don't even have the money to pay for their transport to the facility. But sometimes we actually had to fuel our tank to go to the particular OPD site for us to be able to care for the patients take their blood samples and get the blood samples analyzed.  So that actually affected us in a way,  making us have the passion to work with the patients because sometimes you see most of these TB cases are persons from less privilege and most of them don't even have income.  Some equally complain about their transport and all that and that actually affected us in a way and sometimes seeing most of us as staff when you see most of the patients, we need to assist them in one way or the other. |
| Okay, so what really helped you the most in supporting this decentralization process? | The experience |
| Okay the experience in the work? | Yes, that actually really helped me I've gotten a whole lot of experience ever since I joined this TB work as a whole.  That helps me to have more zeal to support the patients.  Sometimes we discover that there are some cases that are not necessarily TB cases, you understand, they are not necessarily TB cases, there are some apart from the line of investigation that they included for us to go for the patients.  But there are some critical cases that you see, maybe as a result of TB or some other ailment that may be related to TB and all that.  So we have to extend our tentacle in helping them do some of those tests, sometimes free because sometimes when you see the patients they don't even have money to pay.  Some complain they do not have food to eat to even take these drugs and all that.  So that alone will also help you to lay your little support on them by assisting them to do some of these things for free.  Sometimes, in fact, I remember one time in I think that was in Oron I decided to support a patient with at least 2 pints of blood because of the level of the case, the PCV was very low and myself and the doctor in charge was very passionate about the whole thing from taking the drugs, the feeding and the status of the patient's case. So, it was very critical and when you look at the stipend that was given to them it's not even enough. There are staff currently considering the situation of things.  Over time, with the experience that I have gathered actually as a concerned citizen of this state, I also discovered these are our people, they are also from this state, and that's why we are doing our best to help them, so these are our changes. |
| Did you face any challenges? | Not really.  Another challenge that I know is a very big critical case.  Some of these centers that I know don't have X-rays for them to do maybe chest X-rays and all those things.  So those are some of the challenges we feel are critical. Sometimes the centers don't have chest X-rays like Oron the general hospital Their X-ray is not functional. Even in some private sectors, there's no functional place where you can do an X-ray. So, in those cases now are some of the few challenges we face. |
| Okay does decentralized DRTB services compare with existing routine programs in the state,  like before the decentralization and now during that period of intervention how do you compare the two? | To me I don't see any difference.  We've done our parts,  when patients come we attend to the patients, we have the best hands in this facility and all those things.  Take for example, if a patient that has been referred to a teaching hospital even though the patient was far away or maybe his or her site is Eket or Oron and all those things when it gets to maybe when the doctor will say this patient needs to be moved to a teaching hospital, the truth is whether you like it or not you have to pay your way out to that place.  So, it's work we've dedicated to do, it is work we feel has been paid or we've been paid to do.  So sometimes it's not everything, this TB work has to do with passion that's the way. If you don't have you won't be able to go for it, it's something you have got to have passion for. |
| Okay, but we are now comparing the two, the decentralized and the non-decentralized, what's the difference and not necessarily about the patient but about the program? | Okay, apart from the fact that in the decentralized one, we were able to engage more doctors to see the patients and to also cut down some transportation from the patient's side.  So myself I don't see any difference but for the program,  yes. |
| So, what are the advantages of this decentralization process? | Advantages are still the same thing; less transport money on the patient's side,  that's the only thing. |
| So, that's the major advantage? | Yes, and the treatment is also closer to the patients |
| So, any other advantage? | Well, those are the few advantages that I know |
| Any disadvantage observed? | Disadvantage, the only disadvantage that I can say is the situation whereby the doctors that are being engaged to see the patients, maybe when you go there you see the doctor is engaged with other routine work.  But the percentage is very small, it happens maybe like once.  But that does not mean that the core person who's actually with us will actually see the patients.  That does not mean that the work will not be going on. The focal person will also see the patients because sometimes even the state program officer used to follow us to the field. Otherwise, different sites we train different doctors to see the patients.  But as we all know the program crashed with or has one or two important services or engagements, they want to do that they can not tender excuses for, so apart from that it does not really have any other disadvantages. |
| Okay, so what kind of changes did you need to make to support the decentralization of the DRTB services? | Is it me personally? |
| Yes you as a person did you make any adjustments? | Not really |
| Everything went on as agreed on? | Yes |
| Okay. So, there's no extra effort that you put in to ensure that the decentralization process went well? | Well, what effort will you put in when all you know is the way Akwa Ibom state is doing their own,  arranging their own OPD clinic is actually well arranged,  you get notice of the OPD clinic like one week to the time.  That could give you more time maybe to exchange your schedule, plan your events and all that, and know that this particular day the patients will come otherwise. Another effort that we also make to support is that we've been able to place the DRTB patients as one of our high-priority patients. When they come here to this facility assuming it's like a baseline investigation we would have to attend to them first because they will take them upstairs, they come here we have enough bed to take care of them. So, we give them facemasks, they wear their facemasks, we take their samples do their X-rays, and the necessary other investigation and we release them to go. So, we don't keep them. So, those are how much we value them. |
| Okay, so that's good. Now are therecomponents of these interventions that you want not to be changed? Can you remember the components of the intervention, one is decentralized lab services, there is a GX alert system, then they used TB survivals to do counseling, then the other one they give verbal autopsy, they subsidized or supported movement, and baseline investigation, you know, are there any of these components of this decentralization that you want them to keep on.  Also, there is a liaison officer in the state. These are different components of the DRTBdecentralization process. Are there any of these that you would want them to keep and not change? | I think all the components have their vital role like you mentioned, all the components are very important though I want all the components to continue. |
| If you were supposed to Mark one as the best which of them would you choose as the best? | Can you mention them again? |
| Okay, 1. Involving GX alert, no. 2.  Improving tracking of patients that are supporting the mobile-funded CPOs, 3. Improving counseling of patients using DRTB survivors,  4. Decentralizing baseline investigations to more qualified peripheral labs,  5. Providing transport support for patients in baseline investigation,  6. Employing mobile connectivity solutions to improve turnaround time, 7. Verbal autopsies, 8. Decentralizing treatment initiation to the LGA,  and no. 9. Engagement of a state liaison officer.  So which one do you like as the best intervention? | One of the interventions that I've seen as a welcome development in the wave 9 project is when they use a DRTB survivor to also assist the newly diagnosed cases in basic training.  It also helps in encouraging the patients to adhere to their medications.. I've realized that Over time, before now you see the State Program Officers threaten patients with police so as to take their drugs. So whoever brought the idea of using treated DR-TB survivors to join the counseling I say a big thanks to that person It is a good way to give hope to the patients to encourage them to take their drugs knowing that they are not the first to have DR-TB and those that have had it are alive and well. |
| Okay, so how complicated are the decentralized DRTB services, is it complicated, or is it straightforward? | Yes, it's straightforward |
| So, what makes it straightforward? | It's straightforward because in our society now, sometimes you need to bring innovations to every program that you're doing. So, it is straightforward in the sense that we have different centers, and the centers after being notified, or maybe we use a particular place or the TB, and the patients will just come to that particular place and we will do a health talk to them even before we start the clinic on that particular day.  So, it's not a serious thing, it's actually straightforward and it's a welcome development. |
| So, how well does decentralized DRTB services meet the need of your patients? | Well like I said, It actually meets their need because we go to them to take their sample instead of saying they should come to us for us to take their samples.  So, we go to them to take their samples and analyze them. The result will also benefit the patients. |
| So, are you saying that you meet their expectations? | Yes, we did |
| So, can you define the specific ways you met their expectations? | The expectation is that some of the investigations are been ordered, you understand to help in their treatment. Once you diagnose, once you get the test done and get the result and the results evaluate the state of the patients, why won't you meet the expectations of the patients? So we definitely meet their expectations. apart from the fact that at least I know that some of that investigation might not really be a kind of directly related to the TB diagnosis.  But sometimes you will see some critical cases that you have to state if you may need for the program to add one of two investigations to the already applied investigation of the day |
| Which investigation do you have in mind? | Okay, before now we used to have a thyroid function test, but at a point, we now removed the thyroid function test. You know it's also a way of maybe PSA for those above 60 years, maybe PSA for those above 60 years. Because sometimes you know it's a supporting program and as a result, we could also do that as a baseline investigation whilst even in females they have free pregnancy tests for them, you just have to do it and not even be as the result of the drugs they are taking.  You see we are all human, what is good for A should also be done for B.  You see that investigation will also help you so that we can channel to them. Some of them don't even know their prostate level,  their thyroid function, and those things.  These are the things before now KNCV was actually the one taking charge of this program. I know some of those investigations were actually done before now but I know over time maybe as a result of a review of the drugs they started withdrawing some of the tests. |
| In this state do you think there is a strong need to even increase the decentralization or even reduce it? | Okay, now I think we have senatorial districts in the state,  so whenever you work or whatever you feel you have to do it is based on the senatorial district there. So the way they decentralized it I think it's very ok. Akwa Ibom state is not like Cross River state that the land mass is very wide, do you get, that there are some states in the northern part of the country that have a large land mass and all those things?  So you have to also try to put down this thing otherwise you abuse this thing because you know you will also bear in mind that if decentralizing needs more you will also involve more expenses. So, for now the work has been going on smoothly we haven't had any challenges concerning doing the work over the years.  So, if there is money, I will plead they will channel it to patients’ esteemed stipends.  What they are giving them considering the economy of today is poor and not enough. Some of them have been complaining. You know, that stipends alone is what encourages most of them to come out. So I plead that they should look well in that direction. But telling this Ikot-Ekpene, Uyo, Eket,  Oron,  four of them are okay. |
| So, what supports are available to help you to help you to adopt these decentralized DRTB services?  Any support from Agencies or organizations? | I don't know if there's any,  but the only support that they came up with was when we did the investigations that are being requested by giving the OPD and equally the baseline investigation that we're being paid, the facility is being paid. Although we have also pleaded for a review of the money that is also paid to us. You know you can't do like chest x-ray for #3,500  and for the last how many years it is still #3,500. Up till now the film that we used to buy #80,000, it is about #120,000. But because you signed a contract you cannot go and increase any amount until you can actually request and plead to the company to look into the support that has been given to you. |
| So, the support you mean, is it the improvement of the price of the chest X-ray? | Is not just the price of the chest X-ray but almost all the investigations that have been done. Like the kidney function test return #3,400, full blood count, you can ask any of the medical laboratory scientists who are running a five-part differential auto analysis in this particular place. When you finish, I can take you to our lab and you will see the machines we are using. We are using a first-hand machine in this particular facility but we are trying to meet the standard of the patient's needs. So, you put money to get money in all those things and all that too you will also put money to the required things, maintenance,and all those things,  #3,500 in liver function test even full blood count they are putting full blood count #1,700 is like abusing the price. But then, since it's a monthly investigation a monthly thing, since it's like a turnover of things that are coming it's better than nothing |
| Any incentives apart from the support coming from the organization? Any incentives to support? | There is no incentive available |
| What do you think that this state has maybe by setting or by culture that has helped or not, nor that has affected either to improve or reduce the implementation of DRTB treatment services?  Is there any setting in the state culture or any other thing that might have helped the implementation process or hindered it? | Culture |
| Culture; the setting of the structure in the state, the working formula in the state, any other thing? | Nothing apart from the fact that even we are still pleading with the state government to assist in this particular program but apart from that, the Focal Person, the State Program Officers, and the TB team in Akwa Ibom state are really up and doing.  They are very committed by way of sensitization, there's no event they don't go to do sensitization even in church, this sensitization also helps in DRTB processes |
| But are there any other processes that hindered decentralization processes since the crack team helped in improving the decentralization process?  Any other factor that is working against the decentralization process? | I don't think |
| Okay, what has been your motivation to ensure the implementation of the decentralized DRTB services? | Consistency of the program,  ever since I joined this TB program,  they have been very passionate and consistent. That alone actually motivates me. |
| Okay, any other thing? | Nothing |
| Okay, so how confident are you about being able to implement decentralized DRTB services consistently? | Very confident and ready for the work if given another opportunity to sign an MOU for the program |
| Okay, so what gives you that confidence, any special specific thing? | Well, you know when you have good machines, you very good machines, it's a fully diagnostic center. We even have a mammography machine, we have ultrasound machine here, our lab basically is an automation laboratory, that a lot of confidence that whatever task I'm given I will be able to handle it |
| What about your colleagues do you think they build that confidence that decentralization will work? | Well, whatever you're trained you can actually do it well. I believe with time we will embark on adequate training to create awareness about this decentralization.  We have already started decentralization. I don't know if they want to do a more upgraded decentralization again from what we've been practicing because I know their lab in Ikot-Ekpene,  Eket Side,  Uyo.  So, we've been working perfectly well so I don't think there's a problem. So, I believe that my colleagues in other centers are also ready |
| How do decentralized DRTB services fit with your existing work and services? | Like I said the medical lab scientists, my work is to do the investigations and this is part of my work.  I have a passion for medical lab science as a practice which was why I went into this coalition. It is not a coincidence it's something that I have a passion for.  I've been doing a whole lot of investigation and all that so I derive joy in doing things like this because it brings experience and it also gives me ideas.  Also, people can use this as research work and it will let you know what's going on, what value really raised during your baseline, what are the possible things when you see the trend, and all those things you will be very passionate about and prepared about the work.  So far there have been no challenges in doing the work perfectly |
| Okay in your own opinion will the decentralized DRTB complement the existing routine practice? | Sorry at this point if I may understand let me ask, we've been practicing decentralized centers to an extent in the state,  so I don't know because most of these questions are actually interrelated and I don't know at this point whether we want to do per Local government because when you talk of decentralizing like that,  whether you want to do it per local government but apart from that I will prefer like I said Akwa Ibom State,  state differs you understand,  most of them in land mass and all those things.  So, apart from that, I don't know whether we want to mostly split it among Local governments.  Otherwise to me, for us not to have value for the program.  Because sometimes if you go to a program and see like 20 or 15 persons in an OPD it will make more sense than going there to meet only 2 or 3 persons.  It will feel like maybe these people are not really serious about it and so many people have this TB but it is only me and you that will be there. Sometimes some persons will not come and if they come and see they are not the only person that is suffering from this particular ailment, from there they will come and see another person that will be very bad in terms of, you know even this DRTB has stages of the infection. So, I will actually advise in my candid opinion because this is Akwa Ibom state, we can leave it at that. The state that has more liaison officers like Cross River State, from Calabar to maybe Obubra and all those things, are very far away, that alone can be understood.  But in Akwa Ibom State the land mass is not all that far, the Local government are quite close to each other.  For example, if you have 1 or 2 children, sometimes they feel loneliness and all those things.  But if you have a family that is up to 8 or 10 they will sit down and maybe encourage discourse with one another.  So that's my very valid points |
| So, are you in support of the current level of decentralization in the state? | Very in support |
| Is there any other thing you want to comment on the decentralized DRTB services? | Well, I want to thank the program, they have been of immense help to the patients and I pray that the program continues.  Sometimes on my own, we see some cases where I will now say what if the DRTB treatment was not discovered, you know implemented.  Before now we just knew HIV it was really about HIV cases and all those things but these DRTB cases are really of help to people who are being diagnosed.  We pray that the program will be continued and that more support to the patients financially because whatever we are doing, we should not take away the support of the patients that the program would have been given. That alone also really encourages the patients to come and it will encourage the program. |
| So, you're in support that the financial support of the patients should be increased? | Very well in support. Also, the GeneXpert site should be visited more often.  By the grace of God to my knowledge, this is the only private lab in Akwa Ibom state with a GeneXpert.  I've been in the GeneExpert Center and over time we've been doing so much in diagnosing cases.  If it wasn't given to us, the cases that we diagnosed we wouldn't have diagnosed them.  Probably they might have given to other centers or maybe might have died undiagnosed.  So, that line is a very big case. Thank the federal government for supporting us with that GeneXpert and we also plead with them to come into the solar that was given to us because some of them have some issues. I know they have been coming around to see how they can help, but generally, if there is a way this program can assist the facility in the maintenance of that particular geneXpert. It's also a way to support to some extent because if the machine is down, we will not be diagnosing, we will not render services and by so doing we may not know if the machine is down for like 2 to 3 months or maybe 2 months or maybe in between could have gotten maybe 3 to 5 diagnosis. So, it is something the program needs to check because currently, the AC we are using is this kind of AC that is not solar dependent. So, we are using about 1.5 horse drainage power solar and also a fridge for storage of the sample. When the program was given to us, we were told that it was supposed to come with solar AC and a Refrigerator that is also solar-dependent or a better refrigerator but up till now, we have not seen and because we need to work, so we've overused the solar as a whole. |
| So, is there any other thing you want to chip in? | Well, like I said I will also say that the prices of the investigations that are being done should also be looked into.  They are doing so much for the program but the money is not encouraging.  So they should consider the prices of those things considering the current inflation in the country so the price should be looked into.  Overall we will be doing the work very fine, the State Program Manager and the State Focal Person have been doing wonderfully well.  They have been doing well since they came into the program and they are very committed to the program |
| Thank you very much.  So that's the interview with the state man focal person  Thank you sir for the time. | You are welcome. |
|  |  |
|  |  |
|  |  |
|  |  |
|  |  |
|  |  |
|  |  |
|  |  |
|  |  |
|  |  |

**Title of Interview/discussion: KII/B13**

**Date of interview: 7/8/2023**

| **Interviewer/facilitator/questions** | **Respondent/responses** |
| --- | --- |
| What do you think of TB reach WAVE 9 intervention, which is decentralized DRTB service? | Well, for me, TB wave 9 which was actually meant for the case and actually to close the gap, enrolment gap that was found in the PMDTs units. but when it comes to decentralization will I say, we were technically decentralized before now as a state. Yes, because we have our case in the four major centres, Uyo, Ikot Ekpeneg, Eket, and Oron. So, for that reason we didn’t even see much need because that was, you know those four areas were actually carved out for where most of our patients were. So, it was very convenient for us. We technically did not decentralize it throughout the wave nine project. Well, we try to centralise the cases of the laboratory services. We had one or two issues with vendors out of town and it was, we had a lot of back and forth, some refused to give the services, some felt the prices were not ok, so for the reason we technically did not decentralize to the level we were supposed to decentralize. But not really, though I know there was an impact by the team |
| Okay. So, what do you think was the important aspect you appreciated and the ones we did not in the concept of decentralization | Okay. In the concept of decentralization, well I think I like is the fact that patients could boast to get their business done closest to them. But the fact is that what are we expecting when it comes to human behavior? Some people, because of stigmatization for whatever reason, still prefer to come and do their test even in the capital, some feel the test will be inferior if they do it in the peripheral health facility instead of coming to the center. So, for whatever reason we saw patients, who we told to find the facility closest to them, prefer to come to Uyo for that testing. And now the transportation cost is more and the support given by one of our partners for that is grossly inadequate. Even before the fuel price increase, it was inadequate. So, we’ve been having back and forth with them, but I will say on the whole that one or two lessons were learned throughout the wave nine project. |
| Okay. So, in other words you have already said the ones you appreciated and the ones you did not. | Okay, the one I did not appreciate |
| Okay | Yes, I have, I have said that |
| Okay yes - okay yes.What has affected or influenced your practice of decentralized DRTB services in your state? | What has affected our practice, well will I say anything affected it. I think apart from the vendor in laboratory service, I don’t think much has really changed, Akwa Ibom was already a decentralized state. Before they were recognised. |
| Where and where, were the decentralization before wave 9 | Uyo, Ikot Ekpeneg, Eket and Orom. |
| Is it in terms of ………….? | Geo-political zone |
| Is it in terms of initiation of treatment, community care or in terms of running of tests or those other new components, like the counseling of patients that TB wave 9 brought were and where will the state fit in. | Okay. Yes, if you ask me the highest point for wave 9 was the fact of using survivors in the counseling process, to me, it was a time breaker. Because once you see someone that tells you I have been through this thing you see that there is nothing there, I think it actually meant a lot for some of our patients. And I think we did not really have the result of poor drug adherence since the wave nine compared to the previous one, because by the time you see someone telling you I took this drug for so-so number of months, I feel better now, and I have gone back to work and I’ve gone back to this. You actually see the person and see that the person looks ok. I think it was a game changer. But the other challenging issue we had I think we still have issues around them the part of documentation where they want to test a patient is still an issue, because here and there we have some issues of people not still writing proper contact address of this patient. I know RedAid has done their bests to get us more specialized result whereby we can get more than one contact of the person involved. I think we still need to make a deeper dive to solve that particular area when it comes to that. |
| Okay. Apart from the patients counselling is there another component that appeared as another magic one. | well, there is a magic one I thought of, (don’t distract me), there is another magic one(you see I don’t like what you did I have forgotten what I want to say) |
| So, we are talking about another magic one. | Yes, there is another magic one that works in everywhere and every sector which is money. And it didn’t happen in this project because we are seeing people, you in this project we look and the PMTBCs before now, there used to be a lot of money attached to it for the patients and for their upkeep, transportations. Those things are not really there in the program now and there was no upgrade in the figures attached to the patients for their treatments, and for that reason some people will say I don’t have money for my check-up, I don’t have money for things like that and they just shut down there I think that magic one, that absent magic one is actually because by the time you tell someone u will give him money a reasonable amount of money for to and fro, I think a lot of them will actually be able to meet up so I think that was the magic one that was concealed and I am hoping moving forward it will be reviewed |
| Okay, thank you we are just under braker and we are coming in main action so and then your States is a bit unique because you said you are already in the decentralized to an extent so how does decentralized DRTB services compare with the routine existing a program in your state now | Well, I will still say that we are technically not decentralized during wave 9, TB reach wave 9, what we use to do the particular their adaptation to the wave 9 we have was about the vendor the uyo vendor for follow up testing goes to all the OPD vendors he goes, he is mobile so he moves with the mobile ECG machine, does ECG there, does the other test there so we basically use our vendor for baseline testing but for follow up it think it was easy because a system was already adapted within the state before the road where by the time we got to the OPD person, the lab scientist comes with his vehicle and all the sample containers what is it called the vaccine that is the sample carrier, take all the samples they do the ECG there on the site and then move them then patient now go back to their different, get check up and move back. And I think it's really been helping us because by the time we think of opening a new patients even to go for example we have a lab and the lab is not situated in the OPD site so even coming to the site the patient will still need to go to the laboratory for their investigation but now we now brought in a way that in a particular site where the patient is coming to take treatment is the exact place the patient will get his resignation done .and that's is the same site that will be used as follow up tasting. I think we did everything for every OPD site to be like a one-stop shop. It has been our initiative in the state before the wave 9 program. |
| Okay. I can see your state is already a bit unique in this very aspect. However, let's go to the normal routine for other states. How do you compare that with decentralized DRTBs services | Well I think because geographically Akwa ibom state is quite blessed you know we have , the state capital where we are is central in the state so every Local government is just at the maximum will be the periphery of the circle not at the center so for that reason it's very easy for us to manipulate the timing and accessing these patients compared to other states like cross rivers state I know is very unique even though I'm not supposed to call the name of the state but I need to, it's a corridor. I used to call it Gaza street but it's a corridor where but you could drive like about six to seven hours I know that's an issue but in Akwa Ibom state one way or the other within an 1hr-30 minutes.You can get any local government headquarters. And we have a good road network in the state and so you can get to any of those places, so that really worked for us compared to some other states. The decentralized, the geographical location of the state capital to all the peripheral health facilities and also assistant, and we have good labs at this site it's just that we are having more of technical issues, MOU issues with the laboratories we use more of the ones at Ikot Ekpene one of our sites. He helped out to do baseline test and all of that but in Eket, and Oron we don't really have labs that are willing to do the test there was one we had and we had a lot of back and forth, machines are bad this that and that and that. We had a lot of back and forth and even Oron said that they refused the price of test in the MOU and these tests are too poor that they will not break even with the test. So that's how we get there. |
| Okay, so let's start with the advantages. What advantages does decentralized DRTBs services have | Well, the advantages is that, we will have patients not going too far for services also we have the fact that patients can easily receive care as soon as possible compared to having to come to the center. You know at every peripheral site we have creation papers there. We have the supervisors that are there so for one reason or the other they are close, it makes them closer to the patient than coming centrally so that's one. Another advantages of decentralization is the fact that it also structuring, you know, you can imagine when we have only one creation you see all the patients, you see it will be, it will not be wonderful you can imagine having up to eight, go to each , our open days are two days, go to each of the sites like twice every other day so for everybody that is , so for everyday hopefully Monday, Tuesdays, Monday go to Eket and Orom Tuesday you go to Ikot Ekpene and Uyo. So,for that reason we have, that if it was to do work initially the work would tire the person and the person would get burnt out. They have different technician at different sites and you know one way or the other the technicians are now the ones carrying the one bonding to these patients You know if one person goes and see all the patients you may not truly appreciate but if you know you are seeing like five patient in your OPD the technical man will first be there and I think the care gets better. |
| So it's that enough for advantages. | Well, it's not like, when we move around in the. |
| Okay, okay, so what are the disadvantages sir. | Disadvantages, the state team has to access those sites too, and the support for it is nothing to write home about because if state team members are supposed to be there, there should be some form of calculation as regard the transportation and maybe probably lodge but those from whatever we get from supportive partners is a far cry for what I think is ideal. |
| So, what kind of change do you need to make for the decentralized DRTB services to work effectively | Changes, changes change, well financial changes, sorry it's not like I like money too much, but we need to make financial changes, let the patients breath, let them have a little more resources with them to at least access that OPD site because even if they put OPDs site 50 kilometers from somebody's house the person don't have transportation he won't come so I think that's the number one. So, if we are decentralizing, decentralizing if we don't really get, the patient cannot be supported enough to make it there we still need to continue with. There is another counseling style, where we are using survivors to counsel patients. I think that works well. The essential support for these patients, well I think it should be looked at and also recruitment of the supporters, I think we will actually be doing something when we are getting more credible people to do it to the treatment supporters compared to formally. You know when you have a little boy who is a treatment support to his mother, you know it won't work out, because you can't tell the boy to go and take your drugs, that could mean starvation for the child. So those are one or two of the issues I think we would work around. |
| So are you advocating that for the entire DRTB program or just for the DRTB treatment scheme. | Well, I will say for the all programs. I have said about the staff, the state team, I have also talked about the patient and even the technicians at the field. |
| I mean that one of the treatment supporters of a child. | The Treatment supporters of a child yes, that one is something I just made mention of. It's something I will take into consideration of. All those adjustments are being done. Following the rebranding of our PMTBC package. |
| Are there components of the intervention that is, this DRTB intervention that should not be altered. | Well, I think the medication should be altered, it should get less poor care regimens of our patients, I didn't mention that, that's why I said I shall look alike. You know the pill burden is quite high for DRTB, some take as much as 24 pills everyday who does that and you expect adherence. Now we have the glue stream reset that is at work, and then I think we are supposed to be narrowing down to the pill part regimen now, I don't know what is happening. I think we should have gone to that. We went to that, I think decentralization will be better than we are even arguing now. Because we have to direct all local governments, we could just let it go back to the way that we were doing our DRTB treatment whereby it's the TBLS that will see this TB case and place the person on the bill part regimen. And with the bill part regimen the investigations are going to be reduced. Going to be reduced we will not have all those and the side effect also will be reduced.  A whole lot of things will be dropped down even the base and those other specifications. I think with that we will do a little more, if we can do that, I think we cannot make the health care workers even at the peripheral stage. All the armor tanks that are rolling out for DRTB will be better resources, better outcome |
| But let me still touch on those components again. You remembered you mentioned that, about patient counseling. The other component is the decentralization of labs so we are just coming back to them, which of them do you want to remain as they are. With regards to these interventions . | Can't I choose both |
| No, You can choose as many as possible the only component. You have already talked about the patients' counseling services which you said it's the game changer or I've forgotten exactly, it's just that it's not All of them that work at the same pace and at the same smooth way. So, Let me know the component that you want to remain intact. | Okay, I will still want the OPD sites to remain intact, I want the inclusion of the laboratory services. I think we could do some little adjustment, like I said for some of us the lab is actually the price, the MOU that's the issue. If an upgrade is being done, I think we will be able to have that done properly because even though we are feeling good that we are running a close to single partner run package, we still have issues, because a one’ man lion squad will not really work. What if our vendor cannot make it for any reason at all that means we will have a total collapse of the system. So, we might really want an upgrade in the rate for the tests and for that reason we will not be able to, (what word do I use) degrade them into our laboratory schedule. Yes, that's another one. Which other components |
| Do you still stand on the question counseling? | Yes |
| It should remain? | Yes, it should still remain. In fact, I think she has already done much for us |
| So how complicated is the decentralized DRTB service? Or do u think it's not | Complicated, it's not complicated, the only issue we have is the clinical part of in- all our clinicians have not been formally trained on programmatic management of DR-TB. All the clinicians for Delta have not been trained. I have had a mini training and that's when I was a DRTB focal person, currently one of our clinicians has not been trained for a very long time. And the other two clinicians have no any form of clinical training and they virtually get technical support from me which we know is not the best, because when I'm not on ground I am not on ground and we won't know what is happening to the patients so they just use their initiative to give their thoughtful idea as clinicians. So, if it is possible, we want more than one clinician trained. So that we can have trained clinicians so that, for any reason one is on leave, one is not available, the other person stands in. |
| Okay,that means It's not complicated, it's straightforward then. | Yes, it's not complicated.  It's straightforward. The doctor will call them and they come and they treat them. |
| How well does decentralized DRTB services meet the needs of your patients | I think that it is the patients that will answer that |
| From your own position, they will also answer from their own part | Okay. Cause I know, from the patient that agrees to tell because if I say they may say this person is just talking rubbish. I think it's best for them to say. From my own side. I will say the patients are not really happy, the pill burden, the lack of financial support. I don't think they are really-really happy about that. Some of the things. Some of them were at the point of life and death and some were in the illness phase, they just want to come out of the illness phase, otherwise I don't think they are so excited about it. But they are the ones to answer that. |
| Okay, in your state now because you're speaking for the state has there been a strong need to increase or reduce the decentralization process? | Well, we have actually increased recently but that's on paper, but physically we have not done that because increasing the site will mean all the resources. We are going to have an accumulation of trade for that site, which is not too good for us. But on paper we have it that we have 5 new OPD sites. We noticed that some clients were around there. Apart from that we have not seen any reason to increase it. And on that I think we should strengthen what we have on ground before increasing the site. |
| Okay. But has there been any need to increase or decrease | No need |
| No need to crease or decrease. | No need to reduce no need to increase except to strengthen |
| Okay the basic thing now is to strengthen | Yes |
| Okay, why do you support to strengthen then any major | Strengthen Capacity building a little more funding of the activities of the OPD, and even for the patients. |
| More support for the patients | Yes, more support for the patients. Transportation cost.Yea, transportation for the patients both for the OPDs officials both the state team and the local team, I think they need some kind of support. |
| Okay. So, we are coming to the support. So what supports are available to help you to adapt or to adopt decentralized DRTB services in your state. | What supports are available. Not much because I think I think nationally we have the state and the majority are supportedd. I think whatever is made available to us is not adequate at the moment maybe towards then but currently is not adequate. But for those that are driving late to the national program, I think the program is a little robust. So, my plea will be that let some of the states, let KSTB as an organization if they still continue to support the PMDT they should still look at what the national is doing in regard to PMDT services and try and adapt it |
| So, you are saying that there is a difference between the two | Yes |
| So which one is better then | I think the national seems better |
| So, for your own state what support are available, let's go down what KNCV is doing or has been doing and then let's see where the gaps are perhaps | We have two major partners that are totally in tune with our activity. KNCV manage more of the transportation for the healthcare workers to and fro the OPDs site. Maybe lunch or support for the technicians while ARFH handles the support for the patients that's social support transportation support and equally laboratory support because they help to manage the payment for the test both the baseline and the follow up test so for both of them, I think reviews need to be done for us to move further because we currently will be having low OPD attendees and of course, there is a fuel increase and some of them, how much are they paid. One thousand and you know one thousand can technically take you nowhere. And these people come from some interior area inside that it will take at least three thousand one way to take you to the OPD site, even though we are not expecting the program to handle all of that but at least let the amount be raised. |
| So, are you saying the supports are not enough | Yes |
| But I want you to point out the exact support apart from the support for technicians and OPD attendance for patients any other supports? | That's what I'm saying the patients do not have what we call transport support and also social support. Transport support is supposed to transport them to the OPD site and back, which is a thousand naira. We all know a thousand naira can hardly move you within an urban area to and fro not to talk of rural area and also the social support is meant to give them at least two thousand naira, and that social support is meant to give them at least one little meal, a meal or two, and we know two thousand can do almost nothing at the moment and we know these people are people that are commonly malnourished you know, the very poor among us. So, they need a little support of what we have |
| Also, can there be any support you advocate using this opportunity? | Palliatives |
| Palliative is a complex word. | I think just a little increase in whatever they are been given. I'm not expecting that if they spend five thousand to the clinic, they should get five thousand, no. But if it is five thousand and they could get three thousand naira. Maybe we should manage things for them. Transportation comes based on their management from the OPD centers so that your coming from this village is how much the distance to the OPD. And they are paid something commensurate I think we will make more sense in that |
| Any other additional form of support you may advocate. | A little more increase in the patients visit. Because I know the sick those who go to visit them, at every point in time. And they will need a little more for what they have transportation cost has gone ahead. Speed boat for them to be able to go round to see those people you know are not living in the urban center, most of them live in the rural center. So, for that reason, they will talk for themselves during the interview? But I know they should give them more access to visit patients and know how they are faring, that's for those for case study base that give them more environment to track those patients that has been diagnosed and bring them to treatment. |
| What kind of incentives now are there to ensure the maintenance of decentralized services | I just mentioned all of them |
| Which of them is successful | Well, the incentive, the success rate I can't tell but I think at least for the patient test |
| Okay. But you believe if all these things are in put the whole thing will work | Yes, it will work. |
| How do you think that your state setting in the state will improve or reduce the decentralized DRTB services. The settings in the state how did it help, how did it affect either for good or for bad in the concept. | The setting is good enough. We can touch ever local government comfortably from here without having to go for 6 to 7 hours you know, it makes a lot of sense and again for some reason we have the treatment center for those that are very ill. And the treatment center is located at Uyo. So that when they are admitted they are closer to the state team and also to the consultant. And for any reason they have any issue, we are standby to give any form of assistance technical or otherwise for the management of the unit. I think the architecture of the state is good. I think is just a little more here and there can make it to work even better. |
| Ok. So, what has been your motivation for wanting to help for sure the implementation of decentralized DRTB services. | My motivation is the fact that we have seen that decentralization has been moved and it's getting better. We've seen more cases we are having better enrollment you know, and I think the case is there is a little better. Even though 8 wasn't in the system we have other previous method that was used for PMDT, but I think overtime we've been getting it right. We've been getting enrollment line. Last quarter we had a total enrollment of about eighty one percent coming back from as little as forty something percent. So, I know that it actually much more better than the way we met it. You know like Oliver twist will always want a little more because one person is a whole life you know, so 87 % means that two patients were not enrolled. 80 will look like a good deal but two patients maybe, breadwinner, mothers, daughter. So, anything less than 100% is not really a good deal. |
| Okay, thank you.So how confident are you about being able to implement decentralized DRTB services consistently in your state | 100% confident. |
| What guarantees that position sir | Well as healthcare workers we are trained to save life and that's what we are called to do, and that's what we are paid to do. And for that reason we will always be there to make sure the system works. We won't allow anybody to just dangle with it. So, whatever is the case we will always be out there to take care of the patients. No matter the formation through the decentralization we will always look for a way around it. To make sure they are ok. I always tell my people in the program that one patient means a lot, that patients are somebody's parents, somebody's child., somebody only child, somebody's husband or wife. So, for that reason we are always called to duty to do what we are supposed to do. |
| Okay. Now you have spoken for yourself, what about your colleagues or your team. How confidence are they too. | I have a very active team I must say. I was DRTB focal person before and the way on which the program has gone after I became program manager is so amazing. I think I have a group of well committed people in the system. The CBOs, media team focal person, the logistics person all being up and doing to make sure that every patient diagnosed gets treatment. So, I will say they are very enthusiastic if not even more than myself |
| Okay. You are confidence about the willingness of your team | Very-very sir. Very confident |
| So how well does decentralize DRTB services fit with your existing work Processes and practices in the state. | Well, I inherited the structure I saw. Even though we have done a little tricky since I came into the program. I think it's the best we have. Because if we are to tell the whole patients to come to Uyo, it will be a very big load to us. We will not see many patients coming down here, you know each OPD patient spent up to a minimum of 4000 naira, for patients that are not in that local government to come and go. Some could cost as much as 8000 naira to access Uyo if it's not decentralized, but for the decentralized point I think obviously it's cheaper for some point and we don't do it based on capital area we do it based on the one closer to you. which one is closer and cheaper to you that is the to attend. |
| So, will decentralized DRTB services replace the existing programs. So, what's your position on that. | I'm in the decentralized position so let's leave it as that. I came into that and I met the structure like this, we twist it a little bit so what I am working with now is what I met, so I don't have a comparative knowledge between two different programs. |
| Okay the major thing is that you are in tune with what is happening now. | Yes, and I think that's the best thing that is happening now |
| Okay, I was trying to get the component I'm missing it so that you can be above the ground, I have almost finished with the questions in my list I think that will be good enough with the way you were thinking that. There are so many, So let me find it so that we can rank it and I think it will help us. Ok in any case let me see what I will do. | I will say that decentralization, good initiative, good interventions when it comes to accessing for case holding for DRTB but I think a whole lot more needs to be done, along side to strengthen it. It's a good structure but I think with strengthening we will be able to achieve more than what we've already done. |
| Okay,so any other thing u may need to comment on the decentralized DRTB services? | No |
| Ok, thank you so much | Thank you. |

**Title of Interview/discussion: KII/B14**

**Date of interview:**

| **Interviewer/facilitator/questions** | **Respondent/responses** |
| --- | --- |
| **Good afternoon sir.** | **Good afternoon.** |
| So, I will start at once.So, what do you think of TB Reach Wave 9 which centred on decentralized DRTB treatment services? | Well, the decentralized DR-TB is a welcome development; In the DR-TB Reach Wave 9, when it came up; it became more active like now we have three DR-TB sites carrying out Base-line investigation. |
| Okay.So, what do you like most about this concept?What is so wonderful about this concept by your own estimation? | Apart from the fact that a lot of people are still yet to access the services, it has helped because the people in the rural areas can now have access to the treatment; actually they were thinking it was witchcraft of a thing but now they understand that it is TB, that it can still be managed and a lot of them have come up with the reality of the sickness about its’ treatment and cure. |
| Okay.Is there any no aspect of the concept; is there any of the concept that is not fitting? | “Long pause” |
| I mean the decentralization process; is there any aspect of the concept or anything about it that is not so fitting? | Apart from the difficulty of the patients coming out maybe because of bad economic situations that hinder them not to come out freely as they should have been doing. |
| What do mean by coming out freely? | The patients; the DR-TB patients coming out for Base-line investigations at OPD centres due to finance. |
| So, what has influenced your practice of decentralized DR-TB treatment services? | Occasionally courtesy of the agency for instance GeneXpert machine was brought to the state by the agency; after we have received about 40 pieces of the GeneXpert machines; we were very happy running the tests and after that it was a very long apart from last week when I returned from zonal meeting that we were able to borrow about 26 pieces from Rivers state and that is the issue. |
| How did that come to the decentralization concept? | You said decentralization? |
| Yes.  What has influenced your practice of decentralized DR-TB treatment services? | “Long pause” |
| What and what influenced your support for it? | Support? |
| Yes, you are working towards the success of it. | Towards the success; if we can have financial support. |
| No, we are talking about you now; you as the focus person; what made you to support this decentralization process? | What makes me to support the decentralization? |
| Yes. | The provision of accessibility to the affected patients; like before they were unable to access treatment but with the decentralization, they are always on treatment because it is nearer to them. |
| So, what pleases you the most in this decentralized DRTB treatment services in the state? | Working towards success. |
| In working towards success, what and what do you think is so wonderful or do you think as good? | It is good; like I said, it helps the patients; it helps them because if you look at these people; some of them are very poor and a little thing like this brings them back to life; it has encouraged them to come out and get themselves treated. |
| Okay,Any challenge so far to the decentralization process?  Did you witness any challenge? | The challenge I feel is that it is still grossly inadequate based on the size of the state; the structure we have based on the decentralization is not enough; there is need for expansion so that TB treatment and services can get closer to affected people and this will better the system in the state. |
| Are you advocating for more decentralization so to say? | Yes, expansion of the decentralization but we also have to look at what we have at the moment that is what they are on the ground; so I would have suggested strengthening of the existing structures and look at how it can go. |
| Okay.But during those decentralization periods were there challenges?What challenges did you encountered? | Finance; financing. |
| Okay.Financing; in what way? | Getting the pre-sample, the labs. work optimally; in other words, been equipped; properly equipped was the problem. |
| Okay; okay.How do you see the decentralized DRTB services compare with the routine existing program that normally do?  How did you see the two before this decentralization and during the period of this decentralization? | We have the routine service and then we have the DRTB service which was almost part of the routine laboratory services. |
| No.Here we are comparing the decentralization and the normal way you have been doing DRTB services; what is the difference between the two? | Okay; the difference, not all health facilities have DRTB activities; but for DR-TB to be taken to zones; for example, we have facilities at Ikot- Ekpene zone; we have Eket zone and in Uyo area we in Oron. So, it helps taken the services closer to the people; it is much better when we compare it with the existing one. |
| In what way is it better than the other one? | Now, before now; when we have only but one facility carrying out this test in Uyo; It means that people from Mgbong will be coming here and for them to come here, it takes them about two days to come here; for people to come from Orong-Anang to carry out Base-line investigation here; it is costly and very-very discouraging. So, this decentralization has helped in getting them to come; encouraging them to come, either for the OPDs or for the first time Base-line investigations. |
| Okay.What are the advantages do DRTB decentralized treatment services have over the normal one that we are all used to? | The decentralization always helps in harnessing the treatment; the coming out of patients to get their treatment than when it could have just been one facility or one treatment centre carrying out the Base-line investigations or the OPDs; so, it has more advantageous spreading this; the reason I said this is if finances are available we could have more of the centres. |
| Okay.Any other advantage? | For now, there are no other ones. |
| Okay.Any disadvantage? | I don’t see any disadvantage. |
| What kind of changes do you need to make for decentralized DRTB treatment services to work effectively? | “Long pause” |
| Was there any change you personally made to ensure that the services worked; the decentralization process worked? | Maybe, in terms of communication? |
| No, as a person; maybe you adjusted one or two things; or one or two schedules; or one or two approaches? | I don’t think I made any. |
| Okay; it functioned effectively in the same way you were doing at the previous times? | Maybe instructing the focal persons to give preference to the service coming into the field; maybe they have more than one activity hence the TB activity does not bring revenue to the facilities; so we encourage them to do that not minding it does not bring revenue to the facilities; so we insist that they should in order to save lives. |
| Okay.Are there components of the intervention that should not be altered or should not be changed?You know, I was reading the interventions:   1. Is GX alert system. 2. Is improving tracking of patients by supporting the global fund-funded community organizations. 3. Improving counselling of patients through introduction of structured pre-treatment counselling and team counselling with DRTB survivors. 4. Decentralizing Base-line investigations; you should be familiar with that? 5. Providing transport supports for patients for Base-line investigations. 6. Is employing mobile connectivity solutions to improve turn-around on Base-line investigations. 7. Is verifying the reported pre-treatment tests using verbal autopsy. 8. Is decentralizing treatment initiative to the local government. 9. Is the engagement of states’ volunteer liaison officer.   So which one do youthink should remain intact? | The decentralization and the payment; improving stipend to the patients that will come for the Base-line investigations. |
| Okay.Any other one? | Another is employing mobile connectivity solutions to improve turn-around on Base-line investigations. |
| Okay.So, those ones should remain intact? | Yes. |
| Alright.How complicated is the decentralized DR-TB treatment services; the program, how complicated was it? | It was not complicated because it is routine service; so, it was not complicated at all. |
| So, how do say it is not complicated at all? | It is because we were doing it as if we are doing our routine services. |
| Okay.How well does the decentralized DRTB treatment service meet the needs of your patients; does it meet the needs of your patients? | Yes, because the decentralized DR-TB treatment service was carried to the health facilities closer to the patients; it meets the need for this decentralization. |
| Okay.That means one aspect is of meeting the needs of the patients? | Yes sir. |
| Any other one? | All other ones are still encouraging but getting treatment services closer to the patients remains the best. |
| Okay.In your facilities and in the state, has there be strong need to increase or decrease the decentralized DRTB treatment services? | “Long pause” |
| Has there been any need? | It is bringing up day-to-day running of activities in different zones, and encourages expansion. |
| Okay; okay.What we are saying now is; is there a strong need to increase or decrease the decentralization process? | There is strong need. |
| Is there any need? | Yes. |
| Why? | There is need because if you come to a place like Oron-Anang; it is very far away from here and if you look the local government like Mgbong, Urunko, Eastern Ugborong; they are very far away from here and then, they still belong to this state. so, there is need to take these services to them and that calls for expansion. |
| Okay.What supports are available to help you to adopt the decentralized DRTB treatment services? | Supports to the treatment services? |
| What supports are available to you now to supports the decentralization? | If you go down to Egunegbong, you have facilities there that we can recruit and add to what we have; if you go down to Mgbong; you will be able to see at least some private facilities that can be included and added to what we have. So, the availability of these structures support. |
| Support is maybe coming from health facilities or the partners; are there supports that are encouraging this decentralization? | Yes, the availability of partners; at least we have KNCV; we have....we have other partners that are supporting the state. |
| Okay.So, what kinds of incentives arethere to ensure the implementation of the decentralized option? | “Long pause” |
| Any existing incentive for that process of project? | The state don’t really have; maybe we are looking at partners to help us with that; like the RedAid periodically coming ; the KNCV and other partners have been helping the state. |
| Okay.How do think that the setting in the state influenced or have affected the decentralization process? | Thy setting? |
| Yes, the setting; the structures in the state. | Just like I said; apart from the private facilities; it only helps all the health facilities because it has spread round all the state. |
| Okay. | With this; by including the private facilities, this can support the decentralization. This is because we still have something to pick up and use at all the zones. |
| Is there any other thing? | “Long pause” |
| Any other support? | None for now. |
| So, what have been your own motivations for wanting to help ensure the implementation of DRTB decentralization project? | For me, many would have died before now without the project, because it is lives saving; it is my encouragement because I feel glad for saving lives. |
| Any other thing? | No. |
| How confident are you about been able to implement the decentralized DRTB services; how confident are you? | I am confident because I have capable hands that can carry out the needs for this decentralization. |
| That’s makes you confident? | Yes. |
| Okay.What about other health workers; are confident about their intentions or ability or willingness to supports and continually supports the decentralization processes? | I told you that I have confident in them; like those doing the Base-line investigations are doing it impressively because we have a system that calls for review that comprised of items in the Base-line lists. |
| Okay.How well does the decentralized DRTB treatment service delivery fit into existing your own work practices and programs? | Like I said before; that we are doing it routinely apart from few ad-hoc staff recruited by partners; other staff civil service; or facility staff who are doing it as part of their routine services. |
| Okay; so, that’s a very good plus to it.Any other thing? | “Long pause” |
| Now; will the decentralized DRTB treatment service replaced or complement the existing approach? | Come again? |
| Decentralize DRTB treatment services; do you advocate that it should replaced old order or it should be adjusted? | It should complement. |
| It should complement? | Yes sir. |
| So, not total replacement? | Yes sir. |
| Okay.Is there any other comment you wish to make? | I want to make this because I am dealing with RedAid and we have worked before now; recently we assessed a laboratory in Eket zone for the first time they carried out the Base-line activities and the complaint now is that it is difficult to carry out the ECG and I remembered that prior to the visit, I may not be very specific, I attended a zonal review meeting and I was given a reportable ECG and a printer to give to that facility and I think since then that facility is working happily; they also appreciated other things like when they look at low charges and I wish the same thing is done to that one at Eket. |
| Okay, whether that will split them up to work very well? | Yes sir. |
| Okay. | And again; I don’t know how you can assist incurring SDR cards that is use for DR-TB; yes we have enough geneXpert in the state but the major problem is the cartridges; like I said as at last week about 26 of them are nothing to write home about, they can’t even see three-quarter; so I don’t know the sources of procuring the cartridges in the country; so, state can assist in the process. |
| Who procured the first machines? | NTSTB. |
| NTSTB? | Yes, so we are working on colour ten before this DRTB. |
| Okay. What does colour ten do? | You know the other GeneXpert were just six before they brought the colour ten; so, it all about the number and the colour when they were brought. |
| Okay. | Yes, it is a better alternative culture; so, if the supply of cartridges have been a limiting factor and if this limiting factor is not there we can work. |
| Okay, any other thing? | No sir. |
| Thank you very much.  That is the end of the interview with the State Quality Assurance Officer. | Thank you sir. |

**Title of Interview/discussion: KII/B15**

**Date of interview: Tuesday, 8^th^ August, 2023.**

| **Interviewer/facilitator/questions** | **Respondent/responses** |
| --- | --- |
| **Good afternoon ma.** | **Good afternoon.** |
| So, our work is on decentralizing TB Wave 9 intervention which was on decentralizing DRTB services.So do you think of TB Wave 9 intervention to decentralize DRTB services? | I think we should we should go on the way it is by making people understand what is DRTB, because I still think is not everybody that has understood it. |
| Okay. | Yes. |
| But we are focusing on these interventions; how did you see these interventions that came up with TB Wave 9 project towards decentralizing DRTB services because all DRTB cases needed to go to the state capital before they could be enrolled in treatment; investigations or for OPD services but with this interventions; services were decentralized, I think Eket is one of them, Oron and Eket-Ekpeneg; so, how do you see this intervention; how good was it or how wonderful was it? | Before, because I still remember one client, that could be two years ago who was a DRTB patient, the mode of the treatment was okay, but now the mode of the treatment; you know before you commenced the treatment you have to take the person to Base-line; though the group here, sometime they normally come with their machine to carry on the Base-line here. So, sometimes by having the DRTB patient, sometimes they find it difficult to go there because of the transport they do give to them; some of them even complain that the problem they are facing now is the cause that they cannot meet up with transport and sometimes, some of them are living or coming from very far places; so before they be here to get their drugs or the doctor to check on them; they used to complain that they don’t have transport fare for that; that they should be something more than what they now collect or receive as transport support. |
| Okay, but how do you see their coming here instead of going to Uyo for such services? | Their coming here; they like it and the thing is more preferable than going to Uyo because they still get the same treatment, attention and services. |
| Okay. So, what is the most important thing you appreciated in this TB Wave 9 intervention? | The most important thing is the drugs; the patient goes straight with the drugs without missing for a day; I think at the end of the day or end of the treatment the patient will be cured. |
| Okay.Our focus now or what we are now looking at is this decentralization whereby person will not go to Uyo to pick medicine, instead go to Ikot-Ekpeneg, Oron or Eket; how do you see that approach? | I think the people that are around this Eket housing should come here to pick their drugs. |
| Okay.How do you think that they like it? | Most times they are here I do ask them; how did you people feel while coming here?  But they said that here is closer to them. |
| Okay.So that is the most important thing? | Yes. |
| Is there any other aspect of that intervention that wasn’t good? | “Long pause” |
| Our focus is on DRTB intervention to decentralize DRTB treatment services. | There is no any other thing that is not good. |
| So, what do you think affected or influenced your support or practice of this decentralize DRTB services? | Is transport fare. |
| No, for you? | Okay, for me? |
| Yes.What made you to support it? | The transport fare. |
| Still the transport fare? | Yes. |
| Even before the hike in prize of fuel and transport fare? | Yes, the patients have been complaining of the transport fare they received and sometimes they think that those days they come for OPD that they should be giving something on their way back. |
| Okay.Is there any challenge with this decentralized DRTB services? | So many! |
| What are the challenges? | 1. You know sometimes when someone is affected with TB, the affected person thinks all the hope is gone; so when you tell the patients to take their drugs especially the blood medicine; they complain they don’t have money to take blood medicine. |
| Any other challenge? | There is no any other challenge. |
| Okay.Do you think that patients face any challenge for coming here instead of going to Uyo for their treatment? | It is only but the transport because they think here is closer for them to get their drugs. |
| Now, how do you compare this approach where DRTB patients are treated in other centres rather than going to Uyo for treatment?Any difference between these two approaches? | No difference but you know here if you keep the drugs at Uyo it might even take them time to get the drugs but since here is closer, they can easily come often. |
| Okay.So, the closeness is the most important thing? | Yes. |
| So, what are the advantages for coming to these centres for DRTB medicines instead of everybody going to Uyo; do you think there is any advantage? | There is one of the DR-TB patients I took to Uyo; when we were inside the vehicle; when the person was coughing, the driver, the passengers started shouting “haha!....haha!!....this kind cough oh!!!... we don’t want it oh!” it was as if they wanted to bring the person down. |
| Were you with the person? | Yes. |
| Okay.That means if the wasn’t going to Uyo, such a thing couldn’t have happened?  Is that what you mean? | I think so. |
| But I asked for advantages; them going there and them coming here; what do you think is the gain? | Here is closer to them. |
| Okay.Apart from that; any other thing? | No any other thing; still the same drugs that we get there. |
| So, any disadvantage with that approach?  By coming here instead of going to Uyo; is there any disadvantage or that? | “Long pause”  Sometimes, if you tell them to go Uyo, they may not even go; they would stop half way and say that they don’t know the place, unless there is a motor to take them there which cannot be possible; maybe today is the day that I have chance to go; another person may not have chance. |
| Before you started this decentralized DRTB services; did you make any changes to the work plan you already have? | No. |
| In taking of DRTB patients?  Is there any form of things you adapted or adjusted? | Before, you know this was coming in an injection form; so before, I do go there every day to give them the injection but now that it is tablet form is more preferable. |
| Which one do you people use now? | The tablets. |
| How many months? | 9 months. |
| Okay.Now, there are components in this DRTB treatment decentralization;   1. There is what they called the GX alert. 2. There is second one whereby the existence of liaison officer. 3. The third one used the DRTB survivors for counselling purposes. 4. Then, the other one; they decentralized lab. Services whereby people can go other areas to do their lab. Test apart from Uyo. 5. Then, another one; they provided support for people to go and do those lab. Services. 6. So, which of this component do think worked very well? | Going to facility to do the test. |
| Based on these components that I named; did you people have any DRTB survivor that was here giving counselling to the DR-TB patients? | No. |
| But you people were doing test here instead of going to Uyo? | Yes with the GeneXpert. |
| So, which this component is very good or worked very well? | The GeneXpert that we are using here; I think that one is the one. |
| Okay.This decentralized DRTB services; how do you rate it?  Do you think that it is a complex or complicated system? | It is a complicated system. |
| What makes it complicated? | It is complicated in the sense that when the patient is not taking the drug correctly. |
| Okay.You know our major focus is taking these medicines from centres outside Uyo; you know before in DRTB management everybody must go to the state capital before they could do test even Base-line test; do you understand? | Yes. |
| But now, what this intervention did was to ensure that people could go to other facilities other than the state capital to be able to access these services; so that is the full system we are focusing on. | Please come up again. |
| You know in DRTB before, everybody must go to the state capital before they could get their medicines, before they could do test and before they could be enrolled into treatment to start with; but now with this TB Wave 9, part of what TB Wave 9 did was to bring in certain innovations to make possible that people don’t need to go to the state capital to do all these tests and other things; so what I am asking now is; this approach how do see it as far as the patients are concerned? | What I am seeing is that taking the drugs to the facilities that are closer to them is the best approach. |
| What supports are available for this decentralized DRTB services?  Are there any supports maybe from NGOs, CBOs, and CSOs either to the patients, health workers or to the Community Based Organizations? | I think that is the transport fare. |
| Do you know the people that give the transport fare? | The KNC. |
| Okay.Which people does the KNS support? | “Long pause” |
| Clients or the health workers? | The health workers. |
| The health workers? | Yes. |
| Apart from that; any other thing? | They do support the client too. |
| In what form? | Still transport. |
| Still transport. Okay, they support both the health workers and the clients? | They support the health workers but they don’t support the patients. |
| Alright.Any incentive apart from the support?  Incentive either to the patient or to the health workers? | No. |
| Okay.How did you people coup; because DRTB people come here? | Yes sir. |
| How did you people coup, adjust or the changes you have made to be able to take care of these services which before now has always been at the state level? | Nothing has been provided for us. |
| Like what do have in mind in terms provision? | I think something to use for self protection like nose mask; you find it difficult to see it here. |
| Is it only here or like that in every other place? | I think the nose mask we are supposed to Use here is the M3 not this ones. |
| Thank god for this one madam. | “Laugh” |
| “Laugh”  Okay, you are saying that there is no support for you? | Yes; no support. |
| But you people have nose masks apart from the fact that you are looking for M95? | We have only but the disposable ones. |
| Okay.And you think it is not okay? | It is not.Why I said is that there is somebody that came from Uyo that time they used the ones they should have given to all staff as we are working here; because they are not using that disposable ones; they use that M95 but we are using this disposable ones. |
| Okay.What have been your motivations in supporting this taking care of DRTB patients here? | Because of the experience I have even in IDH. |
| You worked with IDH? | I did my IT there. |
| Okay.  So that is what motivated you to take care of DRTB patients? | Yes. |
| Any other thing? | Just to help the patients. |
| Just to help the patient? | To make sure nobody dies. |
| Now, I am talking more about DRTB patients coming in here instead of going to Uyo; how confident are you that they will continue to come here to get their treatments? | They like coming here. |
| Why do you think so? | Why I said so is because even the patients that were with me 3 or 4 years ago, they refused going to Uyo for their drugs and treatment; sometimes they asked me get the drugs for them even when there is need for the patients to go there for physical examinations; but when the services were brought here for them to be coming here, they were so happy. |
| Okay.So you are sure it will continue? | Yes sir. |
| What is giving you this confident that they will continue to come? | Because it is not too far. |
| What of other health workers; do you think that they would work towards the patients continue coming here to take their medicine? | Health workers? |
| Your other colleagues; do you think they will support this project that DRTB patients will continue to come here to take their medicines? | You know there is no any work that you are working with happiness that you will not want to continue with it.  So, if you people can make us to be happy the work will continue; if you make us to smile, they will be happy to do it. |
| Okay.So, how well does this decentralized DRTB treatment services; how did it fit into your own existing practices and processes? | “Long pause” |
| How did it fit in?Does it fit very well? | Yes. |
| Before you were not taking care of DRTB patients, or were you? | Yes. |
| Were you? | No. |
| Okay.So, now that you are taking care of them; does it fits into your programs or your practice? | Yes. |
| How? | Because of the experience I have and I have been here for almost 4 years. |
| Okay.Now, DRTB patients are coming here to receive their treatment outside the state capital; now do you want this system to continue or do you think that it should moving hand in hand with those ones going to Uyo to receive treatment? | No, they should be coming here. |
| Why do you support them coming here? | I have said already that they like the mode of their coming here to receive their treatment. |
| Okay. | You know anything you are doing you have to look unto whom you are dealing with; so they like coming here. So, even after the test and realized their RIF; even if you tell them to go to Uyo they will not like it unless you volunteered yourself to go with the patient. |
| Okay.Is there any other thing you want to tell about DRTB patients coming here to take their medicines? | Yes. |
| Okay.What is it? | I like them coming here to take their drugs in the sense that here is close and something will be provided for them. |
| Something like what? | When they come here they said “haa....every day we come here, you only give us drugs, what of food?” |
| “Laugh” | I am telling you oh! |
| Have you given them food before? | We passed through a lot of things with them; they say “every day take drugs; what of food?” |
| Do you think the money or support they are been given is enough? | It is not enough like somebody coming from Ikot-Abasi; I think the amount is N2,500 and this is not enough to take someone from Ikot-Abasi to and fro. |
| So, you think the money should be increased? | Yes. |
| To how much? | To N10,000. |
| Okay.We will tell them. Is there any other thing you want to say? | No. |
| So, thank you very much. | Thank you sir. |

**Title of Interview/discussion: KII/B16**

**Date of interview:08/08/23**

| **Interviewer/facilitator/questions** | **Respondent/responses** |
| --- | --- |
| What do you think of TB ----- Project which is focused on decentralizingDRTB treatment services? | Okay, the project is a welcome project because it will help us to achieve our aim which is 100 percent attendance of OPD for all patients; thereby helping us to follow up and monitoring of these patients as they are on treatment. It will also help us to be able to access our patients because by decentralizing it, a patient now will not have a reason for not attending OPD thereby making us to monitor and see their well-being.  So, it helps us very well. |
| Apart from that intervention, what do you like the most about the name TB ---- intervention or project? | What do I have to? |
| What was the most fascinating aspect of that project? | Okay, the most fascinating aspect of that project is actually the support that is given to the patient that comes and also with that, in the labs, more people are now involved because we are now going to be involving more facilities and labs. So, it is not going to be one person now. More facilities; more health workers will now be involved in the management of DR-TB treatment. We will give other people in the labs the opportunity to know that activities like this is going on.So, it’s interesting. |
| Okay, is there any aspect of the intervention that wasn’t so wonderful? | Yes. All aspect was good; this aspect of the patient will have to wait in particular lab assigned by the programme because it was far; but thank God it is now decentralized. And, we have also this expectation of the patient coming in. some of them from the communities and they don’t have account and they have requested to pay money into the account and there are some Local Government Areas that don’t have banks. So, they have to get another account in order to pay and access that service. So, some of them end up maybe spending half and getting half or little of that service. That is where the issue is; especially the new clients. They must have account before a service is given. |
| Okay, that is an interesting observation. What affected of influenced your practise of decentralized DRTB treatment services in your state?  What made you to be in support of it? | What makes me to be in support is that because of the decentralization, more sites have been able to attain the monthly OPD and we can now effectively monitor these sites so that we can get the results unlike the other ones where some use to have an excuse like mostly transportation or at times, it was raining heavily. Some of them, the Local Government or the community they are is difficult to access transportation, so they will have to look for bike and delays some will now arrive late and may not access treatment as required.So, by decentralizing it, things like that will be taken care of. |
| Okay, let me ask you on a personal note, what were the challenges you encountered in the decentralization project? | A little challenge I encountered you know we have to use facilities to bring in patients that are for this; and I know there are some facilities where no TB patient comes in; maybe they are taking to other facilities where they can go to with their face masks. As they are talking to them with their face masks, the staff there may feel that they are not safe. We need to talk to them. So, with that, because of talking to them, assuring them that with this kind of treatment they are in, they are safe. They are less infectious because they are taking their drugs and I think some of them have started calming down. So that has been the issue and their fear as they are on face masks and other new patients coming in will think that something big is on the way. |
| So how has the decentralized DRTB treatment compared with the routine old method of centralization? | There is a very good result compared to the formal one especially as I said in the attendance of OPD where the patients can now walk into the facility and access their treatment. And also, the supervisor does not need to be running around pleading and begging the patients and trying to convince them, because of the distance the supervisor had to come and talk to them while some of them will say they don’t have money and time. But now, since it is just close by, they can now say we are going to this facility that is within the community. And another thing is that it helps the patient to attend OPD on time because when it was one like I said, some of them will have to come in from their different communities and it takes time and it delays. But now, since it is not far, they can come and they come in on time. And they come in in their good number. |
| What do you think are the advantages of the decentralization | Advantage number one is that it helps us to achieve our goal – OPD attendees of patients. We are able to see our patients because now they can come and we will see them and access them and give them drugs.  Now the pharmacists are also in the team; they come in with the drugs and then there is no stock out that will make us to start planning on steps to achieving it.  And also other health workers are now involved because as they are using other facilities, they now come to know what DR TB means. They patients are there and it will now make them to deliver well. |
| Is that all for the advantages? | For the advantage also it saves time. Like I said, we don’t have to wait for a patient to come from a very far distance before accessing treatment. That is, it for someone coming from a far place; he might be thinking that her condition is too bad that until he gets to Uyo before he will be well. So, now the person can relax and get treatment here. |
| So, any disadvantage? | The disadvantage sometimes, we are looking at stigmatization- sometimes they are afraid their friends or neighbours are now aware of their condition by seeing them. I think that is the one I can remember for now. |
| Okay, thank you for that contribution. So based on your own routine, what changes did you make that made you participate and ensure the success of this decentralization process? | The changes that I made was that I was able to include facilities that are selected as sites, that the health workers accept and we had to prepare their minds. So, it now increased more health workers coming in to enrol |
| Okay, any other one? | Changes like more people are coming and more service providers also. |
| Are there components of this intervention that you will not want to be changed at all? | Not to be changed? |
| Yes, the components. You know we have many components- the GXalert, the state liazoneofficer, use of GRT to survivors and also paying for baseline examination to the decentralization of labs, support for baseline examination and all that.  Which of these components do you want to continue that wayunchanged? | I think all the components are okay and should be continue being in use. |
| If you are to choose one as the best, which one are you going to choose?Which of the components of the project do you think work best or do you think is the best? | Okay, it is actually the start things that are given to the patients and the one that work best as they said is sponsoring the baseline investigation because you know most of these investigations are very expensive and they need them before commencing of treatment.  So, if they were to depend on the patient before they can do xray; some of them may not meet up and it will delay and then at the end of the day, some of them will not receive treatment. That very intervention is very good because it allows us to commence treatment immediately. |
| Okay. But you know when we come to the components; you know the component that concerns labs is decentralization. We have been praying for that baseline investigation so we are asking for those components like there was part of the components, there was the appointment of the Liaison officer for the state. There was GXalert, there sponsored intervention for baseline investigation; decentralization of labs; counselling of the TB survivors and all that. So which onedo you think should come first among them all in terms of workability – how the work? | The GXalert is good because it makes the supervisors to know that they have these things in their Local Government before any other thing, at least. So, if the supervisor has this knowledge that there is DRTB treatment in their Local Government, it will make them to deliver. It is a very good intervention. |
| Okay. Do you think in the Decentralization of the DRTB treatment, do you think it is complicated? | The decentralization? |
| Yes. | I don’t think it is complicated. No, it is not. |
| What made you think so? | It is not complicated because it is actually the same thing we have been doing; only that it is no longer done in one centre but in different centres. It is the same thing and it has not been changed. Only that everybody is not coming to one centre now. |
| Okay, so based on that you don’t think it is complicated? Okay, thank you. So how well does the standardized DR TB treatment service meet the needs of your patients? | 1. The patients now are relaxed. 2. They are ready to attend their OPD monthly as required from all the patients. 3. The supervisor or the health care provider is able to do his work with ease because he don’t have to move the patients rather the patients just walk in to them. 4. Also, they are able to move within and not a far distance. There is control of the infection. 5. It also reduces stigmatization because sometimes ago, we were moving some patients and inside the bus, because the patients were wearing nose masks, other patients in the bus now thought that their case was so severe as they wore face mask. But now because it is a close by facility, it has been reduced. |
| Okay, | And let me add one thing; we do follow up on the patients – follow up in investigation that is needed for assessment- now this patient because he does not have any reason to miss OPD, we are able to collect the sample(especially the sample we have done before) so that we will confirm whether it will be continued or whether if it is a distance and this patient cannot attend OPD. And if we don’t collect the sample, we may not know the person that has failed before. So that is it. |
| Okay. Is there a strong need to increase the tools in the decentralization process | Is there? |
| Is there a need to increase the OPD sites in the decentralization or even to decrease? | That is to centres further? |
| Yes, that is the decentralization. The involvement of health facilities? | If we are using one facility per Local Government, there is no need having all the facilities in a particular Local Government. Maybe you can use two or three Local Government and now use one as the centre. I think it will be okay than having two in one Local Government. |
| Okay, you are saying that you can still decentralise further? | Yes. |
| Okay. So what supports are available to help you adopt the decentralized DRTB? I mean support from agencies and validations towardsdecentralization. Did you receive? | The NGOs are doing their parts-they started by giving drugs to patients and also taking these patients to lab. They support the patients. |
| Support like NGOs taking responsibility of one thing or the other. | ……………………….. Silent |
| Okay, apart from support like going to the lab for the patients, any other things? | For OPD, I think they do support for the counsellors to come and counsel the survivors |
| Okay the support the counsellors? | Yes, they support the counsellors. I am still seeing it as a support because the capacity of the counsellors needs to be built towards that and so the NGOs re doing that. |
| Okay, what about incentives towards the success or successful implementation of decentralized DRTB services? Were there any incentives that were offered? | I don’t think there was |
| Okay none was given?  Okay. | Yes. |
| So how do think that your facility or Local Government culture and setting accepted in the implementation of the decentralized DRTB services? | You mean how they are affected? |
| Yes. Your Local Government setting and culture. | We now know generally about the issue of stigmatization as I said before; and now we are going through the community to treat their brothers or maybe their neighbours. Also the facilities, most health workers are still experiencing cold of DRTB and again thinking it should not be managed in their facility- that it should be taken to where there is isolation and the vaccination was not just vaccination. That was it and I know it affects |
| Okay, so it affected? That means it may increase the number of decentralization? Is there any other setting that improved also? | Yes, that is the culture. |
| The culture did it reduced or it increased? | You know because of the awareness programme that conducted to the communities that TB is not a death sentence on the TV and radio; people made up their minds and started coming in. |
| So it really affected? | Sorry? |
| I mean it affected the implementation that time? | You know that time they did not know but now because of their awareness programme this is going on, they have started accepting it. |
| Okay. So, what have been your motivation for ensuring the support of the decentralization of DRTB services? | One of my motivations is that the patients are happy and they are comfortable because the stress of coming through a distance is no longer there and so we can effectively do follow up and monitoring. This will now increase and improve the implementation.  What motivates me again is that the cure rate is increasing. The patients can be attended to without much stress. |
| Okay. How confident are you that this decentralized TB services will be continued? That is will be sustained? | I am confident because the programme has a goal and because of the decentralization of the treatment, the goal is maintained – our patients are now attending OPDs and our patients are properly followed up because it is closer now and now the rate of defaulters has reduced as a result of being so close and as a result of that, the success story is visible. So I feel with that result, the programme will be encouraged to continue because they have seen the report. Because the defaulters rate and the spread rate have reduced. |
| Okay, so we have talked about how confident are you; what about that of your colleagues, how confident are they? | All my colleagues are feeling that TB should be handled. We all have a common goal in ensuring that our clients are effectively treated starting from the diagnosis- let everybody know about it and then getting to also know that it is not far and we can now do the baseline without stress. And also to know that they can now access OPD and have their results and the patients are being cured. So I feel all my colleagues have that common goal and with that, every other person know about the decentralization. |
| We are almost finishing. So how well the decentralized TB does services fit into existing work processes? | How it fit, you see intending programmes and setting of treatment was not supposed to be something that is stressful in a facility that is closer to where the patient is because the patient don’t need too much spending and wasting of time and so on; so because of that, this decentralization fits into the programme by making health services of TB close to patient as the national programme suggested- it is not something you had to pay so much money before getting the services. |
| Okay. Then were this decentralized TB services complement the already existing centre method of treatment and management? | That is for patients having to move from a far distance? |
| Yes, will they replace it or it will just be used | Yes, it will have to be replaced because of there will no need to use it; for example a patient will come to you where actually there is a centre a little bit far to reach. If we have gotten these centres in other Local Governments, there is no need of still telling somebody to come to Uyo for baseline when there is a lab that is close to the centre. |
| Did I tell you about the intervention? Maybe 1, 2 3, based on your own experience and observation? Let me detail the dimension so that you can now explain better in the way you think they perform.  Number one is GXalert. Number 2 is improving tracking of patients by supporting the Global fund supported community based organizations. Number 3 is improving counselling of patients using DRTB survivors. Number four is decentralizing baseline investigation. Number 5 is providing transport support for baseline investigation. Number 6 is employing mobile connectivity solutions. Number 7 is verifying the rate of croton death using verbal autopsy. Number 8 is decentralizing treatment initiation to the LGA. Number 9 is engagement of a state liaison officer.  So which of these do you think is the first, second or third in terms of how best of these interventions? | You mean the one that is the first? |
| Yes, the one you think that functions most effectively or is very effective. | Okay, of all these interventions? |
| Yes, of all these interventions | I think the one that function effectively is where one |
| So you march it as number one? Do you march it as the best intervention? | All of them are good but the one I also want the most is tracking of these patients because sometimes, some patients are very difficult and the health providers most times find it difficult. Empowering the NGOs that go into involvement to assist in tracking and encouraging patients, I think it helps the programme. |
| Okay, any other one? | Then supporting the baseline investigations. As I said earlier, they are expensive and it is needed before commencement of treatment. And most people that have this infection are mostly the poor. So by supporting this baseline investigation, it ensures that treatment can be commenced as needed instead of somebody to say let me go and look for money. So that supporting of baseline investigation is very good. Then if I am to add number four, I can now be looking at the incentives given to patients to encourage them. We know what we are facing in the area and sometimes we have to run after them to encourage them so that they will respond more. |
| So, you are advocating on how it is going to be sustained? | Yes, |
| Is there any other comment you would want to make with regards to TB rudinal service | I don’t know if they have started ----- because some supervisors sometimes may not be able to attend the OPD but they will be able to communicate with them and ensuring that they are in the hospital. |
| Is there any other thing you want to comment on? We have finished with the established questions | There is no other thing. What I just want to say is that the intervention is a very welcome intervention. It has really helped our clients in being careful and it has been curing them and then in achieving our goal. Because once you are diagnosed, you will have to go and take treatment. It wouldn’t have been easy without the interventions. So it is a welcome intervention and we want to thank TB ream for the support. |
| So, thank you very much for your time. | Thank you Sir |

**Title of Interview/discussion: KII/B17**

**Date of interview: 10/08/23**

| **Interviewer/facilitator/questions** | **Respondent/responses** |
| --- | --- |
| What do you think of that TB reach Wave 9 Intervention aimed at decentralizing DRTB treatment services? | It is a good intervention and a good program |
| What made it good Sir? | What made it good is that treatments are drawn closer to the patients, it helps us to have immediate possible results and solution for out patients. That is the way I look at it. |
| That means that by going closer, it became of much help to the people? | Yes. |
| Is there any aspect of that intervention project that you liked so much? | Yes. Like the area of support to the patient is an interesting area because some of these patients lack things like transport for them to go beyond their environment. Actually, with this little support, it helps and it boost their lives in order for them to have timely access to their services. |
| Is there any aspect that you don’t like or considered very wonderful? | They are all very good, they are all important. Like you talked about the area of counselling and them using survivors to interact with them, it makes them to understand that these cases have been on ground before now. |
| Okay. So, what really influenced you to be part of these decentralised DRTB services? | What influenced me is that it is part of my job as it is, there is nothing that will stop me from doing it as it has been part of my job. |
| Okay. Fundamentally it is part of your job and you just have to fit into it? | Yes |
| What challenges are found in this job? | Practically there are challenges, challenges in the sense that when you are starting a project, the ability to uphold other things around it like the area of incentive to the patients and those supports. If they are not coming timely, it may become a challenge to the patients |
| What you are saying is that there are delays sometimes it is a challenge? | Yes |
| It has challenges in terms of system? | Yes |
| Any other challenges that we may also see on the part of the health worker or on the part of the patients? | On the part of the …………….., you know because of the traditional believe too there are some that even up till now, they do not want to adhere to the treatment of DRTB |
| So, they will say that they don’t want to accept the treatment of DRTB? | Maybe they started, then they will say that they don’t want anymore. The issue of counselling can be done in the community with the patients, you will have to discuss with the community leaders and community influencers. |
| What about challenges on health workers themselves? | The only challenge I have is the patients adhering to the treatment for me to have good result. If they are not available, it is bringing a problem to my personality. |
| Okay, let us look at the components of that intervention. number one is involving the GX alert that we talked about which was modified with the whatsapp group formation. | It is a very interesting component, if there is any way they can step up for us to be going directly to the patients. The supervisor or focal person in charge of the patient, it will be better. |
| If we think of scaling up of that project, do you foresee any challenge? | Maybe if there is no fund, there are areas that we may have default. |
| Number two is on improving the tracking of patients by supporting the Global Fund assisted community based organization, how do you see that component? | It’s a very good component. You know as human beings, there are conditions that may warrant the community workers to get into their homes and investigate. Somebody may be on drugs after one month, two months or three weeks and this person is no longer coming, you need to investigate to know if there is any way that they need to be encouraged, let them be encouraged. |
| Are you advocating that this thing should be scaled up? | Yes |
| Do you foresee challenges in this if you want to scale up? | Its all about money, you cannot go beyond these areas. |
| The third one is improving counselling of patients using DRTB survivors, how do you see that approach? | It’s a nice approach, a very nice approach. Unless otherwise, or if they can be using community influencers and community health workers to talk to the people that are within the community so that if a patient is living in the community, he or she can benefit from the community influencers at the same time. This type of people can always support and encourage the patients. |
| Are you advocating that this thing should scale up? | It should scale up. |
| Do you foresee challenges in this? | Its all about fund. Fund in the sense that sometimes working in these rural areas needs a lot of money, with the latest development in our society we don’t know what will happen next, like in the previous month, we were purchasing fuel at the cost of one hundred plus but now fuel is sold at over six hundred or seven hundred. |
| They are even saying that it will increase again. | The amount of money that we were using to pay transport before have changed and if the budget was not properly budgeted for, it may affect the system. |
| But, do you think that all nooks and crannies’ of the LGAs will have access to DRTB survivors who may be willing to participate in this study | Please come again |
| What I am saying is that there may be some LGA who my not have any DRTB survivor who may be willing to participate? Do you foresee that as a challenge? | It's possible! It’s possible! It’s possible! |
| Decentralising baseline investigations to more qualified peripheral centres, just like we have in Ikot Ekpeneg. How do you see that one Sir? | It’s a very good idea if you can go beyond that because we are looking at the patient interest, if the peripheral centre is close to them and once the person is diagnosed, he or she can have access to that environment, the workers telling them to come may not be very easy. If there will be more centres, it will be better for them. |
| Ohhhhh! You are even advocating for more decentralization? | Yes |
| Is there any possibility of getting lab that will do the type of lab that is required to do the baseline? | There is possibility. It depends on the rule of engagement. |
| It depends on the rule of engagement? | There are areas too where people have problems |
| Okay. The major this is that most people who have these issues are in those areas and there is nothing bad in taking those services to them? | Yes |
| What I am asking is, imaging if you go beyond Ikot Ekpeneg axis, is it possible for us to see a standard lab that will be able to do all those investigations that are needed? | Yes (not with strong word) |
| You understand what I am saying? | Yes, it is possible |
| Because nobody will build that type of lab and put it inside Ikot Ekpeneg? | Exactly |
| Fund may be a challenge | Yes |
| But I have seen that you are in support of further decentralization? | Yes |
| Okay. The fifth one is providing transport support to patients for baseline investigations, that means they are been given transport to go for baseline investigation | Yes, very necessary. |
| Do you think that the amount been paid is very sufficient for that? | No. I may not adequately respond to that |
| Okay. You are under Uyo zone, you may not have the right answer on that? | Yes |
| Okay. The sixth one is employing mobile connectivity to improve turn around time of baseline investigation. | It is very good Sir. |
| Do you have the app in your phone? | Yes |
| Do you know how to use it? | Yes, we check our phones all the time. |
| So, this is a very good measure? | Yes |
| Okay. Now the seventh one, which is verifying reported pre-treatment deaths using verbal autopsy. That is going out to find out whether that person is actually dead or not by asking question. | Yes |
| Was it practiced? | Yes, we actually practices it in some areas |
| Okay. The nineth is the engagement of a state volunteer Liasson officer. | Yes, provided there will be transportation for that person. Like the name implied, volunteer to do what is dedicated to him. |
| Do you think that should be scaled up too? | Yes, it should be scaled up |
| Do you foresee problems or challenges in the scaling up? | Yes. I know that it is supposed to be done voluntarily, if there is a little support that is attached to it, the person will be reluctant if it is withdrawn. The person may not have any other thing doing apart from the voluntary work, he may decide to opt out. That is the area I am looking at. |
| Okay. Of all these components, which one do you think is the most ideal component? | In fact, I look at all of them to be so important as far as this program is concerned. |
| If you are to vote for one, which one will you vote for? | This is a difficult question. In fact the GX alert is very important. |
| You are now talking as a supervisor that you are | Hahahahahahahaha (laughter) |
| That one has affected you so much. | Yes Sir |
| Okay. Thank you very much. We are comparing the centralized and decentralised, how do you see the two? | ……………………… (silence) |
| The time everybody was coming to Uyo to do everything concerning DRTB and now that some people have to go to Ikot-Ekpeneg. | There is a lot of advantage. It has reduced the time that is being wasted in attending to one patient. Then the cost of transportation to Uyo paying the same amount because the transport system was fixed and it may affect the system, now that the decentralization is here, its good. |
| Okay. Apart from that, is there any other issue there? Apart from the issue of transport concerning the patient | It reduced the time being wasted. This has reduced the number of patients. You don’t know what will happen next, so we have to focus our mind towards this area too, we will ask ourselves, what if we have more patients tomorrow, that we need to plan ourselves well before any other thing. |
| Okay. I know you have mentioned this but I will still go around to ask it again. The advantages of this decentralization process | Well, it brings service close to the people, it reduce their time spent travelling to Uyo. In fact, I look at it to be one of the areas that we should find means to sustain and go beyond where we are now, like every other services are getting closer to the people. Patience been close to the facility can make them to have that confidence on what is happening to them. |
| Any disadvantage? | Of decentralization? |
| Yes | The amount spent may be much (if you want to look at it that way). It involves money. That is what I am looking at |
| That’s good enough | Yes |
| What kind of changes did you make in order to accommodate this decentralization process? | ………………………… (silence) |
| Did you make any adjustment? | Actually, I made |
| Can we know the adjustments you made? | The adjustment I made, like in the area of transport, I don’t look at the cost because for one to be paying 1000 to a distance, just for us to have that incentive, whatever we can do to ensure that the patients are comfortable. |
| I want to know the adjustments you made in this decentralization process. | Like in area of treatment, there are times that we give patients injection in their different homes, within that period of being on treatment, I am thinking that if the drugs can be a little bit closer to the patient for the number of days, in as much as the patients adhere to the treatment, it doesn’t affect it, it does not affect me personally.The only thing I am interested in is for them to be cured.  The area that was of challenge is the aspect of the health workers seeing the patient taking the drugs at that time has given us a boost that if we are asked if anything has happened to the patient, we will be able to respond to that. |
| Do you think this decentralization process is complicated or is simple enough? | I do not see it to be complicated. I don’t look at it that way |
| If you say its simple, what makes it simple? | Simple in the sense that it brought service closer to the people so that at any time someone is diagnosed, he or she can then go to a nearby facility with a mere call. You will call the person and ask him or her to come to the nearby facility, he can have access to that facility. That is the area I am looking at |
| Okay. How well did this decentralization meet well to the needs of your patient? | Having baseline investigations done early which is one of the achievements of the intervention gives room to early commencement of treatment. So, it helped. |
| So, it afforded the baseline investigation to come out early | Early! |
| Any other way, any other advantage? | These are areas that I can say |
| Okay. Do you think there is need to further this decentralization? | I have said it before, there is an advantage. |
| Why? | Because we are looking at bringing services closer to the people, generally in Ikot Ekpene setting, we are doing is not enough, its just that we don’t have enough hands. These areas are supposed to be getting closer to the people in the communities. There are people that are living in hard to reach areas, everybody is not paying the same price. Automatically, and just like I said, you have to consider distance in any program, so giving them the same transport money is not proper but you will not be able to explain it to people that are not within the location. If there will be an opportunity or fund, there is need for us to do that. |
| Okay. You are in support of further decentralization? | Totally in support Sir. |
| What supports are available to the patient to help them to adopt to this decentralized DRTB treatment services? | …………………………. (silence) |
| What supports are available, either to the patient or to the health workers to adapt to this decentralised DRTB services/treatment approach? | The support is all about all of them having enough knowledge about it, like the health workers need to be updated. |
| Are there any NGOs that provides any form of support no matter how small | Yes, financial support and sometime you give drugs to the patients and they complain of not having food and knowing how toxic these drugs are, I pity them at some point not to talk of someone that does not have something doing. Once the person receives the drug, he or she cannot work throughout that day or period of time. |
| Okay; but are there NGOs that support this DR-TB decentralization? | Yes; they did, but let me say it is not enough. |
| Okay; where did they provide the support; is it transport or what? | It is the transport. |
| Okay; any other one apart from that? | Transport and communication. |
| Transport and transportation? | Yes. |
| Are they all for the patients or is there any one for the health workers? | The communication is to the health workers, then others to the patients. |
| Okay; so, they are not enough? | They are not enough sir. |
| They should be augmented? | Yes; they should do more. |
| Okay any incentive in support of the decentralization? | I may not have enough knowledge on that area. |
| Okay; did they provide any form of training before the decentralization process started? | Yes, for GX alert; we got trained on that. |
| Was it specifically for GX alert? | Yes, if I can recall. |
| Okay; apart from that there was no any other training? | We have other trainings; like in dispensing and monitoring of those patients; how to handle the patients, there was training. |
| In your facility setting or culture, did it improve or reduce the implementation of this decentralization process? | In our culture; it did not reduce. |
| What of the workability of the facility; how the facility operates; did it in any way affect positively or negatively? | I don’t see it that way. |
| Okay; so, everybody feel comfortable with DRTB patients coming here for the first time for treatment? | Automatically; it is still within our unit; when they are here, we have to talk to them. |
| What about the facility itself; did every member of the facility approved of their coming? | They have to approve because we are trying to if we can reduce the rate of the services in our facility. |
| So, you are just sure that everything fitted well? | “Long pause” |
| Sir, what have been your motivations for your support for this decentralization process? | Like I said before; I like people being themselves at appropriate time; so if that is done to the staff or the patients; at times we have work extra time; so, I feel if that is done I am a little bit okay just to support the process. |
| That sacrifice is there? | Yes. |
| Okay; how confident are you that this decentralization process will be sustained? | I may not be so positive about that because I am not the one that is supporting the project; as a worker if the project is there what stops us. |
| But from your own assessment; do you think that it is sustainable? | It is sustainable provided there are resources; both human and material resources. |
| So, you see resources as the biggest limitation there? | Yes; resources is the biggest limitation; because when material resources and man-power as human resources are not there it means nobody will operate the system. |
| So; you are also confident that you will be able to implement it? | Automatically we can do that. |
| What about your colleagues; do you think they think the same way? | I may not speak for them. |
| But from your own observation? | Few of them may fit in with the project. |
| Okay; so, how well did this decentralized DRTB treatment service fits into your own already existing work processes and practices? | Being health officer; you have to at least be looking at the way possible for us to manage the system because you don’t know who is coming to the facility; so, for you to be specific or making direction to whatever is here. All the interests is to make sure that you have these patients problems been solved. So, you have to look at it from that angle. Facilities are made for the treatment of the patients that have problems. |
| Okay; so, you are saying everything fitted in well? | Automatically, we have to; just for everything to be done. |
| So, I am coming back to that question; so, what adjustments did you make or the facility make? | Like the adjustment we made is just for us to look for the staff that will be willing to work because there in the facility they don’t have space and there is no space, there is no how it can accommodate all the activities for the program. |
| Okay; you look whether there are spaces? | Yes, like while we are here now is because it is an accommodation that we are looking for if they can put it into structures for us to be able to fit in, but in other settings; when we talk about the TB infection; people make reference to IDH or other places; why not take them to this place; so, without having a place that we can at least have focused on them is always a problem too. |
| Okay; so, that influence us to the sighting of this thing here? | Yes. |
| I have already asked this question, but let me repeat it; do we erase the old method of referring every patients to Uyo or do we keep that one like; even if you are at Ikot-Ekpeneg you go to Uyo? | I think it should be better if we get it better than what we did before; I prefer if it is closer to the people. |
| So, you don’t even support the idea of people coming from the peripheral areas coming down to Uyo to get treatment? | No!!! |
| So, you are advocating that the decentralization should continue? | Yes sir. |
| And you are also in support of further decentralization? | Yes. |
| Even though we have already seen it might be a bit challenging? | Yes. |
| Okay sir; sir is there any other thing you may want to comment on or you want to say about the entire project and the interventions? | What I want to comment is that if they can’t sustain it for now; then they will be further development just to see how to do about it because we are dealing with our people. If there is any new idea that can help us manage our people; it should be encouraged because from there we get it better. |
| Okay.Thank you for your time and for the responses you made; we appreciate that.  Thank you very much sir. | Thank you sir.  Thank you sir. |

**Title of Interview/discussion: KII/B18**

**Date of interview: 9/08/23**

| **Interviewer/facilitator/questions** | **Respondent/responses** |
| --- | --- |
| What do you think of that TB reach wave 9 project that aimed at decentralizing DRTB services? | Well, sometimes immediately some patients are diagnosed as DRTB patients, I can put it like this – stigmatization. Some of my patients in Ikot Ekpene refuse to do their baseline, they chose to do their baseline at Uyo. But I feel that they should improve Ikot Ekpene and put more equipment because I have a lot of challenges on samples because of workload, it takes almost one year before you can detect it in some of my samples. If it will be possible for the government to establish another centre here, good. |
| So, you are supporting further decentralization? | Yes. |
| Extended decentralization? | Yes Dr. |
| So that here will be a treatment centre | Yes |
| How do you think you will get health workers to work here, to take care of those patients in the centre? | If it will be possible for the government to make such provision, they will employ more people that will be here |
| Which means you are in support of decentralization? | Yes Dr. |
| Okay. Which aspect of this project do you see as the most wonderful? | The aspect I see as the most wonderful is testing at Ikot -Ekpene because there is work load at Ikot-Ekpene |
| You are happy that they are testing at Ikot-Ekpeneg? | Yes |
| Is there anything that you don’t like so much about the project? | No |
| Okay. For you now, you supported it? | Yes |
| What influenced you to support decentralization? | To less the workload. To less the work load. |
| Work load where? | If the government decides to employ more people here, the work load will be less. |
| But the decentralization brought more work to you because instead of them to go to Uyo, they will come here to meet you, that more work. Don’t you think so? | No Dr. |
| Okay. Explain what you mean by to reduce work load | As you are now, I had 30 samples in the last 2 weeks because the KNCV implemented and brought some people to work, so I had challenges on movement of sample, I called the person that is working at Uyo, the person said that I should not bring the sample IBA because they have a lot of samples there, that I should bring my sample to general hospital at Uruan. The person at Uruan said that I should call person at Ikom. I called the person at Ikom, he said Noooooooo, that they have a lot of samples, that is the challenges I am facing now. |
| How do you think the challenges will be taken care of? | On that part, government should make a way to establish that GeneXpert here at Obot Akara. |
| Remember that you are part of government because you are the Commissioner for TB in this LGA. | Hahahahahahahaha(laughter) |
| The major thing is that they supported the decentralization totally. Now, do you think that the decentralization has some challenges? Did it create obstacles and hindrances? | I think so |
| What challenges do you think it created? | You mean ……………………………… |
| Did you cause results to be delayed? | No. |
| It made result to come out on time? | Yes |
| Okay. But, I am interested in the challenges it created. Do you think it created some challenges? | Silent |
| Before we go on, let us treat this. This DRTB has about 7 or 8 components, 9 of them. We will discuss the one by one; let us see how we go about them.  One is ……… GS alert, you people formed a whatsAPP group whereby anybody that is DR-TB dictated …….. | There will be an alert………..( she interrupted) |
| What is your take on it? | Immediately the person is dictated, the GeneXpert focal person will give us alert, that this person is dictated of DR-TB from your LGA, so the DR-TB focal person will call the person in charge of the LGA to know about it and that they should go and look for the patient |
| How do you see it, is it okay? | Its okay Dr. |
| Do you think that should be sustained? | It should continue. |
| But it has stopped. Have you been getting the alert? | No. Hahahahahahahaha (laughter) |
| Do you think you have cured all the TB cases in Nigeria? | Hahahahahahahaha (laughter) |
| They have not cured all of them. | The GeneXpert alert? |
| What I mean is that there is a whatsAPP group that you people created, whereby you notify yourselves of DR-TB cases that are diagnosed, it has not been working for sometimes now | Yes |
| Do you think that all the DR-TB cases has been cured? | No. |
| Okay. The second one is improving tracking of patient through global fund funded CBOs, how did you see it? | Tracking of patient is helping us to track patient because it is important to track patients to avoid spreading the disease. To track the patients’ treatment and do other things so that other people will not be affected. |
| Okay. That one should continue? | Yes. It should continue Dr. |
| Okay. Improving counselling of patients by DRTB survivors, what the lady that left just did, do you think it is good? | Yes |
| Why do you think its good? | Dr. I am even proud of her, because through her I have about four DR-TB patients cured. Even her husband, she was the one that talked to her husband to accept treatment after being diagnosed of DR-TB. Immediately there is an alert for a DR-TB diagnosis, I will call her that someone has been diagnosed of DRTB. Some of the patients feel stigmatized after the diagnosis but she will make the person to accept treatment for DR-TB. |
| So, that one should continue? | Yes. The government should continue that one. |
| The next one is decentralizing base line investigation to peripheral areas. Instead of doing the test in Uyo, it can be done in Ikot-Ekpeneg, how do you see that one? | Ikot -Ekpene is much better |
| You think that one should continue too? | Yes, it should continue |
| Okay. Then providing transport support to patients for baseline investigations. | That one should continue also |
| Why do you think it should continue? | Dr. we cannot succeed without transportation. Even that small token is encouraging. |
| Is that so? | Yes Dr. |
| Okay. No 6, employing a mobile connectivity solution to improve turnaround of baseline investigations. I think what they were doing is that one the results of the baseline investigations are out; someone will put it in WhatsAPP group or send by email, do you think its okay? | Yes Dr. |
| It should continue? | Yes |
| Then verifying reported DRTB patients who died before they stared treatment. They will send someone there to find out using verbal autopsy. What is your take on that one? | That one should continue. |
| You are not seeing any problem in it, don’t you think that any problem will arise because of that pattern? | It should continue. |
| Okay. Then, what of engagement of a state liason officer? | It should continue |
| If you say that all of them should continue, then name the best of all thesecomponents, which one is the most ideal? | Engagement of state focal people |
| What do you mean by state focal people? | …………………………. (silence) |
| State liason officers? | Yes |
| You think it’s the best? | Yes |
| Because they were working directly with you? What about the GeneXpert alert? | The same thing |
| We want one. | The GeneXpert alert |
| Let us use this opportunity to go through these components so that if we are advising government, we will be advising government on this verycomponent, which one will the government will be in cooperating into what they already have. We will continue now with other questions.  If they tell you to compare this decentralized one with one that everybody goes to Uyo to do everything including baseline investigation, how can you compare the two? | This new method is much better because the distance is closer. For example I had a patient last year who pays transport from ………….. to Uyo, it was painful but now to pay transport from there to Ikot Ekpene will be less. |
| Apart from that, any other thing? | No. sometimes is time wasted. |
| How is time wasted? | Then in Uyo, if you go there by 6:00 in the morning, you will come back by 3:00 in the evening but now when you go to Ikot Ekpene, it will not take up to 3 hours |
| And they will attend to you? | Yes. Fast-fast |
| Any other one? | No |
| So, what are the advantages of these decentralized DRTB treatment? | The advantage is that it makes results to come out early |
| Any other one? | No |
| Does it have any disadvantage | No |
| That means you have started managing DRTB patients here now? | Yes |
| Did you make any changes or adjustments in taking care of these DRTB patients in ensuring that this DRTB services works very well? | Immediately the result for baseline is out, you raise alert with the doctor so that the patients will start taking drugs immediately without wasting more time. |
| Did you make any adjustment to ensure that things are working smoothly? | None |
| You did not make any adjustment? | Yes |
| Okay. Looking at all the components we talked about now, is there anyone that you will not want the government to touch in any way, it should be as it is? | Government should improve the stipend |
| They should improve the stipend? | Yes |
| Which ones do you not want the government to touch in any way, let it remain as it is? | ………………………….. (silence) |
| What about DRTB survivors? | Let that one be continued in the form that it is, |
| Is there anyone you want it to continue the way it is? | No other one |
| What about CBO, they should continue the way they are? | Yes |
| Okay. Do you think that these decentralised DRTB service is very complicated or simple enough? | It is simple enough |
| What made it simple? | Quick result |
| Any other thing? | No |
| How do you think your patients have benefited from these decentralized DRTB treatment? | Formerly, it usually take up to 2 weeks before treatment will commence, but now treatment can commence within one week. |
| So, it is meeting the needs of the patients? | Yes |
| What other ways did it meet the needs of the patients? | ………………………. (silence) |
| Apart from that one? | No other way Dr. |
| No other way? | Yes |
| You have answered this question before, do you think we should be decentralizing the more or we should stop decentralising and focus more on going back to that old method of everybody going to Uyo? | I do not understand |
| Will you support us or do you see the need for us to bring out more DRTB treatment centres apart from Ikot Ekpene, Eket and Uyo? | …………………… (silence) |
| You have already said that you want a centre here………….. | Hahahahahahahaha (laughter) |
| Its as if you want the decentralization to continue so that you create more DRTB centres here? | Yes |
| What do you think will be the problem if they decentralize more | Lack of workers |
| Okay, but you are in support that we should be going more deeper into the community? | Yes |
| What supports are available to patients in making sure that this DRTB decentralization project stands? I am talking about the support that the patients receive. | Is it on issue of drugs? |
| I am talking about partners like the KNCV, RedAid etc, are they supporting patients in any way? | Yes, going for OPD, they usually give them transport stipend |
| Which agency gives them the money? | I think KNCV |
| Is there any other support? | Angels baby home |
| Do they give support? | Yes |
| They give support? | Yes |
| Which people again? | RedAid |
| Are you sure they are giving anything to DRTB patient? | Yes |
| Then, any incentive in place just to ensure that this decentralization works? | They provide materials |
| No. is there any incentive from the government? | Hahahahahahahahaha (laughter) |
| Any incentive from government? | Yes |
| Which one? | Incentive for OPD, incentive for DSNO, incentive for supervisors |
| Are you advocating for this one or is it already in place? | Its already in place |
| Okay. We can count it as support or incentive. So, they are giving you money every month. | Not every month but quarterly. |
| I will tell them to start giving me | Okay |
| How do you think that the setting up of these facilities helped in the decentralization project or working against it? | It is helping |
| How is it helping? | It is helping because there is no longer delay in results |
| What do you think that motivates you well towards the success of DRTB project? | Motivation? |
| Yes. What motivates you to do this work? | Since it is a curable measure, it is not a preventive measure in as much as the person obeys the prescription and follow it accordingly. |
| How confident are you that these DRTB decentralization will continue | I don’t know Dr. |
| Why did you say that you don’t know? | ……………………… (silence) |
| No reason? No reason? | Yes |
| What about other health workers, are they confident that the decentralization is working? | They said that it should continue. |
| How has this decentralization fit into your working plan? | I don’t understand |
| How has these decentralization which has made people not to be going to Uyo anymore for treatment, how has it fit into your work plan? | It makes us to have success rate in our work |
| What do you mean by success rate? | Easy result. Easy starting of treatment for the patients. |
| Withing your work schedule, you have accommodated all these ones now? | Yes |
| All the thing you have been doing and the new assignment? | Yes |
| Do you think one of the centres should be closed down or let the two be? | Let the two be, so that those people around Uyo will benefit from Uyo while those here will benefit from here |
| Why do you want the two to remain? | A centre at Ikot Ekpene and a centre at Ikot Ekpene so that those people around Uyo will benefit from Uyo while those here will benefit from here. This is because if only one centre should stand, it will be difficult for us to achieve the needed results |
| Okay. Is there any other thing you want to tell us about TB reach wave 9 project? About the idea of treating DRTB patients in the community. | That stipend for treatment of TB survivor should be increased, and transportation for the patients to be increased too. |
| What about for the TBNS | That one is God’s work |
| Okay. you will take your money when you go to heaven? | Hahahahahahahaha(laughter) |
| Okay, so you want money for treatment supporter? | Yes, treatment supporters and TB survivors |
| Their money should be increased? | Yes |
| Okay. Any other thing? | If possible, hey should give TBNS motor |
| Motor! | Yes |
| You will say it when you go to Abuja for that meeting | Okay |
| So that they will give you motor. | Okay |
| If they give you people motor, how will you fuel it? | God will provide |
| Okay -okay-okay. Thank you very much Ma. | Thank you Sir. |

**Title of Interview/discussion: KII/b19**

**Date of interview: 8/8/23**

| **Interviewer/facilitator/questions** | **Respondent/responses** |
| --- | --- |
| What do you think of that TB reach wave 9 project that aimed at decentralizing DRTB treatment services? | It has been a service that has communicated a lot to me, help me to guide patients on how to take their drugs. It has been helping in tracking them and follow them up in ensuring that they are taking their drugs. |
| Will you consider the project a successful one? | Yes, it is a successful one. |
| Why do you think so? | Because of the numerous ways it has come to help us to track patients. |
| Just give us an idea of the numerous helps | Like I said before, tracking of patients, visiting them, the OPD activities, transportation for patients, encouragement, counselling and every other thing. |
| By now we have already noted the components, which of these aspects that you have mentioned impressed you more | The aspect that impressed me most is the home visiting for the patients and also the tracking of the patient. |
| Apart from that, any other one? | Generally, most of them have been complaining about transport to pick up their drugs, transport to come here and some of these have been taken care of by the program. |
| What are the one that has been taken care of by this program? | Giving them transport for OPD, it helps to motivate them. |
| Are there some aspects of these intervention that did not do wonderfully well, if I may say that one? | Well, I have not really seen the area that they did not do well, but I am happy to hear that its RedAid that is handling a certain program ……. or skin testing and all that but they did not reach out to every other LGA |
| Okay. We will ask questions to find out whether they have limited coverage. So, what really influenced you to support this aspect of decentralization? | As an organization that are coming in to help, we want to know what they have come to do for us and it is not a must that they will do it. |
| You personally supported the decentralization process, what exactly influenced you to do that? | What really influenced me to do that is when they chose supporters, I cant even remember names of those people that were chosen from different LGA to support |
| Are you talking about the liason officers? | No |
| The CBOs? | Yes, they really picked interest on it |
| So, the CBOs actually help very well? | Yes |
| Is it because of what the CBOs were doing that made you to support the project or what? | Yes |
| Were there challenges associated with the implementation of this decentralization project? | Are you talking about difficulty from the program or the difficulty that we faced? |
| From everywhere. Either from the program or from your side or on the part of the patient | The major problem that we usually have sometimes is the distance of the area where they are living and how they will be able to access their drugs. |
| Most of them live very far from the health facility? | Yes |
| Apart from that, any other one? | Apart from that, their own complain is that the drugs are plenty and they do not have enough food to eat to enable them take their drugs. |
| Okay. Let us discuss the components of this intervention. One of them is making the GeneXpert alert to include supervisors. I understand that it didn’t work very well but I know that all of you created a WhatsApp platform where all diagnosed DRTB result will be made available, how did you see that aspect of this intervention? | I do not understand. |
| You know that if a result of a diagnosis is out, the machine will save an alert for the program manager and other people on the higher scale. Unfortunately, when that program was developed, they did not bear DRTB in mind to receive that alert, part of this intervention is to include …….. in receiving that alert which comes straight from the machine, so when that connectivity issues superseded, what they did was creation of that whatsAPP group so that people will be receiving alert and also encouraging the NAP staff to forward the alert anytime there is a diagnosis. | It was really of great help to us because sometimes we do take our samples to certain lab, until you get there, that is when they will know that this intervention that they will be notified in other for us to pick us the results and take the necessary action. |
| Do you think that was successful? | Yes it was |
| Do you think that should be scaled up nationwide? | Yes it should. |
| Do you envisage any challenge, assuming the national TB program want to scale up, do you envisage any program or challenges because of implementation of that aspect? | I do not. |
| Okay. Next one is improving tracking of patients by supporting the global fund supported/funded CBOs, how did you see that? | It was of help |
| How did it help? | Before this intervention, we were the one that usually go out to implement and initiate treatment but through the help of the CBOs, they can assist us by taking them to baseline investigation and other... |
| The next one is improving patient counselling by using DRTB survivors, how did you see that too? | It was also of a huge help to us. Sometimes they say that two good heads are better than one, when you counsel from this angle, they counsel also and bring in their contributions from different angles. It helped us in handling the patients. |
| Is it possible that this could be scaled up nationwide? | Yes, it should |
| Why should it be done? | Because of the help, the assistance, the encouragement that they have brought |
| In your facility here, did you have any DRTB survivor that was helping to counsel patients before the initiation into this treatment? | Not directly in this facility. In another facility. |
| And the person was willing to be coming? | Yes |
| Okay. The fourth one is decentralizing baseline investigations to peripheral centres. I know that there is a private lab in Ikot-Ekpeneg that does something. How do you see that? | We have a lab in Uyo that is helping us to carry out investigations |
| Yes, you are still within the jurisdiction of Uyo | Yes |
| Providing transport support for patient during baseline investigations. You said that one helped? | Very well Sir. But most of them, when you call them to come for baseline investigation, they will not want to come until you tell them to come that there will be transport support for them, then they will come. |
| Did it help? | Yes, very well |
| Could it be scaled up? | Yes, it should be scaled up |
| Any limitation to the scaling up process? | …………………………… (silence) |
| You are not seeing the money involved as a limitation? | Hahahahahahahaha (laughter). Money will come |
| Money will come? | Yes |
| Okay. Then verifying ……………. Using verbal autopsy, did you witness this at all? | Yes |
| Then decentralizing treatment initiation to the level of Local Government Area. Did you practice this? | Yes |
| Do you think its of help? | Yes |
| And then, engagement of a state volunteer Liaison officer, did you come in contact with state liaison officer? | I do not understand this part |
| Okay. This project employed a state Liaison officer who was moving round and coordinating the activities of the TB Reach Wave 9 Project, so I am asking you whether you came in contact with the state Liaison officer during the time | Yes |
| How did she assist you in your work? | The SLO assisted us in our works in the sense that immediately an alert is sent to them, then she will follow us bumper to bumper to make sure that the patients commence treatment immediately. |
| So, the bumper to bumper worked very well? | Yes |
| Okay. So, we are now having a decentralized DRTB services but before now, everybody was doing a more centralised fashion, where every patient was expected to come to Uyo for anything that is related to DRTB, now it has been decentralized, how do you compare the two? | What do you mean by decentralization? |
| You know you are still going to Uyo? | Yes |
| What they did is to make patients that are within Ikot-Ekpeneg axis to be using IDH | Yes |
| And patients within Eket to use Emmanuel hospital, now we have three centres within the state and their OPDs were done in these various areas, it prevented or made it possible that people do not need to come to Uyo for most of the activities as it pertained to DRTB treatment, so I am asking, what do you have to say about comparing the two program options? | What I have to say is that the decentralisation has helped a lot because before now, everybody will have to go to IDH for all the services but now they have centres that are a little closer to the area. |
| In what way did it help us? | It helps to reduce transportation and the service was closer to the people than it used to be |
| What do you call the advantages of this decentralization project? | …………………… (silence). The benefit of the project? |
| Yes, to the patients, to the members of the family, to the health workers, to the national program. What are those benefits? | The benefit is that, like I said about the transportation, two persons would have paid for to go to Ikot Ekpene has been reduced especially now that fuel prize is high, they will say that they will not be able to go, let the patient go by themselves but now that it has been located in a closer area, the supporter can back up the patient to enable them to attend. |
| Okay. Any other advantage? | Aside these ones. It saves time |
| It saves time? | Yes |
| Patients time or health workers’ time? | Both. The tendency of travelling down to Ikot-Ekpene has been shortened |
| Okay. Any disadvantage? | I will say that there is no disadvantage, but had it been it is brought down to the LGA level, it would have been of more advantages. |
| So, you are supporting further decentralization? | Yes, there are some patients that are in Uyo, yet they are still complaining. If there is any way they can bring it down to LGA level, it would have been better |
| Do you think that the LGAs have such capacity to handle that? | Yes |
| Do you think there will be enough NAPs in all the LGAs able to perform most of the test if not all but the basic ones that are required as baseline investigation? | In some LGAs, we do not have enough health workers that will handle those things. |
| That means there will be some limitation if we want to do further decentralization of this program. | Yes |
| Did you do any changes or adjustment to be able to handle this decentralization project? | We saw the opportunity and we embraced it. |
| Those components that we discussed, which of them do you want the system to accept completely (100%), no changes? Remember the involvement of GX alert, using of DRTB survivors as counsellors and provision of support for baseline investigation and then having a state volunteer liason officer. Is there any of them that you will prefer that it will be adopted the way they are into to? | If I am asked the way you are asking me, I will say that all of them should be accepted the way they are. |
| All of the | Yes |
| Okay, if you are to name one as the best, which one will you mention as the best? | I will name transportation for baseline |
| And? | GX alert |
| GX alert because it involves you directly? | Yes |
| Do you think that this decentralization process is complicated in any way? | No |
| Do you think its simple? | Yes |
| What made it simple? | That you will get an alert of a DRTB patient, it is your work to go and look out for the patient. The next thing is to go for investigation, then after the investigation, you go for counselling and the treatment will commence. |
| You feel it is seamless, it is simple enough, it is straight forward? | Yes |
| Okay. What support do you think were available to the patient during these decentralization project, maybe from NGOs or from any other body | The transportation supports that are being given to the DR-TB patients during OPD |
| That is one of them, which agency was providing this support? | Angel’s Home Foundation |
| Any other one? | No other one |
| Okay. Any incentive? | No other incentive apart from that |
| What about the health workers? | There is always transportation for health workers that are coming to provide services? |
| Which agency is responsible for that? | KNCV |
| Okay. Is there anything within your LGA, both in setting, working formula in your LGA or culture that you think that it worked against the decentralization or that helped in the decentralization project during that period of implementation? | The culture that is affecting us right now is the culture of a certain season like this season……………………….. (stopped) |
| Farming? | Not really farming |
| Raining season? | Not raining season |
| What is it? | Its Ekpo festival |
| Masquerade? | Yes, if you are not a member and if you are not initiated in it, you cannot come out and women cannot be seen around there and they cannot move freely. |
| How long does it last? | Just a month |
| A whole month? | Yes, in the month of August |
| What do you mean by not move freely, you are here now? | Yes. If they want to bring out the Ekpo, a woman is not allowed to be there, even some men except if you are a member. |
| Any other one, maybe about the facility setting? | No problem with the facility setting |
| Even though there is no road to enter here? | No. there is a limitation because the road leading to some of the facilities are bad. |
| Okay. It’s alright. You have technically answered this but let us discuss it. Do you think there is actually need for further decentralization? | Yes |
| What is the reason for that? | I believe that if it is more closer to the people, we will have better results. |
| You even went very far, you are asking for the decentralization to take place in every LGA, how sustainable can that be? | I believe it will be sustained through the help of Global Fund and other NGOs that are helping us |
| Okay, the major thing is for the services to be closer to the people? | Yes |
| Okay. What has been your motivation in supporting the decentralization of DRTB services? | Is it the motivation of survivors? |
| You as a person, that has made you to be up and doing in this decentralization process | Me as a person. What gives me pleasure is to get those patients healed and for them to take their treatment at the right time and drugs should be available for them |
| Okay. Any other thing? | No |
| How confident are you about implementing and sustaining this decentralization process? | ………………………….. (silence) |
| Are you confident that it will be sustained? | Yes |
| What gives you that confident? | What gave me the confidence is that it was not so from the beginning it was not so, they used to go to only one centre, the way they have been able to bring it to the 3 senatorial districts, so I believe it can happen in all the LGAs |
| So, you believe that it could be sustained? | Yes |
| Do you think that other health workers share this view that it should be sustained? | I believe they could |
| Alright. So, how well did this decentralized DR-TB services fit into your already existing work pattern and practices? | How it fit? |
| Yes, the other time you said that there was no adjustment made, does it mean it fitted perfectly well into what you do? | I will say “Yes” that it fit in perfectly in my work |
| In any case, going back to that other first question, you didn’t make any wonderful adjustment to be able to accommodate anything that come up in the decentralization process. | ………………………. (silence) |
| Yours is different because you have been going to Uyo and you are still going to Uyo. Some other people have been told to move down to Ikot Ekpeneg, do you understand, its as if the statuesque remained the same for you | Yes |
| Now, we have a decentralized system already where patients go to Uyo, Ikot Ekpene and Eket, do you think that this process should replace old one where everybody is coming to Uyo or do we allow it to decentralized, which one is better? | I think that going to different senatorial zones will be better because the patient will go to centres that are closer to them |
| That means they should scrap the old method of decentralization? | Yes |
| Is there anything that you think you want to let us know as regards this DR-TB Reach Wave 9 Project? | The only thing is, if there is any way they can improve their transport, it will help them. |
| So, this transport is really a challenge to them? | Yes |
| If they want to improve it now, how much do you think will be appropriate to pay? | I wouldn’t be able to mention any specific prize. But if you look at the condition of the country someone can decide on what should be given. |
| How much are they receiving as transport? | I can’t say |
| Okay. No problem. Apart from that, any other observation? | No other one |
| Thank you for your time and audience given to us, we appreciate. Thank you very much | Thank you Sir. |

**Title of Interview/discussion: KII/B20**

**Date of interview:**

| **Interviewer/facilitator/questions** | **Respondent/responses** |
| --- | --- |
| How do you relate this decentralization process whereby patients don’t need to go to the state capital for base line investigation and what we call monthly opass. In that they now in akwaibom state specifically, they have three cites, Ikot Ekpeneg, Oromand Eket where patients now do most things they ought to have done in Uyo which is the state capital. How did you see that sir | Actually when this, I will call it innovation came, I looked at it as a very interesting innovation because judging from the fact that you are dealing with very poor people, who need to access the service travelling from far end to where they are identified to Uyo will require so much for them to go to the place and access service as well as the issues of being exposed to unnecessary road traffic problem, then outside that to avoid such, these patients could access this care and the result will come as soon as possible because they are able to access with the area where they are identified rather than looking for an opportunity for them to think of going to that place because they will now be scheduled before they can go to Uyo which is farther than where they are staying but when they are to access this care within the area they are staying it shortens the duration of accessing the care. |
| Ok, thank you.What do you like must about that approach, that decentralization process, what is wonderful and fascinating about it? | The prompt attention to patient and making the results of the baseline available within a short period of time |
| Okay. Do you think it has a low side, some aspect of it that is not so wonderful, I mean the program or the project entirely. | Well, I will look at that, that it depends on who is actually judging the Negotiation side, of it is the patient, I think is much of negatives in it. For the patient to access the treatment within the region the funders will think of spending much because of going that far. |
| Now as a private person, what influence your support for this decentralization process. Why did you express your support for it by being involved. | For one TB, is a thing that can affect everybody and anybody is being part of the team that can resolve matters concerning the particular ailment is a great joy to me to be a part of solidary. Their problems as soon as possible. |
| So that help you, that was the most important aspect that made you be | Yes. That is one of them |
| Okay, are there others too? | Ahh yes, I would also consider the fact that being in private, I see it as a recognition of my contribution as a private person not necessarily the government that are to have my input as a private person in such a project |
| Okay. So did it came with challenges may be in the process may be in the process of signing whatever memorandumof system they must have presented, the type of test the program requires of you to do, all these things GM | Challenges in terms of?  Exactly, for me, the challenges in that aspect came from the fact that some of the tests that are required for me to do, I was not able to do them I had to go the extra mile to get the materials so as to perform the tests and more importantly too, the pricing is far far below the general pricing in the private sector and it became worse when the economic difficulties emerged last year, the cash crush, then the prices of the reagents doubled and tripled but we were still asked to charge as low as what we were still asked to sign for. Theirs is also the issue of being exposed to TB, when the members of staff in my form realized that we were having DR-T patients, they were frightened to that stage that when TB patients come they will prefer that I should handle them, it was really challenging. |
| So how did you cope them, how did you cope with all these challenges cause I see them as numerous. | For the aspect of my staff, I had to set them provided them with the necessary protective materials like the face mask, to be using , but for the pricing, honestly it is a thing that , infact it has really gotten me so-so down in trying to provide the materials that I am supposed to use for my regular practice, using them to do the test that at the end of the day when you realize that there is no gain. I only have the joy because I participate, particularly because I was posted as a government staff to IDH and I stayed there for up to 10 years in IDH, so I became part of that system to handle TB patients, so that is the major thing that gave me the backing to continue. When once I see such patients, I always feel for them because if I were to say since things are the way they are let me just back out it wouldn’t all go well, even up to this day. Look last week or two weeks ago, I got my regular patients, whose sputum was sent for GeneXpert and discovered, he was high MTB and I called TBLS for the patient to start mediation. because I hear you have to let the program know their status, I don’t consider that, I do that on my own and I could recall another one, there is a young girl that came in with funny issues, I think she is a sickler and she was coughing, so I took the pain to collect the sputum and went to GeneXpert did the test which was MTB detected, so I also had to link the TBLS and the patient was placed on treatment the Father was so-so happy, so changing system is really a big issue for people. |
| Thank you very much sir for chose explanation. Let me go to these aspect of the component of the program that directed affect you. One is the decentralizing the map investigation to peripheral labs like yours. Now if the nations TB central program to scale this up, do you see it as a very good approach, do you see it as a good step to take. | Scaling up as in form of |
| You know this is more like a centered study done in Akwa Ibom and Oyo state. So, assuming from the result, because all they were doing, the aim is to reduce the lost to follow up or noninitiation into treatment for DRTB patience. Just to function like process rather than we have DRTB patience and they still remain in the community coughing and all that so assuming they are going to do it, do you see it as a very good step, that the only one we should or could adapt. | Actually, in one of the statements that I made I strongly wish that the program should be scaled up to that extent, why because TB like I have said before is a disease that can affect anybody, anywhere, so if the program is scaled up in such a way, we will be able to tame down the spread. When these patients are taken at the prime time and the results are available and treatment is given on time. With time it reduces loss to follow up because if I say I am in Akwa Ibom and may be nothing like that happening in Lagos or Kaduna, then there is no problem, why, it is a big problem to us here, because our people will go there and return, if the same thing is not done there, when we are trying to solve a problem here, they will now keep bringing from those areas this kind of intervention is not there down to where the intervention is said to be going on and making nonsense of the program in this area. So, I will say it is a very good idea it is scaled up to areas especially where we know there is a high volume of TB cases. |
| Okay. If the scale up as a good idea that it is the same problem, the same challenges, you talked of may arise, how do you think, what other problems do you think or the problem that could emanate from that scale up process and how do you think the national TB program or any other person like you should handle, these challenges what are the current challenges and what can be done to handle it? | Look the challenges that we have already talked about. |
| Yes. | In as much as we are in a situation where by, we would want things to be better in anything that we are doing as business, I believe that these areas where the intervention would have been scaled up, if they face such challenges, they could also make little sacrifice so as to sustain the program in such an area. For instance like the changing, one might not need to have so much of a gain out of it but if still the economy will warrant increase in pricing, I think if funders outside, the care funders, if interested people could fall in, to assist the funding to assist the national program in funding the program, it will be better to sustain it but in a situation where by we don’t have finances to assist I believe those who are in the program should be able to bear some losses to sustain the program. |
| So, the next one is employing and mobile connectivity solution to improve turnaround time associated with base line investigation; I don’t know whether you practice it here | That innovation, I have engaged it so much, right from during primary training and now the implementation. Immediately for instance, if I do my test now, I simply go to the DR-TB IMS platform and key in my results and with that all participants could view the results in a matter of minutes or hours of inputting the results in. So, it is a very good innovation, I have challenges concerning it. The only challenge is that I understand that some of the key factors in the program were unable to access the IMS platform and that warranted after inputting it into the DRTB, IMS platform, I will also snap the results and send directly to the key people involved. |
| So that is a lot of sacrifice | Yes, that is the challenge that I saw. I have been denied of it, there is sometimes I was asked, I said I have already inputted my result oh so and so person is unable to key into the platform, so the only option was to snap and send via WhatsApp |
| So, you see that has a very good approach | Very good indeed if there is no challenge in maintaining that platform, I don’t know, you know these that are handling the app, I don’t know how they do it, but I hope they don’t have challenges in maintaining, but far us or for me using it is very great. |
| So, you don’t see any problem with that in a scale, assuming them is going to be a scale on that approach | That will depend on the level of those who are involved in this ICT issues for instance my colleagues in other states, if they don’t have knowledge in using such platform they might have issues with inputting. But I believe if they are properly trained on how to use and they are enlightened, that challenge might be reduced. |
| So, we are back to the main project as we have disclosed the complement as it remains to you as we are specific on the tool and I’m happy for this light you threw on that digital connectivity network. You see before, am sure you are aware that all our death patient were going to the central area and now we decentralize, can we compare the two system. Any different between the two? the centralize and the decentralize. | I will still continue to say, for the patient at this centre, decentralize is a positive but I don’t know whether the funders, whether there is any challenge with the decentralization but I will say as far as the patients are doing their baseline as fast as possible, decentralization is very good and is comparatively better than centralization. Central place, one place for all the state, transportation cost, especially now, currently transportation cost is the terrible apart from I still say, it’s exposing the patient and again the care giver who brings them to unnecessary road issues |
| Okay, you have already talked about advantages of that system, so I will skip that, now I think you have hinted on disadvantages now. What changes did you adapt to fit into these programs.You know, adjustment you needed to do in other to fit into the program | Like trying to acquire what I did not get, like I want to be specific, like doing analyses, I was not doing analyses, but when I saw that for baseline test that amylase is there, I had to immediately include it, though generally my machine does any type it’s only differences is just the reagents so I had to look for money and then gather the materials. |
| Okay thank you. So, this decentralization process, do you think it is complicated in any way? Or simple and straight forward | Except for instance, like let me say my experience, one of the local governments, the Zone that I am here, I have not been getting patients from there, they still go to Ugo so I don’t know whether that could be complication but for me, I still insist that patient within the region could have better access to care than going from wherever to one place and one place might be overwhelmed by so many patients and the turnaround time might be more prolonged than necessary but the decentralization tends to reduce the turnaround time to minimal acceptable period. So, I wouldn’t think it is a complication except of only you will make me know the complications. |
| So,Sir it is obvious you are in support of the decentralization what about further decentralization, how do you see it? | If the funders evaluating the whole system of further decentralization sees it could be convenient and they wouldn’t have challenges, well and good, cause we still want to get the patients reach care as soon as possible, so if there is a program chat can do that and would not warrant extra cost, why not. |
| You think it will be a better approach. Like making the patients arriving at their own door steps so to say. | Except may be the only challenges I will consider, I don’t know is same people look at TB as funny sickness, so doing within the area that are so familiar might bring stigmatization and sometimes, the patient might not want to do it within the area that they are well known. So I think that might be a challenge that might not be so necessary to take decentralizing as far as ……… |
| Even getting equipment like yours beyond this fare, I think it might still be very difficult do you understand, getting a lab of this standard to beg and the reach where we are now, might be another negative point because it may be very difficult, even see the adjustment you made | Exactly because for one to get the kind of equipment to do these kinds of test, it will be a waste if they don’t support. |
| Sir, we are going to talk about your motivation what motivated you, we will skip that so. Now you have talked about your staff, about them being apprehensive at the initial stage and the other aspects of your center that are evaded or hinder to a little extent no matter how little. This decentralization process because it is the first time they are coming in contact with dead patients and it was like which one is this? but theyencountered that, so are there other issues, observation that aided the system here, no matter how little, hindered the process to. | I don’t think, assisting the program on decentralization was at least seeing that even with the challenges that we are able to key into the program without opting out but to say what could have presented itself as a negative feedback to the decentralization, may be the initial stage trying to get all that test because at first whatever they needed for a center we could not get them, see o I don’t know whether that could have been at the initial time, until we were able to put ourselves together to make sure that all the things that were needed in the baseline are done there otherwise I don’t know, because we have not been involved anyway of our activity that was not required. |
| Now you have spoken so well of this decentralization, and confident that this thing is sustainable, this approach, this decentralization approach, how confident are you that it could be sustained | I think that lies entirely on funding, as far as there is that commitment to fund, I think it will be sustained except there is shortfall of funds to manage it has it is. If there is that continuous funding for the program, I believe it can be sustained except for the financial challenges |
| So how well sir does the decentralization process fit into your own existing process and practices | It fits so easily.With the challenges that I have mentioned, it is only that I was not encouraged, state quality assurance office kept telling me, at least you have been in the program so continue, he said thatwhat we are going to face it has a challenge and that I take whatsoever may be hopefully in future, may be not minding how it is now, it might be better, I said okay. I know the good thing I have seen in TB than when I came into TB, there was dairy TB microscopy for diagnoses and that I know out of what the program and its international finders saw that was coming and of microscope diagnoses to GeneXpert that I also believe if are faced with challenges of trying to do this program that it might also bring good things on the future that was the thing, that was in my mind because, as I have already said, you know tomorrow I will tell people that I used mirror to examine microscope and diagnose TB, because like yesterday we had challenges, so for us to keep the program running, we had to use mirror and examine three samples, for diagnoses and two samples for follow up. But that now people who are in TB if they are hearing that they are now using GeneXpert, so their initial challenges was that things that are good is always a good thing to do, hoping that they will bring better results. |
| But sir this decentralization process, do you think it could influence the whole model of going to Uyo everybody or should it complement it, or how did you see it? | I think having the two together tend to be having confusion, for what kind of patient will keep going to the central place and which patient will be going for the decentralized care? so I believe it’s either depending on the fact that, it’s either decentralization or the previous ways. But having the two together it will look confusing as who are to go to. If the two can work together, if there will be an outline of how that should run otherwise, they have to follow one pathway but if the funder look at the program and saw it as a positive one, according to their aim and objective, why not ? |
| Sir, this is a very great insight you have thrown for us especially the experience working with the three samples for diagnosis with Minor and microscopy and the present day GeneXpert. Is there any other comment you would like to say both on DRTB to and the decentralization process, my own question are finish any comment from you? | Yes, firstly I am happy that this program of handlingDRTB is set in place and filtered very slowly because of the impact that is very obvious, it’s allowed in the society because I know TB in first place ordinarily was seen as a disease that cannot be healed, but now people are of a know that it can now be treated, but talking of DRTB in the society is very scary. So, the intervention, reducing DRTB is very commendable and appreciated, I would say more enlightenment for the rich people to be aware of this kind of program, so that it is possible they can key in to assist to make sure that, access to care by the DRTB patients increases and also apart from trying to eliminate the DRTB, the DRTB case should be as much as possible reduced completely because of the impact it brings to the society |
| Thank you very much for your time and the insight you have thrown in this. | Thank you Sir |
