# Supplementary material for: Enhancing programmatic scale-up: Applying the consolidated framework for implementation research to evaluate decentralized drug-resistant tuberculosis services in Southern Nigeria
Source: PLoS One. 2025 Feb 21;20(2):e0318274. doi: 10.1371/journal.pone.0318274 (PMC11844838; doi:10.1371/journal.pone.0318274)
Supplement: Attachment — Submitted filename: Response to Reviewers.docx [file pone.0318274.s005.docx]

Department of Community Medicine,

Alex-Ekwueme Federal University Ndufu-Alike,

Ebonyi State, Nigeria

19/12/2024

**Re:** **Enhancing Programmatic Scale-Up: Applying the Consolidated Framework for Implementation Research to Evaluate Decentralized Drug-Resistant Tuberculosis Services in Southern Nigeria**

Dear Academic Editor,

Plos One

Thank you for the opportunity to revise and resubmit our manuscript. The authors appreciate the valuable comments and suggestions provided by you and the reviewers, which have greatly improved the quality of our work. Below are a detailed point-by-point response to the academic editor’s and reviewers’ comments.

**Academic Editors’ comments**

**Comment:** Please state what role the funders took in the study.

**Response:** This has been stated as, “*The funders had no role in study design, data collection and analysis, decision to publish, or preparation of the manuscript*" in the manuscript.

**Comment:** If your ethics statement is written in any section besides the Methods, please move it…

**Response:** The ethics statement has been moved to the methods section

**Comment:** Please include captions for your Supporting Information files at the end of your manuscript

**Response: This has been done**

**Comment:** Please review your reference list to ensure that it is complete and correct. If you have cited papers that have been retracted

**Response:** Done. Article no 29 in the previous reference list was removed as it is no longer relevant following revisions, while article no. 10 and 11 in the current list have been newly added.

**Comment:** Please confirm at this time whether or not your submission contains all raw data required to replicate the results of your study.

**Response:** We can confirm that all raw data required to replicate the results of our study have been submitted. The data availability statement has been revised to **‘**All relevant data are available in the Supporting Information file (S3 TRANSCRIPT)**’**

**Comment:** Can you please confirm that all participants gave consent for interview transcript to be published?

**Response:** We can confirm that all gave consent for interview the anonymised interview transcript to be published. This has been stated in the ethical consideration sub-section of the manuscript

**Comment:** please also confirm that the transcripts do not contain any potentially identifying information

**Response:** We can confirm that the transcripts do not contain any potentially identifying information. Thank you.

**Reviewer #1**

**Comment:** Please describe criteria for deciding when no further sampling was necessary in the data collection part (e.g., sampling saturation):

**Response:** Thank you for your comment. This has now been described in the data collection sub-section (page 9)

**Comment:** Please describe data management and security, anonymization/deidentification of excerpts

**Response:** these have been described in the methods (page 9).

**Comment:** Majority of the emergent themes were grouped under the Innovation Characteristics (17 of 38 themes) and Inner Setting (11 of 38 themes) domains respectively. Can you explain the possible reasons why the remaining three parts have less content identified?

**Response:** **Outer Setting, Individual Characteristics**, and **Process** may have received less attention due to the participants' roles, or the stage of implementation, where external or outer setting factors, individual-level issues, or specific implementation strategies may not yet be fully prominent. On the other hand, **Innovation Characteristics** and **Inner Setting** likely reflects their central role in the decentralization of DRTB treatment, and are often the most immediate concerns for key informants

**Comment:** In the CFIR analysis results section, some excerpts can be added to help understand the content and increase credibility.

**Response:** Thanks for your observation. All quotes were summarized in Table 1 to maintain a manageable word count. Some excerpts have now been included accordingly.

**Reviewer #2**

**Comment:** o It is difficult to follow what is the intervention and what are the implementation strategies to deliver the intervention…

**Response:** Thank you for this observation. The core evidence-based intervention was Decentralization of DR-TB treatment initiation services which we implemented as a bundle of interventions that had to be deployed at multiple levels along the patient care pathway, as listed on page 7 under the ‘description of the interventions’. Additional explanation have been included for better description. This paper presents results from the **evaluation of the** implementation experience during **a pilot project to decentralize DRTB treatment initiation (lines 119-120 of the manuscript)**. As such, the focus of this study was to assess the implementation outcomes of feasibility, acceptability, and understand contextual factors influencing the implementation of the decentralized model.

**Comment:** …first mention of the WhatsApp group – is this part of the innovation? Please add to the intervention description or clarify particularly as it a large part of the discussion

**Response:** Thank you for the observation. This has been corrected with a more detailed description of the intervention section, including the role of WhatsApp as with other components of the intervention mentioned in the ‘description of intervention’ sub-section of the methods section (page 7)

**Comment:** Some inconsistent use of the CFIR and the CFIR domain/construct terms use consistent CFIR terms (e.g. external should be outer setting?)

**Response:** Thank you for your clarification. The correction has been made, and the term "external" has been replaced with "outer setting" in the appropriate context. However, in instances where "external" was not specifically referring to the **outer setting**, it has been retained to maintain the intended meaning.

**Comment:** Don’t need to say “CFIR framework” as “framework” is already in the name

**Response**: This has been corrected

**Comment:** COM-B is introduced in the discussion – was this model used to guide analysis and interpretation of the findings? It needs to be presented in the methods and the results rather than only the discussion if it helped to guide the work…

**Response:** Thank you for the observation. The statement have been revised, and the COM-B model removed as it was not originally included in the analysis or interpretation.

**Comment:** The authors may need clarification on what availability of data means. This would include access to all fully transcribed interviews, which I don’t believe are included in full within the manuscript. Currently, the statement reads, “All relevant data are within the manuscript and its Supporting information files”

**Response:** All data relating to the manuscript have been included as supporting information named, ‘Transcript’

**Comment:** additional explanation of what maximum variation purposive sampling is (with citation) and why it was used would be helpful

**Response:** additional explanation along with a citation has been provided

**Comment:** is there a word or phrase missing? One-on-one interviews with diverse stakeholders who were directly involved in implementing the project interventions in both states.

**Response:** Thank you. This has been corrected

**Comment:** capitalization of “CFIR Interview Guide” tool suggests it came from a specific place (CFIRguide.org?) - please cite where the guide comes from

Response: Citation has provided

**Comment:** does this mean you undertook a deductive coding approach rather than inductive? Please clarify this sentence, “We adopted the CFIR constructs as our coding template rather than generating a coding template with our subset of data.”

**Response:** This has been clarified (page 9).

**Comment:** unclear how “sharing lived experience” fits in relative advantage? Can you clarify/explain more clearly what about the intervention design compared to the centralized approach makes it possible to share lived experience

**Response:** Thank you for your comment. The inclusion of **DR-TB survivors** to **counsel new cases** was a key component of the decentralized package of interventions, which distinguishes it from the centralized approach. This element of the design supports **relative advantage** by offering new patients the opportunity to receive peer support from individuals who have personally gone through the challenges of DRTB treatment.

We hope that the revised manuscript addresses all concerns raised. Please let us know if further clarification or additional revisions are required. Thank you once again.

Sincerely,

Chibuike Agu

Corresponding author
